# House dust metagenome and pulmonary function in a US farming population

**DOI:** 10.1186/s40168-024-01823-y

**Published:** 2024-07-18

**Authors:** Mikyeong Lee, Abhishek Kaul, James M. Ward, Qiyun Zhu, Marie Richards, Ziyue Wang, Antonio González, Christine G. Parks, Laura E. Beane Freeman, David M. Umbach, Alison A. Motsinger-Reif, Rob Knight, Stephanie J. London

**Affiliations:** 1 Immunity Inflammation and Disease Laboratory, National Institute of Environmental Health Sciences (NIEHS), Durham, NC 27709 USA; 2 Department of Mathematics and Statistics, Washington State University, Pullman, WA USA; 3 Integrative Bioinformatics Support Group, NIEHS, Durham, NC USA; 4 School of Life Sciences, Arizona State University, Tempe, AZ USA; 5 Biodesign Center for Fundamental and Applied Microbiomics, Arizona State University, Tempe, AZ USA; 6 Westat, Durham, NC USA; 7 Biostatistics and Computational Biology Branch, NIEHS, Durham, NC USA; 8 Department of Pediatrics, University of California San Diego, La Jolla, CA USA; 9 Occupational and Environmental Epidemiology Branch, Division of Cancer Epidemiology and Genetics, National Cancer Institute, Rockville, MD USA; 10 Center for Microbiome Innovation, University of California San Diego, La Jolla, CA USA; 11 Department of Bioengineering, University of California San Diego, La Jolla, CA USA; 12 Department of Computer Science and Engineering, University of California San Diego, La Jolla, CA USA

**Keywords:** Microbiome, Microbiota, Metagenome, Whole genome sequencing, Spirometry, Respiratory function tests

## Abstract

**Background:**

Chronic exposure to microorganisms inside homes can impact respiratory health. Few studies have used advanced sequencing methods to examine adult respiratory outcomes, especially continuous measures. We aimed to identify metagenomic profiles in house dust related to the quantitative traits of pulmonary function and airway inflammation in adults. Microbial communities, 1264 species (389 genera), in vacuumed bedroom dust from 779 homes in a US cohort were characterized by whole metagenome shotgun sequencing. We examined two overall microbial diversity measures: richness (the number of individual microbial species) and Shannon index (reflecting both richness and relative abundance). To identify specific differentially abundant genera, we applied the Lasso estimator with high-dimensional inference methods, a novel framework for analyzing microbiome data in relation to continuous traits after accounting for all taxa examined together.

**Results:**

Pulmonary function measures (forced expiratory volume in one second (FEV_1_), forced vital capacity (FVC), and FEV_1_/FVC ratio) were not associated with overall dust microbial diversity. However, many individual microbial genera were differentially abundant (*p*-value < 0.05 controlling for all other microbial taxa examined) in relation to FEV_1_, FVC, or FEV_1_/FVC. Similarly, fractional exhaled nitric oxide (FeNO), a marker of airway inflammation, was unrelated to overall microbial diversity but associated with differential abundance for many individual genera. Several genera, including *Limosilactobacillus*, were associated with a pulmonary function measure and FeNO, while others, including *Moraxella* to FEV_1_/FVC and *Stenotrophomonas* to FeNO, were associated with a single trait.

**Conclusions:**

Using state-of-the-art metagenomic sequencing, we identified specific microorganisms in indoor dust related to pulmonary function and airway inflammation. Some were previously associated with respiratory conditions; others were novel, suggesting specific environmental microbial components contribute to various respiratory outcomes. The methods used are applicable to studying microbiome in relation to other continuous outcomes.

Video Abstract

**Supplementary Information:**

The online version contains supplementary material available at 10.1186/s40168-024-01823-y.

## Background

Chronic respiratory illnesses pose a major public health burden [1]. Although exposure to microorganisms inside homes has been linked to respiratory health [2], data in adults are limited. A few studies have examined associations of microbial composition with asthma and allergies [3–5]. Continuous outcomes have been largely ignored. No large studies of adult respiratory outcomes that capture environmental microbial exposure using state-of-the-art whole metagenome shotgun sequencing have been reported.

Pulmonary function is a continuous measure of the physiologic state of the lungs in health and disease. Lower pulmonary function associates with poor health-related quality of life [6] and predicts mortality, independently of other risk factors [7]. Compared to genetic [8, 9] and epigenetic [10] factors, less is known regarding potential impacts of exposures to microbial components inside homes on pulmonary function. Lower pulmonary function in asthmatic individuals was associated with exposure to house dust endotoxin, a generic measure of gram-negative bacteria [11]. Some studies reported no significant associations of adult pulmonary function with bacterial or fungal components [12], while others found associations with specific microorganisms [13] or moldiness [14]. These studies measured microbial agents using quantitative PCR (qPCR) which limits the number of microorganisms under investigation [12–14]. Metagenomics provides the opportunity to explore a broad spectrum of microorganisms, including ones that cannot be cultured in a laboratory setting.

Fractional exhaled nitric oxide (FeNO) is a quantitative, noninvasive measure of airway inflammation. In children, exposure to endotoxin in house dust was related to lower FeNO [15]. FeNO levels associated with diverse indoor fungal communities among 55 adults [16]. We find no large sequencing-based study of indoor microbial profiles and FeNO in adults.

We used whole metagenome shotgun sequencing to comprehensively profile microorganisms in house dust from 779 households in the Agricultural Lung Health Study (ALHS), a case-control study of asthma nested within a US farming cohort. We investigated associations of pulmonary function and FeNO with diversity of microbial communities inside homes and with abundance of individual microbial taxa. We implemented a novel framework for analyzing microbiome data in relation to continuous health outcomes.

## Methods

### Study population

Participants were enrolled in the Agricultural Lung Health Study (ALHS), a case-control study of current asthma nested within the Agricultural Health Study (AHS), a cohort of farmers and spouses of farmers in North Carolina (NC) and Iowa (IA) [17]. Details have been described previously (data version P3REL201209.00) [11, 18]. In brief, the ALHS enrolled 3301 participants (1223 asthma cases and 2078 noncases) in 2009–2013. Of these, 2871 received a home visit at which bedroom dust was collected. Of a simple random sample (*N* = 1000) chosen for our previous 16S rRNA amplicon sequencing study, 879 samples passed quality control and were included in our previous dust microbiome analyses [19]. These 879 samples were subjected to whole metagenome shotgun sequencing for more accurate comprehensive characterization of microbial communities.

### Respiratory outcomes

Measurement of pulmonary function and FeNO in ALHS has been described previously [20]. In brief, trained staff measured prebronchodilator spirometric parameters, including the forced expiratory volume in the first second (FEV_1_) and forced vital capacity (FVC), during in-home visits using an EasyOne^®^ spirometer (NDD Medical Technologies, Chelmsford, MA, USA) according to American Thoracic Society guidelines. Participants were asked to avoid use of bronchodilators for at least 6 h before the visit; only 3% of participants failed to comply. We calculated FEV_1_/FVC as a proportion ranging from 0 to 1. FeNO was measured using NIOX MINO (Aerocrine AB, Solna, Sweden) following manufacturer guidelines in duplicate and then averaged. Values below the limit of detection (LOD; < 5 ppb; 5.1%) were assigned to LOD/sqrt(2) = 3.5 ppb.

### House dust collection and whole genome shotgun sequencing

Trained field technicians collected dust samples using a DUSTREAM^™^ Collector (Indoor Biotechnologies, Inc., Charlottesville, VA, USA) from participants’ bedrooms during home visits [11]. Technicians vacuumed a one square yard area on the sleeping surface and on the floor next to the bed. Details on dust samples and DNA extraction were previously described [19]. Extracted DNA samples were sent to Center for Microbiome Innovation, University of California San Diego, for whole genome shotgun metagenomic sequencing using Illumina NovaSeq (Illumina, Inc., San Diego, CA, USA). Processing included (1) trimming of low-quality sequence reads, duplicates, and adapters based on FastQC results (v0.11.5) [21] and (2) identification and removal of potential contaminant sequence reads, not from microbial genomes but from host genomic sources (human, cow, pig, chicken, turkey, horse, goat, sheep, dog, cat, and dust mite) (Table S1) plus PhiX, a spike-in control in an Illumina experiment, using Bowtie2 [22] and KneadData (v0.7.10) [23]. We obtained taxonomic classification of sequences using Kraken2 (v2.1.1) [24] and generated abundance (counts) for each taxon using Bracken (v2.5.0) [25] with RefSeq genomes for bacteria, archaea, eukaryotes, fungi, viruses, and plasmids and NCBI taxonomy information. Additionally, we identified and removed sequences related to potential contamination from sample collection and laboratory reagents (168 taxa) (Table S2) using negative “blank” controls of sterile water and the decontam R package (v1.10.0) [26]. We used both frequency-based (the default threshold of 0.1) and prevalence-based (a stricter threshold of 0.5) methods. For further analyses, we excluded 98 samples having sequence reads < 1000 and taxa having < 0.0005% of the total number of sequence reads across all samples [27, 28] or assigned to Eukaryota and viruses with limited RefSeq genome databases available, leaving microbial abundance data for 1264 species (389 genera) in 781 samples.

### Overall microbial diversity in relation to respiratory outcomes

We calculated two measures of overall microbial diversity within each sample (alpha diversity): richness (the number of individual microbial species) and the Shannon index [29], which reflects both richness and relative abundances of each species. Using linear regression, we evaluated associations of the diversity measures with pulmonary function parameters (FEV_1_, FVC, and FEV_1_/FVC) or FeNO. Due to its negatively skewed distribution, Shannon index was exponentially transformed before association analyses. Covariates for pulmonary function were age, age squared, sex, height, height squared, weight (for FVC only), cigarette smoking (former or current, both relative to never), cigarette pack-years, asthma status (yes/no), state of residence (NC/IA), and ancestry (European/not based on genome-wide genetic information, except for one sample whose information was filled with self-reported race as White). Models for FeNO included abovementioned covariates except age squared and height squared. As sensitivity analyses, we additionally adjusted for season of dust collection (winter/not) which showed associations with overall microbial diversity in ALHS [19].

To avoid bias due to different sequencing depths among samples, abundance data were rarefied to the minimum number of sequences (975) across samples before assessing microbial diversity. After excluding two participants without smoking pack-years, 779 were included in association analyses. FeNO was available for 767 participants. We used R version 4.1.0 to summarize characteristics of the study population and perform association analyses of microbial diversity. We used functions *specnumber* and *diversity* in the vegan R package (v2.6.2) [30] to calculate the richness and the Shannon index, respectively. We set *p*-value < 0.05 as the threshold for statistical significance in diversity analyses.

### Individual microbial taxa differentially abundant in relation to respiratory outcomes

To examine differential abundance of individual taxa in relation to pulmonary function or FeNO, we applied statistical inference techniques that provide accurate tests of hypotheses in large-scale data sets with high-dimensional predictors. We analyzed microbial abundance at the genus level to overcome the sparsity when examining individual species. To lessen the impact of extreme sequence reads on regression models, we used winsorization [31]; for each genus, sequence reads for the samples with five largest numbers of sequence reads were set to the sixth largest number of sequence reads. We converted abundance read counts to relative abundances for each genus and centered and scaled predictor and response variables to remove the need for an intercept term in the regression models. The same covariates were included as in our diversity analyses. We estimated coefficients with the Lasso estimator and tested hypotheses regarding predictor-response associations with a post-selection inference methodology [32, 33]. This method determined whether each microbial taxon (the predictor) was differentially abundant in relation to a respiratory outcome (the response variable) while controlling for all other microbial taxa using Lasso estimation to shrink coefficients of unimportant predictors toward zero. This procedure produces a test of significance of each taxon; but critically, each taxon is tested after removing the effects of all other taxa. Typical one-taxon-at-a-time testing does not involve either shrinkage of unimportant coefficients or adjustment for other taxa. Accordingly, we used a cutoff of *p*-value < 0.05 for statistical significance.

We used R version 3.4.0 for computation with optimization of the Lasso estimator performed by the software *mosek* wrapped through the R package *Rmosek*, which implemented fivefold cross-validation to choose the Lasso regularizer.

## Results

Of the 779 participants, 60% were male. Participants were 62 years old on average and from NC (32%) or IA (68%) (Table 1). As expected, individuals with asthma exhibited statistically significantly lower lung function and higher FeNO than noncases (Table S3).

**Table 1:** Characteristics of study participants (*N* = 779)

Characteristic	*N* (%) or mean ± SD
Age, years	62 ± 11
Sex	
Male	468 (60)
Female	311 (40)
Height, cm	171 ± 10
Weight, kg	90 ± 21
Smoking status	
Never	506 (65)
Former	227 (29)
Current	46 (6)
Pack-years in ever smokers	21 ± 23
Asthma status	
Yes	295 (38)
No	484 (62)
State of residence	
North Carolina	246 (32)
Iowa	533 (68)
Ancestry	
European	746 (96)
Not	33 (4)
Season of dust collection	
Winter	178 (23)
Not	601 (77)
Pulmonary function parameters	
FEV_1_, ml	2649 ± 853
FVC, ml	3620 ± 1036
FEV_1_/FVC	0.73 ± 0.10
FeNO^a^, ppb	2.8 ± 0.7

^a^Fractional exhaled nitric oxide, available in 767 participants. Values were natural log (ln) transformed for normality

**Fig. 1:**
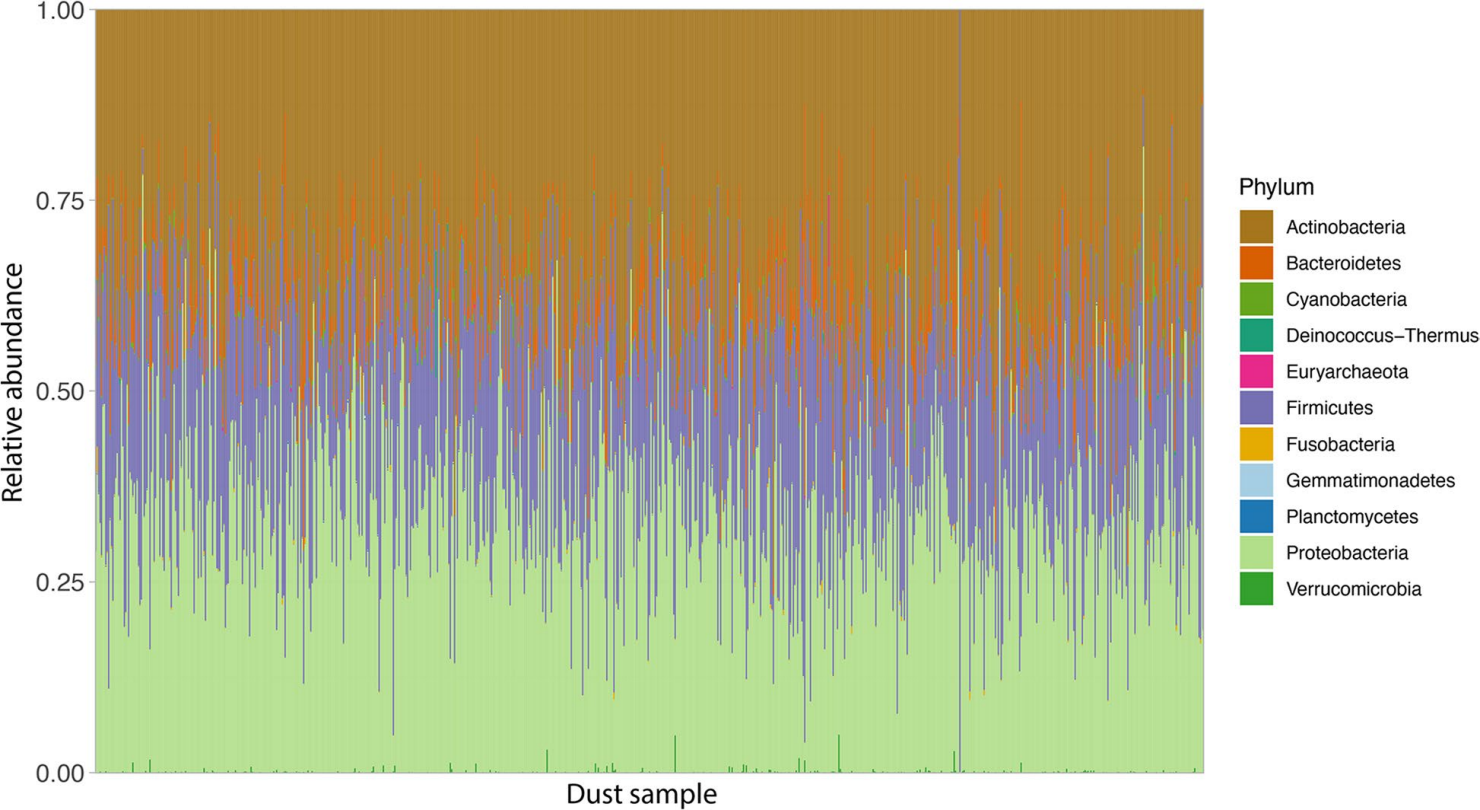
Phylum level summary of microbial taxa across all dust samples (*n* = 779). This figure shows the phylum level summary of relative abundance in each sample. The *x*-axis indicates house dust samples examined, and the *y*-axis represents relative abundance at the phylum level

After quality-control filtering, our house dust metagenome data included 173,766,690 sequence reads, with 223,064 sequence reads per sample on average. The taxa identified were 1264 species assigned to 389 genera. Most species (1260; 99.7%) were from 13 distinct phyla within Bacteria; only a few were from Archaea (Table S4). Of the 13 bacterial phyla, four predominated: Proteobacteria (39%), Actinobacteria (33%), Firmicutes (16%), and Bacteroidetes (11%). At the genus level, the genera *Staphylococcus* (phylum Firmicutes), *Pseudomonas* (phylum Proteobacteria), *Brevibacterium* (phylum Actinobacteria) were more abundant than other genera, and the three account for about 30% of the dust microbial communities. The three most abundant species were *Brevibacterium aurantiacum* (phylum Actinobacteria), *Cutibacterium acnes* (phylum Actinobacteria), and *Staphylococcus aureus* (phylum Firmicutes); each made up about 5–7% of the microbial communities. Microbial community compositions at the phylum level varied by sample (Fig. 1).

For overall diversity measures, the average richness was 241 (*SD* 40), and Shannon H index was 4.4 (*SD* 0.43) (Figure S1). Overall microbial diversity in house dust was not significantly related to respiratory outcomes. For both richness and Shannon index, higher diversity was related to higher pulmonary function parameters (FEV_1_ and FEV_1_/FVC), but these associations were not statistically significant (Table S5). Similar patterns were seen for FeNO.

**Fig. 2:**
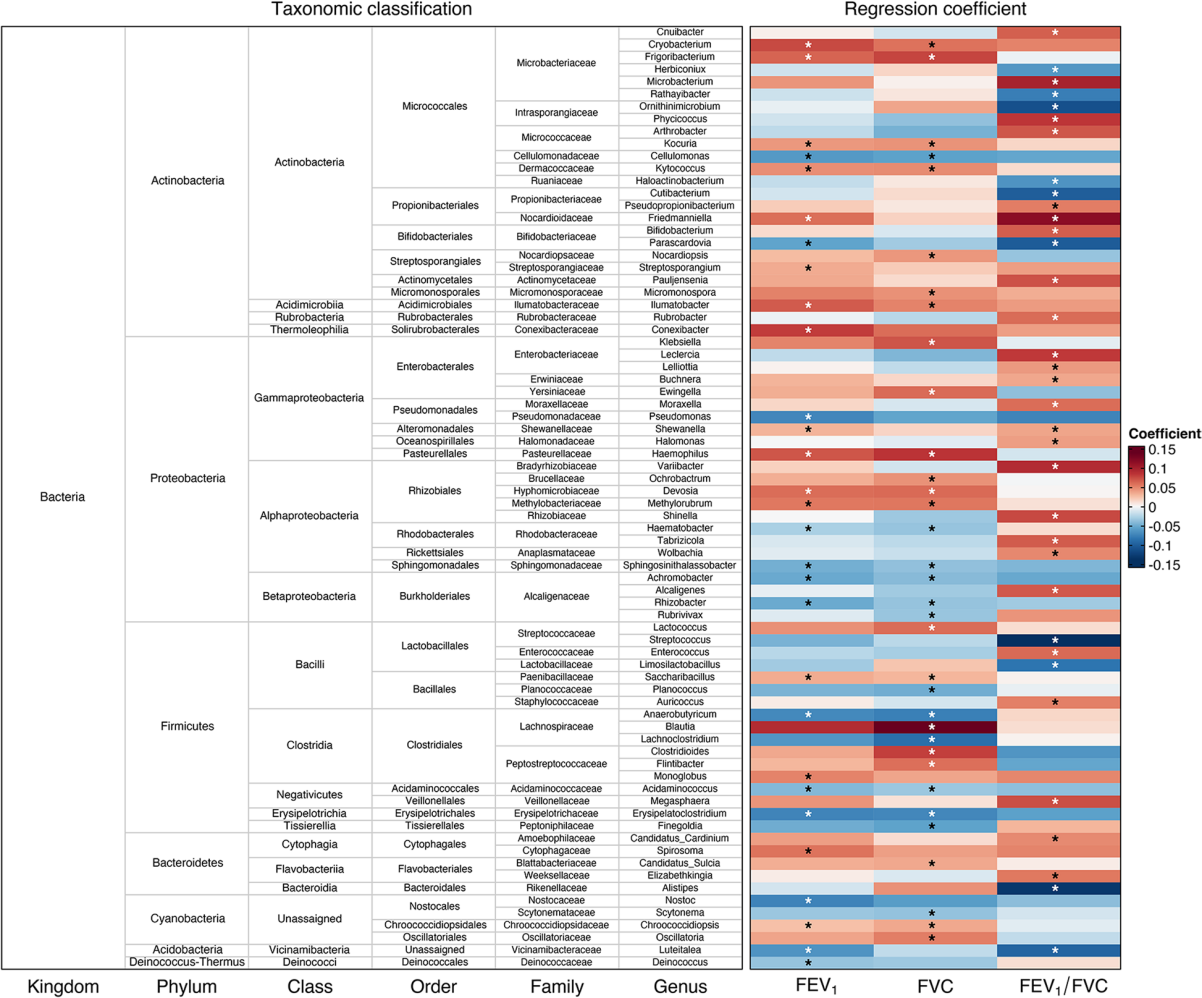
Heatmap of genera differentially abundant in relation to one or more pulmonary function parameters. The three rightmost columns visualize regression coefficients with statistical significance separately by pulmonary function parameter (FEV_1_, FVC, or FEV_1_/FVC). The six leftmost columns provide taxonomic classification (from kingdom to genus) for each taxon. Coding for *p*-value threshold is * for *p*-value < 0.05 after controlling for all other microbial taxa examined

**Fig. 3:**
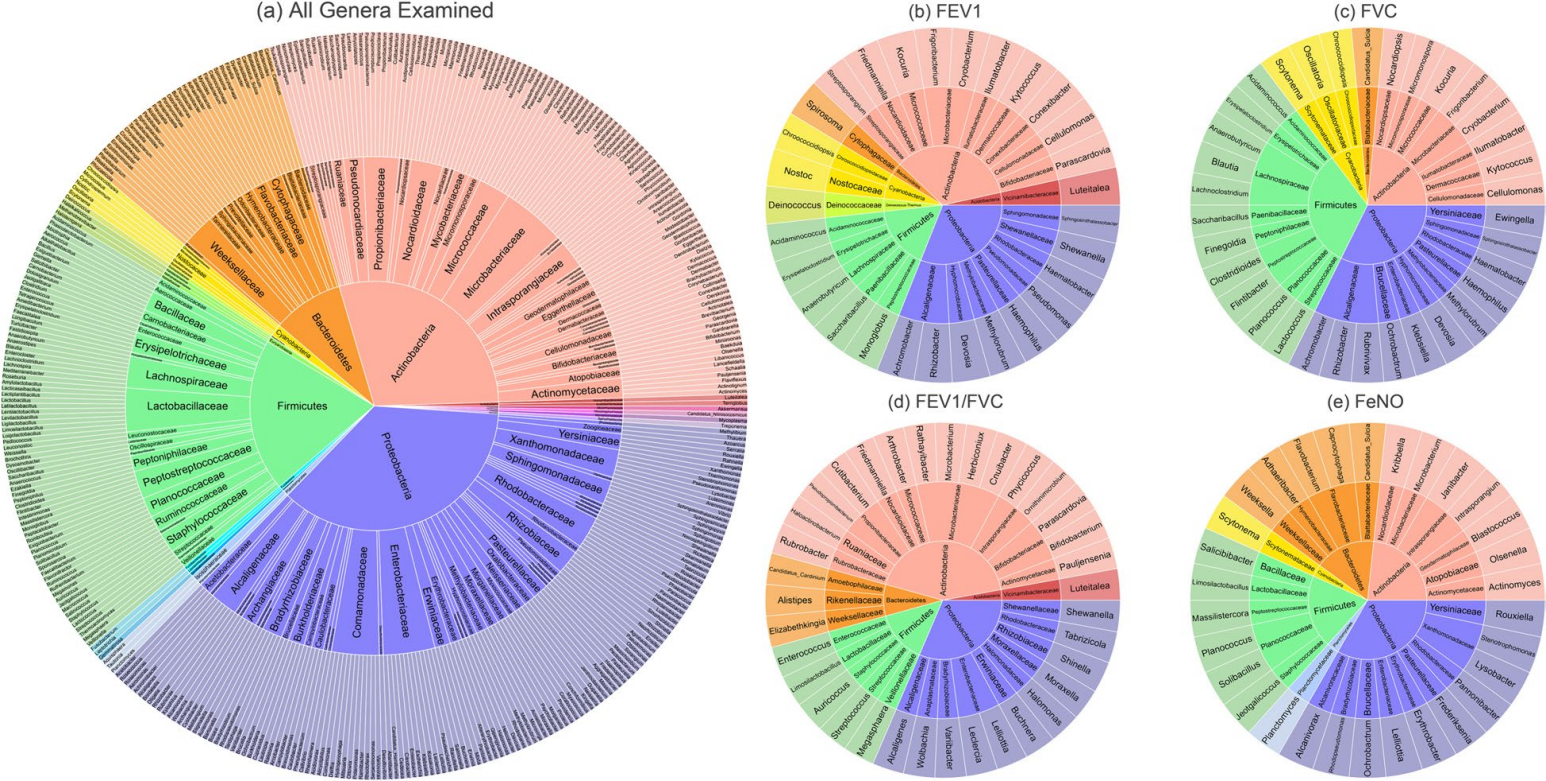
Sunburst plots visualizing membership of microbial taxa significantly associated with a health outcome at different taxonomic levels. Each sunburst plot displays a hierarchical summary of phylum-, family-, and genus-level taxonomic name (from center to edge respectively) for **a** all genera examined and genera differentially abundant (*p*-value < 0.05 controlling for all other microbial taxa examined) in relation to each outcome: **b** FEV_1_, **c** FVC, **d** FEV_1_/FVC, and **e** FeNO

Many individual microbial genera were differentially abundant in relation to pulmonary function. Of the 389 genera examined, 76 were related to one or more pulmonary function parameters (*p*-value < 0.05) in analyses that adjusted for all other microbial taxa (Fig. 2, Table 2). Most were from the bacterial phyla Actinobacteria (*N* = 25), Proteobacteria (*N* = 23), or Firmicutes (*N* = 17). Slightly, more genera showed positive than negative associations: 55% for FEV_1_, 62% for FVC, and 71% for FEV_1_/FVC. Of the 76 genera, 22 were associated with two parameters, including *Ilumatobacter* (phylum Actinobacteria), *Chroococcidiopsis* (phylum Cyanobacteria), and *Anaerobutyricum* (phylum Firmicutes). The remaining 54 genera were uniquely associated with a specific parameter, including *Streptococcus* (phylum Firmicutes) and *Moraxella* (phylum Proteobacteria) (Table S6). Significant genera were largely from more abundant phyla including Proteobacteria and Actinobacteria (Fig. 3). Notably, of two genera from phylum Acidobacteria examined in this work, one *Luteitalea* showed significant associations with FEV_1_ and FEV_1_/FVC.

**Table 2:** Genera differentially abundant (*p*-value < 0.05 controlling for all other microbial taxa examined) in relation to pulmonary function parameters

**Phylum**	**Family**	**Genus**	**Coefficient**	***p*-value**
**Trait: FEV**_**1**_				
Acidobacteria	Vicinamibacteraceae	*Luteitalea*	−0.066	0.012
Actinobacteria	Bifidobacteriaceae	*Parascardovia*	−0.055	0.012
Actinobacteria	Cellulomonadaceae	*Cellulomonas*	−0.062	0.004
Actinobacteria	Conexibacteraceae	*Conexibacter*	0.081	0.020
Actinobacteria	Dermacoccaceae	*Kytococcus*	0.046	0.038
Actinobacteria	Ilumatobacteraceae	*Ilumatobacter*	0.068	0.001
Actinobacteria	Microbacteriaceae	*Cryobacterium*	0.076	0.013
Actinobacteria	Microbacteriaceae	*Frigoribacterium*	0.064	0.005
Actinobacteria	Micrococcaceae	*Kocuria*	0.043	0.040
Actinobacteria	Nocardioidaceae	*Friedmanniella*	0.059	0.023
Actinobacteria	Streptosporangiaceae	*Streptosporangium*	0.035	0.031
Bacteroidetes	Cytophagaceae	*Spirosoma*	0.058	0.028
Cyanobacteria	Chroococcidiopsidaceae	*Chroococcidiopsis*	0.026	0.046
Cyanobacteria	Nostocaceae	*Nostoc*	−0.076	0.036
Deinococcus-Thermus	Deinococcaceae	*Deinococcus*	−0.037	0.049
Firmicutes	Acidaminococcaceae	*Acidaminococcus*	−0.043	0.022
Firmicutes	Erysipelotrichaceae	*Erysipelatoclostridium*	−0.075	0.029
Firmicutes	Lachnospiraceae	*Anaerobutyricum*	−0.072	0.045
Firmicutes	Paenibacillaceae	*Saccharibacillus*	0.034	0.049
Firmicutes	Peptostreptococcaceae	*Monoglobus*	0.051	0.022
Proteobacteria	Alcaligenaceae	*Achromobacter*	−0.052	0.005
Proteobacteria	Alcaligenaceae	*Rhizobacter*	−0.052	0.004
Proteobacteria	Hyphomicrobiaceae	*Devosia*	0.060	0.031
Proteobacteria	Methylobacteriaceae	*Methylorubrum*	0.055	0.004
Proteobacteria	Pasteurellaceae	*Haemophilus*	0.071	0.020
Proteobacteria	Pseudomonadaceae	*Pseudomonas*	−0.074	0.047
Proteobacteria	Rhodobacteraceae	*Haematobacter*	−0.029	0.030
Proteobacteria	Shewanellaceae	*Shewanella*	0.031	0.008
Proteobacteria	Sphingomonadaceae	*Sphingosinithalassobacter*	−0.049	0.010
**Trait: FVC**				
Actinobacteria	Cellulomonadaceae	*Cellulomonas*	−0.057	0.003
Actinobacteria	Dermacoccaceae	*Kytococcus*	0.047	0.025
Actinobacteria	Ilumatobacteraceae	*Ilumatobacter*	0.051	0.015
Actinobacteria	Microbacteriaceae	*Cryobacterium*	0.058	0.040
Actinobacteria	Microbacteriaceae	*Frigoribacterium*	0.078	0.001
Actinobacteria	Micrococcaceae	*Kocuria*	0.044	0.026
Actinobacteria	Micromonosporaceae	*Micromonospora*	0.045	0.044
Actinobacteria	Nocardiopsaceae	*Nocardiopsis*	0.043	0.026
Bacteroidetes	Blattabacteriaceae	*Candidatus_Sulcia*^*a*^	0.036	0.049
Cyanobacteria	Chroococcidiopsidaceae	*Chroococcidiopsis*	0.036	0.007
Cyanobacteria	Oscillatoriaceae	*Oscillatoria*	0.053	0.033
Cyanobacteria	Scytonemataceae	*Scytonema*^*a*^	−0.037	0.044
Firmicutes	Acidaminococcaceae	*Acidaminococcus*	−0.034	0.024
Firmicutes	Erysipelotrichaceae	*Erysipelatoclostridium*	−0.073	0.014
Firmicutes	Lachnospiraceae	*Anaerobutyricum*	−0.078	0.006
Firmicutes	Lachnospiraceae	*Blautia*	0.139	0.038
Firmicutes	Lachnospiraceae	*Lachnoclostridium*	−0.089	0.032
Firmicutes	Paenibacillaceae	*Saccharibacillus*	0.030	0.047
Firmicutes	Peptoniphilaceae	*Finegoldia*	−0.056	0.044
Firmicutes	Peptostreptococcaceae	*Clostridioides*	0.080	0.011
Firmicutes	Peptostreptococcaceae	*Flintibacter*	0.059	0.020
Firmicutes	Planococcaceae	*Planococcus*^*a*^	−0.050	0.014
Firmicutes	Streptococcaceae	*Lactococcus*	0.060	0.004
Proteobacteria	Alcaligenaceae	*Achromobacter*	−0.042	0.028
Proteobacteria	Alcaligenaceae	*Rhizobacter*	−0.036	0.025
Proteobacteria	Alcaligenaceae	*Rubrivivax*	−0.035	0.050
Proteobacteria	Brucellaceae	*Ochrobactrum*^*a*^	0.045	0.038
Proteobacteria	Enterobacteriaceae	*Klebsiella*	0.071	0.026
Proteobacteria	Hyphomicrobiaceae	*Devosia*	0.061	0.023
Proteobacteria	Methylobacteriaceae	*Methylorubrum*	0.056	0.006
Proteobacteria	Pasteurellaceae	*Haemophilus*	0.085	0.004
Proteobacteria	Rhodobacteraceae	*Haematobacter*	−0.036	0.007
Proteobacteria	Sphingomonadaceae	*Sphingosinithalassobacter*	−0.038	0.026
Proteobacteria	Yersiniaceae	*Ewingella*	0.061	0.046
**Trait: FEV**_**1**_**/FVC**				
Acidobacteria	Vicinamibacteraceae	*Luteitalea*	−0.096	0.016
Actinobacteria	Actinomycetaceae	*Pauljensenia*	0.072	0.026
Actinobacteria	Bifidobacteriaceae	*Bifidobacterium*	0.067	0.005
Actinobacteria	Bifidobacteriaceae	*Parascardovia*	−0.103	0.003
Actinobacteria	Intrasporangiaceae	*Ornithinimicrobium*	−0.112	0.004
Actinobacteria	Intrasporangiaceae	*Phycicoccus*	0.084	0.047
Actinobacteria	Microbacteriaceae	*Cnuibacter*	0.066	0.042
Actinobacteria	Microbacteriaceae	*Herbiconiux*	−0.064	0.016
Actinobacteria	Microbacteriaceae	*Microbacterium*^*a*^	0.099	0.020
Actinobacteria	Microbacteriaceae	*Rathayibacter*	−0.079	2.1 × 10^−4^
Actinobacteria	Micrococcaceae	*Arthrobacter*	0.070	0.043
Actinobacteria	Nocardioidaceae	*Friedmanniella*	0.117	0.001
Actinobacteria	Propionibacteriaceae	*Cutibacterium*	−0.101	0.011
Actinobacteria	Propionibacteriaceae	*Pseudopropionibacterium*	0.053	0.049
Actinobacteria	Ruaniaceae	*Haloactinobacterium*	−0.066	0.022
Actinobacteria	Rubrobacteraceae	*Rubrobacter*	0.061	0.012
Bacteroidetes	Amoebophilaceae	*Candidatus_Cardinium*	0.048	0.049
Bacteroidetes	Rikenellaceae	*Alistipes*	−0.132	0.025
Bacteroidetes	Weeksellaceae	*Elizabethkingia*	0.055	0.045
Firmicutes	Enterococcaceae	*Enterococcus*	0.061	0.008
Firmicutes	Lactobacillaceae	*Limosilactobacillus*^*a*^	−0.085	0.037
Firmicutes	Staphylococcaceae	*Auricoccus*	0.052	0.022
Firmicutes	Streptococcaceae	*Streptococcus*	−0.144	0.011
Firmicutes	Veillonellaceae	*Megasphaera*	0.073	0.049
Proteobacteria	Alcaligenaceae	*Alcaligenes*	0.069	0.012
Proteobacteria	Anaplasmataceae	*Wolbachia*	0.049	0.027
Proteobacteria	Bradyrhizobiaceae	*Variibacter*	0.091	0.002
Proteobacteria	Enterobacteriaceae	*Leclercia*	0.083	0.029
Proteobacteria	Enterobacteriaceae	*Lelliottia*^*a*^	0.042	2.7 × 10^−4^
Proteobacteria	Erwiniaceae	*Buchnera*	0.036	0.026
Proteobacteria	Halomonadaceae	*Halomonas*	0.040	0.012
Proteobacteria	Moraxellaceae	*Moraxella*	0.060	0.009
Proteobacteria	Rhizobiaceae	*Shinella*	0.076	0.023
Proteobacteria	Rhodobacteraceae	*Tabrizicola*	0.068	0.014
Proteobacteria	Shewanellaceae	*Shewanella*	0.037	0.006

Association results were obtained from linear regression with each microbial taxon as the predictor and a pulmonary function parameter as the outcome while controlling for all other microbial taxa. Therefore, a cutoff of *p*-value < 0.05 was used for statistical significance. Covariates were age, age squared, sex, height, height squared (weight for FVC only), cigarette smoking (former or current, both relative to never), pack-years of cigarette smoking, asthma status (yes/no), state of residence (NC/IA), and ancestry (European/not). For each taxon, a positive regression coefficient means that a change in the relative abundance results in a change in the measure trait in the same direction. Negative coefficients imply an inverse association between the relative abundance and the measured trait

^a^Also associated with FeNO. See Table 3. All genera from kingdom Bacteria

For FeNO, we found 30 differentially abundant genera (*p*-value < 0.05) after controlling for all other microbial taxa, including *Stenotrophomonas* (phylum Proteobacteria) (Table 3). Notably, seven of these, including the bacterial genus *Limosilactobacillus* (phylum Firmicutes), were also related to a pulmonary function parameter (Table S6).

**Table 3:** Genera differentially abundant (*p*-value < 0.05 controlling for all other microbial taxa examined) in relation to FeNO

Phylum	Family	Genus	Coefficient	*p*-value
Actinobacteria	Actinomycetaceae	*Actinomyces*	−0.125	0.010
Actinobacteria	Atopobiaceae	*Olsenella*	0.106	0.019
Actinobacteria	Geodermatophilaceae	*Blastococcus*	−0.066	0.039
Actinobacteria	Intrasporangiaceae	*Intrasporangium*	−0.145	3.4 × 10^−4^
Actinobacteria	Intrasporangiaceae	*Janibacter*	−0.069	0.037
Actinobacteria	Microbacteriaceae	*Microbacterium*^*a*^	−0.106	0.034
Actinobacteria	Nocardioidaceae	*Kribbella*	−0.081	0.012
Bacteroidetes	Blattabacteriaceae	*Candidatus_Sulcia*^*a*^	−0.073	0.033
Bacteroidetes	Flavobacteriaceae	*Capnocytophaga*	0.132	0.011
Bacteroidetes	Flavobacteriaceae	*Flavobacterium*	0.089	0.038
Bacteroidetes	Hymenobacteraceae	*Adhaeribacter*	−0.077	0.036
Bacteroidetes	Weeksellaceae	*Weeksella*	0.039	0.024
Cyanobacteria	Scytonemataceae	*Scytonema*^*a*^	0.074	0.046
Firmicutes	Bacillaceae	*Salicibibacter*	0.098	0.030
Firmicutes	Lactobacillaceae	*Limosilactobacillus*	−0.087	0.027
Firmicutes	Peptostreptococcaceae	*Massilistercora*	−0.076	0.017
Firmicutes	Planococcaceae	*Planococcus*^*a*^	0.070	0.035
Firmicutes	Planococcaceae	*Solibacillus*	−0.081	4.9 × 10^−4^
Firmicutes	Staphylococcaceae	*Jeotgalicoccus*	0.124	0.027
Planctomycetes	Planctomycetaceae	*Planctomyces*	0.079	0.011
Proteobacteria	Alcanivoracaceae	*Alcanivorax*	0.096	3.2 × 10^−5^
Proteobacteria	Bradyrhizobiaceae	*Rhodopseudomonas*	0.071	0.035
Proteobacteria	Brucellaceae	*Ochrobactrum*^*a*^	0.086	0.007
Proteobacteria	Enterobacteriaceae	*Lelliottia*^*a*^	−0.046	8.0 × 10^−4^
Proteobacteria	Erythrobacteraceae	*Erythrobacter*	−0.041	0.004
Proteobacteria	Pasteurellaceae	*Frederiksenia*	−0.151	6.4 × 10^−5^
Proteobacteria	Rhodobacteraceae	*Pannonibacter*	0.075	0.023
Proteobacteria	Xanthomonadaceae	*Lysobacter*	0.096	0.011
Proteobacteria	Xanthomonadaceae	*Stenotrophomonas*	0.115	2.9 × 10^−4^
Proteobacteria	Yersiniaceae	*Rouxiella*	0.190	0.023

Association results were obtained from linear regression with each microbial taxon as the predictor and FeNO as the outcome while controlling for all other microbial taxa. Therefore, a cutoff of *p*-value < 0.05 was used for statistical significance. Covariates were age, sex, height, cigarette smoking (former or current, both relative to never), pack-years of cigarette smoking, asthma status (yes/no), state of residence (NC/IA), and ancestry (European/not). For each taxon, a positive regression coefficient means that a change in the relative abundance results in a change in the measure trait in the same direction. Negative coefficients imply an inverse association between the relative abundance and the measured trait

^a^Also associated with at least one pulmonary function parameter (FEV_1_, FVC, or FEV_1_/FVC) (see Table 2). All genera from kingdom Bacteria

When we interrogated our data at the species level, we found 189 (of 1264) species (106 genera) significantly related to lung function and 80 species (51 genera) significantly related to FeNO (*p*-value < 0.05 after accounting for all species examined together, Table S7). Of genera related to lung function and/or FeNO in the species level results, 63 containing 145 species were not significant in the genus level results. Of the 76 genera related to lung function in our genus level association results, 47 (62%) genera contained one or more significant species in the species level results (Table S8). Of the 30 genera associated with FeNO in the genus level results, 19 (63%) genera contained species exhibiting significant associations in the species level results (Table S9).

When we examined our 16S data [19], of the 76 genera related to lung function in our metagenome data, 31 were present. Of these, six, including *Moraxella* (phylum Proteobacteria), were significant (Table S10). Of the 30 genera associated with FeNO in our metagenome data, 11 were present, and 1 genera *Stenotrophomonas* (phylum Proteobacteria) showed significance (Table S11).

## Discussion

To our knowledge, this is the first large study to assess metagenome profiles in house dust using whole metagenome shotgun sequencing to examine associations of house dust microbiota with respiratory outcomes in adults. By comprehensively profiling microorganisms in house dust, we identified many individual microbial genera differentially abundant in relation to pulmonary function and/or airway inflammation. Among the genera that we identified as related to pulmonary function, several have been linked to pathogenesis of lung diseases previously, but others have not. Our results suggest that chronic exposure to specific microorganisms indoors may play a role in occupants’ respiratory outcomes.

House dust contains diverse microbial profiles. In our study, bacteria from four phyla (Proteobacteria, Actinobacteria, Firmicutes, and Bacteroidetes) predominated; each phylum included > 10% of the total species. This finding is similar to that generated using 16S rRNA amplicon sequencing [19]. Our whole metagenome sequencing provides more accurate identification of microbial species and adds nonbacterial microorganisms not targeted in 16S technology [34].

There are few data on associations between microbial communities measured in house dust and pulmonary function and none using whole metagenome sequencing. While we were not able to identify an independent indoor dust microbiome dataset to replicate our findings, we found some of our results overlapping with findings from a recent oral microbiome study of pulmonary function in Norwegian adults [35]. That study used 16S rRNA amplicon sequencing data and examined categorized levels of pulmonary function (low vs normal) and airway inflammation (eosinophilic inflammation vs normal). From a look-up analysis of our differentially abundant genera in their results, we were able to validate some of our findings [35]. Of genera identified for FEV_1_ and FVC in our dust data, the genus *Achromobacter* (phylum Proteobacteria) showed same directional (inverse) associations with the two lung function parameters (*FDR* < 0.05) in their oral microbiome data. Among genera we identified for FeNO, the genus *Janibacter* (phylum Actinobacteria) was negatively associated with FeNO in the oral microbiome data. The overlap between our findings and theirs provides partial validation for our findings.

Notably, several genera we identified as differentially abundant in relation to pulmonary function or airway inflammation have been linked to pathogenesis of lung diseases. The genus *Streptococcus* (phylum Firmicutes; family Streptococcaceae) was inversely associated with FEV_1_/FVC in our data; it contains several species, including *Streptococcus pneumoniae* and *Streptococcus pyogenes*, well known to cause pneumonia [36]. We also found the genus *Moraxella* (phylum Proteobacteria; family Moraxellaceae) related to FEV_1_/FVC. *Moraxella* is a genus of gram-negative bacteria and includes *Moraxella catarrhalis*, frequently observed in sputum of COPD patients and related to asthma exacerbations [37, 38]. *M. catarrhalis*, a known pathogen, is not generally considered an environmental microbe because it requires a human host to survive; however, it can live in dried sputum in the environment for up to 3 weeks [39]. Our identification of an association of this organism in dust with a pulmonary outcome hints at potential interactions between the human and indoor dust microbiomes. An additional genus positively associated with FEV_1_/FVC was *Bifidobacterium*. A recent review recognized potential contributions of probiotics, including the genus *Bifidobacterium*, in management of respiratory diseases [40]. The genus *Limosilactobacillus* associated with both FEV_1_/FVC and FeNO in our data includes *Limosilactobacillus reuteri*, which also has probiotic properties [41]. We observed the genus *Stenotrophomonas* (phylum Proteobacteria; family Xanthomonadaceae) significantly related to FeNO. The genus includes *Stenotrophomonas maltophilia*, a common multidrug-resistant organism related to severe lung infections in individuals with cystic fibrosis [42, 43].

Findings from earlier studies of pulmonary function and microbial components using older technologies are limited in the number of microbial agents investigated [11–14]. Most studies analyzed bacterial or fungal agents with qPCR, but results are not comparable across studies because the microbial components examined differed [12–14]. We generated a comprehensive set of metagenomic profiles in house dust using whole metagenome sequencing and identified specific microbial signatures related to pulmonary function and airway inflammation.

Sparsity due to excessive zero counts in microbiome data poses challenges in statistical analysis. Applying an additional filtering step to remove rare taxa and analyzing differential abundance data at the genus level enabled us to reduce spurious associations potentially induced by sparsity. Analysis at the species level results in greater sparsity. For some genera, we were able to identify specific species contributing to the significant associations at the genus level. We also found genera significant in the genus level results, but the species level analysis gave no significant species within that genus. These genus level associations were not driven by a single species but by the combined effects of different species.

Whole metagenome sequencing can capture microbial community composition with higher accuracy than 16S technology. In the same population, we identified many more microbial taxa and significant associations using whole genome sequencing microbiome data [34] compared to 16S [19]. Similarly, in this investigation of continuous health outcomes, we found some genera significant in our metagenome data were also related to lung function and/or FeNO in our 16S data (*p*-value < 0.05 after accounting for all genus level taxa examined together). Given the different microbial taxa and compositions captured using the two sequencing methods and the unique nature of our statistical method of considering all identified taxa in the same model, validation of these microorganisms identified using metagenome sequencing in our 16S data is reassuring and useful for comparison with prior 16S literature.

In a usual differential abundance analysis, researchers examine each microbial taxon one at a time and thus execute statistical testing with correction for the number of individual taxa included in the analysis. In contrast, the statistical method we implemented examines each microbial taxon after accounting for all microbial taxa identified in the data. The dust microbiome is inherently a mixture, and individuals are exposed to all of the identified organisms simultaneously. Therefore, our method of accounting for all the other taxa in dust may be more appropriate and can provide additional insights into understanding the impact of this complex environmental exposure on human health.

The high-dimensional metagenome data analysis method we implemented addresses the limited availability of statistical analysis methods suitable for continuous outcomes and metagenome data also allowing for adjustment for covariates. While it is relatively new in the context of metagenome data analysis, it is built upon a strong statistical foundation [32, 33]. Using this high-dimensional method in the analysis of a continuous outcome is a strength of our study and should be useful to researchers studying impacts of the microbiome on other continuous outcomes and will advance metagenome analysis.

In our dust metagenome sequencing data, we observed 3% of sequence reads mapped to *Dermatophagoides farinae*, a known dust mite associated with asthma symptoms [44]. Examining relative abundance of this organism in relation to lung function and airway inflammation is of interest.

Recent studies suggest impacts of air pollutants on indoor microbiome in urban populations [45, 46]. Due to lack of air pollution data at the time of home visit, we are not able assess how air pollution plays a role in associations between indoor microbiome and respiratory health. We also note that the expected variability in air pollution in this predominantly rural population would limit the ability to detect impacts on the indoor microbiome. Future research is warranted to disentangle the complex relationships between air pollution, indoor microbiome, and respiratory health.

This study has limitations. We analyzed a single dust sample from each house to reflect the usual home condition and measured only bedroom dust. Exposure assessment would be improved by collecting samples at multiple time points. Improved exposure assessment could enhance our ability to robustly detect associations between dust microbial composition and the outcomes under study. Because most individuals spend a large portion of their day in the bedroom, it is a highly relevant single location to sample. That our study population is from a US farming cohort could potentially limit the generalizability of our findings; however, because farm exposures contribute to higher microbial diversity [19, 47] in house dust, our farming population might improve our ability to identify associations with health outcomes. Because of the unique characteristics of our study — house dust metagenome characterized using whole genome sequencing together with adult respiratory outcomes — we could not identify a replication population. Therefore, we looked up taxa we identified in literature and found associations of some with lung diseases. We were unable to examine absolute bacterial load using sequencing data. Although we increased the number of microorganisms examined by using whole metagenome sequencing, sequencing methods do not allow absolute quantification of microbial abundance. Nevertheless, our differential abundance method allowed identification of directions of associations. Though it would be useful, we lack matching human microbiome data. Besides, potentially impacting the human microbiome, however, the house dust metagenome is an environmental exposure that can directly impact health outcomes. Thus, the associations we observed are of interest. Finally, the cross-sectional observational study design limited our ability to draw causal inferences. Associations could reflect influences of microbial exposures on respiratory outcomes or alternatively reflect the influence of occupants’ respiratory health on the microbial composition of house dust.

Key strengths of our study include the large sample size as the first indoor metagenomic study of pulmonary function in adults. We generated comprehensive metagenome profiles using whole genome shotgun sequencing. Compared to the older 16S rRNA amplicon sequencing which obtains information on operational taxonomic units (OTUs) based on sequence similarity, shotgun metagenomic sequencing can lead to more accurate detection of microorganisms by direct sequencing fragments of the genome. We applied several quality-control steps and filtering criteria to improve the quality of metagenome abundance data before association analyses; in particular, we removed poor quality sequence reads and rare microbial taxa. Our dataset with 252,595 (25%) zero microbial counts across all samples was much less sparse than typically seen for metagenome data [48]. Excluding rare taxa, reducing zero counts, and removing extreme outliers by winsorization should reduce false-positive findings. By using whole genome shotgun sequencing, we also captured nonbacterial profiles: 23 species from four phyla within Eukaryota, 4 from three phyla within Archaea, and 3 from two viral phyla. We applied recently developed inference methods that allowed us to examine associations between a high-dimensional predictor (house dust metagenome) and respiratory outcomes while accounting for the many taxa examined together. Because humans are exposed to the entire community of microorganisms, it is crucial to use an appropriate high-dimensional modeling approach like ours that probes the complex relationships among microorganisms. In addition to pulmonary function, we analyzed FeNO, a marker of airway inflammation. With no prior whole metagenome sequencing-based studies of microbial exposure, our findings of individual genera associated with pulmonary function and FeNO could inform mechanisms between exposure to microbes indoors and respiratory inflammation.

Our study fills the knowledge gap in the current literature by validating previously known disease-related microorganisms in the study of continuous measures reflecting respiratory health as well as identifying novel associations. Microbes related to several lung diseases appear to correlate with lung function and/or airway inflammation regardless of disease status. Validation in additional population studies reinforced by mechanistic studies could provide the basis for actionable guidelines for farmers and public health professionals.

We found microbial signatures in house dust associated with continuous measures of pulmonary function and airway inflammation in adults. Although overall microbial diversity was not significantly related to pulmonary function, many specific genera were differentially abundant in relation to pulmonary function and/or airway inflammation. Further investigation of the genera identified could inform contributions of exposure to indoor microorganisms to respiratory health. This comprehensive investigation of microbial signatures in house dust and adult respiratory outcomes could help elucidate complex mechanisms of chronic exposure to house dust and respiratory health across the life course.

### Supplementary Information

**Additional file 1:** Online methods: 1. Identification of differentially abundant taxa. 2. Whole genome shotgun metagenomic sequencing and quality control steps.

**Additional file 2:** Supplemental figure: **Figure S1.** Distributions of alpha diversity measures: richness and exponentially transformed Shannon H index. Supplemental tables: **Table S1.** Reference genomes used to evaluate host contaminant related sequence reads. **Table S2.** Taxonomic classification of 168 taxa removed after evaluation of potential contaminant DNA sequence using the decontam R package. **Table S3.** Pulmonary function parameters and airway inflammation by asthma status. **Table S4.** Taxonomic classification of the 1264 species, from Bacteria and Archaea, included in our metagenome analysis of house dust. **Table S5.** Overall microbial diversity in relation to pulmonary function parameters and FeNO. **Table S6.** Taxa differentially abundant (P-value<0.05 after accounting for all other taxa examined together) in relation to one or more pulmonary function parameters and/or FeNO. **Table S7.** Taxa differentially abundant (P-value<0.05 after accounting for all other taxa examined together) in relation to one or more pulmonary function parameters and/or FeNO: Species level analysis results. **Table S8.** Species level association results for the 76 genera related to lung function parameters (p-value<0.05) in the genus level differential abundance analysis results. **Table S9.** Species level association results for the 30 genera related to FeNO (P<0.05) in the genus level differential abundance analysis results. **Table S10.** Associations of lung function parameters with 31 genera examined in our 16S data. **Table S11.** Associations of FeNO with 11 genera examined in our 16S data.

**Additional file 3:** Online Supplement: Complete association results.

## Data Availability

Metagenome data used for this analysis are available at the Sequence Read Archive (SRA) under project number PRJNA975673 (https://www.ncbi.nlm.nih.gov/sra/). Complete association results are in the Online supplement.
