# Supplementary material for: House dust metagenome and pulmonary function in a US farming population
Source: Microbiome. 2024 Jul 18;12:129. doi: 10.1186/s40168-024-01823-y (PMC11256371; doi:10.1186/s40168-024-01823-y)
Supplement: Supplementary file 2 — Additional file 1: Online methods: 1. Identification of differentially abundant taxa. 2. Whole genome shotgun metagenomic sequencing and quality control steps. [file 40168_2024_1823_MOESM1_ESM.pdf]

Online Methods

Identification of differentially abundant taxa

Recent developments in the statistical literature provide valid methods for inference in high-dimensional regression models. An overview of these methods can be found in Chernozhukov et al. (2015) and Ning and Liu (2017). When the number of predictors (p) is large compared to the sample size (n), as in our data, traditional methods based on least squares either cannot be implemented or yield very low statistical power. Algorithm 1 (below), reproduced from these references, is the main algorithm required for inference.

The model assumed in the analyses is a multiple linear regression model, i. e., $y_i = x_i^T \beta + \varepsilon_i$. where the number of predictors is potentially large. Here y_i represents a phenotype measurement (i.e., a pulmonary function trait or FeNO) where $i = 1, ..., n$. indexes participants. The vector $x_i = (x_{i1}, ..., x_{ip})^T$ contains all predictor variables including relative abundances of microbial taxa and any additional covariates.

Association between predictors and the phenotype is measured via the vector of regression coefficients $\beta = (\beta_1, ...\beta_p)^T$, where β_j measures the association between the jth predictor and the phenotype while controlling for all other predictors.

Inference on associations is performed via the test,

$$H_0 : \beta_j = 0 \,(\text{no association}), \quad H_a : \beta_j \neq 0. \tag{1}$$

This test is carried out for each predictor variable. Regression coefficients are estimated via the Lasso estimator, and the computation of p-values for the test in (1) is decribed in Algorithm 1.

Algorithm 1 Inference on regression parameters with many predictors

1: Compute the Lasso estimate $\hat{\beta}$ of β.

2: Compute regularized estimates $\hat{\mu}^j$ by regressing the jth predictor x_{ij} on the vector of remaining predictors, denoted $x_{i,-j}$, namely,

$$\arg\min_{\mu} \left\{ \frac{1}{n} \sum_{i=1}^{n} (x_{ij} - x_{i,-j}^T \mu)^2 + \lambda \|\mu\|_1 \right\}$$

3: Compute $\hat{\Sigma}_j = \frac{1}{n} \sum_{i=1}^{n} (x_{ij} - x_{i,-j}^T \hat{\mu}^j) x_{ij}$ and the refitted estimate of the target coefficient β_j as

$$\tilde{\beta}_j = \frac{\hat{\Sigma}_j^{-1}}{n} \sum_{i=1}^{n} (x_{ij} - x_{i,-j}^T \hat{\mu}^j)(y_i - x_{i,-j} \hat{\beta}_{-j})$$

4: Test hypothesis (1) by the result $\sqrt{n}(\tilde{\beta}_j - \beta_j) \sim \mathcal{N}(0, \sigma_j^2)$ where σ_j^2 can also be consistently estimated from the data.

An additional constraint is imposed in this optimization: the sum of the relative abundances is 1.

References

Chernozhukov, V., C. Hansen, and M. Spindler (2015). Valid post-selection and post-regularization inference: An elementary, general approach. *Annu. Rev. Econ. 7*(1), 649–688.

Ning, Y., and H. Liu. (2017). A general theory of hypothesis tests and confidence regions for sparse high dimensional models. *The Annals of Statistics 45*(1), 158–195.

Whole genome shotgun metagenomic sequencing and quality control steps

Center for Microbiome Innovation, University of California San Diego completed library preparation, multiplexing, and whole genome shotgun sequencing using standard protocols [1]. FastQC v0.11.5 [2] was used to assess the quality of reads by Phred quality score, GC content, the presence of adapters, overrepresented k-mers, duplicated reads rate, and PCR artifacts or contaminations. Low-quality and adaptor sequences were removed using Atropos [3]. Bases with a Phred score < 15 and throw-away reads < 100 bp (*-q 15 --minimum-length 100*) were trimmed, starting from the end of the read. The internal sequencing standard PhiX 174 and human sequences were identified using bowtie2 [4], SAMtools [5], and BEDtools [6] to remove aligned sequences and their mates. The above procedures were performed in the Qiita pipeline [7] by the IGM Genomics Center at the University of California San Diego.

All raw reads of both ends (3' or 5') passed the basic FastQC figures (per base sequence quality and per sequence quality scores), and no low-quality sequences remained. Bimodal shape was observed in per sequence GC content, indicating the wide distribution of genome GC content across multiple species in metagenomic samples.

As an additional quality control step, we performed in silico separation of bacterial reads from contaminant reads using KneadData v0.7.10 (https://huttenhower.sph.harvard.edu/kneaddata/) with default settings *(--bypass-trim –serial --bowtie2-options=" --very-sensitive")*. In addition to the human and PhiX genomes, we included farm animals (cows (ARS-UCD1.2), pigs (Sscrofa11.1), chickens (GRCg6a), turkeys (Turkey_5.1), horses (EquCab3.0), goats (ARS1) and sheep (Oar_rambouillet_v1.0)), pets (dogs (CanFam3.1) and cats (Felis_catus_9.0)), and dust mites (Dfa_Genome_UMICH_USM_1.1) as these are potential contamination sources. We downloaded the corresponding animal reference genomes from the NCBI database and built them for the bowtie2 index (Table S1).

We then classified the resulting paired-end reads using Kraken2 v2.1.1 [8] with pre-compiled data comprising RefSeq genomes for bacteria, archaea, eukaryotes, fungi, viruses, and plasmids and NCBI taxonomy information, with a confidence score threshold of 0.05 *(--confidence 0.05*), to enhance the accuracy of taxonomic assignments [9]. We then ran the Kraken2 output against Bracken v2.5.0 [10] with default parameters (*-r 100 -l S -t 10*) to quantify abundance at the species level. We built the Bracken database with the default 35-mers length. Tables S2 and S3 summarize the overall statistics of read sequences and the proportion of each host genome contaminant across samples.

Due to low biomass in the dust samples, we separately performed and processed two sequencing runs. Metagenomics datasets from low biomass samples are particularly vulnerable to microbial contamination from the sample collection instrument, sequencing kit, and laboratory reagents. We incorporated 'blank' controls by sequencing sterile water without adding dust sample DNA extractions [11]. We used both frequency (the default threshold of 0.1) and prevalence (a stricter threshold of 0.5) based methods in the decontam R package v1.10.0 [12] to identify contaminated DNA sequences not present in the sampled community for each run. We then removed contaminants identified in either run. Using this process, we filtered out 168 taxa (Table S4). After separately conducting preprocessing and filtering for each run, we generated pooled abundance data by summing the abundance data from both runs.

References

1. Sanders JG, Nurk S, Salido RA, Minich J, Xu ZZ, Zhu Q, Martino C, Fedarko M, Arthur TD, Chen F, Boland BS, Humphrey GC, Brennan C, Sanders K, Gaffney J, Jepsen K, Khosroheidari M, Green C, Liyanage M, Dang JW, Phelan VV, Quinn RA, Bankevich A, Chang JT, Rana TM, Conrad DJ,

Sandborn WJ, Smarr L, Dorrestein PC, Pevzner PA, Knight R. Optimizing sequencing protocols for leaderboard metagenomics by combining long and short reads. *Genome Biol* 2019: 20(1): 226.
2. Andrews S. FastQC: a quality control tool for high throughput sequence data. Available online at: http://www.bioinformatics.babraham.ac.uk/projects/fastqc. 2010.
3. Didion JP, Martin M, Collins FS. Atropos: specific, sensitive, and speedy trimming of sequencing reads. *Peerj* 2017: 5: e3720.
4. Langmead B, Salzberg SL. Fast gapped-read alignment with Bowtie 2. *Nature methods* 2012: 9(4): 357-359.
5. Li H, Handsaker B, Wysoker A, Fennell T, Ruan J, Homer N, Marth G, Abecasis G, Durbin R. The Sequence Alignment/Map format and SAMtools. *Bioinformatics* 2009: 25(16): 2078-2079.
6. Quinlan AR, Hall IM. BEDTools: a flexible suite of utilities for comparing genomic features. *Bioinformatics* 2010: 26(6): 841-842.
7. Gonzalez A, Navas-Molina JA, Kosciolek T, McDonald D, Vazquez-Baeza Y, Ackermann G, DeReus J, Janssen S, Swafford AD, Orchanian SB, Sanders JG, Shorenstein J, Holste H, Petrus S, Robbins-Pianka A, Brislawn CJ, Wang M, Rideout JR, Bolyen E, Dillon M, Caporaso JG, Dorrestein PC, Knight R. Qiita: rapid, web-enabled microbiome meta-analysis. *Nature methods* 2018: 15(10): 796-798.
8. Wood DE, Lu J, Langmead B. Improved metagenomic analysis with Kraken 2. *Genome Biol* 2019: 20(1): 257.
9. Ye SH, Siddle KJ, Park DJ, Sabeti PC. Benchmarking Metagenomics Tools for Taxonomic Classification. *Cell* 2019: 178(4): 779-794.
10. Lu J, Breitwieser FP, Thielen P, Salzberg SL. Bracken: estimating species abundance in metagenomics data. *Peerj Comput Sci* 2017.
11. Quince C, Walker AW, Simpson JT, Loman NJ, Segata N. Shotgun metagenomics, from sampling to analysis. *Nat Biotechnol* 2017: 35(9): 833-844.
12. Davis NM, Proctor DM, Holmes SP, Relman DA, Callahan BJ. Simple statistical identification and removal of contaminant sequences in marker-gene and metagenomics data. *Microbiome* 2018: 6(1): 226.
