# Supplementary material for: House dust metagenome and pulmonary function in a US farming population
Source: Microbiome. 2024 Jul 18;12:129. doi: 10.1186/s40168-024-01823-y (PMC11256371; doi:10.1186/s40168-024-01823-y)
Supplement: Supplementary file 3 — Additional file 2: Supplemental figure: Figure S1. Distributions of alpha diversity measures: richness and exponentially transformed Shannon H index. Supplemental tables: Table S1. Reference genomes used to evaluate host contaminant related sequence reads. Table S2. Taxonomic classification of 168 taxa removed after evaluation of potential contaminant DNA sequence using the decontam R package. Table S3. Pulmonary function parameters and airway inflammation by asthma status. Table S4. Taxonomic classification of the 1264 species, from Bacteria and Archaea, included in our metagenome analysis of house dust. Table S5. Overall microbial diversity in relation to pulmonary function parameters and FeNO. Table S6. Taxa differentially abundant (P-value<0.05 after accounting for all other taxa examined together) in relation to one or more pulmonary function parameters and/or FeNO. Table S7. Taxa differentially abundant (P-value<0.05 after accounting for all other taxa examined together) in relation to one or more pulmonary function parameters and/or FeNO: Species level analysis results. Table S8. Species level association results for the 76 genera related to lung function parameters (p-value<0.05) in the genus level differential abundance analysis results. Table S9. Species level association results for the 30 genera related to FeNO (P<0.05) in the genus level differential abundance analysis results. Table S10. Associations of lung function parameters with 31 genera examined in our 16S data. Table S11. Associations of FeNO with 11 genera examined in our 16S data. [file 40168_2024_1823_MOESM2_ESM.pdf]

Supplemental Figure

Figure S1. Distributions of alpha diversity measures: richness and exponentially transformed Shannon H index.

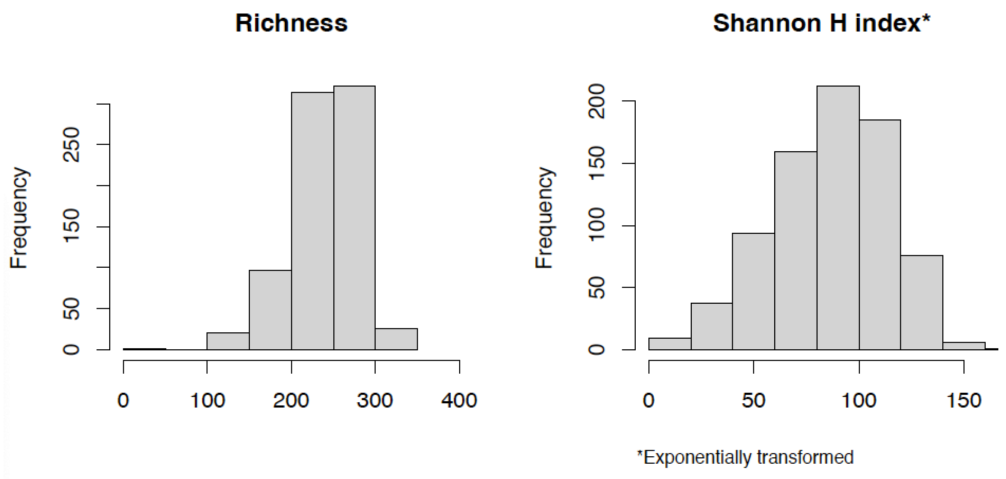

This figure visualizes the distribution of overall alpha diversity measures (n=779). Overall alpha diversity measures (richness and Shannon H index) were calculated using functions specnumber() and diversity(), respectively, in the vegan R package (v2.6.2). Shannon index was exponentially transformed for normality and used in the association analyses. To avoid bias due to different sequencing depths among samples, abundance data were rarefied to the minimum number of sequences (975) across samples before assessing microbial diversity.

Supplemental Tables

Table S1. Reference genomes used to evaluate host contaminant related sequence reads.

Host genome sources	Assembly	Annotation	Proportion of sequences identified as host genome contaminants across samples, mean (SD)	
			Sequencing Run 1	Sequencing Run 2
Cow	ARS-UCD1.2 (RefSeq: GCF_002263795.1)	NCBI Release 106	4.60 (2.51)	4.79 (4.80)
Pig	Sscrofa11.1 (RefSeq: GCF_000003025.6)	NCBI Release 106	4.30 (16.20)	4.27 (16.04)
Chicken	GRCg6a (RefSeq: GCF_000002315.6)	NCBI Release 104a	0.32 (1.68)	0.32 (1.45)
Turkey	Turkey_5.1 (RefSeq: GCF_000146605.3)	NCBI Release 103	0.06 (1.01)	0.06 (0.63)
Horse	EquCab3.0 (RefSeq: GCF_002863925.1)	NCBI Release 103	0.10 (0.28)	0.10 (0.33)
Goat	ARS1 (RefSeq: GCF_001704415.1)	NCBI Release 102	0.15 (2.21)	0.02 (0.22)
Sheep	Oar_rambouillet_v1.0 (RefSeq: GCF_002742125.1)	NCBI Release 104	0.01 (0.33)	0.01 (0.14)
Dog	CanFam3.1 (RefSeq: GCF_000002285.3)	NCBI Release 106	5.47 (12.08)	5.40 (12.02)
Cat	Felis_catus_9.0 (RefSeq: GCF_000181335.3)	NCBI Release 104	1.98 (6.64)	1.88 (5.57)
Dust mite	Dfa_Genome_UMICH_USM_1.1 (GenBank: GCA_002085665.2)	NA	2.98 (4.70)	3.00 (4.81)
Total			19.98 (20.93)	19.85 (20.83)

Table S2. Taxonomic classification of 168 taxa removed after evaluation of potential contaminant DNA sequence using the *decontam* R package.

Kingdom	Phylum	Class	Order	Family	Genus	Species
k__Bacteria	p__Proteobacteria	c__Alphaproteobacteria	o__Rickettsiales	f__Rickettsiaceae	g__Rickettsia	s__montanensis
k__Viruses	p__Artverviricota	c__Revtraviricetes	o__Ortervirales	f__Retroviridae	g__Gammaretrovirus	s__Porcine type-C oncovirus
k__Bacteria	p__Tenericutes	c__Mollicutes	o__Mycoplasmatales	f__Mycoplasmataceae	g__Mycoplasma	s__pullorum
k__Viruses	p__Uroviricota	c__Caudoviricetes	o__Caudovirales	f__Siphoviridae	g__	s__Lactococcus phage BM13
k__Bacteria	p__Firmicutes	c__Bacilli	o__Bacillales	f__Listeriaceae	g__Listeria	s__ivanovii
k__Bacteria	p__Proteobacteria	c__Gammaproteobacteria	o__Alteromonadales	f__Pseudoalteromonadaceae	g__Pseudoalteromonas	s__donghaensis
k__Bacteria	p__Proteobacteria	c__Gammaproteobacteria	o__Alteromonadales	f__Alteromonadaceae	g__Paraglaciecola	s__psychrophila
k__Bacteria	p__Thermotogae	c__Thermotogae	o__Petrotogales	f__Petrotogaceae	g__Defluviitoga	s__tunisiensis
k__Eukaryota	p__Fornicata	c__	o__Diplomonadida	f__Hexamitidae	g__Giardia	s__intestinalis
k__Viruses	p__Uroviricota	c__Caudoviricetes	o__Caudovirales	f__Siphoviridae	g__Saphexavirus	s__Enterococcus phage vB_EfaS_IME198
k__Viruses	p__Uroviricota	c__Caudoviricetes	o__Caudovirales	f__Siphoviridae	g__	s__Klebsiella phage KPN N137
k__Bacteria	p__Proteobacteria	c__Gammaproteobacteria	o__Vibrionales	f__Vibrionaceae	g__Vibrio	s__campbellii
k__Viruses	p__Uroviricota	c__Caudoviricetes	o__Caudovirales	f__Podoviridae	g__Kochitakasuvirus	s__Pseudomonas virus KPP25
k__Viruses	p__Uroviricota	c__Caudoviricetes	o__Caudovirales	f__Autographiviridae	g__Pifdecavirus	s__Pseudomonas virus PFP1
k__Archaea	p__Euryarchaeota	c__Thermococci	o__Thermococcales	f__Thermococcaceae	g__Palaeococcus	s__pacificus
k__Viruses	p__Uroviricota	c__Caudoviricetes	o__Caudovirales	f__Siphoviridae	g__Skunavirus	s__Lactococcus phage 56301
k__Bacteria	p__Firmicutes	c__Clostridia	o__Natranaerobiales	f__Natranaerobiaceae	g__Natranaerobius	s__thermophilus
k__Viruses	p__Uroviricota	c__Caudoviricetes	o__Caudovirales	f__Siphoviridae	g__Skunavirus	s__Lactococcus virus jj50
k__Bacteria	p__Cyanobacteria	c__	o__Synechococcales	f__Synechococcaceae	g__Synechococcus	s__sp. RS9909
k__Bacteria	p__Proteobacteria	c__Gammaproteobacteria	o__Legionellales	f__Coxiellaceae	g__Thiolapillus	s__brandeum
k__Bacteria	p__Proteobacteria	c__Alphaproteobacteria	o__Rhodospirillales	f__Acetobacteraceae	g__Swingsia	s__samuiensis
k__Bacteria	p__Chlamydiae	c__Chlamydiia	o__Parachlamydiales	f__Parachlamydiaceae	g__Candidatus Protochlamydia	s__naegleriophila
k__Viruses	p__Uroviricota	c__Caudoviricetes	o__Caudovirales	f__Myoviridae	g__Alcyoneusvirus	s__Klebsiella virus K64-1
k__Bacteria	p__Tenericutes	c__Mollicutes	o__Entomoplasmatales	f__Spiroplasmataceae	g__Spiroplasma	s__apis
k__Bacteria	p__Proteobacteria	c__Epsilonproteobacteria	o__Campylobacterales	f__Campylobacteraceae	g__Malaciobacter	s__canalis
k__Bacteria	p__Spirochaetes	c__Spirochaetia	o__Spirochaetales	f__Spirochaetaceae	g__Oceanispirochaeta	s__crateris
k__Viruses	p__Uroviricota	c__Caudoviricetes	o__Caudovirales	f__Siphoviridae	g__Nickievirus	s__Pseudomonas virus nickie
k__Viruses	p__Uroviricota	c__Caudoviricetes	o__Caudovirales	f__Siphoviridae	g__Galunavirus	s__Gordonia virus GAL1
k__Bacteria	p__Firmicutes	c__Bacilli	o__Bacillales	f__Paenibacillaceae	g__Paenibacillus	s__terrae
k__Bacteria	p__Proteobacteria	c__Gammaproteobacteria	o__Pasteurellales	f__Pasteurellaceae	g__Haemophilus	s__aegyptius
k__Viruses	p__Cressdnaviricota	c__Repensiviricetes	o__Geplafuvirales	f__Genomoviridae	g__	s__Bemisia-associated genomovirus AdDF
k__Viruses	p__Cossaviricota	c__Papovaviricetes	o__Zurhausenvirales	f__Papillomaviridae	g__Alphapapillomavirus	s__4
k__Viruses	p__Uroviricota	c__Caudoviricetes	o__Caudovirales	f__Siphoviridae	g__Sextaecvirus	s__Rhodococcus phage RRH1
k__Viruses	p__Nucleocytoviricota	c__Megaviricetes	o__Algavirales	f__Phycodnaviridae	g__	s__Orpheovirus IHUMI-LCC2
k__Bacteria	p__Firmicutes	c__Bacilli	o__Bacillales	f__Bacillaceae	g__Bacillus	s__sp. A260
k__Bacteria	p__Firmicutes	c__Bacilli	o__Bacillales	f__Bacillaceae	g__Bacillus	s__sp. MD-5
k__Bacteria	p__Actinobacteria	c__Actinobacteria	o__Candidatus Nanopelagicales	f__Candidatus Nanopelagicaceae	g__Candidatus Planktophila	s__versatilis
k__Viruses	p__Uroviricota	c__Caudoviricetes	o__Caudovirales	f__Siphoviridae	g__Trinavirus	s__Rhodococcus virus Trina
k__Bacteria	p__Firmicutes	c__Bacilli	o__Bacillales	f__Paenibacillaceae	g__Paenibacillus	s__sp. M-152
k__Viruses	p__Uroviricota	c__Caudoviricetes	o__Caudovirales	f__Myoviridae	g__Gaprivervirus	s__Escherichia virus VR25
k__Archaea	p__Euryarchaeota	c__Methanomicrobia	o__Methanomicrobiales	f__Methanomicrobiaceae	g__Methanoplanus	s__limicola
k__Bacteria	p__Proteobacteria	c__Gammaproteobacteria	o__Enterobacterales	f__Enterobacteriaceae	g__Leclercia	s__Enterobacteriaceae endosymbiont of Donacia piscatrix
k__Viruses	p__Uroviricota	c__Caudoviricetes	o__Caudovirales	f__Herelleviridae	g__Twortvirus	s__Staphylococcus virus Twort
k__Viruses	p__Uroviricota	c__Caudoviricetes	o__Caudovirales	f__Siphoviridae	g__Abidjanvirus	s__Pseudomonas virus Ab18

k__Viruses	p__Uroviricota	c__Caudoviricetes	o__Caudovirales	f__Demerecviridae	g__Myunavirus	s__Pectobacterium virus My1
k__Bacteria	p__Proteobacteria	c__Gammaproteobacteria	o__Enterobacterales	f__Morganellaceae	g__Providencia	s__sp. 2.29
k__Bacteria	p__Proteobacteria	c__Betaproteobacteria	o__Burkholderiales	f__Burkholderiaceae	g__Mycoavidus	s__cysteinexigens
k__Bacteria	p__Bacteroidetes	c__Flavobacteriia	o__Flavobacteriales	f__Blattabacteriaceae	g__Blattabacterium	s__sp. (Blaberus giganteus)
k__Viruses	p__Cossaviricota	c__Papovaviricetes	o__Zurhausenvirales	f__Papillomaviridae	g__Gammapapillomavirus	s__10
k__Bacteria	p__Proteobacteria	c__Gammaproteobacteria	o__Alteromonadales	f__Alteromonadaceae	g__Alteromonas	s__australica
k__Viruses	p__Uroviricota	c__Caudoviricetes	o__Caudovirales	f__Siphoviridae	g__Cheoctovirus	s__Mycobacterium phage Mantra
k__Bacteria	p__Proteobacteria	c__Epsilonproteobacteria	o__Nautiliales	f__Nautiliaceae	g__Nautilia	s__profundicola
k__Viruses	p__Uroviricota	c__Caudoviricetes	o__Caudovirales	f__Myoviridae	g__Obolenskvirus	s__Acinetobacter virus WCHABP12
k__Bacteria	p__Tenericutes	c__Mollicutes	o__Mycoplasmatales	f__Mycoplasmataceae	g__Mycoplasma	s__tullyi
k__Bacteria	p__Proteobacteria	c__Gammaproteobacteria	o__Thiotrichales	f__Francisellaceae	g__Francisella	s__sp. FSC1006
k__Viruses	p__Uroviricota	c__Caudoviricetes	o__Caudovirales	f__Podoviridae	g__Gamaleyavirus	s__Escherichia virus EC1UPM
k__Viruses	p__Uroviricota	c__Caudoviricetes	o__Caudovirales	f__Siphoviridae	g__Skunavirus	s__Lactococcus phage 37201
k__Viruses	p__Artverviricota	c__Revtraviricetes	o__Ortervirales	f__Caulimoviridae	g__Badnavirus	s__Piper yellow mottle virus
k__Bacteria	p__Thermotogae	c__Thermotogae	o__Kosmotogales	f__Kosmotogaceae	g__Kosmotoga	s__olearia
k__Viruses	p__Uroviricota	c__Caudoviricetes	o__Caudovirales	f__Siphoviridae	g__Nymphadoravirus	s__Gordonia virus Zirinka
k__Viruses	p__Uroviricota	c__Caudoviricetes	o__Caudovirales	f__Siphoviridae	g__Nymphadoravirus	s__Gordonia virus Kita
k__Viruses	p__Uroviricota	c__Caudoviricetes	o__Caudovirales	f__Myoviridae	g__Simpcentumvirus	s__Stenotrophomonas virus Smp131
k__Viruses	p__Uroviricota	c__Caudoviricetes	o__Caudovirales	f__Siphoviridae	g__Yangvirus	s__Arthobacter virus Yang
k__Viruses	p__Peploviricota	c__Herviviricetes	o__Herpesvirales	f__Herpesviridae	g__Varicellovirus	s__Canid alphaherpesvirus 1
k__Viruses	p__Uroviricota	c__Caudoviricetes	o__Caudovirales	f__Myoviridae	g__Tequatrovirus	s__Enterobacteria phage RB27
k__Viruses	p__Uroviricota	c__Caudoviricetes	o__Caudovirales	f__Siphoviridae	g__	s__Mycobacterium phage Sparky
k__Bacteria	p__Proteobacteria	c__Epsilonproteobacteria	o__Campylobacterales	f__Campylobacteraceae	g__Campylobacter	s__subantarcticus
k__Viruses	p__Uroviricota	c__Caudoviricetes	o__Caudovirales	f__Autographiviridae	g__Pifdecavirus	s__Pseudomonas virus Pf10
k__Viruses	p__Uroviricota	c__Caudoviricetes	o__Caudovirales	f__Siphoviridae	g__Samistivirus	s__Streptomyces virus Mildred21
k__Bacteria	p__Cyanobacteria	c__Gloeobacteria	o__Synechococcales	f__Synechococcaceae	g__Synechococcus	s__sp. CC9311
k__Bacteria	p__Proteobacteria	c__Gammaproteobacteria	o__Vibrionales	f__Vibrionaceae	g__Vibrio	s__neocaledonicus
k__Bacteria	p__Firmicutes	c__Bacilli	o__Bacillales	f__Bacillaceae	g__Bacillus	s__toyonensis
k__Viruses	p__Cossaviricota	c__Papovaviricetes	o__Zurhausenvirales	f__Papillomaviridae	g__Deltapapillomavirus	s__4
k__Viruses	p__Nucleocytoviricota	c__Pokkesviricetes	o__Chitovirales	f__Nimaviridae	g__Whispovirus	s__White spot syndrome virus
k__Viruses	p__Peploviricota	c__Herviviricetes	o__Herpesvirales	f__Herpesviridae	g__Percavirus	s__Equid gammaherpesvirus 5
k__Viruses	p__Uroviricota	c__Caudoviricetes	o__Caudovirales	f__Drexlerviridae	g__Webervirus	s__Enterobacter virus F20
k__Bacteria	p__Chlamydiae	c__Chlamydiia	o__Chlamydiales	f__Chlamydiaceae	g__Chlamydia	s__pneumoniae
k__Viruses	p__Uroviricota	c__Caudoviricetes	o__Caudovirales	f__Autographiviridae	g__Teseptimavirus	s__Escherichia virus 64795ec1
k__Viruses	p__Uroviricota	c__Caudoviricetes	o__Caudovirales	f__Podoviridae	g__	s__Clostridium phage CpV1
k__Bacteria	p__Aquificae	c__Aquificae	o__Aquificales	f__Aquificaceae	g__Hydrogenobacter	s__thermophilus
k__Viruses	p__Cossaviricota	c__Papovaviricetes	o__Zurhausenvirales	f__Papillomaviridae	g__Alphapapillomavirus	s__5
k__Viruses	p__Uroviricota	c__Caudoviricetes	o__Caudovirales	f__Autographiviridae	g__Vectrevirus	s__Citrobacter CrRp3
k__Viruses	p__Uroviricota	c__Caudoviricetes	o__Caudovirales	f__Autographiviridae	g__Vectrevirus	s__Escherichia virus VEc3
k__Bacteria	p__Nitrospirae	c__Nitrospira	o__Nitrospirales	f__Nitrospiraceae	g__Leptospirillum	s__sp. Group II 'CF-1'
k__Viruses	p__Uroviricota	c__Caudoviricetes	o__Caudovirales	f__Podoviridae	g__	s__Streptomyces phage Immanuel3
k__Viruses	p__Uroviricota	c__Caudoviricetes	o__Caudovirales	f__Ackermannviridae	g__Agtrevirus	s__Enterobacter phage EspM4VN
k__Viruses	p__Uroviricota	c__Caudoviricetes	o__Caudovirales	f__Siphoviridae	g__Skunavirus	s__Lactococcus virus sk1
k__Viruses	p__Uroviricota	c__Caudoviricetes	o__Caudovirales	f__Siphoviridae	g__Limdunavirus	s__Leuconostoc virus LN12
k__Bacteria	p__Tenericutes	c__Mollicutes	o__Mycoplasmatales	f__Mycoplasmataceae	g__Mycoplasma	s__maculosum
k__Bacteria	p__Proteobacteria	c__Epsilonproteobacteria	o__Campylobacterales	f__Campylobacteraceae	g__Campylobacter	s__sp. RM16704
k__Viruses	p__Uroviricota	c__Caudoviricetes	o__Caudovirales	f__Siphoviridae	g__Skunavirus	s__Lactococcus phage jm2
k__Viruses	p__Uroviricota	c__Caudoviricetes	o__Caudovirales	f__Myoviridae	g__Vequintavirus	s__Escherichia virus Murica
k__Viruses	p__Uroviricota	c__Caudoviricetes	o__Caudovirales	f__Chaseviridae	g__Carltongylesvirus	s__Escherichia phage Mangalitsa
k__Bacteria	p__Firmicutes	c__Clostridia	o__Clostridiales	f__Peptococcaceae	g__Desulfotomaculum	s__nigrificans

k__Bacteria	p__Proteobacteria	c__Gammaproteobacteria	o__Alteromonadales	f__Pseudoalteromonadaceae	g__Pseudoalteromonas	s__spongiae
k__Bacteria	p__Proteobacteria	c__Alphaproteobacteria	o__Rhizobiales	f__Brucellaceae	g__Brucella	s__ovis
k__Bacteria	p__Proteobacteria	c__Deltaproteobacteria	o__Desulfurellales	f__Desulfurellaceae	g__Desulfurella	s__acetivorans
k__Bacteria	p__Proteobacteria	c__Gammaproteobacteria	o__Enterobacterales	f__Morganellaceae	g__Xenorhabdus	s__poinarii
k__Bacteria	p__Proteobacteria	c__Gammaproteobacteria	o__Legionellales	f__Coxiellaceae	g__Candidatus Thioglobus	s__sp. NP1
k__Viruses	p__Uroviricota	c__Caudoviricetes	o__Caudovirales	f__Siphoviridae	g__	s__Streptococcus phage M102
k__Viruses	p__Uroviricota	c__Caudoviricetes	o__Caudovirales	f__Autographiviridae	g__Pijolavirus	s__Pseudomonas virus PspYZU08
k__Bacteria	p__Spirochaetes	c__Spirochaetia	o__Leptospirales	f__Leptospiraceae	g__Leptospira	s__tipperaryensis
k__Bacteria	p__Proteobacteria	c__Gammaproteobacteria	o__Enterobacterales	f__Enterobacteriaceae	g__Enterobacter	s__mori
k__Viruses	p__Uroviricota	c__Caudoviricetes	o__Caudovirales	f__Podoviridae	g__Bjornvirus	s__Pseudomonas virus Bjorn
k__Viruses	p__Uroviricota	c__Caudoviricetes	o__Caudovirales	f__Siphoviridae	g__Septimatrevirus	s__Pseudomonas virus Ab26
k__Bacteria	p__Proteobacteria	c__Gammaproteobacteria	o__Enterobacterales	f__Enterobacteriaceae	g__Citrobacter	s__sp. RHBSTW-00944
k__Viruses	p__Uroviricota	c__Caudoviricetes	o__Caudovirales	f__Myoviridae	g__Chakrabartyvirus	s__Pseudomonas virus pf16
k__Bacteria	p__Proteobacteria	c__Alphaproteobacteria	o__Rickettsiales	f__Candidatus Midichloriaceae	g__Candidatus Fokinia	s__solitaria
k__Viruses	p__Uroviricota	c__Caudoviricetes	o__Caudovirales	f__Siphoviridae	g__Hedwigvirus	s__Gordonia virus Hedwig
k__Bacteria	p__Tenericutes	c__Mollicutes	o__Mycoplasmatales	f__Mycoplasmataceae	g__Mycoplasma	s__hyosynoviae
k__Viruses	p__Uroviricota	c__Caudoviricetes	o__Caudovirales	f__Siphoviridae	g__	s__Rhodococcus phage Whack
k__Viruses	p__Uroviricota	c__Caudoviricetes	o__Caudovirales	f__Siphoviridae	g__Skunavirus	s__Lactococcus phage 79201
k__Viruses	p__Uroviricota	c__Caudoviricetes	o__Caudovirales	f__Myoviridae	g__Alexandravirus	s__Aggregatibacter phage S1249
k__Viruses	p__Uroviricota	c__Caudoviricetes	o__Caudovirales	f__Podoviridae	g__	s__Pectobacterium phage vB_PatP_CB4
k__Viruses	p__Uroviricota	c__Caudoviricetes	o__Caudovirales	f__Autographiviridae	g__Drulisvirus	s__Shigella virus SFN6B
k__Viruses	p__Uroviricota	c__Caudoviricetes	o__Caudovirales	f__Autographiviridae	g__Vectrevirus	s__Escherichia virus LL11
k__Viruses	p__Uroviricota	c__Caudoviricetes	o__Caudovirales	f__Podoviridae	g__Sortsnevirus	s__Escherichia virus Sortsne
k__Bacteria	p__Cyanobacteria	c__	o__Synechococcales	f__Merismopediaceae	g__Synechocystis	s__sp. CACIAM 05
k__Bacteria	p__Proteobacteria	c__Gammaproteobacteria	o__Enterobacterales	f__Enterobacteriaceae	g__Lelliottia	s__Enterobacteriaceae endosymbiont of Donacia proxima
k__Bacteria	p__Proteobacteria	c__Gammaproteobacteria	o__Vibrionales	f__Vibrionaceae	g__Photobacterium	s__gaetbulicola
k__Viruses	p__Uroviricota	c__Caudoviricetes	o__Caudovirales	f__Siphoviridae	g__Hendrixvirus	s__Enterobacteria phage mEp235
k__Viruses	p__Uroviricota	c__Caudoviricetes	o__Caudovirales	f__Siphoviridae	g__	s__Streptococcus phage APCM01
k__Bacteria	p__Firmicutes	c__Bacilli	o__Bacillales	f__Paenibacillaceae	g__Paenibacillus	s__sp. lzh-N1
k__Bacteria	p__Proteobacteria	c__Epsilonproteobacteria	o__Campylobacterales	f__Campylobacteraceae	g__Campylobacter	s__cuniculorum
k__Viruses	p__Uroviricota	c__Caudoviricetes	o__Caudovirales	f__Siphoviridae	g__Ceetrepovirus	s__Corynebacterium virus Darwin
k__Bacteria	p__Tenericutes	c__Mollicutes	o__Mycoplasmatales	f__Mycoplasmataceae	g__Mycoplasma	s__bovoculi
k__Bacteria	p__Tenericutes	c__Mollicutes	o__Mycoplasmatales	f__Mycoplasmataceae	g__Mycoplasmopsis	s__glycophila
k__Archaea	p__Crenarchaeota	c__Thermoprotei	o__Thermoproteales	f__Thermoproteaceae	g__Vulcanisaeta	s__distributa
k__Archaea	p__Euryarchaeota	c__Methanococci	o__Methanococcales	f__Methanococcaceae	g__Methanothermococcus	s__okinawensis
k__Viruses	p__Uroviricota	c__Caudoviricetes	o__Caudovirales	f__Siphoviridae	g__Abidjanvirus	s__Pseudomonas virus PaMx11
k__Bacteria	p__Proteobacteria	c__Gammaproteobacteria	o__Vibrionales	f__Vibrionaceae	g__Vibrio	s__sp. HDW18
k__Viruses	p__Uroviricota	c__Caudoviricetes	o__Caudovirales	f__Siphoviridae	g__Wizardvirus	s__Gordonia virus Twister6
k__Bacteria	p__Proteobacteria	c__Alphaproteobacteria	o__Rhizobiales	f__Brucellaceae	g__Brucella	s__suis
k__Viruses	p__Artverviricota	c__Revtraviricetes	o__Ortervirales	f__Retroviridae	g__Gammaretrovirus	s__Feline leukemia virus
k__Bacteria	p__Tenericutes	c__Mollicutes	o__Entomoplasmatales	f__Spiroplasmataceae	g__Spiroplasma	s__turonicum
k__Archaea	p__Crenarchaeota	c__Thermoprotei	o__Acidilobales	f__Acidilobaceae	g__Acidilobus	s__saccharovorans
k__Viruses	p__Uroviricota	c__Caudoviricetes	o__Caudovirales	f__Siphoviridae	g__Maxrubnervirus	s__Achromobacter phage phiAxp-2
k__Viruses	p__Uroviricota	c__Caudoviricetes	o__Caudovirales	f__Myoviridae	g__Moonvirus	s__Citrobacter virus Moon
k__Viruses	p__Uroviricota	c__Caudoviricetes	o__Caudovirales	f__Demerecviridae	g__Tequintavirus	s__Escherichia virus OSYSP
k__Bacteria	p__Spirochaetes	c__Spirochaetia	o__Spirochaetales	f__Borreliaceae	g__Borrelia	s__coriaceae
k__Archaea	p__Crenarchaeota	c__Thermoprotei	o__Desulfurococcales	f__Pyrodictiaceae	g__Pyrodictium	s__delaneyi
k__Viruses	p__Hofneiviricota	c__Faserviricetes	o__Tubulavirales	f__Inoviridae	g__Lineavirus	s__Escherichia virus I22
k__Viruses	p__Uroviricota	c__Caudoviricetes	o__Caudovirales	f__Siphoviridae	g__Skunavirus	s__Lactococcus phage phi145
k__Viruses	p__Uroviricota	c__Caudoviricetes	o__Caudovirales	f__Siphoviridae	g__Saphexavirus	s__Streptococcus virus SPQS1

k__Viruses	p__Uroviricota	c__Caudoviricetes	o__Caudovirales	f__Siphoviridae	g__Sansavirus	s__Caulobacter virus Sansa
k__Viruses	p__Negarnaviricota	c__Ellioviricetes	o__Bunyavirales	f__Phenuiviridae	g__Hudovirus	s__Lepidopteran hudovirus
k__Archaea	p__Crenarchaeota	c__Thermoprotei	o__Sulfolobales	f__Sulfolobaceae	g__Sulfolobus	s__sp. S-194
k__Viruses	p__Uroviricota	c__Caudoviricetes	o__Caudovirales	f__Myoviridae	g__Phapecoctavirus	s__Escherichia virus Schickermooser
k__Viruses	p__Artverviricota	c__Revtraviricetes	o__Ortervirales	f__Nudiviridae	g__	s__Esparto virus
k__Bacteria	p__Cyanobacteria	c__	o__Nostocales	f__Scytonemataceae	g__Brasilonema	s__octagenarum
k__Bacteria	p__Proteobacteria	c__Gammaproteobacteria	o__Thiotrichales	f__Piscirickettsiaceae	g__Thiomicrospira	s__sp. S5
k__Archaea	p__Euryarchaeota	c__Thermococci	o__Thermococcales	f__Thermococcaceae	g__Pyrococcus	s__sp. ST04
k__Bacteria	p__Proteobacteria	c__Gammaproteobacteria	o__Enterobacterales	f__Enterobacteriaceae	g__Candidatus Blochmannia	s__Enterobacteriaceae endosymbiont of Donacia cinerea
k__Viruses	p__Uroviricota	c__Caudoviricetes	o__Caudovirales	f__Podoviridae	g__Luzseptimavirus	s__Pseudomonas virus LUZ7
k__Viruses	p__Uroviricota	c__Caudoviricetes	o__Caudovirales	f__Autographiviridae	g__Berlinvirus	s__Erwinia virus FE44
k__Viruses	p__Uroviricota	c__Caudoviricetes	o__Caudovirales	f__Siphoviridae	g__Poindextervirus	s__Caulobacter virus CcrBL10
k__Viruses	p__Uroviricota	c__Caudoviricetes	o__Caudovirales	f__Siphoviridae	g__Murrayvirus	s__Salmonella virus Lumpael
k__Viruses	p__Uroviricota	c__Caudoviricetes	o__Caudovirales	f__Myoviridae	g__Obolenskvirus	s__Acinetobacter virus WCHABP1
k__Viruses	p__Uroviricota	c__Caudoviricetes	o__Caudovirales	f__Myoviridae	g__Plaisancevirus	s__Pseudomonas virus PMW
k__Bacteria	p__Spirochaetes	c__Spirochaetia	o__Spirochaetales	f__Borreliaceae	g__Borreliella	s__valaisiana
k__Viruses	p__Uroviricota	c__Caudoviricetes	o__Caudovirales	f__Siphoviridae	g__Vieuvirus	s__Acinetobacter phage vB_AbaS_TRS1
k__Bacteria	p__Cyanobacteria	c__	o__Synechococcales	f__Synechococcaceae	g__Synechococcus	s__sp. PCC 8807
k__Viruses	p__Uroviricota	c__Caudoviricetes	o__Caudovirales	f__Siphoviridae	g__Samistivirus	s__Streptomyces virus Paradiddles
k__Viruses	p__Uroviricota	c__Caudoviricetes	o__Caudovirales	f__Siphoviridae	g__Triavirus	s__Staphylococcus phage vB_SauS_phi2
k__Viruses	p__Uroviricota	c__Caudoviricetes	o__Caudovirales	f__Drexlerviridae	g__Webervirus	s__Klebsiella phage vB_KpnS_Alina
k__Viruses	p__Uroviricota	c__Caudoviricetes	o__Caudovirales	f__Myoviridae	g__Hapunavirus	s__Halomonas virus HAP1
k__Bacteria	p__Spirochaetes	c__Spirochaetia	o__Leptospirales	f__Leptospiraceae	g__Leptospira	s__mayottensis
k__Viruses	p__Uroviricota	c__Caudoviricetes	o__Caudovirales	f__Podoviridae	g__Ithacavirus	s__Escherichia virus Pollock

Table S3. Pulmonary function parameters and airway inflammation by asthma status.

	Asthma cases (n=295)	Asthma noncases (n=484)	P-value
Pulmonary function parameters			
FEV_1, ml	2411 ± 879	2795 ± 804	1.92×10^{-9}
FVC, ml	3410 ± 1072	3748 ± 993	1.37×10^{-5}
FEV_1/FVC	0.70 ± 0.11	0.74 ± 0.08	1.50×10^{-8}
FeNO*, ppb	2.94 ± 0.77	2.73 ± 0.61	0.0001

*Fractional exhaled nitric oxide, available in 292 asthma cases and 475 noncases. Values were natural log (ln) transformed for normality.

To examine differences between Asthma cases and controls, a t-test was performed using the function t.test() in R.

Table S4. Taxonomic classification of the 1264 species, from Bacteria and Archaea, included in our metagenome analysis of house dust.

Kingdom	Phylum	Class	Order	Family	Genus	Species
Bacteria	Proteobacteria	Betaproteobacteria	Burkholderiales	Comamonadaceae	Comamonas	kerstersii
Bacteria	Proteobacteria	Betaproteobacteria	Burkholderiales	Oxalobacteraceae	Massilia	violaceinigra
Bacteria	Proteobacteria	Gammaproteobacteria	Xanthomonadales	Rhodanobacteraceae	Rhodanobacter	denitrificans
Bacteria	Proteobacteria	Gammaproteobacteria	Pseudomonadales	Pseudomonadaceae	Pseudomonas	prosekii
Bacteria	Proteobacteria	Alphaproteobacteria	Rhizobiales	Hyphomicrobiaceae	Devosia	sp._I507
Bacteria	Bacteroidetes	Flavobacteriia	Flavobacteriales	Weeksellaceae	Kaistella	daneshvariae
Bacteria	Actinobacteria	Actinobacteria	Corynebacteriales	Nocardiaceae	Rhodococcus	sp._PBTS_1
Bacteria	Proteobacteria	Epsilonproteobacteria	Campylobacterales	Campylobacteraceae	Campylobacter	concisus
Bacteria	Firmicutes	Bacilli	Bacillales	Bacillaceae	Gemella	sanguinis
Bacteria	Proteobacteria	Gammaproteobacteria	Enterobacterales	Erwiniaceae	Mixta	intestinalis
Bacteria	Proteobacteria	Alphaproteobacteria	Sphingomonadales	Sphingomonadaceae	Sphingomonas	sp._HDW15B
Bacteria	Proteobacteria	Betaproteobacteria	Burkholderiales	Comamonadaceae	Variovorax	sp._HW608
Bacteria	Proteobacteria	Epsilonproteobacteria	Campylobacterales	Campylobacteraceae	Arcobacter	cryaerophilus
Bacteria	Bacteroidetes	Bacteroidia	Bacteroidales	Bacteroidaceae	Bacteroides	xylanisolvens
Bacteria	Proteobacteria	Gammaproteobacteria	Enterobacterales	Enterobacteriaceae	Lelliottia	amnigena
Bacteria	Actinobacteria	Actinobacteria	Propionibacteriales	Nocardioidaceae	Aeromicrobium	choanae
Bacteria	Actinobacteria	Actinobacteria	Streptomycetales	Streptomycetaceae	Streptomyces	katrae
Bacteria	Proteobacteria	Gammaproteobacteria	Pseudomonadales	Pseudomonadaceae	Pseudomonas	psychrotolerans
Bacteria	Proteobacteria	Gammaproteobacteria	Enterobacterales	Yersiniaceae	Serratia	odorifera
Bacteria	Firmicutes	Bacilli	Bacillales	Planococcaceae	Planococcus	antarcticus
Bacteria	Actinobacteria	Actinobacteria	Corynebacteriales	Corynebacteriaceae	Corynebacterium	sp._zg-917
Bacteria	Proteobacteria	Gammaproteobacteria	Pseudomonadales	Pseudomonadaceae	Pseudomonas	syringae_group_genomosp._3
Bacteria	Proteobacteria	Gammaproteobacteria	Pseudomonadales	Moraxellaceae	Acinetobacter	sp._NEB149
Bacteria	Proteobacteria	Gammaproteobacteria	Pseudomonadales	Pseudomonadaceae	Pseudomonas	taetrolens
Bacteria	Proteobacteria	Gammaproteobacteria	Pseudomonadales	Pseudomonadaceae	Pseudomonas	sp._NS1(2017)
Bacteria	Firmicutes	Bacilli	Lactobacillales	Lactobacillaceae	Lacticaseibacillus	Lactobacillus_manihotivorans
Bacteria	Bacteroidetes	Bacteroidia	Bacteroidales	Rikenellaceae	Alistipes	megaguti
Bacteria	Actinobacteria	Actinobacteria	Micrococcales	Microbacteriaceae	Microbacterium	sediminis
Bacteria	Proteobacteria	Gammaproteobacteria	Pasteurellales	Pasteurellaceae	Pasteurella	multocida
Bacteria	Proteobacteria	Gammaproteobacteria	Xanthomonadales	Xanthomonadaceae	Xanthomonas	citri
Bacteria	Firmicutes	Bacilli	Bacillales	Staphylococcaceae	Staphylococcus	caprae
Bacteria	Actinobacteria	Actinobacteria	Micrococcales	Brevibacteriaceae	Brevibacterium	sp._o2
Bacteria	Actinobacteria	Actinobacteria	Micromonosporales	Micromonosporaceae	Micromonospora	zamorensis
Bacteria	Firmicutes	Bacilli	Lactobacillales	Lactobacillaceae	Ligilactobacillus	ruminis
Bacteria	Proteobacteria	Gammaproteobacteria	Pseudomonadales	Moraxellaceae	Acinetobacter	sp._FDAARGOS_724
Bacteria	Actinobacteria	Actinobacteria	Corynebacteriales	Tsukamurellaceae	Tsukamurella	tyrosinosolvens
Bacteria	Actinobacteria	Actinobacteria	Propionibacteriales	Nocardioidaceae	Nocardioides	sp._SB3-45
Bacteria	Actinobacteria	Actinobacteria	Streptosporangiales	Nocardiopsaceae	Nocardiopsis	alba
Bacteria	Bacteroidetes	Bacteroidia	Bacteroidales	Bacteroidaceae	Bacteroides	heparinolyticus
Bacteria	Proteobacteria	Alphaproteobacteria	Rhizobiales	Bradyrhizobiaceae	Variibacter	gotjawalensis
Bacteria	Bacteroidetes	Bacteroidia	Bacteroidales	Bacteroidaceae	Phocaeicola	vulgatus
Bacteria	Proteobacteria	Alphaproteobacteria	Rhodobacterales	Rhodobacteraceae	Tabrizicola	piscis
Bacteria	Actinobacteria	Actinobacteria	Streptosporangiales	Streptosporangiaceae	Streptosporangium	sp._caverna
Bacteria	Actinobacteria	Actinobacteria	Actinomycetales	Actinomycetaceae	Actinomyces	sp._oral_taxon_414
Bacteria	Proteobacteria	Gammaproteobacteria	Enterobacterales	Erwiniaceae	Pantoea	eucalypti
Bacteria	Firmicutes	Bacilli	Bacillales	Staphylococcaceae	Staphylococcus	aureus
Bacteria	Actinobacteria	Actinobacteria	Micrococcales	Cellulomonadaceae	Cellulomonas	flavigena
Bacteria	Proteobacteria	Alphaproteobacteria	Sphingomonadales	Sphingomonadaceae	Sphingomonas	alpina
Bacteria	Bacteroidetes	Sphingobacteriia	Sphingobacteriales	Sphingobacteriaceae	Sphingobacterium	mizutaii

Bacteria	Proteobacteria	Gammaproteobacteria	Pseudomonadales	Moraxellaceae	Acinetobacter	bereziniae
Bacteria	Actinobacteria	Actinobacteria	Micrococcales	Micrococcaceae	Kocuria	sp._KD4
Bacteria	Actinobacteria	Actinobacteria	Pseudonocardiales	Pseudonocardiaceae	Saccharomonospora	glauca
Bacteria	Proteobacteria	Alphaproteobacteria	Sphingomonadales	Sphingomonadaceae	Sphingomonas	sp._PAMC26645
Bacteria	Proteobacteria	Alphaproteobacteria	Rhodobacterales	Rhodobacteraceae	Rhodobacter	sphaeroides
Bacteria	Actinobacteria	Actinobacteria	Corynebacteriales	Corynebacteriaceae	Corynebacterium	glyciniphilum
Bacteria	Fusobacteria	Fusobacteriia	Fusobacteriales	Fusobacteriaceae	Fusobacterium	mortiferum
Bacteria	Proteobacteria	Gammaproteobacteria	Pseudomonadales	Pseudomonadaceae	Pseudomonas	graminis
Bacteria	Proteobacteria	Alphaproteobacteria	Rhizobiales	Rhizobiaceae	Rhizobium	flavum
Bacteria	Actinobacteria	Actinobacteria	Micrococcales	Microbacteriaceae	Microbacterium	foliorum
Bacteria	Bacteroidetes	Bacteroidia	Bacteroidales	Bacteroidaceae	Bacteroides	caccae
Bacteria	Proteobacteria	Epsilonproteobacteria	Campylobacterales	Campylobacteraceae	Campylobacter	upsaliensis
Bacteria	Firmicutes	Clostridia	Clostridiales	Peptostreptococcaceae	Peptacetobacter	hiranonis
Bacteria	Bacteroidetes	Cytophagia	Cytophagales	Hymenobacteraceae	Hymenobacter	russus
Bacteria	Firmicutes	Bacilli	Bacillales	Staphylococcaceae	Staphylococcus	hominis
Bacteria	Firmicutes	Bacilli	Bacillales	Bacillaceae	Salicibibacter	halophilus
Bacteria	Actinobacteria	Actinobacteria	Micrococcales	Intrasporangiaceae	Janibacter	indicus
Bacteria	Proteobacteria	Alphaproteobacteria	Rhizobiales	Brucellaceae	Ochrobactrum	pseudogrignonense
Bacteria	Actinobacteria	Actinobacteria	Propionibacteriales	Propionibacteriaceae	Tessaracoccus	aquimaris
Bacteria	Firmicutes	Bacilli	Lactobacillales	Enterococcaceae	Enterococcus	faecalis
Bacteria	Bacteroidetes	Chitinophagia	Chitinophagales	Chitinophagaceae	Flavisolibacter	sp._17J28-1
Bacteria	Actinobacteria	Actinobacteria	Corynebacteriales	Mycobacteriaceae	Mycobacterium	sp._YC-RL4
Bacteria	Firmicutes	Bacilli	Bacillales	Bacillaceae	Bacillus	cereus
Bacteria	Proteobacteria	Alphaproteobacteria	Rhizobiales	Bradyrhizobiaceae	Rhodopseudomonas	palustris
Bacteria	Proteobacteria	Gammaproteobacteria	Pseudomonadales	Pseudomonadaceae	Pseudomonas	nitroreducens
Bacteria	Bacteroidetes	Sphingobacteriia	Sphingobacteriales	Sphingobacteriaceae	Pedobacter	suwonensis
Bacteria	Firmicutes	Bacilli	Bacillales	Bacillaceae	Exiguobacterium	sp._Helios
Bacteria	Firmicutes	Bacilli	Bacillales	Thermoactinomycetaceae	Thermoactinomyces	vulgaris
Bacteria	Firmicutes	Bacilli	Lactobacillales	Lactobacillaceae	Lactobacillus	crispatus
Bacteria	Proteobacteria	Alphaproteobacteria	Rhodobacterales	Rhodobacteraceae	Roseivivax	sp._THAF30
Bacteria	Actinobacteria	Actinobacteria	Micrococcales	Cellulomonadaceae	Oerskovia	sp._KBS0722
Bacteria	Actinobacteria	Actinobacteria	Micrococcales	Intrasporangiaceae	Serinicoccus	profundi
Bacteria	Actinobacteria	Actinobacteria	Micrococcales	Microbacteriaceae	Microbacterium	testaceum
Bacteria	Actinobacteria	Actinobacteria	Micrococcales	Microbacteriaceae	Agrococcus	jejuensis
Bacteria	Bacteroidetes	Flavobacteriia	Flavobacteriales	Flavobacteriaceae	Flavobacterium	johnsoniae
Bacteria	Firmicutes	Clostridia	Clostridiales	Peptostreptococcaceae	Massilistercora	timonensis
Bacteria	Actinobacteria	Actinobacteria	Bifidobacteriales	Bifidobacteriaceae	Bifidobacterium	breve
Bacteria	Firmicutes	Clostridia	Clostridiales	Lachnospiraceae	Lachnospira	eligens
Bacteria	Proteobacteria	Alphaproteobacteria	Rhizobiales	Methylobacteriaceae	Methylobacterium	sp._AMS5
Bacteria	Actinobacteria	Actinobacteria	Corynebacteriales	Nocardiaceae	Rhodococcus	sp._DMU1
Bacteria	Actinobacteria	Actinobacteria	Micrococcales	Microbacteriaceae	Leucobacter	muris
Bacteria	Proteobacteria	Gammaproteobacteria	Pasteurellales	Pasteurellaceae	Haemophilus	haemolyticus
Bacteria	Actinobacteria	Actinobacteria	Micrococcales	Microbacteriaceae	Microbacterium	paraoxydans
Archaea	Euryarchaeota	Halobacteria	Natrialbales	Natrialbaceae	Haloterrigena	daqingensis
Bacteria	Actinobacteria	Actinobacteria	Actinomycetales	Actinomycetaceae	Actinomyces	viscosus
Bacteria	Firmicutes	Bacilli	Lactobacillales	Lactobacillaceae	Lentilactobacillus	Lactobacillus_buchneri
Bacteria	Firmicutes	Bacilli	Lactobacillales	Lactobacillaceae	Lactobacillus	johnsonii
Bacteria	Firmicutes	Bacilli	Bacillales	Listeriaceae	Brochothrix	thermosphacta
Bacteria	Proteobacteria	Betaproteobacteria	Burkholderiales	Oxalobacteraceae	Duganella	sp._AF9R3
Bacteria	Firmicutes	Bacilli	Bacillales	Bacillaceae	Exiguobacterium	sp._MH3
Bacteria	Proteobacteria	Alphaproteobacteria	Caulobacterales	Caulobacteraceae	Brevundimonas	vesicularis
Bacteria	Proteobacteria	Epsilonproteobacteria	Campylobacterales	Helicobacteraceae	Helicobacter	canis

Bacteria	Proteobacteria	Gammaproteobacteria	Pseudomonadales	Pseudomonadaceae	Pseudomonas	luteola
Bacteria	Actinobacteria	Actinobacteria	Micrococcales	Micrococcaceae	Pseudarthrobacter	equi
Bacteria	Actinobacteria	Actinobacteria	Micrococcales	Dermacoccaceae	Dermacoccus	nishinomiyaensis
Bacteria	Proteobacteria	Gammaproteobacteria	Enterobacterales	Enterobacteriaceae	Pseudescherichia	vulneris
Bacteria	Actinobacteria	Actinobacteria	Micrococcales	Intrasporangiaceae	Intrasporangium	calvum
Bacteria	Firmicutes	Bacilli	Lactobacillales	Leuconostocaceae	Leuconostoc	mesenteroides
Bacteria	Firmicutes	Bacilli	Lactobacillales	Lactobacillaceae	Lactobacillus	acidophilus
Bacteria	Actinobacteria	Actinobacteria	Micrococcales	Micrococcaceae	Paenarthrobacter	sp._YJN-D
Bacteria	Proteobacteria	Gammaproteobacteria	Pseudomonadales	Pseudomonadaceae	Pseudomonas	lurida
Bacteria	Actinobacteria	Actinobacteria	Streptosporangiales	Nocardiopsaceae	Thermobifida	fusca
Bacteria	Actinobacteria	Actinobacteria	Micrococcales	Micrococcaceae	Kocuria	sp._BT304
Bacteria	Bacteroidetes	Flavobacteriia	Flavobacteriales	Weeksellaceae	Cruoricaptor	ignavus
Bacteria	Actinobacteria	Actinobacteria	Corynebacteriales	Corynebacteriaceae	Corynebacterium	simulans
Bacteria	Proteobacteria	Gammaproteobacteria	Pseudomonadales	Pseudomonadaceae	Pseudomonas	sp._MRSN12121
Bacteria	Actinobacteria	Actinobacteria	Micrococcales	Microbacteriaceae	Plantibacter	flavus
Bacteria	Firmicutes	Bacilli	Lactobacillales	Streptococcaceae	Lactococcus	raffinolactis
Bacteria	Actinobacteria	Actinobacteria	Corynebacteriales	Dietziaceae	Dietzia	psychralcaliphila
Bacteria	Proteobacteria	Gammaproteobacteria	Pseudomonadales	Pseudomonadaceae	Pseudomonas	entomophila
Bacteria	Actinobacteria	Actinobacteria	Micrococcales	Intrasporangiaceae	Arsenicicoccus	sp._oral_taxon_190
Bacteria	Actinobacteria	Actinobacteria	Micrococcales	Microbacteriaceae	Frondihabitans	sp._PAMC_28766
Bacteria	Proteobacteria	Gammaproteobacteria	Pseudomonadales	Pseudomonadaceae	Pseudomonas	migulae
Bacteria	Firmicutes	Erysipelotrichia	Erysipelotrichales	Erysipelotrichaceae	Erysipelatoclostridium	Clostridium_innocuum
Bacteria	Firmicutes	Bacilli	Bacillales	Staphylococcaceae	Staphylococcus	lugdunensis
Bacteria	Proteobacteria	Gammaproteobacteria	Enterobacterales	Yersiniaceae	Serratia	sp._FDAARGOS_506
Bacteria	Proteobacteria	Alphaproteobacteria	Rhizobiales	Methylobacteriaceae	Methylobacterium	durans
Bacteria	Actinobacteria	Actinobacteria	Corynebacteriales	Nocardiaceae	Nocardia	yunnanensis
Bacteria	Actinobacteria	Actinobacteria	Micromonosporales	Micromonosporaceae	Actinoplanes	sp._N902-109
Bacteria	Actinobacteria	Actinobacteria	Micrococcales	Dermabacteraceae	Brachybacterium	saurashtrense
Bacteria	Actinobacteria	Actinobacteria	Propionibacteriales	Nocardioidaceae	Kribbella	qitaiheensis
Bacteria	Bacteroidetes	Flavobacteriia	Flavobacteriales	Blattabacteriaceae	Blattabacterium	cuenoti
Bacteria	Actinobacteria	Actinobacteria	Micrococcales	Cellulomonadaceae	Cellulomonas	sp._H30R-01
Bacteria	Bacteroidetes	Cytophagia	Cytophagales	Cytophagaceae	Fibrella	sp._ES10-3-2-2
Bacteria	Proteobacteria	Alphaproteobacteria	Sphingomonadales	Sphingomonadaceae	Sphingomonas	sp._IC081
Bacteria	Actinobacteria	Actinobacteria	Propionibacteriales	Propionibacteriaceae	Auraticoccus	monumenti
Bacteria	Actinobacteria	Actinobacteria	Actinomycetales	Actinomycetaceae	Flaviflexus	sp._H23T48
Bacteria	Actinobacteria	Actinobacteria	Corynebacteriales	Corynebacteriaceae	Corynebacterium	glaucum
Bacteria	Proteobacteria	Betaproteobacteria	Burkholderiales	Burkholderiaceae	Burkholderia	gladioli
Bacteria	Actinobacteria	Actinobacteria	Corynebacteriales	Corynebacteriaceae	Corynebacterium	kutscheri
Bacteria	Actinobacteria	Actinobacteria	Micrococcales	Promicromonosporaceae	Cellulosimicrobium	sp._BI34T
Bacteria	Bacteroidetes	Bacteroidia	Bacteroidales	Bacteroidaceae	Bacteroides	uniformis
Bacteria	Firmicutes	Bacilli	Lactobacillales	Streptococcaceae	Streptococcus	vestibularis
Bacteria	Actinobacteria	Actinobacteria	Micrococcales	Micrococcaceae	Kocuria	indica
Bacteria	Firmicutes	Bacilli	Lactobacillales	Leuconostocaceae	Leuconostoc	pseudomesenteroides
Bacteria	Actinobacteria	Actinobacteria	Corynebacteriales	Corynebacteriaceae	Corynebacterium	endometrii
Bacteria	Bacteroidetes	Cytophagia	Cytophagales	Hymenobacteraceae	Hymenobacter	sedentarius
Bacteria	Proteobacteria	Alphaproteobacteria	Rhizobiales	Methylobacteriaceae	Microvirga	ossetica
acteria	Actinobacteria	Actinobacteria	Micrococcales	Intrasporangiaceae	Phycicoccus	endophyticus
Bacteria	Actinobacteria	Actinobacteria	Nakamurellales	Nakamurellaceae	Nakamurella	multipartita
Bacteria	Proteobacteria	Gammaproteobacteria	Pseudomonadales	Pseudomonadaceae	Pseudomonas	versuta
Bacteria	Firmicutes	Bacilli	Bacillales	Bacillaceae	Exiguobacterium	sp._AT1b
Bacteria	Actinobacteria	Actinobacteria	Micrococcales	Microbacteriaceae	Cryobacterium	arcticum
Bacteria	Proteobacteria	Alphaproteobacteria	Rhizobiales	Rhizobiaceae	Agrobacterium	larrymoorei

Bacteria	Bacteroidetes	Bacteroidia	Bacteroidales	Bacteroidaceae	Bacteroides	sp._HF-5287
Bacteria	Actinobacteria	Actinobacteria	Propionibacteriales	Nocardioidaceae	Micropruina	glycogenica
Bacteria	Firmicutes	Bacilli	Bacillales	Staphylococcaceae	Staphylococcus	auricularis
Bacteria	Verrucomicrobia	Verrucomicrobiae	Verrucomicrobiales	Akkermansiaceae	Akkermansia	muciniphila
Bacteria	Proteobacteria	Gammaproteobacteria	Pseudomonadales	Pseudomonadaceae	Pseudomonas	sp._02C_26
Bacteria	Actinobacteria	Actinobacteria	Micrococcales	Microbacteriaceae	Microbacterium	esteraromaticum
Bacteria	Proteobacteria	Gammaproteobacteria	Pseudomonadales	Pseudomonadaceae	Pseudomonas	fluorescens
Bacteria	Actinobacteria	Actinobacteria	Propionibacteriales	Nocardioidaceae	Nocardioides	sp._603
Bacteria	Proteobacteria	Gammaproteobacteria	Pseudomonadales	Moraxellaceae	Acinetobacter	radioresistens
Bacteria	Proteobacteria	Betaproteobacteria	Burkholderiales	Comamonadaceae	Ottowia	oryzae
Bacteria	Proteobacteria	Gammaproteobacteria	Pseudomonadales	Pseudomonadaceae	Pseudomonas	moraviensis
Bacteria	Proteobacteria	Alphaproteobacteria	Sphingomonadales	Sphingomonadaceae	Sphingopyxis	fribergensis
Bacteria	Firmicutes	Bacilli	Lactobacillales	Enterococcaceae	Enterococcus	casseliflavus
Bacteria	Proteobacteria	Gammaproteobacteria	Pasteurellales	Pasteurellaceae	Frederiksenia	canicola
Bacteria	Proteobacteria	Alphaproteobacteria	Caulobacterales	Caulobacteraceae	Caulobacter	sp._Ji-3-8
Bacteria	Firmicutes	Bacilli	Bacillales	Staphylococcaceae	Staphylococcus	sp._23_2_7_LY
Bacteria	Firmicutes	Bacilli	Lactobacillales	Leuconostocaceae	Weissella	jogaejeotgali
Bacteria	Proteobacteria	Alphaproteobacteria	Rhizobiales	Bradyrhizobiaceae	Bosea	sp._Tri-49
Bacteria	Proteobacteria	Alphaproteobacteria	Caulobacterales	Caulobacteraceae	Brevundimonas	mediterranea
Bacteria	Proteobacteria	Alphaproteobacteria	Sphingomonadales	Sphingomonadaceae	Novosphingobium	pentaromativorans
Bacteria	Proteobacteria	Alphaproteobacteria	Rhodobacterales	Rhodobacteraceae	Paracoccus	contaminans
Bacteria	Proteobacteria	Alphaproteobacteria	Rhizobiales	Rhizobiaceae	Shinella	sp._HZN7
Bacteria	Firmicutes	Bacilli	Lactobacillales	Enterococcaceae	Enterococcus	hirae
Bacteria	Firmicutes	Clostridia	Clostridiales	Lachnospiraceae	Blautia	sp._SC05B48
Bacteria	Proteobacteria	Gammaproteobacteria	Enterobacterales	Yersiniaceae	Serratia	marcescens
Bacteria	Proteobacteria	Gammaproteobacteria	Pseudomonadales	Moraxellaceae	Acinetobacter	sp._ACNIH1
Bacteria	Proteobacteria	Betaproteobacteria	Burkholderiales	Comamonadaceae	Polaromonas	sp._JS666
Bacteria	Proteobacteria	Betaproteobacteria	Burkholderiales	Comamonadaceae	Acidovorax	avenae
Bacteria	Actinobacteria	Actinobacteria	Actinomycetales	Actinomycetaceae	Actinomyces	israelii
Bacteria	Proteobacteria	Alphaproteobacteria	Rhodospirillales	Acetobacteraceae	Acetobacter	pasteurianus
Bacteria	Proteobacteria	Betaproteobacteria	Burkholderiales	Comamonadaceae	Delftia	tsuruhatensis
Bacteria	Actinobacteria	Actinobacteria	Streptomycetales	Streptomycetaceae	Streptomyces	sp._NEAU-sy36
Bacteria	Firmicutes	Clostridia	Clostridiales	Lachnospiraceae	Lachnoclostridium	sp._YL32
Bacteria	Proteobacteria	Gammaproteobacteria	Pseudomonadales	Moraxellaceae	Psychrobacter	arcticus
Bacteria	Proteobacteria	Alphaproteobacteria	Rhizobiales	Hyphomicrobiaceae	Devosia	sp._A16
Bacteria	Proteobacteria	Gammaproteobacteria	Xanthomonadales	Xanthomonadaceae	Stenotrophomonas	maltophilia
Bacteria	Bacteroidetes	Flavobacteriia	Flavobacteriales	Blattabacteriaceae	Candidatus_Sulcia	muelleri
Bacteria	Proteobacteria	Gammaproteobacteria	Xanthomonadales	Xanthomonadaceae	Luteimonas	yindakuii
Bacteria	Proteobacteria	Alphaproteobacteria	Sphingomonadales	Sphingomonadaceae	Novosphingobium	sp._P6W
Bacteria	Firmicutes	Clostridia	Clostridiales	Ruminococcaceae	Ruminococcus	bicirculans
Bacteria	Proteobacteria	Alphaproteobacteria	Sphingomonadales	Sphingomonadaceae	Sphingopyxis	macrogoltabida
Bacteria	Proteobacteria	Alphaproteobacteria	Rhodobacterales	Rhodobacteraceae	Paracoccus	yeei
Bacteria	Acidobacteria	Vicinamibacteria		Vicinamibacteraceae	Luteitalea	pratensis
Bacteria	Proteobacteria	Alphaproteobacteria	Caulobacterales	Caulobacteraceae	Brevundimonas	sp._scallop
Bacteria	Proteobacteria	Alphaproteobacteria	Sphingomonadales	Sphingomonadaceae	Sphingomonas	rhizophila
Bacteria	Actinobacteria	Actinobacteria	Propionibacteriales	Propionibacteriaceae	Tessaracoccus	flavus
Bacteria	Proteobacteria	Epsilonproteobacteria	Campylobacterales	Campylobacteraceae	Campylobacter	hominis
Bacteria	Proteobacteria	Alphaproteobacteria	Rickettsiales	Anaplasmataceae	Wolbachia	endosymbiont_of_Ctenocephalides_felis_wCfeT
Bacteria	Actinobacteria	Actinobacteria	Bifidobacteriales	Bifidobacteriaceae	Bifidobacterium	pseudocatenulatum
Bacteria	Actinobacteria	Actinobacteria	Corynebacteriales	Gordoniaceae	Gordonia	sp._KTR9
Bacteria	Firmicutes	Bacilli	Lactobacillales	Lactobacillaceae	Limosilactobacillus	Lactobacillus_vaginalis
Bacteria	Bacteroidetes	Flavobacteriia	Flavobacteriales	Weeksellaceae	Epilithonimonas	vandammei

Bacteria	Actinobacteria	Actinobacteria	Actinomycetales	Actinomycetaceae	Flaviflexus	sp._dk850
Bacteria	Proteobacteria	Alphaproteobacteria	Rhodobacterales	Rhodobacteraceae	Paracoccus	sp._BM15
Bacteria	Actinobacteria	Actinobacteria	Corynebacteriales	Mycobacteriaceae	Mycobacterium	kansasii
Bacteria	Actinobacteria	Actinobacteria	Propionibacteriales	Nocardioidaceae	Aeromicrobium	erythreum
Bacteria	Proteobacteria	Betaproteobacteria	Burkholderiales	Oxalobacteraceae	Duganella	sp._GN2-R2
Bacteria	Actinobacteria	Actinobacteria	Propionibacteriales	Nocardioidaceae	Nocardioides	sp._HDW12A
Bacteria	Actinobacteria	Actinobacteria	Micrococcales	Sanguibacteraceae	Sanguibacter	keddieii
Bacteria	Actinobacteria	Actinobacteria	Corynebacteriales	Mycobacteriaceae	Mycolicibacterium	madagascariense
Bacteria	Actinobacteria	Actinobacteria	Corynebacteriales	Mycobacteriaceae	Mycolicibacterium	sediminis
Bacteria	Proteobacteria	Gammaproteobacteria	Pseudomonadales	Moraxellaceae	Psychrobacter	alimentarius
Bacteria	Proteobacteria	Alphaproteobacteria	Sphingomonadales	Sphingomonadaceae	Sphingosinithalassobacter	sp._CS137
Bacteria	Proteobacteria	Betaproteobacteria	Burkholderiales	Oxalobacteraceae	Janthinobacterium	agaricidamnosum
Bacteria	Cyanobacteria		Chroococcidiopsidales	Chroococcidiopsidaceae	Chroococcidiopsis	thermalis
Bacteria	Bacteroidetes	Flavobacteriia	Flavobacteriales	Flavobacteriaceae	Capnocytophaga	cynodegmi
Bacteria	Bacteroidetes	Sphingobacteriia	Sphingobacteriales	Sphingobacteriaceae	Sphingobacterium	lactis
Bacteria	Actinobacteria	Actinobacteria	Micrococcales	Microbacteriaceae	Curtobacterium	sp._csp3
Bacteria	Proteobacteria	Gammaproteobacteria	Pseudomonadales	Pseudomonadaceae	Pseudomonas	simiae
Bacteria	Actinobacteria	Actinobacteria	Propionibacteriales	Nocardioidaceae	Nocardioides	mesophilus
Bacteria	Proteobacteria	Alphaproteobacteria	Rhizobiales	Rhizobiaceae	Neorhizobium	galegae
Bacteria	Proteobacteria	Betaproteobacteria	Burkholderiales	Burkholderiaceae	Lautropia	mirabilis
Bacteria	Proteobacteria	Gammaproteobacteria	Enterobacterales	Enterobacteriaceae	Enterobacter	asburiae
Bacteria	Actinobacteria	Actinobacteria	Micrococcales	Microbacteriaceae	Microbacterium	sp._YJN-G
Bacteria	Bacteroidetes	Cytophagia	Cytophagales	Cytophagaceae	Rhodocytophaga	rosea
Bacteria	Proteobacteria	Gammaproteobacteria	Xanthomonadales	Xanthomonadaceae	Xanthomonas	oryzae
Bacteria	Firmicutes	Negativicutes	Veillonellales	Veillonellaceae	Veillonella	nakazawae
Bacteria	Proteobacteria	Gammaproteobacteria	Xanthomonadales	Rhodanobacteraceae	Luteibacter	pinisoli
Bacteria	Proteobacteria	Gammaproteobacteria	Xanthomonadales	Xanthomonadaceae	Stenotrophomonas	rhizophila
Bacteria	Actinobacteria	Actinobacteria	Micrococcales	Brevibacteriaceae	Brevibacterium	luteolum
Bacteria	Planctomycetes	Planctomycetia	Isosphaerales	Isosphaeraceae	Aquisphaera	giovannonii
Bacteria	Actinobacteria	Actinobacteria	Micrococcales	Microbacteriaceae	Microbacterium	sp._Y-01
Bacteria	Actinobacteria	Actinobacteria	Micromonosporales	Micromonosporaceae	Actinoplanes	derwentensis
Bacteria	Actinobacteria	Rubrobacteria	Rubrobacterales	Baekduiaceae	Baekduia	soli
Bacteria	Proteobacteria	Gammaproteobacteria	Enterobacterales	Enterobacteriaceae	Klebsiella	variicola
Bacteria	Proteobacteria	Gammaproteobacteria	Pseudomonadales	Moraxellaceae	Moraxella	osloensis
Bacteria	Proteobacteria	Gammaproteobacteria	Pseudomonadales	Pseudomonadaceae	Pseudomonas	sp._OIL-1
Bacteria	Actinobacteria	Actinobacteria	Corynebacteriales	Gordoniaceae	Gordonia	terrae
Bacteria	Bacteroidetes	Flavobacteriia	Flavobacteriales	Weeksellaceae	Chryseobacterium	indologenes
Bacteria	Actinobacteria	Actinobacteria	Actinomycetales	Actinomycetaceae	Pauljensenia	hongkongensis
Bacteria	Actinobacteria	Actinobacteria	Micrococcales	Intrasporangiaceae	Phycicoccus	sp._HDW14
Bacteria	Proteobacteria	Deltaproteobacteria	Myxococcales	Archangiaceae	Stigmatella	aurantiaca
Bacteria	Proteobacteria	Gammaproteobacteria	Enterobacterales	Yersiniaceae	Rahnella	aquatilis
Bacteria	Bacteroidetes	Cytophagia	Cytophagales	Hymenobacteraceae	Hymenobacter	baengnokdamensis
Bacteria	Proteobacteria	Gammaproteobacteria	Pseudomonadales	Moraxellaceae	Acinetobacter	sp._NEB_394
Bacteria	Firmicutes	Clostridia	Clostridiales	Peptostreptococcaceae	Romboutsia	ilealis
Bacteria	Actinobacteria	Actinobacteria	Corynebacteriales	Corynebacteriaceae	Corynebacterium	pseudotuberculosis
Bacteria	Proteobacteria	Gammaproteobacteria	Aeromonadales	Aeromonadaceae	Aeromonas	salmonicida
Bacteria	Actinobacteria	Actinobacteria	Micrococcales	Beutenbergiaceae	Miniimonas	sp._S16
Bacteria	Proteobacteria	Gammaproteobacteria	Enterobacterales	Enterobacteriaceae	Enterobacter	cloacae
Bacteria	Actinobacteria	Actinobacteria	Pseudonocardiales	Pseudonocardiaceae	Pseudonocardia	sp._HH130630-07
Bacteria	Proteobacteria	Gammaproteobacteria	Enterobacterales	Erwiniaceae	Pantoea	rwandensis
Bacteria	Firmicutes	Bacilli	Lactobacillales	Streptococcaceae	Streptococcus	intermedius
Bacteria	Actinobacteria	Actinobacteria	Bifidobacteriales	Bifidobacteriaceae	Bifidobacterium	catenulatum

Bacteria	Proteobacteria	Gammaproteobacteria	Enterobacterales	Erwiniaceae	Pantoea	sp._CCBC3-3-1
Bacteria	Actinobacteria	Actinobacteria	Micrococcales	Micrococcaceae	Arthrobacter	crystallopoietes
Bacteria	Firmicutes	Bacilli	Lactobacillales	Streptococcaceae	Streptococcus	australis
Bacteria	Proteobacteria	Gammaproteobacteria	Xanthomonadales	Xanthomonadaceae	Luteimonas	granuli
Bacteria	Proteobacteria	Alphaproteobacteria	Rhizobiales	Bradyrhizobiaceae	Bradyrhizobium	erythrophlei
Bacteria	Actinobacteria	Actinobacteria	Micrococcales	Microbacteriaceae	Plantibacter	sp._PA-3-X8
Bacteria	Actinobacteria	Actinobacteria	Pseudonocardiales	Pseudonocardiaceae	Pseudonocardia	sp._CGMCC_4.1532
Bacteria	Proteobacteria	Alphaproteobacteria	Sphingomonadales	Sphingomonadaceae	Sphingobium	sp._YG1
Bacteria	Proteobacteria	Alphaproteobacteria	Rickettsiales	Rickettsiaceae	Rickettsia	endosymbiont_of_Bemisia_tabaci
Bacteria	Actinobacteria	Actinobacteria	Geodermatophilales	Geodermatophilaceae	Geodermatophilus	obscurus
Bacteria	Proteobacteria	Betaproteobacteria	Burkholderiales		Methylibium	sp._Pch-M
Bacteria	Proteobacteria	Gammaproteobacteria	Pseudomonadales	Moraxellaceae	Acinetobacter	seifertii
Bacteria	Proteobacteria	Gammaproteobacteria	Enterobacterales	Yersiniaceae	Serratia	symbiotica
Bacteria	Proteobacteria	Betaproteobacteria	Neisseriales	Neisseriaceae	Neisseria	subflava
Bacteria	Actinobacteria	Actinobacteria	Corynebacteriales	Gordoniaceae	Gordonia	polyisoprenivorans
Bacteria	Firmicutes	Clostridia	Clostridiales	Lachnospiraceae	Anaerostipes	hadrus
Bacteria	Firmicutes	Bacilli	Lactobacillales	Streptococcaceae	Streptococcus	sp._LPB0220
Bacteria	Actinobacteria	Actinobacteria	Corynebacteriales	Corynebacteriaceae	Corynebacterium	timonense
Bacteria	Proteobacteria	Gammaproteobacteria	Enterobacterales	Yersiniaceae	Rouxiella	badensis
Bacteria	Proteobacteria	Alphaproteobacteria	Rhizobiales	Methylobacteriaceae	Methylobacterium	currus
Bacteria	Bacteroidetes	Cytophagia	Cytophagales	Hymenobacteraceae	Adhaeribacter	sp._KUDC8001
Bacteria	Actinobacteria	Actinobacteria	Corynebacteriales	Corynebacteriaceae	Corynebacterium	doosanense
Bacteria	Proteobacteria	Betaproteobacteria	Burkholderiales	Comamonadaceae	Acidovorax	sp._16-35-5
Bacteria	Bacteroidetes	Flavobacteriia	Flavobacteriales	Flavobacteriaceae	Capnocytophaga	sp._FDAARGOS_737
Bacteria	Proteobacteria	Betaproteobacteria	Burkholderiales	Comamonadaceae	Comamonas	testosteroni
Bacteria	Proteobacteria	Alphaproteobacteria	Rhizobiales	Rhizobiaceae	Rhizobium	sp._007
Bacteria	Actinobacteria	Actinobacteria	Actinomycetales	Actinomycetaceae	Actinomyces	sp._oral_taxon_897
Bacteria	Firmicutes	Bacilli	Lactobacillales	Streptococcaceae	Streptococcus	agalactiae
Bacteria	Bacteroidetes	Flavobacteriia	Flavobacteriales	Flavobacteriaceae	Flavobacterium	sp._KBS0721
Bacteria	Bacteroidetes	Bacteroidia	Bacteroidales	Porphyromonadaceae	Porphyromonas	gingivalis
Bacteria	Firmicutes	Bacilli	Lactobacillales	Streptococcaceae	Streptococcus	salivarius
Bacteria	Proteobacteria	Gammaproteobacteria	Pseudomonadales	Pseudomonadaceae	Pseudomonas	koreensis
Bacteria	Proteobacteria	Gammaproteobacteria	Pseudomonadales	Moraxellaceae	Acinetobacter	lwoffii
Bacteria	Actinobacteria	Actinobacteria	Propionibacteriales	Propionibacteriaceae	Propionimicrobium	sp._Marseille-P3275
Bacteria	Proteobacteria	Gammaproteobacteria	Pseudomonadales	Moraxellaceae	Psychrobacter	sp._DAB_AL43B
Bacteria	Proteobacteria	Alphaproteobacteria	Rhizobiales	Methylobacteriaceae	Methylobacterium	sp._17Sr1-43
Bacteria	Actinobacteria	Actinobacteria	Pseudonocardiales	Pseudonocardiaceae	Saccharomonospora	viridis
Bacteria	Actinobacteria	Actinobacteria	Micrococcales	Brevibacteriaceae	Brevibacterium	sp._WO024
Bacteria	Actinobacteria	Actinobacteria	Micrococcales	Microbacteriaceae	Microbacterium	sp._BH-3-3-3
Bacteria	Proteobacteria	Gammaproteobacteria	Enterobacterales	Enterobacteriaceae	Klebsiella	oxytoca
Bacteria	Actinobacteria	Actinobacteria	Corynebacteriales	Nocardiaceae	Rhodococcus	sp._B7740
Bacteria	Proteobacteria	Alphaproteobacteria	Rhizobiales	Bradyrhizobiaceae	Bosea	sp._RAC05
Bacteria	Proteobacteria	Gammaproteobacteria	Enterobacterales	Erwiniaceae	Pantoea	sp._PSNIH1
Bacteria	Actinobacteria	Actinobacteria	Micrococcales	Micrococcaceae	Arthrobacter	sp._U41
Bacteria	Firmicutes	Bacilli	Lactobacillales	Lactobacillaceae	Lactobacillus	sp._3B(2020)
Bacteria	Actinobacteria	Actinobacteria	Micrococcales	Microbacteriaceae	Protaetiibacter	intestinalis
Bacteria	Actinobacteria	Actinobacteria	Micromonosporales	Micromonosporaceae	Micromonospora	tulbaghiae
Bacteria	Firmicutes	Bacilli	Bacillales	Staphylococcaceae	Staphylococcus	felis
Bacteria	Proteobacteria	Gammaproteobacteria	Pseudomonadales	Pseudomonadaceae	Pseudomonas	sp._SNU_WT1
Bacteria	Actinobacteria	Actinobacteria	Corynebacteriales	Nocardiaceae	Rhodococcus	rhodochrous
Bacteria	Actinobacteria	Actinobacteria	Micrococcales	Intrasporangiaceae	Janibacter	limosus
Bacteria	Firmicutes	Negativicutes	Veillonellales	Veillonellaceae	Veillonella	parvula

Bacteria	Proteobacteria	Gammaproteobacteria	Enterobacterales	Erwiniaceae	Pantoea	sp._SO10
Bacteria	Firmicutes	Bacilli	Lactobacillales	Leuconostocaceae	Weissella	cibaria
Bacteria	Actinobacteria	Actinobacteria	Micrococcales	Dermabacteraceae	Brachybacterium	faecium
Bacteria	Actinobacteria	Actinobacteria	Corynebacteriales	Corynebacteriaceae	Corynebacterium	jeikeium
Bacteria	Bacteroidetes	Flavobacteriia	Flavobacteriales	Weeksellaceae	Chryseobacterium	balustinum
Bacteria	Proteobacteria	Alphaproteobacteria	Sphingomonadales	Sphingomonadaceae	Sphingomonas	sp._LMO-1
Bacteria	Proteobacteria	Alphaproteobacteria	Sphingomonadales	Sphingomonadaceae	Sphingomonas	melonis
Bacteria	Bacteroidetes	Bacteroidia	Bacteroidales	Prevotellaceae	Prevotella	melaninogenica
Bacteria	Firmicutes	Bacilli	Bacillales	Planococcaceae	Exiguobacterium	sibiricum
Bacteria	Actinobacteria	Actinobacteria	Propionibacteriales	Nocardioidaceae	Kribbella	flavida
Bacteria	Bacteroidetes	Flavobacteriia	Flavobacteriales	Weeksellaceae	Empedobacter	falsenii
Bacteria	Actinobacteria	Actinobacteria	Micrococcales	Dermacoccaceae	Kytococcus	sedentarius
Bacteria	Proteobacteria	Gammaproteobacteria	Xanthomonadales	Xanthomonadaceae	Luteimonas	sp._MC1825
Bacteria	Bacteroidetes	Bacteroidia	Bacteroidales	Bacteroidaceae	Bacteroides	caecimuris
Bacteria	Proteobacteria	Alphaproteobacteria	Sphingomonadales	Sphingomonadaceae	Sphingomonas	sanxanigenens
Bacteria	Actinobacteria	Actinobacteria	Micrococcales	Microbacteriaceae	Rathayibacter	sp._VKM_Ac-2804
Bacteria	Proteobacteria	Alphaproteobacteria	Rhizobiales	Rhizobiaceae	Rhizobium	pusense
Bacteria	Bacteroidetes	Sphingobacteriia	Sphingobacteriales	Sphingobacteriaceae	Sphingobacterium	thalpophilum
Bacteria	Firmicutes	Bacilli	Bacillales	Bacillaceae	Bacillus	pumilus
Bacteria	Proteobacteria	Alphaproteobacteria	Rhizobiales	Bradyrhizobiaceae	Bradyrhizobium	japonicum
Bacteria	Bacteroidetes	Bacteroidia	Bacteroidales	Prevotellaceae	Prevotella	dentalis
Bacteria	Actinobacteria	Actinobacteria	Corynebacteriales	Corynebacteriaceae	Corynebacterium	ureicelerivorans
Bacteria	Firmicutes	Clostridia	Clostridiales	Lachnospiraceae	Anaerobutyricum	hallii
Bacteria	Proteobacteria	Alphaproteobacteria	Rhizobiales	Methylobacteriaceae	Methylobacterium	radiotolerans
Bacteria	Proteobacteria	Betaproteobacteria	Burkholderiales	Oxalobacteraceae	Massilia	putida
Bacteria	Firmicutes	Bacilli	Bacillales	Planococcaceae	Planococcus	sp._MB-3u-03
Bacteria	Bacteroidetes	Flavobacteriia	Flavobacteriales	Flavobacteriaceae	Flavobacterium	sp._MDT1-60
Bacteria	Firmicutes	Bacilli	Lactobacillales	Carnobacteriaceae	Jeotgalibaca	sp._PTS2502
Bacteria	Actinobacteria	Actinobacteria	Bifidobacteriales	Bifidobacteriaceae	Bifidobacterium	adolescentis
Bacteria	Actinobacteria	Actinobacteria	Micrococcales	Microbacteriaceae	Agrococcus	carbonis
Bacteria	Proteobacteria	Betaproteobacteria	Burkholderiales	Oxalobacteraceae	Massilia	plicata
Bacteria	Actinobacteria	Thermoleophilia	Solirubrobacterales	Conexibacteraceae	Conexibacter	woesei
Bacteria	Firmicutes	Bacilli	Lactobacillales	Carnobacteriaceae	Carnobacterium	sp._17-4
Bacteria	Actinobacteria	Actinobacteria	Micrococcales	Intrasporangiaceae	Janibacter	melonis
Bacteria	Actinobacteria	Actinobacteria	Corynebacteriales	Corynebacteriaceae	Corynebacterium	efficiens
Bacteria	Actinobacteria	Actinobacteria	Micrococcales	Promicromonosporaceae	Cellulosimicrobium	cellulans
Bacteria	Actinobacteria	Actinobacteria	Micrococcales	Brevibacteriaceae	Brevibacterium	linens
Bacteria	Proteobacteria	Gammaproteobacteria	Enterobacterales	Yersiniaceae	Ewingella	americana
Bacteria	Firmicutes	Bacilli	Lactobacillales	Lactobacillaceae	Limosilactobacillus	fermentum
Bacteria	Bacteroidetes	Bacteroidia	Bacteroidales	Bacteroidaceae	Bacteroides	fragilis
Bacteria	Firmicutes	Bacilli	Lactobacillales	Streptococcaceae	Streptococcus	pneumoniae
Bacteria	Bacteroidetes	Bacteroidia	Bacteroidales	Prevotellaceae	Paraprevotella	xylaniphila
Bacteria	Bacteroidetes	Flavobacteriia	Flavobacteriales	Weeksellaceae	Chryseobacterium	indoltheticum
Bacteria	Proteobacteria	Gammaproteobacteria	Enterobacterales	Enterobacteriaceae	Citrobacter	sp._RHBSTW-00053
Bacteria	Actinobacteria	Acidimicrobiia	Acidimicrobiales	Ilumatobacteraceae	Ilumatobacter	coccineus
Bacteria	Proteobacteria	Alphaproteobacteria	Rhizobiales	Aurantimonadaceae	Aureimonas	altamirensis
Bacteria	Actinobacteria	Actinobacteria	Propionibacteriales	Nocardioidaceae	Nocardioides	euryhalodurans
Bacteria	Actinobacteria	Actinobacteria	Corynebacteriales	Mycobacteriaceae	Mycolicibacterium	psychrotolerans
Bacteria	Proteobacteria	Alphaproteobacteria	Caulobacterales	Caulobacteraceae	Caulobacter	flavus
Bacteria	Proteobacteria	Alphaproteobacteria	Sphingomonadales	Sphingomonadaceae	Sphingomonas	koreensis
Bacteria	Actinobacteria	Actinobacteria	Micrococcales	Microbacteriaceae	Microbacterium	sp._HY82
Bacteria	Proteobacteria	Alphaproteobacteria	Rhizobiales	Bradyrhizobiaceae	Bradyrhizobium	paxllaeri

Bacteria	Proteobacteria	Betaproteobacteria	Neisseriales	Neisseriaceae	Neisseria	meningitidis
Bacteria	Proteobacteria	Alphaproteobacteria	Rhodospirillales	Acetobacteraceae	Roseomonas	mucosa
Bacteria	Proteobacteria	Alphaproteobacteria	Rhizobiales	Bradyrhizobiaceae	Bosea	sp._ANAM02
Bacteria	Firmicutes	Bacilli	Lactobacillales	Streptococcaceae	Streptococcus	gwangjuense
Bacteria	Actinobacteria	Actinobacteria	Micrococcales	Brevibacteriaceae	Brevibacterium	sp._Marine
Bacteria	Proteobacteria	Alphaproteobacteria	Sphingomonadales	Sphingomonadaceae	Sphingomonas	sp._LK11
Bacteria	Proteobacteria	Alphaproteobacteria	Rhodobacterales	Rhodobacteraceae	Paracoccus	mutanolyticus
Bacteria	Proteobacteria	Gammaproteobacteria	Pseudomonadales	Moraxellaceae	Acinetobacter	nosocomialis
Bacteria	Firmicutes	Erysipelotrichia	Erysipelotrichales	Erysipelotrichaceae	Amedibacterium	intestinale
Bacteria	Proteobacteria	Gammaproteobacteria	Pseudomonadales	Pseudomonadaceae	Pseudomonas	saudiphocaensis
Bacteria	Proteobacteria	Gammaproteobacteria	Pseudomonadales	Pseudomonadaceae	Oblitimonas	alkaliphila
Bacteria	Proteobacteria	Gammaproteobacteria	Pseudomonadales	Pseudomonadaceae	Pseudomonas	stutzeri
Bacteria	Actinobacteria	Actinobacteria	Micrococcales	Micrococcaceae	Pseudarthrobacter	phenanthrenivorans
Bacteria	Proteobacteria	Betaproteobacteria	Burkholderiales	Alcaligenaceae	Alcaligenes	faecalis
Bacteria	Proteobacteria	Gammaproteobacteria	Pasteurellales	Pasteurellaceae	Haemophilus	influenzae
Bacteria	Actinobacteria	Actinobacteria	Corynebacteriales	Mycobacteriaceae	Mycolicibacterium	rhodesiae
Bacteria	Bacteroidetes	Bacteroidia	Bacteroidales	Rikenellaceae	Alistipes	finegoldii
Bacteria	Proteobacteria	Gammaproteobacteria	Xanthomonadales	Xanthomonadaceae	Arenimonas	daejeonensis
Bacteria	Proteobacteria	Gammaproteobacteria	Enterobacterales	Morganellaceae	Providencia	stuartii
Bacteria	Proteobacteria	Betaproteobacteria	Burkholderiales	Alcaligenaceae	Oligella	urethralis
Bacteria	Proteobacteria	Gammaproteobacteria	Pseudomonadales	Pseudomonadaceae	Pseudomonas	litoralis
Bacteria	Actinobacteria	Actinobacteria	Corynebacteriales	Corynebacteriaceae	Corynebacterium	genitalium
Bacteria	Firmicutes	Bacilli	Bacillales	Staphylococcaceae	Staphylococcus	equorum
Bacteria	Actinobacteria	Actinobacteria	Corynebacteriales	Nocardiaceae	Rhodococcus	fascians
Bacteria	Proteobacteria	Alphaproteobacteria	Rhizobiales	Bradyrhizobiaceae	Tardiphaga	robiniae
Bacteria	Proteobacteria	Gammaproteobacteria	Chromatiales	Chromatiaceae	Rheinheimera	sp._LHK132
Bacteria	Firmicutes	Bacilli	Lactobacillales	Enterococcaceae	Tetragenococcus	halophilus
Bacteria	Firmicutes	Bacilli	Lactobacillales	Streptococcaceae	Streptococcus	thermophilus
Bacteria	Tenericutes	Mollicutes	Mycoplasmatales	Mycoplasmataceae	Mycoplasma	cynos
Bacteria	Proteobacteria	Gammaproteobacteria	Pseudomonadales	Moraxellaceae	Acinetobacter	piscicola
Bacteria	Firmicutes	Tissierellia	Tissierellales	Peptoniphilaceae	Anaerococcus	prevotii
Bacteria	Proteobacteria	Gammaproteobacteria	Enterobacterales	Yersiniaceae	Serratia	surfactantfaciens
Bacteria	Proteobacteria	Alphaproteobacteria	Sphingomonadales	Erythrobacteraceae	Altererythrobacter	sp._TH136
Bacteria	Bacteroidetes	Bacteroidia	Bacteroidales	Porphyromonadaceae	Porphyromonas	crevioricanis
Bacteria	Proteobacteria	Betaproteobacteria	Neisseriales	Neisseriaceae	Neisseria	elongata
Bacteria	Proteobacteria	Gammaproteobacteria	Oceanospirillales	Halomonadaceae	Halomonas	hydrothermalis
Bacteria	Firmicutes	Bacilli	Lactobacillales	Carnobacteriaceae	Carnobacterium	maltaromaticum
Bacteria	Actinobacteria	Actinobacteria	Micrococcales	Microbacteriaceae	Cnuibacter	physcomitrellae
Bacteria	Proteobacteria	Alphaproteobacteria	Sphingomonadales	Erythrobacteraceae	Qipengyuania	sediminis
Bacteria	Actinobacteria	Actinobacteria	Bifidobacteriales	Bifidobacteriaceae	Bifidobacterium	thermophilum
Bacteria	Proteobacteria	Betaproteobacteria	Burkholderiales	Oxalobacteraceae	Massilia	sp._NR_4-1
Bacteria	Planctomycetes	Planctomycetia	Isosphaerales	Isosphaeraceae	Tautonia	plasticadhaerens
Bacteria	Bacteroidetes	Cytophagia	Cytophagales	Amoebophilaceae	Candidatus_Cardinium	Cardinium_endosymbiont_of_Sogatella_furcifera
Bacteria	Actinobacteria	Actinobacteria	Micromonosporales	Micromonosporaceae	Actinoplanes	sp._OR16
Bacteria	Proteobacteria	Betaproteobacteria	Burkholderiales	Oxalobacteraceae	Massilia	lutea
Bacteria	Proteobacteria	Gammaproteobacteria	Pseudomonadales	Pseudomonadaceae	Pseudomonas	rhizosphaerae
Bacteria	Actinobacteria	Actinobacteria	Corynebacteriales	Corynebacteriaceae	Corynebacterium	aurimucosum
Bacteria	Actinobacteria	Actinobacteria	Propionibacteriales	Nocardioidaceae	Nocardioides	dokdonensis
Bacteria	Proteobacteria	Gammaproteobacteria	Enterobacterales	Erwiniaceae	Mixta	calida
Bacteria	Proteobacteria	Gammaproteobacteria	Enterobacterales	Enterobacteriaceae	Raoultella	ornithinolytica
Bacteria	Bacteroidetes	Flavobacteriia	Flavobacteriales	Flavobacteriaceae	Flavobacterium	sp._HYN0086
Bacteria	Actinobacteria	Actinobacteria	Micrococcales	Dermabacteraceae	Brachybacterium	sp._SGAir0954

Bacteria	Actinobacteria	Actinobacteria	Corynebacteriales	Nocardiaceae	Rhodococcus	erythropolis
Bacteria	Proteobacteria	Gammaproteobacteria	Xanthomonadales	Xanthomonadaceae	Lysobacter	oculi
Bacteria	Proteobacteria	Gammaproteobacteria	Pseudomonadales	Moraxellaceae	Psychrobacter	sp._P11G5
Bacteria	Actinobacteria	Actinobacteria	Corynebacteriales	Corynebacteriaceae	Corynebacterium	resistens
Bacteria	Actinobacteria	Actinobacteria	Micrococcales	Microbacteriaceae	Frigoribacterium	sp._NBH87
Bacteria	Actinobacteria	Actinobacteria	Corynebacteriales	Corynebacteriaceae	Corynebacterium	striatum
Bacteria	Proteobacteria	Betaproteobacteria	Burkholderiales	Burkholderiaceae	Burkholderia	stabilis
Bacteria	Deinococcus-Thermus	Deinococci	Deinococcales	Deinococcaceae	Deinococcus	ficus
Bacteria	Proteobacteria	Gammaproteobacteria	Enterobacterales	Enterobacteriaceae	Leclercia	sp._29361
Bacteria	Firmicutes	Bacilli	Bacillales	Staphylococcaceae	Staphylococcus	xylosus
Bacteria	Actinobacteria	Actinobacteria	Micrococcales	Cellulomonadaceae	Cellulomonas	fimi
Bacteria	Proteobacteria	Gammaproteobacteria	Enterobacterales	Morganellaceae	Proteus	mirabilis
Bacteria	Actinobacteria	Actinobacteria	Corynebacteriales	Mycobacteriaceae	Mycolicibacterium	doricum
Bacteria	Actinobacteria	Actinobacteria	Corynebacteriales	Corynebacteriaceae	Corynebacterium	ammoniagenes
Bacteria	Firmicutes	Bacilli	Bacillales	Staphylococcaceae	Staphylococcus	pasteuri
Bacteria	Bacteroidetes	Flavobacteriia	Flavobacteriales	Weeksellaceae	Elizabethkingia	miricola
Bacteria	Firmicutes	Clostridia	Clostridiales	Hungateiclostridiaceae	Fastidiosipila	sanguinis
Bacteria	Proteobacteria	Betaproteobacteria	Burkholderiales	Oxalobacteraceae	Massilia	oculi
Bacteria	Bacteroidetes	Bacteroidia	Bacteroidales	Bacteroidaceae	Bacteroides	ovatus
Bacteria	Proteobacteria	Betaproteobacteria	Burkholderiales	Comamonadaceae	Acidovorax	sp._JMULE5
Bacteria	Proteobacteria	Betaproteobacteria	Rhodocyclales	Rhodocyclaceae	Aromatoleum	aromaticum
Bacteria	Firmicutes	Bacilli	Bacillales	Staphylococcaceae	Auricoccus	indicus
Bacteria	Actinobacteria	Actinobacteria	Propionibacteriales	Nocardioidaceae	Nocardioides	ungokensis
Bacteria	Proteobacteria	Betaproteobacteria	Burkholderiales	Alcaligenaceae	Achromobacter	spanius
Bacteria	Proteobacteria	Alphaproteobacteria	Sphingomonadales	Sphingomonadaceae	Sphingomonas	hengshuiensis
Bacteria	Actinobacteria	Actinobacteria	Micrococcales	Dermabacteraceae	Brachybacterium	avium
Bacteria	Actinobacteria	Actinobacteria	Bifidobacteriales	Bifidobacteriaceae	Gardnerella	vaginalis
Bacteria	Actinobacteria	Actinobacteria	Micrococcales	Microbacteriaceae	Microbacterium	sp._CBA3102
Bacteria	Firmicutes	Clostridia	Clostridiales	Lachnospiraceae	Enterocloster	bolteae
Bacteria	Bacteroidetes	Bacteroidia	Bacteroidales	Bacteroidaceae	Bacteroides	sp._A1C1
Bacteria	Firmicutes	Bacilli	Lactobacillales	Streptococcaceae	Lactococcus	lactis
Bacteria	Actinobacteria	Actinobacteria	Micrococcales	Intrasporangiaceae	Ornithinimicrobium	pratense
Bacteria	Firmicutes	Bacilli	Bacillales	Bacillaceae	Bacillus	anthracis
Bacteria	Proteobacteria	Gammaproteobacteria	Pseudomonadales	Pseudomonadaceae	Pseudomonas	parafulva
Bacteria	Proteobacteria	Gammaproteobacteria	Pseudomonadales	Pseudomonadaceae	Pseudomonas	libanensis
Bacteria	Actinobacteria	Actinobacteria	Corynebacteriales	Gordoniaceae	Gordonia	sp._JH63
Bacteria	Firmicutes	Bacilli	Lactobacillales	Streptococcaceae	Streptococcus	sobrinus
Bacteria	Actinobacteria	Actinobacteria	Sporichthyales	Sporichthyaceae	Epidermidibacterium	keratini
Bacteria	Firmicutes	Bacilli	Bacillales	Staphylococcaceae	Staphylococcus	pseudintermedius
Bacteria	Proteobacteria	Deltaproteobacteria	Myxococcales	Archangiaceae	Cystobacter	fuscus
Bacteria	Actinobacteria	Actinobacteria	Propionibacteriales	Nocardioidaceae	Nocardioides	sp._JS614
Bacteria	Firmicutes	Bacilli	Lactobacillales	Lactobacillaceae	Lactobacillus	helveticus
Bacteria	Proteobacteria	Betaproteobacteria	Neisseriales	Neisseriaceae	Neisseria	sp._oral_taxon_014
Bacteria	Bacteroidetes	Cytophagia	Cytophagales	Hymenobacteraceae	Hymenobacter	sp._BRD128
Bacteria	Proteobacteria	Betaproteobacteria	Burkholderiales	Oxalobacteraceae	Massilia	sp._YMA4
Bacteria	Spirochaetes	Spirochaetia	Spirochaetales	Spirochaetaceae	Treponema	succinifaciens
Bacteria	Proteobacteria	Alphaproteobacteria	Rhizobiales	Methylobacteriaceae	Methylobacterium	sp._4-46
Bacteria	Bacteroidetes	Flavobacteriia	Flavobacteriales	Flavobacteriaceae	Flavobacterium	crocinum
Bacteria	Proteobacteria	Alphaproteobacteria	Sphingomonadales	Sphingomonadaceae	Sphingomonas	insulae
Bacteria	Actinobacteria	Actinobacteria	Micrococcales	Micrococcaceae	Kocuria	rosea
Bacteria	Firmicutes	Bacilli	Bacillales	Bacillaceae	Bacillus	velezensis
Bacteria	Actinobacteria	Actinobacteria	Propionibacteriales	Nocardioidaceae	Nocardioides	sp._zg-579

Bacteria	Actinobacteria	Actinobacteria	Propionibacteriales	Nocardioidaceae	Mumia	sp._ZJ1417
Bacteria	Proteobacteria	Alphaproteobacteria	Rhizobiales	Brucellaceae	Brucella	intermedia
Bacteria	Proteobacteria	Alphaproteobacteria	Rhizobiales	Methylobacteriaceae	Methylobacterium	phyllosphaerae
Bacteria	Cyanobacteria		Nostocales	Nostocaceae	Cylindrospermum	stagnale
Bacteria	Firmicutes	Clostridia	Clostridiales	Lachnospiraceae	Mediterraneibacter	Ruminococcus_gnavus
Bacteria	Proteobacteria	Alphaproteobacteria	Rhodospirillales	Rhodospirillaceae	Skermanella	pratensis
Bacteria	Actinobacteria	Actinobacteria	Micrococcales	Microbacteriaceae	Rathayibacter	sp._VKM_Ac-2805
Bacteria	Proteobacteria	Gammaproteobacteria	Pseudomonadales	Moraxellaceae	Acinetobacter	pittii
Bacteria	Actinobacteria	Actinobacteria	Micrococcales	Microbacteriaceae	Curtobacterium	sp._SGAir0471
Bacteria	Proteobacteria	Betaproteobacteria	Burkholderiales	Oxalobacteraceae	Massilia	flava
Bacteria	Actinobacteria	Actinobacteria	Micrococcales	Microbacteriaceae	Agromyces	aureus
Bacteria	Actinobacteria	Actinobacteria	Propionibacteriales	Nocardioidaceae	Nocardioides	sp._78
Bacteria	Actinobacteria	Actinobacteria	Micrococcales	Microbacteriaceae	Microbacterium	sp._PAMC_28756
Bacteria	Actinobacteria	Actinobacteria	Micromonosporales	Micromonosporaceae	Actinoplanes	teichomyceticus
Bacteria	Proteobacteria	Betaproteobacteria	Burkholderiales	Oxalobacteraceae	Massilia	sp._LPB0304
Bacteria	Actinobacteria	Actinobacteria	Corynebacteriales	Corynebacteriaceae	Corynebacterium	ulcerans
Bacteria	Proteobacteria	Gammaproteobacteria	Xanthomonadales	Xanthomonadaceae	Stenotrophomonas	sp._364
Bacteria	Proteobacteria	Gammaproteobacteria	Enterobacterales	Morganellaceae	Proteus	vulgaris
Bacteria	Cyanobacteria		Nostocales	Nostocaceae	Nostoc	sp._NIES-2111
Bacteria	Proteobacteria	Betaproteobacteria	Burkholderiales	Burkholderiaceae	Burkholderia	pseudomallei
Bacteria	Actinobacteria	Actinobacteria	Corynebacteriales	Nocardiaceae	Rhodococcus	coprophilus
Bacteria	Firmicutes	Bacilli	Lactobacillales	Carnobacteriaceae	Carnobacterium	sp._CP1
Bacteria	Proteobacteria	Betaproteobacteria	Burkholderiales	Comamonadaceae	Rhodoferax	koreense
Bacteria	Proteobacteria	Alphaproteobacteria	Rhizobiales	Rhizobiaceae	Rhizobium	sp._TCK
Bacteria	Actinobacteria	Actinobacteria	Micrococcales	Microbacteriaceae	Microbacterium	oryzae
Bacteria	Actinobacteria	Actinobacteria	Micromonosporales	Micromonosporaceae	Micromonospora	echinofusca
Bacteria	Proteobacteria	Betaproteobacteria	Burkholderiales	Comamonadaceae	Comamonas	koreensis
Bacteria	Proteobacteria	Betaproteobacteria	Neisseriales	Neisseriaceae	Kingella	oralis
Bacteria	Bacteroidetes	Sphingobacteriia	Sphingobacteriales	Sphingobacteriaceae	Sphingobacterium	sp._G1-14
Bacteria	Proteobacteria	Alphaproteobacteria	Sphingomonadales	Sphingomonadaceae	Sphingomonas	ginsengisoli_An_et_al._2013
Bacteria	Firmicutes	Bacilli	Lactobacillales	Carnobacteriaceae	Dolosigranulum	pigrum
Bacteria	Proteobacteria	Alphaproteobacteria	Rhizobiales	Methylobacteriaceae	Methylobacterium	sp._WL1
Bacteria	Firmicutes	Bacilli	Lactobacillales	Leuconostocaceae	Weissella	confusa
Bacteria	Firmicutes	Bacilli	Lactobacillales	Carnobacteriaceae	Jeotgalibaca	arthritidis
Bacteria	Actinobacteria	Actinobacteria	Pseudonocardiales	Pseudonocardiaceae	Pseudonocardia	sp._Gen01
Bacteria	Proteobacteria	Alphaproteobacteria	Sphingomonadales	Sphingomonadaceae	Sphingomonas	panacis
Bacteria	Proteobacteria	Gammaproteobacteria	Pseudomonadales	Moraxellaceae	Acinetobacter	sp._YH12138
Bacteria	Actinobacteria	Actinobacteria	Micrococcales	Cellulomonadaceae	Cellulomonas	sp._PSBB021
Bacteria	Proteobacteria	Gammaproteobacteria	Xanthomonadales	Xanthomonadaceae	Lysobacter	sp._TY2-98
Bacteria	Actinobacteria	Actinobacteria	Micrococcales	Microbacteriaceae	Plantibacter	sp._M259
Bacteria	Bacteroidetes	Cytophagia	Cytophagales	Hymenobacteraceae	Hymenobacter	sp._BRD67
Bacteria	Proteobacteria	Gammaproteobacteria	Enterobacterales	Erwiniaceae	Pantoea	agglomerans
Bacteria	Actinobacteria	Actinobacteria	Propionibacteriales	Propionibacteriaceae	Propioniciclava	sp._HDW11
Bacteria	Firmicutes	Bacilli	Lactobacillales	Streptococcaceae	Streptococcus	pseudopneumoniae
Bacteria	Proteobacteria	Betaproteobacteria	Burkholderiales	Oxalobacteraceae	Janthinobacterium	svalbardensis
Bacteria	Proteobacteria	Betaproteobacteria	Burkholderiales	Comamonadaceae	Variovorax	sp._PMC12
Bacteria	Actinobacteria	Actinobacteria	Micrococcales	Brevibacteriaceae	Brevibacterium	aurantiacum
Bacteria	Firmicutes	Bacilli	Lactobacillales	Streptococcaceae	Streptococcus	oralis
Bacteria	Bacteroidetes	Cytophagia	Cytophagales	Hymenobacteraceae	Hymenobacter	swuensis
Bacteria	Firmicutes	Bacilli	Bacillales	Bacillaceae	Exiguobacterium	sp._N4-1P
Bacteria	Proteobacteria	Alphaproteobacteria	Rhodospirillales	Acetobacteraceae	Roseomonas	sp._FDAARGOS_362
Bacteria	Bacteroidetes	Bacteroidia	Bacteroidales	Tannerellaceae	Tannerella	forsythia

Bacteria	Proteobacteria	Gammaproteobacteria	Pseudomonadales	Moraxellaceae	Acinetobacter	baumannii
Bacteria	Firmicutes	Bacilli	Bacillales	Staphylococcaceae	Staphylococcus	haemolyticus
Bacteria	Proteobacteria	Betaproteobacteria	Burkholderiales	Alcaligenaceae	Bordetella	holmesii
Bacteria	Proteobacteria	Gammaproteobacteria	Pseudomonadales	Pseudomonadaceae	Pseudomonas	sp._B10
Bacteria	Proteobacteria	Betaproteobacteria	Burkholderiales	Comamonadaceae	Acidovorax	carolinensis
Bacteria	Proteobacteria	Alphaproteobacteria	Sphingomonadales	Sphingomonadaceae	Sphingomonas	sp._FARSPH
Bacteria	Proteobacteria	Alphaproteobacteria	Rhizobiales	Hyphomicrobiaceae	Devosia	sp._1566
Bacteria	Proteobacteria	Betaproteobacteria	Burkholderiales	Burkholderiaceae	Cupriavidus	oxalaticus
Bacteria	Firmicutes	Bacilli	Lactobacillales	Streptococcaceae	Lactococcus	garvieae
Bacteria	Bacteroidetes	Sphingobacteriia	Sphingobacteriales	Sphingobacteriaceae	Sphingobacterium	hotanense
Bacteria	Actinobacteria	Actinobacteria	Bifidobacteriales	Bifidobacteriaceae	Bifidobacterium	bifidum
Bacteria	Actinobacteria	Actinobacteria	Micrococcales	Jonesiaceae	Jonesia	denitrificans
Bacteria	Actinobacteria	Actinobacteria	Corynebacteriales	Corynebacteriaceae	Corynebacterium	matruchotii
Bacteria	Proteobacteria	Gammaproteobacteria	Pseudomonadales	Moraxellaceae	Acinetobacter	guillouiae
Archaea	Euryarchaeota	Methanobacteria	Methanobacteriales	Methanobacteriaceae	Methanobrevibacter	smithii
Bacteria	Proteobacteria	Alphaproteobacteria	Rhizobiales	Rhizobiaceae	Sinorhizobium	sp._RAC02
Bacteria	Proteobacteria	Betaproteobacteria	Neisseriales	Neisseriaceae	Conchiformibius	steedae
Bacteria	Proteobacteria	Alphaproteobacteria	Rhizobiales	Methylobacteriaceae	Methylobacterium	sp._17Sr1-1
Bacteria	Proteobacteria	Betaproteobacteria	Burkholderiales	Comamonadaceae	Hydrogenophaga	sp._RAC07
Bacteria	Proteobacteria	Betaproteobacteria	Burkholderiales	Comamonadaceae	Mitsuaria	sp._7
Bacteria	Proteobacteria	Betaproteobacteria	Burkholderiales	Comamonadaceae	Comamonas	thiooxydans
Bacteria	Bacteroidetes	Bacteroidia	Bacteroidales	Bacteroidaceae	Bacteroides	thetaiotaomicron
Bacteria	Bacteroidetes	Cytophagia	Cytophagales	Hymenobacteraceae	Pontibacter	russatus
Bacteria	Actinobacteria	Actinobacteria	Micrococcales	Microbacteriaceae	Microbacterium	aurum
Bacteria	Proteobacteria	Gammaproteobacteria	Xanthomonadales	Xanthomonadaceae	Thermomonas	sp._XSG
Bacteria	Proteobacteria	Gammaproteobacteria	Pseudomonadales	Moraxellaceae	Acinetobacter	venetianus
Bacteria	Actinobacteria	Actinobacteria	Propionibacteriales	Nocardioidaceae	Nocardioides	sp._S-1144
Bacteria	Firmicutes	Bacilli	Lactobacillales	Aerococcaceae	Aerococcus	viridans
Bacteria	Proteobacteria	Gammaproteobacteria	Pseudomonadales	Pseudomonadaceae	Pseudomonas	xanthomarina
Bacteria	Firmicutes	Bacilli	Bacillales	Staphylococcaceae	Staphylococcus	nepalensis
Bacteria	Actinobacteria	Actinobacteria	Corynebacteriales	Mycobacteriaceae	Mycolicibacterium	litorale
Bacteria	Actinobacteria	Actinobacteria	Streptosporangiales	Nocardiopsaceae	Nocardiopsis	dassonvillei
Bacteria	Actinobacteria	Actinobacteria	Micrococcales	Microbacteriaceae	Microbacterium	sp._ABRD_28
Bacteria	Proteobacteria	Gammaproteobacteria	Pseudomonadales	Pseudomonadaceae	Pseudomonas	psychrophila
Bacteria	Proteobacteria	Alphaproteobacteria	Rhodospirillales	Acetobacteraceae	Roseomonas	gilardii
Bacteria	Actinobacteria	Actinobacteria	Micrococcales	Dermabacteraceae	Brachybacterium	vulturis
Bacteria	Firmicutes	Bacilli	Bacillales	Staphylococcaceae	Staphylococcus	saprophyticus
Bacteria	Proteobacteria	Gammaproteobacteria	Enterobacterales	Enterobacteriaceae	Enterobacter	sp._SA187
Bacteria	Firmicutes	Clostridia	Clostridiales	Lachnospiraceae	Roseburia	hominis
Bacteria	Actinobacteria	Actinobacteria	Corynebacteriales	Dietziaceae	Dietzia	lutea
Bacteria	Proteobacteria	Betaproteobacteria	Burkholderiales	Oxalobacteraceae	Massilia	umbonata
Bacteria	Firmicutes	Bacilli	Bacillales	Staphylococcaceae	Macrococcus	canis
Bacteria	Proteobacteria	Alphaproteobacteria	Rhizobiales	Hyphomicrobiaceae	Rhodoplanes	sp._Z2-YC6860
Bacteria	Proteobacteria	Gammaproteobacteria	Pseudomonadales	Moraxellaceae	Acinetobacter	indicus
Bacteria	Actinobacteria	Actinobacteria	Pseudonocardiales	Pseudonocardiaceae	Amycolatopsis	mediterranei
Bacteria	Proteobacteria	Alphaproteobacteria	Rhizobiales	Rhizobiaceae	Rhizobium	oryzihabitans
Bacteria	Firmicutes	Clostridia	Clostridiales	Lachnospiraceae	Blautia	producta
Bacteria	Proteobacteria	Alphaproteobacteria	Caulobacterales	Caulobacteraceae	Brevundimonas	vancanneytii
Bacteria	Actinobacteria	Actinobacteria	Corynebacteriales	Corynebacteriaceae	Corynebacterium	humireducens
Bacteria	Actinobacteria	Actinobacteria	Micrococcales	Cellulomonadaceae	Cellulomonas	gilvus
Bacteria	Proteobacteria	Alphaproteobacteria	Rhodobacterales	Rhodobacteraceae	Pseudooceanicola	algae
Bacteria	Proteobacteria	Gammaproteobacteria	Pseudomonadales	Pseudomonadaceae	Pseudomonas	sp._Lz4W

Bacteria	Actinobacteria	Acidimicrobiia	Acidimicrobiales	Iamiaceae	Actinomarinicola	tropica
Bacteria	Actinobacteria	Actinobacteria	Corynebacteriales	Corynebacteriaceae	Corynebacterium	singulare
Bacteria	Proteobacteria	Gammaproteobacteria	Pseudomonadales	Moraxellaceae	Acinetobacter	gyllenbergii
Bacteria	Actinobacteria	Actinobacteria	Corynebacteriales	Nocardiaceae	Rhodococcus	ruber
Bacteria	Actinobacteria	Actinobacteria	Bifidobacteriales	Bifidobacteriaceae	Bifidobacterium	dentium
Bacteria	Actinobacteria	Actinobacteria	Micrococcales	Microbacteriaceae	Rathayibacter	sp._VKM_Ac-2760
Bacteria	Actinobacteria	Actinobacteria	Micrococcales	Brevibacteriaceae	Brevibacterium	sp._CS2
Bacteria	Actinobacteria	Actinobacteria	Corynebacteriales	Mycobacteriaceae	Mycolicibacterium	gilvum
Bacteria	Bacteroidetes	Bacteroidia	Bacteroidales	Prevotellaceae	Prevotella	oris
Bacteria	Proteobacteria	Alphaproteobacteria	Rhodobacterales	Rhodobacteraceae	Haematobacter	massiliensis
Bacteria	Proteobacteria	Betaproteobacteria	Burkholderiales	Comamonadaceae	Ramlibacter	tataouinensis
Bacteria	Firmicutes	Bacilli	Lactobacillales	Streptococcaceae	Streptococcus	sp._oral_taxon_431
Bacteria	Firmicutes	Bacilli	Lactobacillales	Enterococcaceae	Enterococcus	mundtii
Bacteria	Proteobacteria	Betaproteobacteria	Burkholderiales	Comamonadaceae	Variovorax	paradoxus
Bacteria	Actinobacteria	Actinobacteria	Propionibacteriales	Nocardioidaceae	Nocardioides	sambongensis
Bacteria	Proteobacteria	Gammaproteobacteria	Xanthomonadales	Rhodanobacteraceae	Luteibacter	rhizovicinus
Bacteria	Proteobacteria	Gammaproteobacteria	Enterobacterales	Morganellaceae	Providencia	rettgeri
Bacteria	Bacteroidetes	Bacteroidia	Bacteroidales	Rikenellaceae	Alistipes	communis
Bacteria	Proteobacteria	Gammaproteobacteria	Enterobacterales	Enterobacteriaceae	Enterobacter	roggenkampii
Bacteria	Actinobacteria	Actinobacteria	Micrococcales	Micrococcaceae	Kocuria	palustris
Bacteria	Bacteroidetes	Flavobacteriia	Flavobacteriales	Flavobacteriaceae	Salegentibacter	sp._T436
Bacteria	Bacteroidetes	Flavobacteriia	Flavobacteriales	Weeksellaceae	Chryseobacterium	sp._cx-624
Bacteria	Proteobacteria	Alphaproteobacteria	Rickettsiales	Rickettsiaceae	Rickettsia	bellii
Bacteria	Actinobacteria	Actinobacteria	Micromonosporales	Micromonosporaceae	Micromonospora	siamensis
Bacteria	Firmicutes	Clostridia	Clostridiales	Lachnospiraceae	Enterocloster	clostridioformis
Bacteria	Bacteroidetes	Flavobacteriia	Flavobacteriales	Weeksellaceae	Chryseobacterium	carnipullorum
Bacteria	Bacteroidetes	Sphingobacteriia	Sphingobacteriales	Sphingobacteriaceae	Sphingobacterium	multivorum
Bacteria	Deinococcus-Thermus	Deinococci	Deinococcales	Deinococcaceae	Deinococcus	sp._D7000
Bacteria	Actinobacteria	Actinobacteria	Micrococcales	Brevibacteriaceae	Brevibacterium	sandarakinum
Bacteria	Actinobacteria	Actinobacteria	Corynebacteriales	Corynebacteriaceae	Corynebacterium	camporealensis
Bacteria	Proteobacteria	Alphaproteobacteria	Sphingomonadales	Sphingomonadaceae	Sphingomonas	indica
Bacteria	Proteobacteria	Alphaproteobacteria	Rhodobacterales	Rhodobacteraceae	Paracoccus	pantotrophus
Bacteria	Proteobacteria	Gammaproteobacteria	Pseudomonadales	Pseudomonadaceae	Pseudomonas	fragi
Bacteria	Proteobacteria	Gammaproteobacteria	Enterobacterales	Enterobacteriaceae	Citrobacter	rodentium
Bacteria	Actinobacteria	Actinobacteria	Bifidobacteriales	Bifidobacteriaceae	Bifidobacterium	pseudolongum
Bacteria	Bacteroidetes	Cytophagia	Cytophagales	Hymenobacteraceae	Hymenobacter	sp._NBH84
Bacteria	Firmicutes	Bacilli	Lactobacillales	Streptococcaceae	Streptococcus	sp._oral_taxon_064
Bacteria	Actinobacteria	Actinobacteria	Propionibacteriales	Nocardioidaceae	Nocardioides	sp._ZJ1313
Bacteria	Proteobacteria	Alphaproteobacteria	Caulobacterales	Caulobacteraceae	Brevundimonas	sp._GW460-12-10-14-LB2
Bacteria	Proteobacteria	Alphaproteobacteria	Rhizobiales	Bradyrhizobiaceae	Bosea	sp._F3-2
Bacteria	Actinobacteria	Actinobacteria	Micrococcales	Promicromonosporaceae	Cellulosimicrobium	sp._JZ28
Bacteria	Actinobacteria	Actinobacteria	Micromonosporales	Micromonosporaceae	Actinoplanes	missouriensis
Bacteria	Proteobacteria	Gammaproteobacteria	Pseudomonadales	Moraxellaceae	Acinetobacter	sp._MYb10
Bacteria	Proteobacteria	Alphaproteobacteria	Caulobacterales	Caulobacteraceae	Brevundimonas	diminuta
Bacteria	Actinobacteria	Actinobacteria	Propionibacteriales	Propionibacteriaceae	Cutibacterium	acnes
Bacteria	Proteobacteria	Gammaproteobacteria	Pseudomonadales	Pseudomonadaceae	Pseudomonas	alcaliphila
Bacteria	Proteobacteria	Alphaproteobacteria	Rhodobacterales	Rhodobacteraceae	Paracoccus	liaowanqingii
Bacteria	Proteobacteria	Deltaproteobacteria	Myxococcales	Myxococcaceae	Myxococcus	xanthus
Bacteria	Bacteroidetes	Bacteroidia	Bacteroidales	Bacteroidaceae	Bacteroides	sp._CACC_737
Bacteria	Proteobacteria	Deltaproteobacteria	Desulfovibrionales	Desulfovibrionaceae	Desulfovibrio	fairfieldensis
Bacteria	Proteobacteria	Alphaproteobacteria	Sphingomonadales	Sphingomonadaceae	Sphingomonas	sp._NIC1
Bacteria	Actinobacteria	Actinobacteria	Propionibacteriales	Nocardioidaceae	Aeromicrobium	sp._MF47

Bacteria	Actinobacteria	Actinobacteria	Corynebacteriales	Nocardiaceae	Rhodococcus	sp._PBTS_2
Bacteria	Firmicutes	Bacilli	Lactobacillales	Streptococcaceae	Streptococcus	sanguinis
Bacteria	Actinobacteria	Actinobacteria	Micrococcales	Microbacteriaceae	Agrococcus	sp._SGAir0287
Bacteria	Firmicutes	Bacilli	Lactobacillales	Lactobacillaceae	Lactobacillus	gasseri
Bacteria	Proteobacteria	Gammaproteobacteria	Enterobacterales	Enterobacteriaceae	Escherichia	coli
Bacteria	Actinobacteria	Actinobacteria	Micrococcales	Microbacteriaceae	Microbacterium	sp._TPU_3598
Bacteria	Actinobacteria	Coriobacteriia	Coriobacteriales	Atopobiaceae	Libanicoccus	massiliensis
Bacteria	Firmicutes	Bacilli	Lactobacillales	Lactobacillaceae	Lacticaseibacillus	paracasei
Bacteria	Proteobacteria	Gammaproteobacteria	Enterobacterales	Enterobacteriaceae	Klebsiella	michiganensis
Bacteria	Actinobacteria	Actinobacteria	Corynebacteriales	Corynebacteriaceae	Corynebacterium	flavescens
Bacteria	Bacteroidetes	Bacteroidia	Bacteroidales		Phocaeicola	salanitronis
Bacteria	Actinobacteria	Actinobacteria	Corynebacteriales	Corynebacteriaceae	Corynebacterium	sp._NML98-0116
Bacteria	Actinobacteria	Actinobacteria	Micrococcales	Microbacteriaceae	Rathayibacter	toxicus
Bacteria	Firmicutes	Negativicutes	Veillonellales	Veillonellaceae	Veillonella	dispar
Bacteria	Proteobacteria	Alphaproteobacteria	Caulobacterales	Caulobacteraceae	Phenylobacterium	zucineum
Bacteria	Deinococcus-Thermus	Deinococci	Deinococcales	Deinococcaceae	Deinococcus	wulumuqiensis
Bacteria	Actinobacteria	Actinobacteria	Pseudonocardiales	Pseudonocardiaceae	Pseudonocardia	sp._HH130629-09
Bacteria	Proteobacteria	Gammaproteobacteria	Enterobacterales	Enterobacteriaceae	Atlantibacter	hermannii
Bacteria	Proteobacteria	Gammaproteobacteria	Pseudomonadales	Moraxellaceae	Acinetobacter	junii
Bacteria	Proteobacteria	Gammaproteobacteria	Pseudomonadales	Pseudomonadaceae	Pseudomonas	umsongensis
Bacteria	Proteobacteria	Alphaproteobacteria	Sphingomonadales	Sphingomonadaceae	Sphingomonas	paucimobilis
Bacteria	Actinobacteria	Actinobacteria	Propionibacteriales	Nocardioidaceae	Nocardioides	humi
Bacteria	Proteobacteria	Alphaproteobacteria	Sphingomonadales	Sphingomonadaceae	Sphingomonas	sp._AAP5
Bacteria	Proteobacteria	Deltaproteobacteria	Myxococcales	Archangiaceae	Archangium	gephyra
Bacteria	Actinobacteria	Actinobacteria	Corynebacteriales	Mycobacteriaceae	Mycolicibacterium	arabiense
Bacteria	Actinobacteria	Actinobacteria	Pseudonocardiales	Pseudonocardiaceae	Saccharopolyspora	coralli
Bacteria	Firmicutes	Bacilli	Bacillales	Staphylococcaceae	Staphylococcus	vitulinus
Bacteria	Bacteroidetes	Sphingobacteriia	Sphingobacteriales	Sphingobacteriaceae	Sphingobacterium	sp._B29
Bacteria	Bacteroidetes	Flavobacteriia	Flavobacteriales	Weeksellaceae	Planobacterium	Chryseobacterium_taklimakanense
Bacteria	Firmicutes	Bacilli	Lactobacillales	Lactobacillaceae	Lactobacillus	jensenii
Bacteria	Proteobacteria	Gammaproteobacteria	Enterobacterales	Erwiniaceae	Buchnera	aphidicola
Bacteria	Bacteroidetes	Bacteroidia	Bacteroidales	Bacteroidaceae	Bacteroides	sp._CBA7301
Bacteria	Firmicutes	Bacilli	Bacillales	Staphylococcaceae	Staphylococcus	simulans
Bacteria	Proteobacteria	Gammaproteobacteria	Pseudomonadales	Pseudomonadaceae	Pseudomonas	aeruginosa
Bacteria	Actinobacteria	Actinobacteria	Micrococcales	Intrasporangiaceae	Tetrasphaera	sp._HKS02
Bacteria	Actinobacteria	Actinobacteria	Corynebacteriales	Corynebacteriaceae	Corynebacterium	sp._Marseille-Q3630
Bacteria	Actinobacteria	Actinobacteria	Micrococcales	Micrococcaceae	Kocuria	flava
Bacteria	Actinobacteria	Actinobacteria	Propionibacteriales	Nocardioidaceae	Nocardioides	sp._MC1495
Bacteria	Bacteroidetes	Bacteroidia	Bacteroidales	Bacteroidaceae	Bacteroides	intestinalis
Bacteria	Bacteroidetes	Bacteroidia	Bacteroidales	Bacteroidaceae	Bacteroides	coprosuis
Bacteria	Proteobacteria	Gammaproteobacteria	Xanthomonadales	Xanthomonadaceae	Xanthomonas	translucens
Bacteria	Proteobacteria	Gammaproteobacteria	Pasteurellales	Pasteurellaceae	Haemophilus	parainfluenzae
Bacteria	Actinobacteria	Actinobacteria	Micromonosporales	Micromonosporaceae	Micromonospora	sagamiensis
Bacteria	Bacteroidetes	Bacteroidia	Bacteroidales	Rikenellaceae	Alistipes	dispar
Bacteria	Actinobacteria	Actinobacteria	Bifidobacteriales	Bifidobacteriaceae	Bifidobacterium	pullorum
Bacteria	Actinobacteria	Actinobacteria	Micrococcales	Microbacteriaceae	Curtobacterium	pusillum
Bacteria	Actinobacteria	Actinobacteria	Pseudonocardiales	Pseudonocardiaceae	Pseudonocardia	sp._AL041005-10
Bacteria	Proteobacteria	Gammaproteobacteria	Enterobacterales	Enterobacteriaceae	Leclercia	adecarboxylata
Bacteria	Actinobacteria	Actinobacteria	Pseudonocardiales	Pseudonocardiaceae	Pseudonocardia	autotrophica
Bacteria	Proteobacteria	Betaproteobacteria	Burkholderiales	Comamonadaceae	Acidovorax	sp._1608163
Bacteria	Proteobacteria	Alphaproteobacteria	Caulobacterales	Caulobacteraceae	Caulobacter	mirabilis
Bacteria	Proteobacteria	Betaproteobacteria	Burkholderiales	Comamonadaceae	Variovorax	sp._PAMC_28711

Bacteria	Proteobacteria	Gammaproteobacteria	Enterobacterales	Pectobacteriaceae	Pectobacterium	brasiliense
Bacteria	Bacteroidetes	Cytophagia	Cytophagales	Cytophagaceae	Spirosoma	pollinicola
Bacteria	Proteobacteria	Gammaproteobacteria	Pseudomonadales	Pseudomonadaceae	Pseudomonas	chlororaphis
Bacteria	Proteobacteria	Gammaproteobacteria	Pseudomonadales	Moraxellaceae	Acinetobacter	sp._TTH0-4
Bacteria	Bacteroidetes	Cytophagia	Cytophagales	Cytophagaceae	Spirosoma	rigui
Bacteria	Proteobacteria	Gammaproteobacteria	Enterobacterales	Enterobacteriaceae	Raoultella	Plautia_stali_symbiont
Bacteria	Proteobacteria	Betaproteobacteria	Burkholderiales	Alcaligenaceae	Xylophilus	rhododendri
Bacteria	Actinobacteria	Actinobacteria	Micrococcales	Brevibacteriaceae	Brevibacterium	siliguriense
Bacteria	Bacteroidetes	Flavobacteriia	Flavobacteriales	Weeksellaceae	Chryseobacterium	sp._3008163
Bacteria	Actinobacteria	Actinobacteria	Micrococcales	Cellulomonadaceae	Cellulomonas	sp._JZ18
Bacteria	Actinobacteria	Actinobacteria	Micrococcales	Intrasporangiaceae	Ornithinimicrobium	sp._AMA3305
Bacteria	Deinococcus-Thermus	Deinococci	Deinococcales	Deinococcaceae	Deinococcus	gobiensis
Bacteria	Bacteroidetes	Bacteroidia	Bacteroidales	Rikenellaceae	Alistipes	shahii
Bacteria	Firmicutes	Bacilli	Bacillales	Bacillaceae	Gemella	haemolysans
Bacteria	Bacteroidetes	Sphingobacteriia	Sphingobacteriales	Sphingobacteriaceae	Sphingobacterium	sp._CZ-2
Bacteria	Actinobacteria	Actinobacteria	Streptomycetales	Streptomycetaceae	Streptomyces	sp._SM17
Bacteria	Actinobacteria	Actinobacteria	Propionibacteriales	Propionibacteriaceae	Microlunatus	phosphovorus
Bacteria	Actinobacteria	Actinobacteria	Micrococcales	Microbacteriaceae	Curtobacterium	sp._BH-2-1-1
Bacteria	Actinobacteria	Actinobacteria	Micrococcales	Microbacteriaceae	Microbacterium	oleivorans
Bacteria	Firmicutes	Erysipelotrichia	Erysipelotrichales	Erysipelotrichaceae	Longibaculum	sp._KGMB06250
Bacteria	Actinobacteria	Actinobacteria	Micrococcales	Cellulomonadaceae	Cellulomonas	sp._Y8
Bacteria	Proteobacteria	Gammaproteobacteria	Enterobacterales	Yersiniaceae	Serratia	fonticola
Bacteria	Proteobacteria	Betaproteobacteria	Neisseriales	Neisseriaceae	Neisseria	sicca
Bacteria	Firmicutes	Bacilli	Lactobacillales	Lactobacillaceae	Limosilactobacillus	reuteri
Bacteria	Firmicutes	Bacilli	Lactobacillales	Aerococcaceae	Abiotrophia	defectiva
Bacteria	Proteobacteria	Alphaproteobacteria	Sphingomonadales	Sphingomonadaceae	Sphingobium	yanoikuyae
Bacteria	Proteobacteria	Betaproteobacteria	Rhodocyclales	Zoogloeaceae	Azoarcus	pumilus
Bacteria	Firmicutes	Clostridia	Clostridiales	Ruminococcaceae	Ruthenibacterium	lactatiformans
Bacteria	Proteobacteria	Gammaproteobacteria	Enterobacterales	Pectobacteriaceae	Pectobacterium	carotovorum
Bacteria	Proteobacteria	Gammaproteobacteria	Pseudomonadales	Moraxellaceae	Psychrobacter	sp._P11F6
Bacteria	Proteobacteria	Alphaproteobacteria	Rhizobiales	Bradyrhizobiaceae	Bradyrhizobium	icense
Bacteria	Proteobacteria	Gammaproteobacteria	Xanthomonadales	Xanthomonadaceae	Xanthomonas	hortorum
Archaea	Thaumarchaeota	Nitrososphaeria	Nitrososphaerales	Nitrososphaeraceae	Candidatus_Nitrosocosmicus	oleophilus
Bacteria	Proteobacteria	Gammaproteobacteria	Xanthomonadales	Xanthomonadaceae	Pseudoxanthomonas	mexicana
Bacteria	Proteobacteria	Gammaproteobacteria	Pseudomonadales	Pseudomonadaceae	Pseudomonas	sp._NIBRBAC000502773
Bacteria	Proteobacteria	Gammaproteobacteria	Enterobacterales	Enterobacteriaceae	Escherichia	fergusonii
Bacteria	Actinobacteria	Actinobacteria	Micrococcales	Dermabacteraceae	Dermabacter	jinjuensis
Bacteria	Proteobacteria	Alphaproteobacteria	Rhizobiales	Methylobacteriaceae	Methylobacterium	sp._C1
Bacteria	Actinobacteria	Actinobacteria	Corynebacteriales	Nocardiaceae	Nocardia	asteroides
Bacteria	Firmicutes	Bacilli	Bacillales	Staphylococcaceae	Staphylococcus	pettenkoferi
Bacteria	Proteobacteria	Alphaproteobacteria	Rhodobacterales	Rhodobacteraceae	Paracoccus	zhejiangensis
Bacteria	Proteobacteria	Gammaproteobacteria	Enterobacterales	Enterobacteriaceae	Klebsiella	quasipneumoniae
Bacteria	Proteobacteria	Betaproteobacteria	Rhodocyclales	Zoogloeaceae	Thauera	sp._MZ1T
Bacteria	Proteobacteria	Epsilonproteobacteria	Campylobacterales	Campylobacteraceae	Arcobacter	skirrowii
Bacteria	Actinobacteria	Actinobacteria	Micrococcales	Cellulomonadaceae	Cellulomonas	sp._40-2
Bacteria	Actinobacteria	Actinobacteria	Micrococcales	Dermabacteraceae	Brachybacterium	ginsengisoli
Bacteria	Actinobacteria	Actinobacteria	Propionibacteriales	Propionibacteriaceae	Cutibacterium	granulosum
Bacteria	Proteobacteria	Alphaproteobacteria	Rickettsiales	Anaplasmataceae	Wolbachia	pipientis
Bacteria	Firmicutes	Bacilli	Lactobacillales	Lactobacillaceae	Lactobacillus	delbrueckii
Bacteria	Firmicutes	Negativicutes	Veillonellales	Veillonellaceae	Veillonella	atypica
Bacteria	Actinobacteria	Actinobacteria	Micrococcales	Intrasporangiaceae	Ornithinimicrobium	flavum
Bacteria	Cyanobacteria		Nostocales	Nostocaceae	Nostoc	flagelliforme

Bacteria	Actinobacteria	Actinobacteria	Micrococcales	Micrococcaceae	Arthrobacter	sp._NEB_688
Bacteria	Actinobacteria	Actinobacteria	Corynebacteriales	Corynebacteriaceae	Corynebacterium	lactis
Bacteria	Proteobacteria	Alphaproteobacteria	Rhizobiales	Rhizobiaceae	Ensifer	adhaerens
Bacteria	Actinobacteria	Actinobacteria	Micrococcales	Microbacteriaceae	Leucobacter	triazinivorans
Bacteria	Proteobacteria	Alphaproteobacteria	Rhizobiales	Brucellaceae	Ochrobactrum	pituitosum
Bacteria	Actinobacteria	Actinobacteria	Propionibacteriales	Nocardioidaceae	Pimelobacter	simplex
Bacteria	Proteobacteria	Alphaproteobacteria	Sphingomonadales	Sphingomonadaceae	Sphingomonas	sp._CL5.1
Bacteria	Bacteroidetes	Bacteroidia	Bacteroidales	Porphyromonadaceae	Porphyromonas	cangingivalis
Bacteria	Actinobacteria	Actinobacteria	Propionibacteriales	Nocardioidaceae	Marmoricola	scoriae
Bacteria	Proteobacteria	Alphaproteobacteria	Rhodobacterales	Rhodobacteraceae	Phaeobacter	gallaeciensis
Bacteria	Proteobacteria	Alphaproteobacteria	Sphingomonadales	Sphingomonadaceae	Sphingomonas	sp._XS-10
Bacteria	Proteobacteria	Betaproteobacteria	Burkholderiales	Comamonadaceae	Serpentinomonas	raichei
Bacteria	Actinobacteria	Actinobacteria	Corynebacteriales	Corynebacteriaceae	Corynebacterium	xerosis
Bacteria	Bacteroidetes	Sphingobacteriia	Sphingobacteriales	Sphingobacteriaceae	Pedobacter	sp._G11
Bacteria	Proteobacteria	Gammaproteobacteria	Enterobacterales	Erwiniaceae	Pantoea	stewartii
Bacteria	Proteobacteria	Alphaproteobacteria	Caulobacterales	Caulobacteraceae	Brevundimonas	sp._M20
Bacteria	Actinobacteria	Actinobacteria	Propionibacteriales	Nocardioidaceae	Nocardioides	daphniae
Bacteria	Actinobacteria	Actinobacteria	Propionibacteriales	Propionibacteriaceae	Tessaracoccus	defluvii
Bacteria	Bacteroidetes	Flavobacteriia	Flavobacteriales	Weeksellaceae	Empedobacter	stercoris
Bacteria	Actinobacteria	Actinobacteria	Micrococcales	Microbacteriaceae	Curtobacterium	sp._MR_MD2014
Bacteria	Actinobacteria	Actinobacteria	Micrococcales	Micrococcaceae	Rothia	nasimurium
Bacteria	Actinobacteria	Actinobacteria	Corynebacteriales	Nocardiaceae	Nocardia	brasiliensis
Bacteria	Proteobacteria	Gammaproteobacteria	Pseudomonadales	Pseudomonadaceae	Pseudomonas	sp._LG1E9
Bacteria	Actinobacteria	Actinobacteria	Propionibacteriales	Propionibacteriaceae	Propionibacterium	acidifaciens
Bacteria	Proteobacteria	Alphaproteobacteria	Sphingomonadales	Sphingomonadaceae	Sphingomonas	sp._AP4-R1
Bacteria	Bacteroidetes	Cytophagia	Cytophagales	Hymenobacteraceae	Adhaeribacter	swui
Bacteria	Firmicutes	Bacilli	Bacillales	Planococcaceae	Planococcus	rifietoensis
Bacteria	Firmicutes	Bacilli	Bacillales	Planococcaceae	Sporosarcina	ureae
Bacteria	Proteobacteria	Gammaproteobacteria	Xanthomonadales	Xanthomonadaceae	Xanthomonas	sp._SS
Bacteria	Actinobacteria	Actinobacteria	Corynebacteriales	Dietziaceae	Dietzia	sp._oral_taxon_368
Bacteria	Actinobacteria	Actinobacteria	Corynebacteriales	Corynebacteriaceae	Corynebacterium	diphtheriae
Bacteria	Actinobacteria	Actinobacteria	Corynebacteriales	Mycobacteriaceae	Mycolicibacterium	gadium
Bacteria	Proteobacteria	Gammaproteobacteria	Xanthomonadales	Xanthomonadaceae	Lysobacter	enzymogenes
Bacteria	Firmicutes	Bacilli	Lactobacillales	Streptococcaceae	Streptococcus	anginosus
Bacteria	Firmicutes	Bacilli	Lactobacillales	Carnobacteriaceae	Carnobacterium	inhibens
Bacteria	Proteobacteria	Gammaproteobacteria	Enterobacterales	Enterobacteriaceae	Enterobacter	sp._638
Bacteria	Firmicutes	Bacilli	Lactobacillales	Lactobacillaceae	Latilactobacillus	Lactobacillus_sakei
Bacteria	Proteobacteria	Gammaproteobacteria	Enterobacterales	Erwiniaceae	Pantoea	dispersa
Bacteria	Proteobacteria	Gammaproteobacteria	Cellvibrionales	Cellvibrionaceae	Cellvibrio	sp._PSBB023
Bacteria	Proteobacteria	Alphaproteobacteria	Rickettsiales	Rickettsiaceae	Rickettsia	felis
Bacteria	Actinobacteria	Coriobacteriia	Eggerthellales	Eggerthellaceae	Gordonibacter	urolithinfaciens
Bacteria	Cyanobacteria		Oscillatoriales	Microcoleaceae	Microcoleus	sp._PCC_7113
Bacteria	Proteobacteria	Gammaproteobacteria	Pseudomonadales	Moraxellaceae	Acinetobacter	johnsonii
Bacteria	Actinobacteria	Actinobacteria	Micrococcales	Microbacteriaceae	Microbacterium	chocolatum
Bacteria	Firmicutes	Clostridia	Clostridiales	Lachnospiraceae	Blautia	argi
Bacteria	Actinobacteria	Actinobacteria	Micrococcales	Micrococcaceae	Arthrobacter	agilis
Bacteria	Proteobacteria	Alphaproteobacteria	Sphingomonadales	Sphingomonadaceae	Sphingomonas	sp._Cra20
Bacteria	Proteobacteria	Alphaproteobacteria	Rhizobiales	Rhizobiaceae	Rhizobium	hidalgonense
Bacteria	Actinobacteria	Actinobacteria	Micrococcales	Ruaniaceae	Luteimicrobium	xylanilyticum
Bacteria	Proteobacteria	Alphaproteobacteria	Rhizobiales	Methylobacteriaceae	Methylorubrum	extorquens
Bacteria	Cyanobacteria		Oscillatoriales	Oscillatoriaceae	Oscillatoria	nigro-viridis
Bacteria	Actinobacteria	Rubrobacteria	Rubrobacterales	Rubrobacteraceae	Rubrobacter	sp._SCSIO_52909

Bacteria	Bacteroidetes	Cytophagia	Cytophagales	Hymenobacteraceae	Pontibacter	pudoricolor
Bacteria	Proteobacteria	Gammaproteobacteria	Pseudomonadales	Pseudomonadaceae	Pseudomonas	sp._NP-1
Bacteria	Acidobacteria	Acidobacteriia	Acidobacteriales	Acidobacteriaceae	Terriglobus	roseus
Bacteria	Proteobacteria	Alphaproteobacteria	Rhizobiales	Methylobacteriaceae	Methylorubrum	populi
Bacteria	Actinobacteria	Actinobacteria	Streptosporangiales	Streptosporangiaceae	Nonomuraea	sp._ATCC_55076
Bacteria	Bacteroidetes	Cytophagia	Cytophagales	Hymenobacteraceae	Hymenobacter	sp._APR13
Bacteria	Firmicutes	Bacilli	Bacillales	Staphylococcaceae	Staphylococcus	epidermidis
Bacteria	Proteobacteria	Deltaproteobacteria	Myxococcales	Polyangiaceae	Sorangium	cellulosum
Bacteria	Bacteroidetes	Flavobacteriia	Flavobacteriales	Flavobacteriaceae	Flavobacterium	psychrophilum
Bacteria	Cyanobacteria		Nostocales	Scytonemataceae	Scytonema	sp._HK-05
Bacteria	Proteobacteria	Gammaproteobacteria	Enterobacterales	Enterobacteriaceae	Klebsiella	aerogenes
Bacteria	Proteobacteria	Betaproteobacteria	Burkholderiales	Burkholderiaceae	Burkholderia	sp._THE68
Bacteria	Proteobacteria	Alphaproteobacteria	Sphingomonadales	Sphingomonadaceae	Sphingomonas	sp._LM7
Bacteria	Actinobacteria	Actinobacteria	Micrococcales	Micrococcaceae	Pseudarthrobacter	sp._BIM_B-2242
Bacteria	Actinobacteria	Actinobacteria	Micrococcales	Micrococcaceae	Kocuria	sp._TGY1120_3
Bacteria	Actinobacteria	Actinobacteria	Propionibacteriales	Nocardioidaceae	Nocardioides	sp._WS12
Bacteria	Actinobacteria	Actinobacteria	Micromonosporales	Micromonosporaceae	Phytohabitans	suffuscus
Bacteria	Proteobacteria	Gammaproteobacteria	Pseudomonadales	Pseudomonadaceae	Pseudomonas	protegens
Bacteria	Firmicutes	Bacilli	Bacillales	Staphylococcaceae	Staphylococcus	capitis
Bacteria	Proteobacteria	Alphaproteobacteria	Sphingomonadales	Sphingomonadaceae	Sphingomonas	sediminicola
Bacteria	Proteobacteria	Gammaproteobacteria	Pseudomonadales	Pseudomonadaceae	Pseudomonas	fulva
Bacteria	Proteobacteria	Gammaproteobacteria	Enterobacterales	Enterobacteriaceae	Klebsiella	pneumoniae
Bacteria	Bacteroidetes	Bacteroidia	Bacteroidales	Bacteroidaceae	Bacteroides	cellulosilyticus
Bacteria	Proteobacteria	Gammaproteobacteria	Pseudomonadales	Pseudomonadaceae	Pseudomonas	sp._gcc21
Bacteria	Firmicutes	Clostridia	Clostridiales	Clostridiaceae	Clostridium	perfringens
Bacteria	Actinobacteria	Actinobacteria	Micrococcales	Microbacteriaceae	Cryobacterium	soli
Bacteria	Proteobacteria	Alphaproteobacteria	Sphingomonadales	Sphingomonadaceae	Sphingomonas	daechungensis
Bacteria	Proteobacteria	Gammaproteobacteria	Pseudomonadales	Pseudomonadaceae	Pseudomonas	sediminis
Bacteria	Actinobacteria	Actinobacteria	Propionibacteriales	Nocardioidaceae	Friedmanniella	luteola
Bacteria	Proteobacteria	Alphaproteobacteria	Caulobacterales	Caulobacteraceae	Brevundimonas	sp._SGAir0440
Bacteria	Actinobacteria	Coriobacteriia	Coriobacteriales	Coriobacteriaceae	Collinsella	aerofaciens
Bacteria	Actinobacteria	Actinobacteria	Micrococcales	Microbacteriaceae	Microbacterium	sp._A18JL241
Bacteria	Cyanobacteria		Nostocales	Nostocaceae	Nostoc	sphaeroides
Bacteria	Proteobacteria	Alphaproteobacteria	Rhodobacterales	Rhodobacteraceae	Paracoccus	jeotgali
Bacteria	Proteobacteria	Alphaproteobacteria	Sphingomonadales	Sphingomonadaceae	Sphingomonas	sp._HMP9
Bacteria	Actinobacteria	Actinobacteria	Micrococcales	Microbacteriaceae	Microbacterium	hominis
Bacteria	Actinobacteria	Actinobacteria	Micrococcales	Dermacoccaceae	Dermacoccus	sp._PAMC28757
Bacteria	Proteobacteria	Alphaproteobacteria	Sphingomonadales	Sphingomonadaceae	Sphingomonas	sp._MM-1
Bacteria	Proteobacteria	Alphaproteobacteria	Sphingomonadales	Sphingomonadaceae	Novosphingobium	sp._ES2-1
Bacteria	Actinobacteria	Actinobacteria	Micrococcales	Microbacteriaceae	Herbiconiux	sp._SALV-R1
Bacteria	Actinobacteria	Actinobacteria	Propionibacteriales	Propionibacteriaceae	Propionibacterium	freudenreichii
Bacteria	Firmicutes	Bacilli	Bacillales	Bacillaceae	Bacillus	megaterium
Bacteria	Proteobacteria	Alphaproteobacteria	Rhizobiales	Methylobacteriaceae	Methylobacterium	aquaticum
Bacteria	Actinobacteria	Actinobacteria	Corynebacteriales	Nocardiaceae	Rhodococcus	triatomae
Bacteria	Proteobacteria	Gammaproteobacteria	Pseudomonadales	Pseudomonadaceae	Pseudomonas	syringae
Bacteria	Firmicutes	Bacilli	Bacillales	Staphylococcaceae	Staphylococcus	succinus
Bacteria	Proteobacteria	Gammaproteobacteria	Xanthomonadales	Xanthomonadaceae	Lysobacter	soli
Bacteria	Deinococcus-Thermus	Deinococci	Deinococcales	Deinococcaceae	Deinococcus	swuensis
Bacteria	Actinobacteria	Actinobacteria	Corynebacteriales	Mycobacteriaceae	Mycolicibacterium	frederiksbergense
Bacteria	Proteobacteria	Deltaproteobacteria	Myxococcales	Archangiaceae	Melittangium	boletus
Bacteria	Proteobacteria	Alphaproteobacteria	Rhodobacterales	Rhodobacteraceae	Pannonibacter	phragmitetus
Bacteria	Bacteroidetes	Flavobacteriia	Flavobacteriales	Weeksellaceae	Chryseobacterium	manosquense

Bacteria	Actinobacteria	Actinobacteria	Corynebacteriales	Corynebacteriaceae	Corynebacterium	marinum
Bacteria	Proteobacteria	Gammaproteobacteria	Pseudomonadales	Pseudomonadaceae	Pseudomonas	sp._phDV1
Bacteria	Bacteroidetes	Cytophagia	Cytophagales	Hymenobacteraceae	Hymenobacter	sp._DG01
Bacteria	Proteobacteria	Alphaproteobacteria	Rhodospirillales	Acetobacteraceae	Roseococcus	sp._NIBR12
Bacteria	Actinobacteria	Actinobacteria	Micrococcales	Micrococcaceae	Kocuria	varians
Bacteria	Proteobacteria	Gammaproteobacteria	Enterobacterales	Yersiniaceae	Serratia	proteamaculans
Bacteria	Proteobacteria	Epsilonproteobacteria	Campylobacterales	Campylobacteraceae	Campylobacter	ureolyticus
Bacteria	Firmicutes	Clostridia	Clostridiales	Ruminococcaceae	Faecalibacterium	prausnitzii
Bacteria	Proteobacteria	Gammaproteobacteria	Pasteurellales	Pasteurellaceae	Haemophilus	parahaemolyticus
Bacteria	Actinobacteria	Actinobacteria	Corynebacteriales	Corynebacteriaceae	Corynebacterium	mycetoides
Bacteria	Actinobacteria	Actinobacteria	Propionibacteriales	Nocardioidaceae	Nocardioides	sp._dk3136
Bacteria	Actinobacteria	Actinobacteria	Micrococcales	Microbacteriaceae	Microbacterium	sp._WY121
Bacteria	Actinobacteria	Actinobacteria	Propionibacteriales	Propionibacteriaceae	Pseudopropionibacterium	propionicum
Bacteria	Actinobacteria	Actinobacteria	Propionibacteriales	Propionibacteriaceae	Tessaracoccus	flavescens
Bacteria	Actinobacteria	Actinobacteria	Corynebacteriales	Corynebacteriaceae	Corynebacterium	casei
Bacteria	Firmicutes	Bacilli	Lactobacillales	Streptococcaceae	Streptococcus	lutetiensis
Bacteria	Actinobacteria	Actinobacteria	Streptomycetales	Streptomycetaceae	Streptomyces	sp._INR7
Bacteria	Proteobacteria	Alphaproteobacteria	Rickettsiales	Rickettsiaceae	Rickettsia	japonica
Bacteria	Actinobacteria	Actinobacteria	Propionibacteriales	Nocardioidaceae	Nocardioides	sp._JQ2195
Bacteria	Proteobacteria	Gammaproteobacteria	Xanthomonadales	Xanthomonadaceae	Stenotrophomonas	acidaminiphila
Bacteria	Firmicutes	Bacilli	Lactobacillales	Streptococcaceae	Streptococcus	mitis
Bacteria	Actinobacteria	Actinobacteria	Bifidobacteriales	Bifidobacteriaceae	Bifidobacterium	choerinum
Bacteria	Actinobacteria	Actinobacteria	Micrococcales	Micrococcaceae	Kocuria	turfanensis
Bacteria	Proteobacteria	Alphaproteobacteria	Rhodobacterales	Rhodobacteraceae	Paracoccus	sanguinis
Bacteria	Fusobacteria	Fusobacteriia	Fusobacteriales	Leptotrichiaceae	Leptotrichia	trevisanii
Bacteria	Firmicutes	Bacilli	Lactobacillales	Lactobacillaceae	Lactiplantibacillus	plantarum
Bacteria	Proteobacteria	Alphaproteobacteria	Rhizobiales	Methylobacteriaceae	Methylobacterium	terrae
Bacteria	Bacteroidetes	Flavobacteriia	Flavobacteriales	Weeksellaceae	Kaistella	Chryseobacterium_haifense
Bacteria	Actinobacteria	Actinobacteria	Micrococcales	Intrasporangiaceae	Serinicoccus	marinus
Bacteria	Proteobacteria	Gammaproteobacteria	Enterobacterales	Enterobacteriaceae	Cedecea	neteri
Bacteria	Firmicutes	Clostridia	Clostridiales	Peptostreptococcaceae	Flintibacter	sp._KGMB00164
Bacteria	Proteobacteria	Alphaproteobacteria	Rhizobiales	Rhizobiaceae	Rhizobium	phaseoli
Bacteria	Bacteroidetes	Bacteroidia	Bacteroidales	Prevotellaceae	Prevotella	sp._oral_taxon_299
Bacteria	Actinobacteria	Actinobacteria	Pseudonocardiales	Pseudonocardiaceae	Saccharopolyspora	erythraea
Bacteria	Bacteroidetes	Cytophagia	Cytophagales	Hymenobacteraceae	Hymenobacter	nivis
Bacteria	Bacteroidetes	Flavobacteriia	Flavobacteriales	Weeksellaceae	Chryseobacterium	sp._NEB161
Bacteria	Actinobacteria	Actinobacteria	Micrococcales	Micrococcaceae	Rothia	amarae
Bacteria	Bacteroidetes	Flavobacteriia	Flavobacteriales	Flavobacteriaceae	Capnocytophaga	leadbetteri
Bacteria	Firmicutes	Bacilli	Lactobacillales	Lactobacillaceae	Pediococcus	pentosaceus
Bacteria	Actinobacteria	Actinobacteria	Micrococcales	Microbacteriaceae	Microbacterium	lemovicicum
Bacteria	Proteobacteria	Gammaproteobacteria	Xanthomonadales	Xanthomonadaceae	Luteimonas	sp._YGD11-2
Bacteria	Proteobacteria	Alphaproteobacteria	Rhizobiales	Rhizobiaceae	Rhizobium	grahamii
Bacteria	Firmicutes	Bacilli	Bacillales	Staphylococcaceae	Jeotgalicoccus	saudimassiliensis
Bacteria	Firmicutes	Clostridia	Clostridiales	Peptostreptococcaceae	Monoglobus	pectinilyticus
Bacteria	Proteobacteria	Alphaproteobacteria	Sphingomonadales	Sphingomonadaceae	Novosphingobium	resinovorum
Bacteria	Firmicutes	Clostridia	Clostridiales	Oscillospiraceae	Dysosmobacter	welbionis
Bacteria	Actinobacteria	Actinobacteria	Propionibacteriales	Propionibacteriaceae	Tessaracoccus	timonensis
Bacteria	Firmicutes	Bacilli	Bacillales	Planococcaceae	Planomicrobium	glaciei
Bacteria	Actinobacteria	Actinobacteria	Corynebacteriales	Mycobacteriaceae	Mycobacterium	avium
Bacteria	Proteobacteria	Gammaproteobacteria	Enterobacterales	Enterobacteriaceae	Citrobacter	freundii
Bacteria	Actinobacteria	Actinobacteria	Micrococcales	Microbacteriaceae	Microbacterium	sp._NY27
Bacteria	Actinobacteria	Coriobacteriia	Eggerthellales	Eggerthellaceae	Eggerthella	lenta

Bacteria	Proteobacteria	Gammaproteobacteria	Enterobacterales	Enterobacteriaceae	Enterobacter	cancerogenus
Bacteria	Deinococcus-Thermus	Deinococci	Deinococcales	Deinococcaceae	Deinococcus	sp._NW-56
Bacteria	Proteobacteria	Gammaproteobacteria	Xanthomonadales	Xanthomonadaceae	Xanthomonas	arboricola
Bacteria	Actinobacteria	Actinobacteria	Micrococcales	Microbacteriaceae	Agromyces	flavus
Bacteria	Firmicutes	Bacilli	Lactobacillales	Enterococcaceae	Enterococcus	faecium
Bacteria	Proteobacteria	Gammaproteobacteria	Pseudomonadales	Pseudomonadaceae	Pseudomonas	sp._11K1
Bacteria	Actinobacteria	Actinobacteria	Propionibacteriales	Propionibacteriaceae	Cutibacterium	avidum
Bacteria	Actinobacteria	Actinobacteria	Micrococcales	Micrococcaceae	Kocuria	rhizophila
Bacteria	Bacteroidetes	Bacteroidia	Bacteroidales	Rikenellaceae	Alistipes	onderdonkii
Bacteria	Bacteroidetes	Bacteroidia	Bacteroidales		Phocaeicola	dorei
Bacteria	Proteobacteria	Gammaproteobacteria	Xanthomonadales	Xanthomonadaceae	Stenotrophomonas	sp._SAU14A_NAIMI4_5
Bacteria	Actinobacteria	Actinobacteria	Micrococcales	Micrococcaceae	Rothia	aeria
Bacteria	Firmicutes	Tissierellia	Tissierellales	Peptoniphilaceae	Peptoniphilus	harei
Bacteria	Firmicutes	Bacilli	Bacillales	Bacillaceae	Bacillus	thuringiensis
Bacteria	Proteobacteria	Gammaproteobacteria	Xanthomonadales	Xanthomonadaceae	Pseudoxanthomonas	spadix
Bacteria	Proteobacteria	Gammaproteobacteria	Pseudomonadales	Moraxellaceae	Psychrobacter	sp._G
Bacteria	Actinobacteria	Actinobacteria	Corynebacteriales	Corynebacteriaceae	Corynebacterium	nuruki
Bacteria	Actinobacteria	Actinobacteria	Corynebacteriales	Mycobacteriaceae	Mycobacterium	gallinarum
Bacteria	Firmicutes	Bacilli	Lactobacillales	Leuconostocaceae	Weissella	paramesenteroides
Bacteria	Proteobacteria	Gammaproteobacteria	Pseudomonadales	Pseudomonadaceae	Pseudomonas	sp._StFLB209
Bacteria	Bacteroidetes	Sphingobacteriia	Sphingobacteriales	Sphingobacteriaceae	Sphingobacterium	sp._ML3W
Bacteria	Bacteroidetes	Flavobacteriia	Flavobacteriales	Weeksellaceae	Chryseobacterium	sp.
Bacteria	Firmicutes	Bacilli	Bacillales	Paenibacillaceae	Saccharibacillus	brassicae
Bacteria	Proteobacteria	Alphaproteobacteria	Sphingomonadales	Sphingomonadaceae	Sphingomonas	sp._HKS19
Bacteria	Actinobacteria	Actinobacteria	Propionibacteriales	Nocardioidaceae	Aeromicrobium	sp._592
Bacteria	Proteobacteria	Gammaproteobacteria	Enterobacterales	Erwiniaceae	Erwinia	sp._Ejp617
Bacteria	Firmicutes	Bacilli	Lactobacillales	Streptococcaceae	Streptococcus	pyogenes
Bacteria	Bacteroidetes	Flavobacteriia	Flavobacteriales	Weeksellaceae	Weeksella	virosa
Bacteria	Proteobacteria	Gammaproteobacteria	Enterobacterales	Enterobacteriaceae	Kosakonia	cowanii
Bacteria	Firmicutes	Clostridia	Clostridiales	Ruminococcaceae	Ruminococcus	sp._JE7A12
Bacteria	Proteobacteria	Deltaproteobacteria	Desulfovibrionales	Desulfovibrionaceae	Desulfovibrio	piger
Bacteria	Proteobacteria	Alphaproteobacteria	Caulobacterales	Caulobacteraceae	Brevundimonas	sp._MF30-B
Bacteria	Actinobacteria	Actinobacteria	Propionibacteriales	Propionibacteriaceae	Acidipropionibacterium	acidipropionici
Bacteria	Proteobacteria	Gammaproteobacteria	Aeromonadales	Aeromonadaceae	Aeromonas	hydrophila
Bacteria	Actinobacteria	Actinobacteria	Kineosporiales	Kineosporiaceae	Kineococcus	radiotolerans
Bacteria	Bacteroidetes	Flavobacteriia	Flavobacteriales	Flavobacteriaceae	Flavobacterium	anhuiense
Bacteria	Proteobacteria	Gammaproteobacteria	Enterobacterales	Erwiniaceae	Pantoea	ananatis
Bacteria	Proteobacteria	Gammaproteobacteria	Pseudomonadales	Pseudomonadaceae	Pseudomonas	orientalis
Bacteria	Proteobacteria	Gammaproteobacteria	Pseudomonadales	Pseudomonadaceae	Pseudomonas	oleovorans
Bacteria	Actinobacteria	Actinobacteria	Micrococcales	Microbacteriaceae	Leifsonia	xyli
Bacteria	Actinobacteria	Actinobacteria	Micrococcales	Microbacteriaceae	Rathayibacter	sp._VKM_Ac-2762
Bacteria	Actinobacteria	Actinobacteria	Corynebacteriales	Corynebacteriaceae	Corynebacterium	riegelii
Bacteria	Bacteroidetes	Flavobacteriia	Flavobacteriales	Flavobacteriaceae	Myroides	profundi
Bacteria	Bacteroidetes	Sphingobacteriia	Sphingobacteriales	Sphingobacteriaceae	Pedobacter	sp._KBS0701
Bacteria	Actinobacteria	Actinobacteria	Micrococcales	Microbacteriaceae	Microterricola	viridarii
Bacteria	Actinobacteria	Actinobacteria	Micrococcales	Micrococcaceae	Pseudarthrobacter	chlorophenolicus
Bacteria	Proteobacteria	Alphaproteobacteria	Rhizobiales	Hyphomicrobiaceae	Devosia	sp._S02
Bacteria	Actinobacteria	Actinobacteria	Pseudonocardiales	Pseudonocardiaceae	Pseudonocardia	dioxanivorans
Bacteria	Proteobacteria	Betaproteobacteria	Burkholderiales	Burkholderiaceae	Cupriavidus	taiwanensis
Bacteria	Actinobacteria	Actinobacteria	Corynebacteriales	Corynebacteriaceae	Corynebacterium	urealyticum
Bacteria	Bacteroidetes	Sphingobacteriia	Sphingobacteriales	Sphingobacteriaceae	Sphingobacterium	daejeonense
Bacteria	Actinobacteria	Actinobacteria	Corynebacteriales	Corynebacteriaceae	Corynebacterium	kroppenstedtii

Bacteria	Proteobacteria	Gammaproteobacteria	Pseudomonadales	Pseudomonadaceae	Pseudomonas	mendocina
Bacteria	Proteobacteria	Gammaproteobacteria	Pseudomonadales	Pseudomonadaceae	Pseudomonas	sp._SGAir0191
Bacteria	Firmicutes	Clostridia	Clostridiales	Oscillospiraceae	Oscillibacter	sp._NSJ-62
Bacteria	Firmicutes	Bacilli	Bacillales	Staphylococcaceae	Macrococcus	sp._IME1552
Bacteria	Bacteroidetes	Flavobacteriia	Flavobacteriales	Flavobacteriaceae	Capnocytophaga	sputigena
Bacteria	Actinobacteria	Actinobacteria	Corynebacteriales	Corynebacteriaceae	Corynebacterium	cystitidis
Bacteria	Actinobacteria	Actinobacteria	Micrococcales	Microbacteriaceae	Microbacterium	pygmaeum
Bacteria	Firmicutes	Bacilli	Lactobacillales	Lactobacillaceae	Loigolactobacillus	Lactobacillus_coryniformis
Bacteria	Actinobacteria	Actinobacteria	Corynebacteriales	Nocardiaceae	Nocardia	farcinica
Bacteria	Firmicutes	Bacilli	Bacillales	Staphylococcaceae	Staphylococcus	arlettae
Bacteria	Firmicutes	Bacilli	Lactobacillales	Lactobacillaceae	Latilactobacillus	Lactobacillus_curvatus
Bacteria	Actinobacteria	Actinobacteria	Corynebacteriales	Tsukamurellaceae	Tsukamurella	paurometabola
Bacteria	Bacteroidetes	Cytophagia	Cytophagales	Cytophagaceae	Spirosoma	radiotolerans
Bacteria	Proteobacteria	Gammaproteobacteria	Pseudomonadales	Moraxellaceae	Acinetobacter	schindleri
Bacteria	Actinobacteria	Actinobacteria	Corynebacteriales	Corynebacteriaceae	Corynebacterium	stationis
Bacteria	Actinobacteria	Coriobacteriia	Coriobacteriales	Atopobiaceae	Olsenella	umbonata
Bacteria	Proteobacteria	Gammaproteobacteria	Enterobacterales	Enterobacteriaceae	Salmonella	enterica
Bacteria	Proteobacteria	Alphaproteobacteria	Rhizobiales	Methylobacteriaceae	Methylobacterium	brachiatum
Bacteria	Actinobacteria	Actinobacteria	Micrococcales	Micrococcaceae	Rothia	dentocariosa
Bacteria	Proteobacteria	Gammaproteobacteria	Pseudomonadales	Moraxellaceae	Psychrobacter	sp._PRwf-1
Bacteria	Actinobacteria	Actinobacteria	Micrococcales	Ruaniaceae	Ruania	sp._HY168
Bacteria	Actinobacteria	Actinobacteria	Micrococcales	Micrococcaceae	Glutamicibacter	nicotianae
Bacteria	Proteobacteria	Gammaproteobacteria	Enterobacterales	Enterobacteriaceae	Enterobacter	hormaechei
Bacteria	Proteobacteria	Alphaproteobacteria	Rhizobiales	Rhizobiaceae	Rhizobium	leguminosarum
Bacteria	Proteobacteria	Alphaproteobacteria	Rhizobiales	Brucellaceae	Ochrobactrum	anthropi
Bacteria	Proteobacteria	Betaproteobacteria	Burkholderiales	Comamonadaceae	Delftia	acidovorans
Bacteria	Proteobacteria	Alphaproteobacteria	Rhizobiales	Aurantimonadaceae	Aureimonas	sp._AU20
Bacteria	Actinobacteria	Actinobacteria	Corynebacteriales	Mycobacteriaceae	Mycobacterium	grossiae
Bacteria	Actinobacteria	Actinobacteria	Micrococcales	Micrococcaceae	Rothia	terrae
Bacteria	Firmicutes	Bacilli	Bacillales	Staphylococcaceae	Staphylococcus	kloosii
Bacteria	Firmicutes	Bacilli	Lactobacillales	Streptococcaceae	Streptococcus	mutans
Bacteria	Bacteroidetes	Flavobacteriia	Flavobacteriales	Flavobacteriaceae	Flavobacterium	sangjuense
Bacteria	Bacteroidetes	Bacteroidia	Bacteroidales	Prevotellaceae	Prevotella	scopos
Bacteria	Cyanobacteria		Nostocales	Nostocaceae	Nostoc	sp._Lobaria_pulmonaria_(5183)_cyanobiont
Bacteria	Firmicutes	Bacilli	Lactobacillales	Enterococcaceae	Enterococcus	cecorum
Bacteria	Actinobacteria	Actinobacteria	Propionibacteriales	Nocardioidaceae	Nocardioides	seonyuensis
Bacteria	Proteobacteria	Gammaproteobacteria	Pseudomonadales	Pseudomonadaceae	Pseudomonas	viridiflava
Bacteria	Actinobacteria	Actinobacteria	Micrococcales	Dermabacteraceae	Brachybacterium	sp._P6-10-X1
Bacteria	Proteobacteria	Deltaproteobacteria	Myxococcales	Myxococcaceae	Corallococcus	coralloides
Bacteria	Proteobacteria	Gammaproteobacteria	Enterobacterales	Yersiniaceae	Serratia	plymuthica
Bacteria	Actinobacteria	Actinobacteria	Pseudonocardiales	Pseudonocardiaceae	Lentzea	guizhouensis
Bacteria	Proteobacteria	Gammaproteobacteria	Enterobacterales	Erwiniaceae	Pantoea	vagans
Bacteria	Proteobacteria	Gammaproteobacteria	Pseudomonadales	Moraxellaceae	Acinetobacter	haemolyticus
Bacteria	Actinobacteria	Actinobacteria	Micrococcales	Micrococcaceae	Citricoccus	sp._SGAir0253
Bacteria	Actinobacteria	Actinobacteria	Corynebacteriales	Mycobacteriaceae	Lawsonella	clevelandensis
Bacteria	Proteobacteria	Gammaproteobacteria	Xanthomonadales	Xanthomonadaceae	Xanthomonas	campestris
Bacteria	Actinobacteria	Actinobacteria	Streptomycetales	Streptomycetaceae	Streptomyces	albidoflavus
Bacteria	Proteobacteria	Alphaproteobacteria	Rhizobiales	Phyllobacteriaceae	Mesorhizobium	sp._NBSH29
Bacteria	Actinobacteria	Actinobacteria	Actinomycetales	Actinomycetaceae	Actinomyces	radicidentis
Bacteria	Bacteroidetes	Cytophagia	Cytophagales	Hymenobacteraceae	Hymenobacter	sp._BT182
Bacteria	Actinobacteria	Actinobacteria	Propionibacteriales	Nocardioidaceae	Aeromicrobium	sp._zg-629
Bacteria	Proteobacteria	Gammaproteobacteria	Pseudomonadales	Pseudomonadaceae	Pseudomonas	rhodesiae

Bacteria	Actinobacteria	Actinobacteria	Propionibacteriales	Nocardioidaceae	Aeromicrobium	sp._A1-2
Bacteria	Proteobacteria	Alphaproteobacteria	Rhizobiales	Hyphomicrobiaceae	Devosia	ginsengisoli
Bacteria	Firmicutes	Bacilli	Bacillales	Planococcaceae	Solibacillus	silvestris
Bacteria	Proteobacteria	Alphaproteobacteria	Sphingomonadales	Sphingomonadaceae	Sphingosinicella	sp._BN140058
Bacteria	Actinobacteria	Actinobacteria	Corynebacteriales	Corynebacteriaceae	Corynebacterium	sp._ATCC_6931
Bacteria	Actinobacteria	Actinobacteria	Micrococcales	Microbacteriaceae	Curtobacterium	sp._Csp2
Bacteria	Actinobacteria	Actinobacteria	Corynebacteriales	Gordoniaceae	Gordonia	iterans
Bacteria	Proteobacteria	Alphaproteobacteria	Sphingomonadales	Erythrobacteraceae	Erythrobacter	sp._A30-3
Bacteria	Proteobacteria	Gammaproteobacteria	Pseudomonadales	Moraxellaceae	Acinetobacter	dispersus
Bacteria	Proteobacteria	Gammaproteobacteria	Pseudomonadales	Pseudomonadaceae	Pseudomonas	oryzihabitans
Bacteria	Firmicutes	Bacilli	Lactobacillales	Carnobacteriaceae	Jeotgalibaca	dankookensis
Bacteria	Firmicutes	Bacilli	Bacillales	Planococcaceae	Exiguobacterium	mexicanum
Bacteria	Proteobacteria	Gammaproteobacteria	Pseudomonadales	Moraxellaceae	Acinetobacter	ursingii
Bacteria	Actinobacteria	Actinobacteria	Micrococcales	Intrasporangiaceae	Phycicoccus	dokdonensis
Bacteria	Proteobacteria	Gammaproteobacteria	Pseudomonadales	Moraxellaceae	Psychrobacter	urativorans
Bacteria	Proteobacteria	Gammaproteobacteria	Xanthomonadales	Xanthomonadaceae	Luteimonas	sp._JM171
Bacteria	Bacteroidetes	Bacteroidia	Bacteroidales	Tannerellaceae	Tannerella	sp._oral_taxon_HOT-286
Bacteria	Firmicutes	Bacilli	Bacillales	Staphylococcaceae	Staphylococcus	sciuri
Bacteria	Actinobacteria	Actinobacteria	Corynebacteriales	Corynebacteriaceae	Corynebacterium	imitans
Bacteria	Firmicutes	Bacilli	Bacillales	Staphylococcaceae	Staphylococcus	sp._M0911
Bacteria	Firmicutes	Clostridia	Clostridiales	Lachnospiraceae	Lachnoclostridium	phocaeense
Bacteria	Proteobacteria	Gammaproteobacteria	Aeromonadales	Aeromonadaceae	Aeromonas	caviae
Bacteria	Actinobacteria	Actinobacteria	Actinomycetales	Actinomycetaceae	Actinomyces	sp._oral_taxon_169
Bacteria	Actinobacteria	Actinobacteria	Propionibacteriales	Nocardioidaceae	Nocardioides	anomalus
Bacteria	Proteobacteria	Alphaproteobacteria	Sphingomonadales	Erythrobacteraceae	Altererythrobacter	amylolyticus
Bacteria	Actinobacteria	Actinobacteria	Micrococcales	Intrasporangiaceae	Serinicoccus	chungangensis
Bacteria	Actinobacteria	Actinobacteria	Corynebacteriales	Corynebacteriaceae	Corynebacterium	variabile
Bacteria	Actinobacteria	Actinobacteria	Corynebacteriales	Dietziaceae	Dietzia	timorensis
Bacteria	Proteobacteria	Gammaproteobacteria	Pseudomonadales	Pseudomonadaceae	Pseudomonas	sp._TUM18999
Bacteria	Proteobacteria	Alphaproteobacteria	Rhizobiales	Rhizobiaceae	Agrobacterium	tumefaciens
Bacteria	Actinobacteria	Actinobacteria	Pseudonocardiales	Pseudonocardiaceae	Amycolatopsis	sp._Hca4
Bacteria	Proteobacteria	Gammaproteobacteria	Alteromonadales	Shewanellaceae	Shewanella	sp._FDAARGOS_354
Bacteria	Actinobacteria	Actinobacteria	Propionibacteriales	Nocardioidaceae	Nocardioides	sp._S-713
Bacteria	Firmicutes	Clostridia	Clostridiales	Peptostreptococcaceae	Intestinimonas	butyriciproducens
Bacteria	Firmicutes	Negativicutes	Acidaminococcales	Acidaminococcaceae	Acidaminococcus	intestini
Bacteria	Proteobacteria	Betaproteobacteria	Burkholderiales	Burkholderiaceae	Burkholderia	cenocepacia
Bacteria	Firmicutes	Bacilli	Lactobacillales	Leuconostocaceae	Weissella	hellenica
Bacteria	Firmicutes	Erysipelotrichia	Erysipelotrichales	Erysipelotrichaceae	Faecalitalea	cylindroides
Bacteria	Bacteroidetes	Cytophagia	Cytophagales	Cytophagaceae	Dyadobacter	fermentans
Bacteria	Firmicutes	Bacilli	Bacillales	Staphylococcaceae	Staphylococcus	cohnii
Bacteria	Actinobacteria	Actinobacteria	Micrococcales	Dermabacteraceae	Dermabacter	vaginalis
Bacteria	Proteobacteria	Gammaproteobacteria	Pseudomonadales	Pseudomonadaceae	Pseudomonas	sp._ATCC_13867
Bacteria	Proteobacteria	Alphaproteobacteria	Rhizobiales	Methylobacteriaceae	Methylobacterium	mesophilicum
Bacteria	Firmicutes	Bacilli	Lactobacillales	Carnobacteriaceae	Jeotgalibaca	porci
Bacteria	Actinobacteria	Actinobacteria	Micrococcales	Ruaniaceae	Haloactinobacterium	sp._HY164
Bacteria	Proteobacteria	Gammaproteobacteria	Enterobacterales	Yersiniaceae	Serratia	liquefaciens
Bacteria	Proteobacteria	Betaproteobacteria	Neisseriales	Neisseriaceae	Neisseria	mucosa
Bacteria	Bacteroidetes	Bacteroidia	Bacteroidales	Rikenellaceae	Alistipes	indistinctus
Bacteria	Proteobacteria	Betaproteobacteria	Neisseriales	Neisseriaceae	Neisseria	flavescens
Bacteria	Firmicutes	Bacilli	Lactobacillales	Lactobacillaceae	Ligilactobacillus	Lactobacillus_animalis
Bacteria	Proteobacteria	Betaproteobacteria	Burkholderiales	Burkholderiaceae	Cupriavidus	metallidurans
Bacteria	Actinobacteria	Actinobacteria	Micrococcales	Microbacteriaceae	Rathayibacter	sp._VKM_Ac-2759

Bacteria	Bacteroidetes	Flavobacteriia	Flavobacteriales	Weeksellaceae	Chryseobacterium	taihuense
Bacteria	Bacteroidetes	Cytophagia	Cytophagales	Hymenobacteraceae	Hymenobacter	sp._PAMC_26554
Bacteria	Firmicutes	Clostridia	Clostridiales	Lachnospiraceae	Blautia	hansenii
Bacteria	Fusobacteria	Fusobacteriia	Fusobacteriales	Fusobacteriaceae	Fusobacterium	pseudoperiodonticum
Bacteria	Firmicutes	Bacilli	Lactobacillales	Lactobacillaceae	Amylolactobacillus	amylophilus
Bacteria	Proteobacteria	Betaproteobacteria	Burkholderiales	Comamonadaceae	Acidovorax	sp._KKS102
Bacteria	Proteobacteria	Gammaproteobacteria	Enterobacterales	Enterobacteriaceae	Kosakonia	sp._CCTCC_M2018092
Archaea	Euryarchaeota	Halobacteria	Halobacteriales	Halobacteriaceae	Halalkalicoccus	jeotgali
Bacteria	Proteobacteria	Betaproteobacteria	Burkholderiales	Alcaligenaceae	Rubrivivax	gelatinosus
Bacteria	Actinobacteria	Actinobacteria	Propionibacteriales	Nocardioidaceae	Friedmanniella	sagamiharensis
Bacteria	Proteobacteria	Betaproteobacteria	Burkholderiales	Alcaligenaceae	Achromobacter	xylosoxidans
Bacteria	Actinobacteria	Coriobacteriia	Eggerthellales	Eggerthellaceae	Denitrobacterium	detoxificans
Bacteria	Actinobacteria	Actinobacteria	Micrococcales	Micrococcaceae	Arthrobacter	alpinus
Bacteria	Actinobacteria	Actinobacteria	Streptomycetales	Streptomycetaceae	Streptomyces	clavuligerus
Bacteria	Firmicutes	Bacilli	Lactobacillales	Streptococcaceae	Streptococcus	suis
Bacteria	Proteobacteria	Gammaproteobacteria	Pseudomonadales	Moraxellaceae	Psychrobacter	sp._P2G3
Bacteria	Bacteroidetes	Flavobacteriia	Flavobacteriales	Flavobacteriaceae	Flavobacterium	sp._M31R6
Bacteria	Bacteroidetes	Flavobacteriia	Flavobacteriales	Flavobacteriaceae	Capnocytophaga	gingivalis
Bacteria	Proteobacteria	Gammaproteobacteria	Enterobacterales	Morganellaceae	Morganella	morganii
Bacteria	Firmicutes	Tissierellia	Tissierellales	Peptoniphilaceae	Ezakiella	massiliensis
Bacteria	Actinobacteria	Actinobacteria	Micrococcales	Microbacteriaceae	Microbacterium	sp._RG1
Bacteria	Actinobacteria	Actinobacteria	Geodermatophilales	Geodermatophilaceae	Blastococcus	saxobsidens
Bacteria	Proteobacteria	Alphaproteobacteria	Rhodobacterales	Rhodobacteraceae	Paracoccus	sp._AK26
Bacteria	Actinobacteria	Actinobacteria	Micrococcales	Microbacteriaceae	Rathayibacter	sp._VKM_Ac-2801
Bacteria	Proteobacteria	Gammaproteobacteria	Xanthomonadales	Xanthomonadaceae	Stenotrophomonas	sp._169
Bacteria	Proteobacteria	Gammaproteobacteria	Pseudomonadales	Moraxellaceae	Acinetobacter	sp._10FS3-1
Bacteria	Firmicutes	Negativicutes	Veillonellales	Veillonellaceae	Megasphaera	elsdenii
Bacteria	Proteobacteria	Gammaproteobacteria	Pseudomonadales	Pseudomonadaceae	Pseudomonas	monteilii
Bacteria	Proteobacteria	Gammaproteobacteria	Pseudomonadales	Pseudomonadaceae	Pseudomonas	azotoformans
Bacteria	Actinobacteria	Actinobacteria	Corynebacteriales	Mycobacteriaceae	Mycobacteroides	abscessus
Bacteria	Proteobacteria	Gammaproteobacteria	Pseudomonadales	Pseudomonadaceae	Pseudomonas	sihuiensis
Bacteria	Proteobacteria	Alphaproteobacteria	Rhizobiales	Methylobacteriaceae	Methylobacterium	nodulans
Bacteria	Proteobacteria	Alphaproteobacteria	Sphingomonadales	Sphingomonadaceae	Sphingomonas	taxi
Bacteria	Actinobacteria	Actinobacteria	Actinomycetales	Actinomycetaceae	Schaalia	odontolytica
Bacteria	Bacteroidetes	Bacteroidia	Bacteroidales	Bacteroidaceae	Bacteroides	sp._PHL_2737
Bacteria	Firmicutes	Bacilli	Lactobacillales	Lactobacillaceae	Lactobacillus	amylovorus
Bacteria	Firmicutes	Bacilli	Bacillales	Bacillaceae	Alkalihalobacillus	clausii
Bacteria	Bacteroidetes	Cytophagia	Cytophagales	Hymenobacteraceae	Hymenobacter	sp._PAMC_26628
Bacteria	Bacteroidetes	Bacteroidia	Bacteroidales	Prevotellaceae	Prevotella	intermedia
Bacteria	Proteobacteria	Betaproteobacteria	Burkholderiales	Burkholderiaceae	Burkholderia	plantarii
Bacteria	Actinobacteria	Actinobacteria	Micrococcales	Microbacteriaceae	Microbacterium	sp._XT11
Bacteria	Actinobacteria	Actinobacteria	Actinomycetales	Actinomycetaceae	Actinotignum	schaalii
Bacteria	Bacteroidetes	Bacteroidia	Bacteroidales	Prevotellaceae	Prevotella	denticola
Bacteria	Firmicutes	Erysipelotrichia	Erysipelotrichales	Erysipelotrichaceae	Turicibacter	sp._H121
Bacteria	Actinobacteria	Actinobacteria	Micrococcales	Micrococcaceae	Arthrobacter	sp._YC-RL1
Bacteria	Bacteroidetes	Flavobacteriia	Flavobacteriales	Flavobacteriaceae	Myroides	odoratus
Bacteria	Actinobacteria	Actinobacteria	Micrococcales	Intrasporangiaceae	Ornithinimicrobium	sp._H23M54
Bacteria	Proteobacteria	Gammaproteobacteria	Pseudomonadales	Pseudomonadaceae	Pseudomonas	sp._J380
Bacteria	Bacteroidetes	Cytophagia	Cytophagales	Hymenobacteraceae	Hymenobacter	sp._S2-20-2
Bacteria	Bacteroidetes	Cytophagia	Cytophagales	Hymenobacteraceae	Hymenobacter	sp._DG25B
Bacteria	Firmicutes	Bacilli	Bacillales	Staphylococcaceae	Staphylococcus	warneri
Bacteria	Firmicutes	Bacilli	Lactobacillales	Aerococcaceae	Aerococcus	urinaeequi

Bacteria	Actinobacteria	Actinobacteria	Micrococcales	Bogoriellaceae	Georgenia	sp._Z294
Bacteria	Actinobacteria	Actinobacteria	Micrococcales	Microbacteriaceae	Microbacterium	oxydans
Bacteria	Proteobacteria	Alphaproteobacteria	Sphingomonadales	Sphingomonadaceae	Sphingobium	cloacae
Bacteria	Proteobacteria	Gammaproteobacteria	Pseudomonadales	Moraxellaceae	Psychrobacter	cryohalolentis
Bacteria	Proteobacteria	Gammaproteobacteria	Pseudomonadales	Pseudomonadaceae	Pseudomonas	putida
Bacteria	Actinobacteria	Actinobacteria	Corynebacteriales	Mycobacteriaceae	Mycolicibacterium	phocaicum
Bacteria	Bacteroidetes	Bacteroidia	Bacteroidales	Porphyromonadaceae	Porphyromonas	asaccharolytica
Bacteria	Actinobacteria	Actinobacteria	Corynebacteriales	Mycobacteriaceae	Mycobacteroides	immunogenum
Bacteria	Firmicutes	Bacilli	Lactobacillales	Lactobacillaceae	Ligilactobacillus	salivarius
Bacteria	Fusobacteria	Fusobacteriia	Fusobacteriales	Fusobacteriaceae	Fusobacterium	nucleatum
Bacteria	Actinobacteria	Actinobacteria	Corynebacteriales	Corynebacteriaceae	Corynebacterium	maris
Bacteria	Actinobacteria	Actinobacteria	Corynebacteriales	Nocardiaceae	Rhodococcus	sp._WMMA185
Bacteria	Proteobacteria	Alphaproteobacteria	Sphingomonadales	Sphingomonadaceae	Sphingomonas	sp._NBWT7
Bacteria	Bacteroidetes	Bacteroidia	Bacteroidales	Bacteroidaceae	Bacteroides	sp._HF-162
Bacteria	Actinobacteria	Actinobacteria	Propionibacteriales	Nocardioidaceae	Nocardioides	sp._dk884
Bacteria	Proteobacteria	Gammaproteobacteria	Enterobacterales	Enterobacteriaceae	Raoultella	planticola
Bacteria	Firmicutes	Clostridia	Clostridiales	Ruminococcaceae	Ruminococcus	champanellensis
Bacteria	Proteobacteria	Gammaproteobacteria	Pasteurellales	Pasteurellaceae	Aggregatibacter	aphrophilus
Bacteria	Proteobacteria	Gammaproteobacteria	Enterobacterales	Enterobacteriaceae	Candidatus_Hamiltonella	defensa
Bacteria	Proteobacteria	Gammaproteobacteria	Pseudomonadales	Moraxellaceae	Moraxella	catarrhalis
Bacteria	Actinobacteria	Actinobacteria	Actinomycetales	Actinomycetaceae	Actinomyces	naeslundii
Bacteria	Actinobacteria	Actinobacteria	Propionibacteriales	Propionibacteriaceae	Acidipropionibacterium	jensenii
Bacteria	Proteobacteria	Gammaproteobacteria	Oceanospirillales	Alcanivoracaceae	Alcanivorax	sp._N3-2A
Bacteria	Actinobacteria	Actinobacteria	Micrococcales	Micrococcaceae	Micrococcus	luteus
Bacteria	Actinobacteria	Actinobacteria	Micrococcales	Microbacteriaceae	Microbacterium	sp._No._7
Bacteria	Actinobacteria	Actinobacteria	Propionibacteriales	Nocardioidaceae	Nocardioides	marinisabuli
Bacteria	Actinobacteria	Actinobacteria	Corynebacteriales	Gordoniaceae	Gordonia	bronchialis
Bacteria	Proteobacteria	Alphaproteobacteria	Rhizobiales	Phyllobacteriaceae	Mesorhizobium	sp._M6A.T.Cr.TU.016.01.1.1
Bacteria	Proteobacteria	Alphaproteobacteria	Rhizobiales	Brucellaceae	Brucella	abortus
Bacteria	Bacteroidetes	Bacteroidia	Bacteroidales	Prevotellaceae	Prevotella	jejuni
Bacteria	Bacteroidetes	Flavobacteriia	Flavobacteriales	Flavobacteriaceae	Capnocytophaga	canimorsus
Bacteria	Firmicutes	Bacilli	Bacillales	Staphylococcaceae	Staphylococcus	condimenti
Bacteria	Proteobacteria	Alphaproteobacteria	Rhodobacterales	Rhodobacteraceae	Phaeobacter	inhibens
Bacteria	Firmicutes	Bacilli	Lactobacillales	Lactobacillaceae	Levilactobacillus	Lactobacillus_brevis
Bacteria	Proteobacteria	Gammaproteobacteria	Pseudomonadales	Moraxellaceae	Psychrobacter	sp._KCTC_72983
Bacteria	Bacteroidetes	Bacteroidia	Bacteroidales	Odoribacteraceae	Odoribacter	splanchnicus
Bacteria	Proteobacteria	Gammaproteobacteria	Pseudomonadales	Pseudomonadaceae	Pseudomonas	poae
Bacteria	Actinobacteria	Actinobacteria	Corynebacteriales	Mycobacteriaceae	Mycolicibacterium	tokaiense
Bacteria	Firmicutes	Bacilli	Lactobacillales	Streptococcaceae	Streptococcus	parasanguinis
Bacteria	Firmicutes	Bacilli	Lactobacillales	Streptococcaceae	Streptococcus	cristatus
Bacteria	Actinobacteria	Actinobacteria	Micrococcales	Microbacteriaceae	Protaetiibacter	sp._SSC-01
Bacteria	Actinobacteria	Actinobacteria	Actinomycetales	Actinomycetaceae	Actinomyces	sp._oral_taxon_171
Bacteria	Actinobacteria	Actinobacteria	Micrococcales	Microbacteriaceae	Microbacterium	sp._4R-513
Bacteria	Actinobacteria	Actinobacteria	Propionibacteriales	Nocardioidaceae	Nocardioides	sp._CF8
Bacteria	Proteobacteria	Gammaproteobacteria	Enterobacterales	Erwiniaceae	Pantoea	alhagi
Bacteria	Cyanobacteria		Nostocales	Scytonemataceae	Scytonema	sp._NIES-4073
Bacteria	Actinobacteria	Actinobacteria	Micrococcales	Sanguibacteraceae	Sanguibacter	sp._HDW7
Bacteria	Actinobacteria	Actinobacteria	Micrococcales	Micrococcaceae	Glutamicibacter	mishrai
Bacteria	Proteobacteria	Gammaproteobacteria	Pseudomonadales	Pseudomonadaceae	Pseudomonas	sp._C27(2019)
Bacteria	Proteobacteria	Gammaproteobacteria	Xanthomonadales	Xanthomonadaceae	Stenotrophomonas	sp._LM091
Bacteria	Firmicutes	Bacilli	Lactobacillales	Streptococcaceae	Streptococcus	equinus
Bacteria	Actinobacteria	Actinobacteria	Streptomycetales	Streptomycetaceae	Streptomyces	sp._GS7

Bacteria	Firmicutes	Clostridia	Clostridiales	Lachnospiraceae	Roseburia	intestinalis
Bacteria	Proteobacteria	Gammaproteobacteria	Xanthomonadales	Xanthomonadaceae	Pseudoxanthomonas	suwonensis
Bacteria	Proteobacteria	Gammaproteobacteria	Pseudomonadales	Moraxellaceae	Acinetobacter	calcoaceticus
Bacteria	Planctomycetes	Planctomycetia	Planctomycetales	Planctomycetaceae	Planctomyces	sp._SH-PL62
Bacteria	Actinobacteria	Actinobacteria	Bifidobacteriales	Bifidobacteriaceae	Gardnerella	leopoldii
Bacteria	Actinobacteria	Actinobacteria	Corynebacteriales	Mycobacteriaceae	Mycolicibacterium	smegmatis
Bacteria	Actinobacteria	Actinobacteria	Micrococcales	Brevibacteriaceae	Brevibacterium	sp._YB235
Bacteria	Proteobacteria	Alphaproteobacteria	Caulobacterales	Caulobacteraceae	Brevundimonas	naejangsanensis
Bacteria	Actinobacteria	Actinobacteria	Corynebacteriales	Mycobacteriaceae	Mycobacterium	shigaense
Bacteria	Actinobacteria	Actinobacteria	Corynebacteriales	Corynebacteriaceae	Corynebacterium	minutissimum
Bacteria	Firmicutes	Bacilli	Bacillales	Bacillaceae	Bacillus	subtilis
Bacteria	Firmicutes	Clostridia	Clostridiales	Lachnospiraceae	Lachnoclostridium	Clostridium_scindens
Bacteria	Proteobacteria	Betaproteobacteria	Burkholderiales	Oxalobacteraceae	Massilia	albidiflava
Bacteria	Proteobacteria	Alphaproteobacteria	Sphingomonadales	Sphingomonadaceae	Sphingomonas	lutea
Bacteria	Firmicutes	Bacilli	Lactobacillales	Lactobacillaceae	Limosilactobacillus	Lactobacillus_mucosae
Bacteria	Bacteroidetes	Cytophagia	Cytophagales	Hymenobacteraceae	Hymenobacter	sp._HDW8
Bacteria	Proteobacteria	Gammaproteobacteria	Pseudomonadales	Moraxellaceae	Acinetobacter	sp._WCHA45
Bacteria	Proteobacteria	Betaproteobacteria	Burkholderiales	Comamonadaceae	Hydrogenophaga	sp._NH-16
Bacteria	Actinobacteria	Actinobacteria	Micrococcales	Intrasporangiaceae	Janibacter	sp._YB324
Bacteria	Actinobacteria	Actinobacteria	Micrococcales	Micrococcaceae	Micrococcus	sp._KBS0714
Bacteria	Proteobacteria	Gammaproteobacteria	Enterobacterales	Erwiniaceae	Erwinia	gerundensis
Bacteria	Proteobacteria	Epsilonproteobacteria	Campylobacterales	Campylobacteraceae	Campylobacter	jejuni
Bacteria	Firmicutes	Clostridia	Clostridiales	Peptostreptococcaceae	Clostridioides	difficile
Bacteria	Bacteroidetes	Bacteroidia	Bacteroidales	Prevotellaceae	Prevotella	ruminicola
Bacteria	Firmicutes	Negativicutes	Acidaminococcales	Acidaminococcaceae	Phascolarctobacterium	faecium
Bacteria	Proteobacteria	Gammaproteobacteria	Vibrionales	Vibrionaceae	Vibrio	rumoiensis
Bacteria	Firmicutes	Clostridia	Clostridiales	Clostridiaceae	Clostridium	botulinum
Bacteria	Proteobacteria	Gammaproteobacteria	Pseudomonadales	Pseudomonadaceae	Pseudomonas	sp._Os17
Bacteria	Proteobacteria	Betaproteobacteria	Burkholderiales	Comamonadaceae	Comamonas	aquatica
Bacteria	Fusobacteria	Fusobacteriia	Fusobacteriales	Leptotrichiaceae	Leptotrichia	wadei
Bacteria	Actinobacteria	Actinobacteria	Bifidobacteriales	Bifidobacteriaceae	Bifidobacterium	longum
Bacteria	Actinobacteria	Actinobacteria	Actinomycetales	Actinomycetaceae	Actinomyces	oris
Bacteria	Actinobacteria	Actinobacteria	Bifidobacteriales	Bifidobacteriaceae	Parascardovia	denticolens
Bacteria	Actinobacteria	Actinobacteria	Corynebacteriales	Corynebacteriaceae	Corynebacterium	sanguinis
Bacteria	Bacteroidetes	Cytophagia	Cytophagales	Cytophagaceae	Spirosoma	sp._CJU-R4
Bacteria	Proteobacteria	Alphaproteobacteria	Rhizobiales	Rhizobiaceae	Neorhizobium	sp._NCHU2750
Bacteria	Proteobacteria	Gammaproteobacteria	Xanthomonadales	Xanthomonadaceae	Luteimonas	chenhongjianii
Bacteria	Actinobacteria	Actinobacteria	Micrococcales	Microbacteriaceae	Microbacterium	sp._LKL04
Bacteria	Proteobacteria	Alphaproteobacteria	Caulobacterales	Caulobacteraceae	Brevundimonas	sp._LM2
Bacteria	Actinobacteria	Actinobacteria	Micrococcales	Microbacteriaceae	Cryobacterium	sp._LW097
Bacteria	Actinobacteria	Actinobacteria	Propionibacteriales	Propionibacteriaceae	Propionibacterium	sp._oral_taxon_193
Bacteria	Proteobacteria	Gammaproteobacteria	Pseudomonadales	Moraxellaceae	Acinetobacter	sp._WCHA55
Bacteria	Proteobacteria	Alphaproteobacteria	Rhizobiales	Methylobacteriaceae	Methylobacterium	sp._DM1
Bacteria	Firmicutes	Bacilli	Lactobacillales	Streptococcaceae	Streptococcus	sp._oral_taxon_061
Bacteria	Proteobacteria	Gammaproteobacteria	Xanthomonadales	Xanthomonadaceae	Thermomonas	carbonis
Bacteria	Actinobacteria	Actinobacteria	Micrococcales	Intrasporangiaceae	Serinicoccus	hydrothermalis
Bacteria	Proteobacteria	Alphaproteobacteria	Sphingomonadales	Sphingomonadaceae	Sphingomonas	sp._HMP6
Bacteria	Firmicutes	Bacilli	Lactobacillales	Streptococcaceae	Streptococcus	sp._HSISM1
Bacteria	Proteobacteria	Gammaproteobacteria	Pseudomonadales	Pseudomonadaceae	Pseudomonas	synxantha
Bacteria	Proteobacteria	Gammaproteobacteria	Enterobacterales	Enterobacteriaceae	Citrobacter	portucalensis
Bacteria	Actinobacteria	Actinobacteria	Corynebacteriales	Gordoniaceae	Gordonia	sp._135
Bacteria	Bacteroidetes	Bacteroidia	Bacteroidales	Bacteroidaceae	Bacteroides	sp._HF-5141

Bacteria	Actinobacteria	Actinobacteria	Corynebacteriales	Corynebacteriaceae	Corynebacterium	sp._2019
Bacteria	Proteobacteria	Betaproteobacteria	Burkholderiales	Alcaligenaceae	Bordetella	parapertussis
Bacteria	Bacteroidetes	Flavobacteriia	Flavobacteriales	Weeksellaceae	Kaistella	Chryseobacterium_carnis
Bacteria	Actinobacteria	Actinobacteria	Corynebacteriales	Nocardiaceae	Nocardia	sp._WCH-YHL-001
Bacteria	Proteobacteria	Alphaproteobacteria	Rhodospirillales	Acetobacteraceae	Acetobacter	orientalis
Bacteria	Actinobacteria	Actinobacteria	Propionibacteriales	Nocardioidaceae	Nocardioides	sp._HDW12B
Bacteria	Gemmatimonadetes	Gemmatimonadetes	Gemmatimonadales	Gemmatimonadaceae	Gemmatirosa	kalamazoonesis
Bacteria	Proteobacteria	Betaproteobacteria	Burkholderiales	Comamonadaceae	Alicycliphilus	denitrificans
Bacteria	Actinobacteria	Actinobacteria	Geodermatophilales	Geodermatophilaceae	Modestobacter	marinus
Bacteria	Bacteroidetes	Bacteroidia	Bacteroidales	Bacteroidaceae	Bacteroides	sp._M10
Bacteria	Firmicutes	Clostridia	Clostridiales	Ruminococcaceae	Flavonifractor	plautii
Bacteria	Firmicutes	Bacilli	Bacillales	Staphylococcaceae	Staphylococcus	lentus
Bacteria	Firmicutes	Bacilli	Lactobacillales	Streptococcaceae	Streptococcus	canis
Bacteria	Proteobacteria	Gammaproteobacteria	Xanthomonadales	Xanthomonadaceae	Thermomonas	sp._SY21
Bacteria	Proteobacteria	Betaproteobacteria	Burkholderiales	Burkholderiaceae	Ralstonia	solanacearum
Bacteria	Firmicutes	Clostridia	Clostridiales	Oscillospiraceae	Oscillibacter	sp._PEA192
Bacteria	Proteobacteria	Betaproteobacteria	Burkholderiales	Oxalobacteraceae	Massilia	armeniaca
Bacteria	Actinobacteria	Actinobacteria	Micromonosporales	Micromonosporaceae	Actinoplanes	friuliensis
Bacteria	Firmicutes	Bacilli	Bacillales	Bacillaceae	Bacillus	mycoides
Bacteria	Actinobacteria	Actinobacteria	Micrococcales	Micrococcaceae	Rothia	mucilaginosa
Bacteria	Actinobacteria	Actinobacteria	Micrococcales	Microbacteriaceae	Microbacterium	sp._PM5
Bacteria	Firmicutes	Bacilli	Lactobacillales	Streptococcaceae	Streptococcus	sp._116-D4
Bacteria	Actinobacteria	Actinobacteria	Corynebacteriales	Corynebacteriaceae	Corynebacterium	glutamicum
Bacteria	Proteobacteria	Alphaproteobacteria	Rhizobiales	Bartonellaceae	Bartonella	grahamii
Bacteria	Firmicutes	Bacilli	Lactobacillales	Lactobacillaceae	Ligilactobacillus	Lactobacillus_murinus
Bacteria	Actinobacteria	Actinobacteria	Micrococcales	Micrococcaceae	Glutamicibacter	creatinolyticus
Bacteria	Actinobacteria	Actinobacteria	Micrococcales	Cellulomonadaceae	Cellulomonas	shaoxiangyii
Bacteria	Actinobacteria	Actinobacteria	Corynebacteriales	Mycobacteriaceae	Mycobacterium	chimaera
Bacteria	Proteobacteria	Alphaproteobacteria	Caulobacterales	Caulobacteraceae	Brevundimonas	sp._DS20
Bacteria	Bacteroidetes	Cytophagia	Cytophagales	Hymenobacteraceae	Hymenobacter	qilianensis
Bacteria	Actinobacteria	Actinobacteria	Micrococcales	Microbacteriaceae	Microbacterium	sp._10M-3C3
Bacteria	Actinobacteria	Actinobacteria	Pseudonocardiales	Pseudonocardiaceae	Saccharomonospora	azurea
Bacteria	Actinobacteria	Actinobacteria	Micrococcales	Microbacteriaceae	Curtobacterium	flaccumfaciens
Bacteria	Actinobacteria	Actinobacteria	Corynebacteriales	Corynebacteriaceae	Corynebacterium	frankenforstense
Bacteria	Proteobacteria	Gammaproteobacteria	Pseudomonadales	Pseudomonadaceae	Pseudomonas	extremorientalis
Bacteria	Proteobacteria	Betaproteobacteria	Burkholderiales	Oxalobacteraceae	Massilia	sp._WG5
Bacteria	Proteobacteria	Alphaproteobacteria	Caulobacterales	Caulobacteraceae	Brevundimonas	subvibrioides
Bacteria	Firmicutes	Bacilli	Bacillales	Staphylococcaceae	Salinicoccus	halodurans
Bacteria	Firmicutes	Bacilli	Lactobacillales	Streptococcaceae	Streptococcus	gordonii
Bacteria	Proteobacteria	Alphaproteobacteria	Rhizobiales	Rhizobiaceae	Neorhizobium	sp._SOG26
Bacteria	Proteobacteria	Betaproteobacteria	Burkholderiales	Alcaligenaceae	Rhizobacter	gummiphilus
Bacteria	Bacteroidetes	Sphingobacteriia	Sphingobacteriales	Sphingobacteriaceae	Sphingobacterium	sp._DR205
Bacteria	Actinobacteria	Actinobacteria	Micrococcales	Micrococcaceae	Glutamicibacter	arilaitensis
Bacteria	Actinobacteria	Coriobacteriia	Coriobacteriales	Atopobiaceae	Lancefieldella	parvula
Bacteria	Firmicutes	Negativicutes	Selenomonadales	Selenomonadaceae	Megamonas	funiformis
Bacteria	Proteobacteria	Alphaproteobacteria	Rhodobacterales	Rhodobacteraceae	Paracoccus	sp._Arc7-R13
Bacteria	Proteobacteria	Gammaproteobacteria	Enterobacterales	Erwiniaceae	Erwinia	persicina
Bacteria	Proteobacteria	Alphaproteobacteria	Rhizobiales	Bradyrhizobiaceae	Bradyrhizobium	sp._CCBAU_051011
Bacteria	Proteobacteria	Gammaproteobacteria	Enterobacterales	Erwiniaceae	Pantoea	sp._At-9b
Bacteria	Actinobacteria	Actinobacteria	Corynebacteriales	Mycobacteriaceae	Mycolicibacterium	aurum
Bacteria	Proteobacteria	Gammaproteobacteria	Enterobacterales	Erwiniaceae	Pantoea	sp._MT58
Bacteria	Actinobacteria	Actinobacteria	Micrococcales	Microbacteriaceae	Microbacterium	sp._Nx66

Bacteria	Firmicutes	Tissierellia	Tissierellales	Peptoniphilaceae	Finegoldia	magna
Bacteria	Proteobacteria	Gammaproteobacteria	Pseudomonadales	Pseudomonadaceae	Pseudomonas	alcaligenes
Bacteria	Bacteroidetes	Bacteroidia	Bacteroidales	Odoribacteraceae	Butyricimonas	faecalis
Bacteria	Actinobacteria	Actinobacteria	Micrococcales	Cellulomonadaceae	Actinotalea	sp._JY-7876
Bacteria	Firmicutes	Tissierellia	Tissierellales	Peptoniphilaceae	Anaerococcus	mediterraneensis
Bacteria	Proteobacteria	Gammaproteobacteria	Pseudomonadales	Pseudomonadaceae	Pseudomonas	sp._LTJR-52
Bacteria	Firmicutes	Bacilli	Lactobacillales	Lactobacillaceae	Ligilactobacillus	agilis
Bacteria	Actinobacteria	Actinobacteria	Corynebacteriales	Gordoniaceae	Gordonia	rubripertincta
Bacteria	Actinobacteria	Actinobacteria	Micrococcales	Microbacteriaceae	Clavibacter	michiganensis
Bacteria	Firmicutes	Bacilli	Lactobacillales	Streptococcaceae	Streptococcus	sp._A12
Bacteria	Firmicutes	Bacilli	Lactobacillales	Lactobacillaceae	Lactobacillus	iners
Bacteria	Actinobacteria	Actinobacteria	Micrococcales	Intrasporangiaceae	Ornithinimicrobium	sp._HY006
Bacteria	Actinobacteria	Actinobacteria	Micrococcales	Microbacteriaceae	Rathayibacter	festucae
Bacteria	Proteobacteria	Gammaproteobacteria	Pseudomonadales	Pseudomonadaceae	Pseudomonas	lundensis
Bacteria	Actinobacteria	Actinobacteria	Corynebacteriales	Corynebacteriaceae	Corynebacterium	segmentosum
Bacteria	Proteobacteria	Gammaproteobacteria	Pseudomonadales	Moraxellaceae	Acinetobacter	soli
Bacteria	Actinobacteria	Actinobacteria	Corynebacteriales	Corynebacteriaceae	Corynebacterium	callunae
Bacteria	Bacteroidetes	Bacteroidia	Bacteroidales	Tannerellaceae	Parabacteroides	distasonis
Bacteria	Firmicutes	Erysipelotrichia	Erysipelotrichales	Erysipelotrichaceae	Erysipelatoclostridium	ramosum

Table S5. Overall microbial diversity in relation to pulmonary function parameters and FeNO.

	Main model adjusted for covariates*			Additional adjustment for season (winter/not)		
	Estimate	Standard Error	P-value	Estimate	Standard Error	P-value
**Richness (untransformed)**						
FEV_1	0.6404	0.4811	0.1835	0.6454	0.4815	0.1805
FVC	0.0626	0.5467	0.9089	0.0569	0.5473	0.9172
FEV_1/FVC	0.0001	7.31e-05	0.1257	0.0001	7.30e-05	0.1141
FeNO	0.0009	0.0006	0.1268	0.0009	0.0006	0.1352
**Shannon H Index (exponential transformed)**						
FEV_1	0.52529	0.6997	0.4530	0.5354	0.7006	0.4450
FVC	-0.1275	0.7942	0.8725	-0.1397	0.7953	0.8606
FEV_1/FVC	0.0001	0.0001	0.2014	0.0001	0.0001	0.1784
FeNO	0.0006	0.0009	0.5075	0.0005	0.0009	0.5343

FEV_1 and FVC were measured in ml, FEV_1/FVC was a proportion (0-1), and FeNO was measured in ppb. FeNO values were natural log (ln) transformed for normality. In multivariable linear regression models, microbial diversity measures were the predictor, and pulmonary function traits were the outcome.

*Age, age squared, sex, height, height squared, weight (for FVC only), smoking (never/former/current), pack-years, asthma, state of residence, and ancestry (European/not).

Table S6. Taxa differentially abundant (P-value<0.05 after accounting for all other taxa examined together) in relation to one or more pulmonary function parameters and/or FeNO.

			FEV_1		FVC		FEV_1/FVC		FeNO	
Phylum	Family	Genus	Coef	P-value	Coef	P-value	Coef	P-value	Coef	P-value
Acidobacteria	*Vicinamibacteraceae*	*Luteitalea*	-0.066	0.012	-0.021	0.286	-0.096	0.016	0.041	0.225
Actinobacteria	*Actinomycetaceae*	*Actinomyces*	-0.033	0.322	-0.035	0.245	-0.032	0.513	-0.125	0.010
Actinobacteria	*Actinomycetaceae*	*Pauljensenia*	0.036	0.087	0.013	0.413	0.072	0.026	-0.038	0.440
Actinobacteria	*Atopobiaceae*	*Olsenella*	-0.009	0.699	-0.004	0.847	0.009	0.701	0.106	0.019
Actinobacteria	*Bifidobacteriaceae*	*Bifidobacterium*	0.013	0.529	-0.012	0.538	0.067	0.005	-0.016	0.642
Actinobacteria	*Bifidobacteriaceae*	*Parascardovia*	-0.055	0.012	-0.032	0.128	-0.103	0.003	0.006	0.890
Actinobacteria	*Cellulomonadaceae*	*Cellulomonas*	-0.062	0.004	-0.057	0.003	-0.054	0.347	0.045	0.217
Actinobacteria	*Conexibacteraceae*	*Conexibacter*	0.081	0.020	0.060	0.076	0.040	0.418	-0.067	0.308
Actinobacteria	*Dermacoccaceae*	*Kytococcus*	0.046	0.038	0.047	0.025	0.013	0.718	0.009	0.814
Actinobacteria	*Geodermatophilaceae*	*Blastococcus*	0.032	0.080	0.020	0.225	0.027	0.312	-0.066	0.039
Actinobacteria	*Ilumatobacteraceae*	*Ilumatobacter*	0.068	0.001	0.051	0.015	0.041	0.135	0.028	0.455
Actinobacteria	*Intrasporangiaceae*	*Intrasporangium*	-0.026	0.338	-0.022	0.355	-0.032	0.391	-0.145	3.4E-04
Actinobacteria	*Intrasporangiaceae*	*Janibacter*	-0.009	0.747	-0.020	0.402	0.006	0.807	-0.069	0.037
Actinobacteria	*Intrasporangiaceae*	*Ornithinimicrobium*	-0.006	0.843	0.037	0.291	-0.112	0.004	0.043	0.249
Actinobacteria	*Intrasporangiaceae*	*Phycicoccus*	-0.015	0.678	-0.038	0.240	0.084	0.047	-0.070	0.153
Actinobacteria	*Microbacteriaceae*	*Cnuibacter*	0.004	0.887	-0.014	0.575	0.066	0.042	-0.051	0.278
Actinobacteria	*Microbacteriaceae*	*Cryobacterium*	0.076	0.013	0.058	0.040	0.051	0.125	0.035	0.429
Actinobacteria	*Microbacteriaceae*	*Frigoribacterium*	0.064	0.005	0.078	0.001	-0.005	0.885	-0.013	0.704
Actinobacteria	*Microbacteriaceae*	*Herbiconiux*	-0.015	0.405	0.016	0.347	-0.064	0.016	-0.015	0.540
Actinobacteria	*Microbacteriaceae*	*Microbacterium*	0.044	0.121	0.004	0.881	0.099	0.020	-0.106	0.034
Actinobacteria	*Microbacteriaceae*	*Rathayibacter*	-0.016	0.391	0.008	0.560	-0.079	2.1E-04	-0.002	0.946
Actinobacteria	*Micrococcaceae*	*Arthrobacter*	-0.021	0.401	-0.048	0.058	0.070	0.043	0.034	0.422
Actinobacteria	*Micrococcaceae*	*Kocuria*	0.043	0.040	0.044	0.026	0.016	0.572	0.025	0.478
Actinobacteria	*Micromonosporaceae*	*Micromonospora*	0.051	0.070	0.045	0.044	0.034	0.455	-0.042	0.365
Actinobacteria	*Nocardioidaceae*	*Friedmanniella*	0.059	0.023	0.017	0.515	0.117	0.001	0.071	0.097
Actinobacteria	*Nocardioidaceae*	*Kribbella*	0.017	0.298	0.014	0.427	-0.003	0.908	-0.081	0.012
Actinobacteria	*Nocardiopsaceae*	*Nocardiopsis*	0.028	0.132	0.043	0.026	-0.036	0.193	0.027	0.379
Actinobacteria	*Propionibacteriaceae*	*Cutibacterium*	-0.014	0.646	0.013	0.646	-0.101	0.011	-0.002	0.969
Actinobacteria	*Propionibacteriaceae*	*Pseudopropionibacterium*	0.020	0.225	0.008	0.628	0.053	0.049	-0.010	0.760
Actinobacteria	*Ruaniaceae*	*Haloactinobacterium*	-0.020	0.343	0.008	0.729	-0.066	0.022	0.026	0.423
Actinobacteria	*Rubrobacteraceae*	*Rubrobacter*	-0.004	0.785	-0.023	0.111	0.061	0.012	0.017	0.578
Actinobacteria	*Streptosporangiaceae*	*Streptosporangium*	0.035	0.031	0.020	0.230	0.040	0.110	0.005	0.852
Bacteroidetes	*Amoebophilaceae*	*Candidatus_Cardinium*	0.040	0.101	0.012	0.610	0.048	0.049	0.057	0.068
Bacteroidetes	*Blattabacteriaceae*	*Candidatus_Sulcia*	0.033	0.086	0.036	0.049	0.005	0.859	-0.073	0.033
Bacteroidetes	*Cytophagaceae*	*Spirosoma*	0.058	0.028	0.039	0.134	0.050	0.130	-0.018	0.657
Bacteroidetes	*Flavobacteriaceae*	*Capnocytophaga*	0.011	0.679	0.010	0.674	0.001	0.983	0.132	0.011
Bacteroidetes	*Flavobacteriaceae*	*Flavobacterium*	0.026	0.320	0.013	0.608	0.032	0.343	0.089	0.038
Bacteroidetes	*Hymenobacteraceae*	*Adhaeribacter*	-0.023	0.327	-0.029	0.199	0.018	0.634	-0.077	0.036
Bacteroidetes	*Rikenellaceae*	*Alistipes*	-0.014	0.717	0.045	0.192	-0.132	0.025	-0.027	0.619
Bacteroidetes	*Weeksellaceae*	*Elizabethkingia*	0.006	0.787	-0.011	0.583	0.055	0.045	-0.030	0.403
Bacteroidetes	*Weeksellaceae*	*Weeksella*	-0.015	0.160	-0.014	0.184	0.011	0.656	0.039	0.024
Cyanobacteria	*Chroococcidiopsidaceae*	*Chroococcidiopsis*	0.026	0.046	0.036	0.007	-0.008	0.716	0.002	0.909
Cyanobacteria	*Nostocaceae*	*Nostoc*	-0.076	0.036	-0.060	0.066	-0.040	0.340	-0.028	0.431
Cyanobacteria	*Oscillatoriaceae*	*Oscillatoria*	0.038	0.129	0.053	0.033	-0.020	0.555	-0.053	0.170
Cyanobacteria	*Scytonemataceae*	*Scytonema*	-0.035	0.107	-0.037	0.044	-0.014	0.591	0.074	0.046
Deinococcus-Thermus	*Deinococcaceae*	*Deinococcus*	-0.037	0.049	-0.033	0.061	0.012	0.657	-0.020	0.489
Firmicutes	*Acidaminococcaceae*	*Acidaminococcus*	-0.043	0.022	-0.034	0.024	-0.039	0.312	0.014	0.788
Firmicutes	*Bacillaceae*	*Salicibibacter*	0.001	0.969	-0.001	0.955	-0.003	0.937	0.098	0.030

Firmicutes	*Enterococcaceae*	*Enterococcus*	-0.024	0.167	-0.030	0.118	0.061	0.008	-0.004	0.881
Firmicutes	*Erysipelotrichaceae*	*Erysipelatoclostridium*	-0.075	0.029	-0.073	0.014	-0.057	0.258	0.027	0.565
Firmicutes	*Lachnospiraceae*	*Anaerobutyricum*	-0.072	0.045	-0.078	0.006	0.015	0.794	-0.030	0.536
Firmicutes	*Lachnospiraceae*	*Blautia*	0.091	0.180	0.139	0.038	0.012	0.868	0.011	0.893
Firmicutes	*Lachnospiraceae*	*Lachnoclostridium*	-0.064	0.129	-0.089	0.032	0.003	0.956	0.014	0.798
Firmicutes	*Lactobacillaceae*	*Limosilactobacillus*	-0.032	0.293	0.023	0.485	-0.085	0.037	-0.087	0.027
Firmicutes	*Paenibacillaceae*	*Saccharibacillus*	0.034	0.049	0.030	0.047	0.003	0.912	-0.034	0.312
Firmicutes	*Peptoniphilaceae*	*Finegoldia*	-0.050	0.121	-0.056	0.044	0.030	0.438	0.016	0.747
Firmicutes	*Peptostreptococcaceae*	*Clostridioides*	0.036	0.297	0.080	0.011	-0.064	0.223	0.006	0.906
Firmicutes	*Peptostreptococcaceae*	*Flintibacter*	0.030	0.220	0.059	0.020	-0.054	0.173	0.034	0.494
Firmicutes	*Peptostreptococcaceae*	*Massilistercora*	-0.006	0.798	0.001	0.970	-0.005	0.916	-0.076	0.017
Firmicutes	*Peptostreptococcaceae*	*Monoglobus*	0.051	0.022	0.037	0.059	0.049	0.182	-0.059	0.087
Firmicutes	*Planococcaceae*	*Planococcus*	-0.046	0.062	-0.050	0.014	-0.006	0.869	0.070	0.035
Firmicutes	*Planococcaceae*	*Solibacillus*	0.008	0.830	0.025	0.326	-0.015	0.719	-0.081	4.9E-04
Firmicutes	*Staphylococcaceae*	*Auricoccus*	0.006	0.734	-0.014	0.410	0.052	0.022	-0.013	0.635
Firmicutes	*Staphylococcaceae*	*Jeotgalicoccus*	0.028	0.411	0.023	0.461	0.036	0.471	0.124	0.027
Firmicutes	*Streptococcaceae*	*Lactococcus*	0.045	0.066	0.060	0.004	0.012	0.703	-0.031	0.294
Firmicutes	*Streptococcaceae*	*Streptococcus*	-0.047	0.144	-0.023	0.387	-0.144	0.011	0.074	0.376
Firmicutes	*Veillonellaceae*	*Megasphaera*	0.044	0.074	0.010	0.737	0.073	0.049	-0.005	0.914
Planctomycetes	*Planctomycetaceae*	*Planctomyces*	0.013	0.425	0.021	0.144	-0.006	0.844	0.079	0.011
Proteobacteria	*Alcaligenaceae*	*Achromobacter*	-0.052	0.005	-0.042	0.028	-0.053	0.152	-0.034	0.509
Proteobacteria	*Alcaligenaceae*	*Alcaligenes*	-0.006	0.819	-0.032	0.212	0.069	0.012	0.025	0.507
Proteobacteria	*Alcaligenaceae*	*Rhizobacter*	-0.052	0.004	-0.036	0.025	-0.032	0.279	0.053	0.087
Proteobacteria	*Alcaligenaceae*	*Rubrivivax*	-0.010	0.635	-0.035	0.050	0.044	0.120	-0.021	0.564
Proteobacteria	*Alcanivoracaceae*	*Alcanivorax*	-0.023	0.485	-0.027	0.341	-0.003	0.916	0.096	3.2E-05
Proteobacteria	*Anaplasmataceae*	*Wolbachia*	-0.009	0.480	-0.018	0.139	0.049	0.027	0.019	0.501
Proteobacteria	*Bradyrhizobiaceae*	*Rhodopseudomonas*	-0.020	0.178	-0.004	0.794	-0.042	0.208	0.071	0.035
Proteobacteria	*Bradyrhizobiaceae*	*Variibacter*	0.018	0.421	-0.014	0.441	0.091	0.002	0.026	0.472
Proteobacteria	*Brucellaceae*	*Ochrobactrum*	0.033	0.224	0.045	0.038	-0.002	0.967	0.086	0.007
Proteobacteria	*Enterobacteriaceae*	*Klebsiella*	0.050	0.145	0.071	0.026	-0.007	0.867	-0.078	0.164
Proteobacteria	*Enterobacteriaceae*	*Leclercia*	-0.021	0.398	-0.045	0.068	0.083	0.029	-0.050	0.274
Proteobacteria	*Enterobacteriaceae*	*Lelliottia*	0.003	0.850	-0.021	0.123	0.042	2.7E-04	-0.046	8.0E-04
Proteobacteria	*Erwiniaceae*	*Buchnera*	0.031	0.186	0.015	0.583	0.036	0.026	-0.007	0.765
Proteobacteria	*Erythrobacteraceae*	*Erythrobacter*	-0.001	0.947	0.023	0.140	-0.030	0.207	-0.041	0.004
Proteobacteria	*Halomonadaceae*	*Halomonas*	-0.001	0.952	-0.009	0.561	0.040	0.012	0.021	0.112
Proteobacteria	*Hyphomicrobiaceae*	*Devosia*	0.060	0.031	0.061	0.023	0.001	0.973	0.029	0.511
Proteobacteria	*Methylobacteriaceae*	*Methylorubrum*	0.055	0.004	0.056	0.006	0.011	0.772	-0.018	0.613
Proteobacteria	*Moraxellaceae*	*Moraxella*	0.015	0.457	-0.013	0.525	0.060	0.009	0.003	0.904
Proteobacteria	*Pasteurellaceae*	*Frederiksenia*	0.023	0.270	0.023	0.378	0.027	0.568	-0.151	6.4E-05
Proteobacteria	*Pasteurellaceae*	*Haemophilus*	0.071	0.020	0.085	0.004	-0.014	0.720	-0.042	0.412
Proteobacteria	*Pseudomonadaceae*	*Pseudomonas*	-0.074	0.047	-0.055	0.095	-0.075	0.143	-0.015	0.814
Proteobacteria	*Rhizobiaceae*	*Shinella*	0.000	0.981	-0.033	0.052	0.076	0.023	-0.018	0.613
Proteobacteria	*Rhodobacteraceae*	*Haematobacter*	-0.029	0.030	-0.036	0.007	0.012	0.662	0.009	0.749
Proteobacteria	*Rhodobacteraceae*	*Pannonibacter*	0.009	0.695	0.021	0.237	-0.031	0.479	0.075	0.023
Proteobacteria	*Rhodobacteraceae*	*Tabrizicola*	-0.012	0.485	-0.022	0.165	0.068	0.014	-0.005	0.873
Proteobacteria	*Shewanellaceae*	*Shewanella*	0.031	0.008	0.016	0.205	0.037	0.006	0.011	0.752
Proteobacteria	*Sphingomonadaceae*	*Sphingosinithalassobacter*	-0.049	0.010	-0.038	0.026	-0.045	0.146	0.036	0.328
Proteobacteria	*Xanthomonadaceae*	*Lysobacter*	-0.012	0.661	-0.002	0.936	-0.040	0.212	0.096	0.011
Proteobacteria	*Xanthomonadaceae*	*Stenotrophomonas*	-0.012	0.593	0.006	0.730	-0.034	0.275	0.115	2.9E-04
Proteobacteria	*Yersiniaceae*	*Ewingella*	0.034	0.460	0.061	0.046	-0.039	0.632	0.015	0.785
Proteobacteria	*Yersiniaceae*	*Rouxiella*	0.080	0.092	0.074	0.096	0.023	0.697	0.190	0.023

All genera were from Bacteria.

Table S7. Taxa differentially abundant (P-value<0.05 after accounting for all other taxa examined together) in relation to one or more pulmonary function parameters and/or FeNO: Species level analysis results.

				FEV_1		FVC		FEV_1/FVC		FeNO	
Phylum	Family	Genus	Species	Coef	P-value	Coef	P-value	Coef	P-value	Coef	P-value
Acidobacteria	*Vicinamibacteraceae*	*Luteitalea*	*pratensis*	-0.058	0.010	-0.023	0.232	-0.082	0.017	0.057	0.143
Actinobacteria	*Actinomycetaceae*	*Actinomyces*	*naeslundii*	-0.047	0.142	-0.059	0.041	0.009	0.851	-0.048	0.346
Actinobacteria	*Actinomycetaceae*	*Actinomyces*	*oris*	0.077	0.017	0.073	0.017	0.016	0.740	0.048	0.324
Actinobacteria	*Actinomycetaceae*	*Actinomyces*	*sp._oral_taxon_897*	-0.004	0.871	-0.009	0.627	0.025	0.596	-0.135	0.001
Actinobacteria	*Actinomycetaceae*	*Pauljensenia*	*hongkongensis*	0.026	0.186	0.009	0.576	0.066	0.029	-0.043	0.427
Actinobacteria	*Bifidobacteriaceae*	*Bifidobacterium*	*bifidum*	0.039	0.044	0.027	0.168	0.051	0.040	-0.059	0.059
Actinobacteria	*Bifidobacteriaceae*	*Bifidobacterium*	*catenulatum*	0.005	0.835	-0.016	0.445	0.062	0.040	-0.013	0.810
Actinobacteria	*Bifidobacteriaceae*	*Bifidobacterium*	*longum*	-0.041	0.010	-0.041	0.007	0.018	0.450	-0.031	0.351
Actinobacteria	*Bifidobacteriaceae*	*Bifidobacterium*	*pseudocatenulatum*	-0.056	0.011	-0.066	0.001	0.004	0.912	0.098	0.044
Actinobacteria	*Bifidobacteriaceae*	*Parascardovia*	*denticolens*	-0.060	0.007	-0.030	0.154	-0.103	0.004	-0.006	0.898
Actinobacteria	*Brevibacteriaceae*	*Brevibacterium*	*siliguriense*	-0.038	0.363	-0.105	0.009	0.143	0.049	0.095	0.218
Actinobacteria	*Brevibacteriaceae*	*Brevibacterium*	*sp._CS2*	-0.025	0.281	-0.006	0.808	-0.073	0.014	0.018	0.749
Actinobacteria	*Brevibacteriaceae*	*Brevibacterium*	*sp._Marine*	-0.034	0.378	-0.064	0.022	0.014	0.858	0.085	0.135
Actinobacteria	*Brevibacteriaceae*	*Brevibacterium*	*sp._o2*	0.058	0.073	0.050	0.044	0.078	0.106	-0.029	0.458
Actinobacteria	*Brevibacteriaceae*	*Brevibacterium*	*sp._YB235*	0.007	0.800	0.029	0.237	-0.070	0.050	0.048	0.473
Actinobacteria	*Cellulomonadaceae*	*Cellulomonas*	*fimi*	-0.045	0.037	-0.040	0.028	-0.007	0.820	0.048	0.181
Actinobacteria	*Conexibacteraceae*	*Conexibacter*	*woesei*	0.068	0.012	0.050	0.060	0.022	0.565	-0.055	0.359
Actinobacteria	*Corynebacteriaceae*	*Corynebacterium*	*diphtheriae*	0.039	0.029	0.023	0.165	0.061	0.018	-0.016	0.673
Actinobacteria	*Corynebacteriaceae*	*Corynebacterium*	*doosanense*	0.006	0.663	-0.004	0.723	0.034	0.023	0.001	0.944
Actinobacteria	*Corynebacteriaceae*	*Corynebacterium*	*genitalium*	0.049	0.046	0.020	0.279	0.063	0.084	0.004	0.917
Actinobacteria	*Corynebacteriaceae*	*Corynebacterium*	*maris*	0.077	0.014	0.097	0.007	-0.002	0.953	0.043	0.310
Actinobacteria	*Corynebacteriaceae*	*Corynebacterium*	*matruchotii*	0.041	0.132	0.048	0.042	-0.036	0.453	-0.043	0.333
Actinobacteria	*Corynebacteriaceae*	*Corynebacterium*	*riegelii*	-0.005	0.823	0.000	0.991	-0.003	0.951	-0.113	0.050
Actinobacteria	*Corynebacteriaceae*	*Corynebacterium*	*timonense*	0.011	0.588	0.024	0.190	-0.034	0.322	-0.070	0.035
Actinobacteria	*Corynebacteriaceae*	*Corynebacterium*	*urealyticum*	-0.027	0.116	-0.023	0.174	0.007	0.796	-0.079	0.012
Actinobacteria	*Corynebacteriaceae*	*Corynebacterium*	*ureicelerivorans*	-0.068	0.013	-0.045	0.018	-0.076	0.184	0.022	0.527
Actinobacteria	*Corynebacteriaceae*	*Corynebacterium*	*xerosis*	-0.012	0.765	-0.026	0.447	0.018	0.682	0.130	0.034
Actinobacteria	*Dermabacteraceae*	*Brachybacterium*	*saurashtrense*	0.020	0.530	-0.006	0.836	0.092	0.055	-0.169	0.012
Actinobacteria	*Dermabacteraceae*	*Dermabacter*	*vaginalis*	-0.018	0.581	-0.015	0.583	-0.094	0.047	-0.035	0.276
Actinobacteria	*Dermacoccaceae*	*Dermacoccus*	*sp._PAMC28757*	-0.033	0.035	-0.029	0.063	-0.026	0.226	-0.046	0.258
Actinobacteria	*Geodermatophilaceae*	*Blastococcus*	*saxobsidens*	0.028	0.121	0.013	0.433	0.028	0.305	-0.073	0.033
Actinobacteria	*Gordoniaceae*	*Gordonia*	*sp._JH63*	0.052	0.060	0.005	0.823	0.108	4.4E-04	-0.014	0.726
Actinobacteria	*Ilumatobacteraceae*	*Ilumatobacter*	*coccineus*	0.061	0.003	0.048	0.016	0.028	0.318	0.035	0.389
Actinobacteria	*Intrasporangiaceae*	*Intrasporangium*	*calvum*	-0.017	0.506	-0.011	0.613	-0.035	0.348	-0.156	9.9E-05
Actinobacteria	*Intrasporangiaceae*	*Serinicoccus*	*hydrothermalis*	-0.019	0.521	-0.009	0.716	-0.063	0.178	-0.119	0.017
Actinobacteria	*Intrasporangiaceae*	*Serinicoccus*	*profundi*	0.052	0.012	0.036	0.108	0.063	0.009	0.001	0.974
Actinobacteria	*Microbacteriaceae*	*Cryobacterium*	*soli*	0.015	0.467	-0.017	0.359	0.068	0.034	-0.095	0.014
Actinobacteria	*Microbacteriaceae*	*Cryobacterium*	*sp._LW097*	0.024	0.447	0.032	0.228	-0.019	0.648	0.118	0.026
Actinobacteria	*Microbacteriaceae*	*Curtobacterium*	*pusillum*	0.005	0.838	0.019	0.406	-0.017	0.630	0.099	0.012
Actinobacteria	*Microbacteriaceae*	*Curtobacterium*	*sp._Csp2*	-0.008	0.763	-0.023	0.301	0.080	0.041	-0.035	0.545
Actinobacteria	*Microbacteriaceae*	*Curtobacterium*	*sp._SGAir0471*	0.075	0.015	0.038	0.136	0.115	0.006	-0.026	0.541
Actinobacteria	*Microbacteriaceae*	*Microbacterium*	*aurum*	0.033	0.131	0.014	0.432	0.055	0.141	-0.125	0.001
Actinobacteria	*Microbacteriaceae*	*Microbacterium*	*oleivorans*	0.040	0.121	0.037	0.096	0.007	0.868	-0.129	0.004
Actinobacteria	*Microbacteriaceae*	*Microbacterium*	*sp._HY82*	-0.060	0.020	-0.042	0.076	-0.066	0.112	0.032	0.518
Actinobacteria	*Microbacteriaceae*	*Microbacterium*	*sp._Nx66*	0.045	0.063	0.011	0.597	0.078	0.011	0.031	0.423
Actinobacteria	*Microbacteriaceae*	*Microbacterium*	*sp._RG1*	-0.005	0.845	-0.003	0.877	0.000	0.999	0.078	0.033

Actinobacteria	*Microbacteriaceae*	*Microbacterium*	*sp._WY121*	-0.003	0.855	-0.004	0.809	0.007	0.771	-0.097	0.018
Actinobacteria	*Microbacteriaceae*	*Microbacterium*	*testaceum*	0.043	0.147	0.043	0.122	0.037	0.405	-0.157	0.006
Actinobacteria	*Microbacteriaceae*	*Plantibacter*	*sp._M259*	-0.025	0.516	0.042	0.236	-0.172	0.009	-0.144	0.033
Actinobacteria	*Micrococcaceae*	*Arthrobacter*	*sp._YC-RL1*	-0.040	0.089	-0.052	0.031	0.076	0.018	0.033	0.401
Actinobacteria	*Micrococcaceae*	*Glutamicibacter*	*nicotianae*	-0.027	0.260	-0.023	0.288	-0.024	0.458	0.126	0.006
Actinobacteria	*Micrococcaceae*	*Kocuria*	*palustris*	0.055	0.014	0.052	0.019	0.005	0.844	0.040	0.188
Actinobacteria	*Micromonosporaceae*	*Micromonospora*	*zamorensis*	0.056	0.007	0.022	0.260	0.070	0.003	-0.076	0.096
Actinobacteria	*Mycobacteriaceae*	*Mycobacteroides*	*immunogenum*	-0.006	0.722	-0.023	0.144	0.076	0.005	-0.009	0.768
Actinobacteria	*Mycobacteriaceae*	*Mycolicibacterium*	*rhodesiae*	-0.045	0.052	-0.043	0.037	-0.027	0.481	0.016	0.726
Actinobacteria	*Nocardiaceae*	*Rhodococcus*	*coprophilus*	-0.039	0.093	-0.004	0.865	-0.077	0.019	-0.002	0.953
Actinobacteria	*Nocardiaceae*	*Rhodococcus*	*ruber*	-0.042	0.066	-0.044	0.011	-0.036	0.326	0.026	0.385
Actinobacteria	*Nocardiaceae*	*Rhodococcus*	*sp._PBTS_2*	0.065	0.006	0.054	0.010	0.024	0.518	0.036	0.366
Actinobacteria	*Nocardiaceae*	*Rhodococcus*	*sp._WMMA185*	0.029	0.066	0.011	0.480	0.059	0.013	0.021	0.556
Actinobacteria	*Nocardioidaceae*	*Aeromicrobium*	*sp._MF47*	0.033	0.102	0.038	0.033	-0.031	0.320	-0.037	0.346
Actinobacteria	*Nocardioidaceae*	*Friedmanniella*	*luteola*	0.021	0.387	-0.013	0.600	0.076	0.016	-0.040	0.368
Actinobacteria	*Nocardioidaceae*	*Friedmanniella*	*sagamiharensis*	0.017	0.602	-0.023	0.465	0.122	0.006	0.064	0.236
Actinobacteria	*Nocardioidaceae*	*Kribbella*	*qitaiheensis*	0.036	0.144	0.019	0.328	0.006	0.890	-0.067	0.029
Actinobacteria	*Nocardioidaceae*	*Micropruina*	*glycogenica*	-0.029	0.131	-0.017	0.287	-0.038	0.241	-0.064	0.048
Actinobacteria	*Nocardioidaceae*	*Nocardioides*	*marinisabuli*	-0.035	0.086	-0.049	0.011	0.032	0.273	-0.005	0.873
Actinobacteria	*Nocardioidaceae*	*Nocardioides*	*mesophilus*	-0.009	0.651	-0.019	0.310	0.002	0.945	-0.080	0.032
Actinobacteria	*Nocardioidaceae*	*Nocardioides*	*sp._dk3136*	0.047	0.034	0.036	0.090	0.034	0.179	0.009	0.814
Actinobacteria	*Nocardioidaceae*	*Nocardioides*	*sp._JQ2195*	-0.034	0.245	-0.011	0.654	-0.066	0.192	-0.064	0.034
Actinobacteria	*Propionibacteriaceae*	*Cutibacterium*	*avidum*	-0.012	0.586	-0.011	0.539	-0.002	0.956	0.073	0.024
Actinobacteria	*Propionibacteriaceae*	*Propionibacterium*	*sp._oral_taxon_193*	-0.007	0.684	-0.004	0.682	-0.003	0.916	-0.076	0.032
Actinobacteria	*Ruaniaceae*	*Haloactinobacterium*	*sp._HY164*	-0.023	0.257	-0.002	0.907	-0.067	0.016	0.026	0.427
Actinobacteria	*Rubrobacteraceae*	*Rubrobacter*	*sp._SCSIO_52909*	-0.001	0.926	-0.013	0.352	0.059	0.015	0.012	0.715
Bacteroidetes	*Amoebophilaceae*	*Candidatus_Cardinium*	*Cardinium_endosymbiont_of_Sogatella_furcifera*	0.038	0.100	0.010	0.651	0.052	0.037	0.061	0.052
Bacteroidetes	*Bacteroidaceae*	*Bacteroides*	*caccae*	-0.069	0.028	-0.048	0.074	-0.103	0.041	-0.053	0.295
Bacteroidetes	*Bacteroidaceae*	*Bacteroides*	*caecimuris*	0.012	0.664	-0.015	0.544	0.090	0.050	0.093	0.047
Bacteroidetes	*Bacteroidaceae*	*Bacteroides*	*sp._A1C1*	-0.062	0.046	-0.030	0.269	-0.058	0.291	0.013	0.810
Bacteroidetes	*Bacteroidaceae*	*Bacteroides*	*sp._CACC_737*	0.071	0.056	0.073	0.007	0.021	0.701	-0.040	0.403
Bacteroidetes	*Bacteroidaceae*	*Bacteroides*	*sp._HF-5141*	0.069	0.074	0.032	0.308	0.123	0.047	-0.015	0.822
Bacteroidetes	*Bacteroidaceae*	*Bacteroides*	*sp._HF-5287*	-0.052	0.067	-0.019	0.456	-0.081	0.036	-0.079	0.015
Bacteroidetes	*Bacteroidaceae*	*Bacteroides*	*xylanisolvens*	-0.079	0.070	-0.077	0.024	-0.060	0.399	0.015	0.829
Bacteroidetes	*Bacteroidaceae*	*Phocaeicola*	*vulgatus*	0.037	0.347	-0.012	0.721	0.152	0.025	-0.018	0.789
Bacteroidetes	*Blattabacteriaceae*	*Candidatus_Sulcia*	*muelleri*	0.034	0.080	0.034	0.057	0.002	0.949	-0.078	0.029
Bacteroidetes	*Cytophagaceae*	*Spirosoma*	*rigui*	0.062	0.022	0.050	0.070	0.031	0.462	0.069	0.127
Bacteroidetes	*Flavobacteriaceae*	*Capnocytophaga*	*gingivalis*	0.018	0.466	-0.016	0.480	0.100	0.014	0.075	0.102
Bacteroidetes	*Flavobacteriaceae*	*Flavobacterium*	*anhuiense*	-0.004	0.850	-0.009	0.691	0.026	0.400	0.109	0.003
Bacteroidetes	*Flavobacteriaceae*	*Flavobacterium*	*sp._MDT1-60*	0.022	0.373	-0.010	0.681	0.081	0.011	0.095	0.010
Bacteroidetes	*Hymenobacteraceae*	*Adhaeribacter*	*sp._KUDC8001*	0.007	0.735	0.008	0.691	0.008	0.793	-0.090	0.015
Bacteroidetes	*Hymenobacteraceae*	*Hymenobacter*	*russus*	0.018	0.427	0.046	0.022	-0.038	0.278	0.022	0.475
Bacteroidetes	*Hymenobacteraceae*	*Hymenobacter*	*sedentarius*	0.068	0.027	0.044	0.126	0.061	0.151	-0.030	0.526
Bacteroidetes	*Hymenobacteraceae*	*Hymenobacter*	*sp._APR13*	0.043	0.026	0.037	0.032	0.030	0.232	0.000	0.999
Bacteroidetes	*Hymenobacteraceae*	*Hymenobacter*	*sp._BRD128*	0.062	0.007	0.022	0.302	0.051	0.130	0.019	0.621
Bacteroidetes	*Hymenobacteraceae*	*Hymenobacter*	*sp._PAMC_26554*	-0.010	0.631	0.010	0.594	-0.053	0.082	-0.089	0.018
Bacteroidetes	*Hymenobacteraceae*	*Hymenobacter*	*sp._PAMC_26628*	0.041	0.040	0.018	0.339	0.047	0.089	-0.043	0.267
Bacteroidetes	*Porphyromonadaceae*	*Porphyromonas*	*crevioricanis*	-0.008	0.550	-0.020	0.089	0.023	0.424	0.074	0.012
Bacteroidetes	*Sphingobacteriaceae*	*Pedobacter*	*sp._KBS0701*	0.018	0.344	-0.001	0.965	0.079	0.007	0.030	0.420
Bacteroidetes	*Sphingobacteriaceae*	*Pedobacter*	*suwonensis*	-0.025	0.269	-0.019	0.241	0.007	0.824	0.103	0.040

Bacteroidetes	*Sphingobacteriaceae*	*Sphingobacterium*	*daejeonense*	0.017	0.491	0.006	0.797	-0.010	0.749	0.077	0.037
Bacteroidetes	*Sphingobacteriaceae*	*Sphingobacterium*	*lactis*	0.022	0.128	0.045	3.9E-04	-0.048	0.171	0.010	0.687
Bacteroidetes	*Sphingobacteriaceae*	*Sphingobacterium*	*mizutaii*	0.001	0.973	-0.007	0.743	-0.007	0.826	0.084	0.008
Bacteroidetes	*Sphingobacteriaceae*	*Sphingobacterium*	*sp._ML3W*	0.028	0.432	0.033	0.367	0.006	0.868	0.111	0.010
Bacteroidetes	*Tannerellaceae*	*Tannerella*	*forsythia*	0.017	0.432	-0.002	0.912	0.071	0.043	-0.051	0.246
Bacteroidetes	*Weeksellaceae*	*Chryseobacterium*	*taihuense*	0.051	0.024	0.035	0.219	0.039	0.148	0.033	0.358
Bacteroidetes	*Weeksellaceae*	*Cruoricaptor*	*ignavus*	-0.060	0.025	-0.038	0.128	-0.030	0.367	0.016	0.660
Bacteroidetes	*Weeksellaceae*	*Empedobacter*	*stercoris*	0.053	0.057	0.051	0.043	0.012	0.702	0.017	0.675
Cyanobacteria	*Chroococcidiopsidaceae*	*Chroococcidiopsis*	*thermalis*	0.038	0.045	0.042	0.012	0.003	0.923	0.000	0.987
Cyanobacteria	*Nostocaceae*	*Nostoc*	*sp._Lobaria_pulmonaria_(5183)_cyanobiont*	-0.066	0.030	-0.050	0.063	-0.044	0.341	0.034	0.360
Cyanobacteria	*Oscillatoriaceae*	*Oscillatoria*	*nigro-viridis*	0.031	0.221	0.047	0.048	-0.017	0.643	-0.072	0.081
Cyanobacteria	*Scytonemataceae*	*Scytonema*	*sp._NIES-4073*	-0.048	0.021	-0.038	0.063	-0.048	0.087	0.033	0.485
Firmicutes	*Bacillaceae*	*Bacillus*	*anthracis*	-0.056	0.084	-0.060	0.030	-0.020	0.629	0.052	0.175
Firmicutes	*Bacillaceae*	*Bacillus*	*cereus*	-0.012	0.698	-0.025	0.300	0.043	0.205	-0.074	0.026
Firmicutes	*Bacillaceae*	*Bacillus*	*megaterium*	0.029	0.132	0.025	0.186	0.054	0.037	-0.008	0.818
Firmicutes	*Bacillaceae*	*Salicibibacter*	*halophilus*	-0.007	0.701	-0.010	0.513	0.000	0.992	0.093	0.032
Firmicutes	*Carnobacteriaceae*	*Carnobacterium*	*maltaromaticum*	0.017	0.328	-0.013	0.412	0.057	0.038	0.069	0.063
Firmicutes	*Carnobacteriaceae*	*Carnobacterium*	*sp._CP1*	0.078	0.004	0.048	0.003	0.055	0.138	-0.035	0.280
Firmicutes	*Enterococcaceae*	*Enterococcus*	*faecalis*	-0.017	0.329	-0.020	0.271	0.064	0.023	0.015	0.623
Firmicutes	*Enterococcaceae*	*Enterococcus*	*faecium*	-0.055	0.112	-0.071	0.031	0.047	0.272	-0.033	0.471
Firmicutes	*Erysipelotrichaceae*	*Erysipelatoclostridium*	*ramosum*	-0.065	0.017	-0.071	3.3E-04	-0.014	0.747	0.052	0.150
Firmicutes	*Lachnospiraceae*	*Anaerobutyricum*	*hallii*	-0.057	0.113	-0.083	0.005	0.039	0.499	-0.038	0.483
Firmicutes	*Lachnospiraceae*	*Blautia*	*producta*	0.002	0.937	0.034	0.224	-0.083	0.047	0.037	0.479
Firmicutes	*Lachnospiraceae*	*Enterocloster*	*clostridioformis*	-0.033	0.247	-0.047	0.036	0.005	0.937	0.058	0.138
Firmicutes	*Lactobacillaceae*	*Lactobacillus*	*delbrueckii*	0.041	0.043	0.030	0.119	0.029	0.360	-0.084	0.010
Firmicutes	*Lactobacillaceae*	*Lactobacillus*	*helveticus*	0.040	0.029	-0.007	0.665	0.101	2.8E-05	-0.026	0.535
Firmicutes	*Lactobacillaceae*	*Lactobacillus*	*sp._3B(2020)*	0.015	0.470	-0.012	0.478	0.067	0.018	-0.065	0.091
Firmicutes	*Lactobacillaceae*	*Limosilactobacillus*	*fermentum*	-0.011	0.614	0.026	0.289	-0.070	0.004	-0.061	0.013
Firmicutes	*Lactobacillaceae*	*Limosilactobacillus*	*Lactobacillus_mucosae*	-0.007	0.812	-0.003	0.910	-0.030	0.416	0.103	0.044
Firmicutes	*Peptoniphilaceae*	*Finegoldia*	*magna*	-0.052	0.105	-0.057	0.034	0.013	0.775	-0.010	0.865
Firmicutes	*Peptostreptococcaceae*	*Flintibacter*	*sp._KGMB00164*	0.041	0.129	0.075	0.002	-0.059	0.140	0.035	0.438
Firmicutes	*Planococcaceae*	*Planococcus*	*sp._MB-3u-03*	-0.062	0.012	-0.052	0.002	-0.037	0.353	0.020	0.477
Firmicutes	*Staphylococcaceae*	*Auricoccus*	*indicus*	0.007	0.709	-0.014	0.437	0.056	0.012	-0.003	0.932
Firmicutes	*Staphylococcaceae*	*Salinicoccus*	*halodurans*	0.019	0.423	-0.008	0.747	0.100	0.012	-0.007	0.885
Firmicutes	*Staphylococcaceae*	*Staphylococcus*	*caprae*	-0.042	0.046	-0.036	0.068	-0.014	0.715	0.001	0.970
Firmicutes	*Staphylococcaceae*	*Staphylococcus*	*cohnii*	0.037	0.060	0.015	0.356	0.074	0.019	0.005	0.905
Firmicutes	*Staphylococcaceae*	*Staphylococcus*	*condimenti*	0.021	0.220	0.012	0.442	0.062	0.045	-0.046	0.299
Firmicutes	*Staphylococcaceae*	*Staphylococcus*	*kloosii*	-0.031	0.272	-0.013	0.485	-0.084	0.162	0.064	0.018
Firmicutes	*Staphylococcaceae*	*Staphylococcus*	*pettenkoferi*	-0.064	2.3E-04	-0.055	1.5E-04	-0.032	0.346	0.005	0.892
Firmicutes	*Staphylococcaceae*	*Staphylococcus*	*simulans*	-0.035	0.043	-0.044	0.001	0.048	0.027	0.005	0.876
Firmicutes	*Staphylococcaceae*	*Staphylococcus*	*succinus*	0.026	0.203	0.048	0.041	-0.025	0.428	0.005	0.900
Firmicutes	*Staphylococcaceae*	*Staphylococcus*	*vitulinus*	-0.014	0.501	0.004	0.840	-0.075	0.033	-0.007	0.809
Firmicutes	*Streptococcaceae*	*Streptococcus*	*equinus*	0.053	0.002	0.032	0.040	0.016	0.535	0.031	0.262
Firmicutes	*Streptococcaceae*	*Streptococcus*	*gordonii*	-0.065	0.011	-0.091	7.5E-05	-0.033	0.513	-0.033	0.475
Firmicutes	*Streptococcaceae*	*Streptococcus*	*gwangjuense*	0.019	0.472	-0.016	0.542	0.089	0.016	0.010	0.786
Firmicutes	*Streptococcaceae*	*Streptococcus*	*pyogenes*	-0.032	0.148	-0.049	0.011	0.016	0.707	0.015	0.734
Firmicutes	*Streptococcaceae*	*Streptococcus*	*sanguinis*	-0.028	0.397	0.004	0.864	-0.125	0.028	0.040	0.279
Firmicutes	*Streptococcaceae*	*Streptococcus*	*sp._116-D4*	-0.071	0.026	-0.065	0.034	-0.050	0.435	0.062	0.438
Firmicutes	*Streptococcaceae*	*Streptococcus*	*thermophilus*	0.031	0.250	0.041	0.040	-0.029	0.468	-0.047	0.181
Firmicutes	*Veillonellaceae*	*Veillonella*	*atypica*	-0.067	0.002	-0.027	0.130	-0.107	0.004	0.028	0.460

Planctomycetes	*Planctomycetaceae*	*Planctomyces*	*sp._SH-PL62*	0.013	0.445	0.020	0.177	0.000	0.992	0.083	0.009
Proteobacteria	*Acetobacteraceae*	*Roseomonas*	*gilardii*	0.050	0.004	0.068	0.033	0.005	0.908	-0.077	0.018
Proteobacteria	*Acetobacteraceae*	*Roseomonas*	*mucosa*	-0.059	0.051	-0.043	0.033	-0.044	0.507	0.024	0.569
Proteobacteria	*Acetobacteraceae*	*Roseomonas*	*sp._FDAARGOS_362*	0.036	0.069	0.040	0.023	-0.031	0.288	0.084	0.046
Proteobacteria	*Alcaligenaceae*	*Achromobacter*	*xylosoxidans*	-0.068	0.001	-0.044	0.019	-0.077	0.011	-0.022	0.701
Proteobacteria	*Alcaligenaceae*	*Alcaligenes*	*faecalis*	-0.007	0.810	-0.031	0.237	0.065	0.012	0.026	0.499
Proteobacteria	*Alcaligenaceae*	*Bordetella*	*parapertussis*	-0.029	0.184	-0.045	0.017	0.029	0.434	-0.032	0.453
Proteobacteria	*Alcaligenaceae*	*Rhizobacter*	*gummiphilus*	-0.048	0.006	-0.033	0.026	-0.033	0.288	0.065	0.057
Proteobacteria	*Alcaligenaceae*	*Rubrivivax*	*gelatinosus*	-0.011	0.583	-0.037	0.039	0.047	0.103	-0.012	0.754
Proteobacteria	*Alcanivoracaceae*	*Alcanivorax*	*sp._N3-2A*	-0.029	0.356	-0.031	0.259	-0.005	0.850	0.096	1.5E-04
Proteobacteria	*Anaplasmataceae*	*Wolbachia*	*pipientis*	0.007	0.584	-0.007	0.547	0.049	0.022	0.010	0.717
Proteobacteria	*Aurantimonadaceae*	*Aureimonas*	*sp._AU20*	0.049	0.120	0.027	0.385	0.024	0.559	-0.127	0.020
Proteobacteria	*Bradyrhizobiaceae*	*Bradyrhizobium*	*erythrophlei*	0.047	0.060	0.071	0.006	-0.027	0.516	-0.086	0.050
Proteobacteria	*Bradyrhizobiaceae*	*Bradyrhizobium*	*icense*	-0.048	0.017	-0.027	0.140	-0.038	0.299	0.037	0.471
Proteobacteria	*Bradyrhizobiaceae*	*Bradyrhizobium*	*paxllaeri*	0.032	0.185	0.003	0.887	0.075	0.022	-0.070	0.117
Proteobacteria	*Bradyrhizobiaceae*	*Variibacter*	*gotjawalensis*	0.011	0.624	-0.018	0.333	0.102	0.001	0.025	0.527
Proteobacteria	*Brucellaceae*	*Brucella*	*intermedia*	0.049	0.030	0.033	0.089	0.025	0.397	-0.006	0.884
Proteobacteria	*Brucellaceae*	*Ochrobactrum*	*pseudogrignonense*	0.041	0.073	0.029	0.130	0.022	0.537	0.088	0.025
Proteobacteria	*Burkholderiaceae*	*Burkholderia*	*stabilis*	0.036	0.028	0.023	0.077	0.021	0.480	0.046	0.205
Proteobacteria	*Caulobacteraceae*	*Brevundimonas*	*sp._DS20*	-0.039	0.057	-0.018	0.341	-0.077	0.040	0.062	0.079
Proteobacteria	*Caulobacteraceae*	*Brevundimonas*	*sp._GW460-12-10-14-LB2*	-0.044	0.129	0.001	0.969	-0.092	0.042	-0.075	0.184
Proteobacteria	*Caulobacteraceae*	*Brevundimonas*	*sp._M20*	0.034	0.047	0.015	0.336	0.057	0.046	0.003	0.925
Proteobacteria	*Caulobacteraceae*	*Brevundimonas*	*sp._scallop*	-0.028	0.345	-0.010	0.712	-0.064	0.181	0.224	1.4E-04
Proteobacteria	*Comamonadaceae*	*Acidovorax*	*sp._KKS102*	-0.004	0.918	-0.023	0.451	0.060	0.028	-0.049	0.109
Proteobacteria	*Comamonadaceae*	*Comamonas*	*aquatica*	-0.029	0.136	-0.034	0.054	0.009	0.730	-0.074	0.046
Proteobacteria	*Comamonadaceae*	*Comamonas*	*kerstersii*	0.045	5.7E-04	0.042	0.002	-0.004	0.912	0.082	0.009
Proteobacteria	*Comamonadaceae*	*Hydrogenophaga*	*sp._NH-16*	-0.054	0.032	-0.047	0.012	-0.067	0.124	0.026	0.441
Proteobacteria	*Enterobacteriaceae*	*Citrobacter*	*portucalensis*	-0.033	0.082	-0.052	0.010	0.070	0.007	-0.044	0.097
Proteobacteria	*Enterobacteriaceae*	*Enterobacter*	*cancerogenus*	-0.016	0.222	-0.024	0.009	0.015	0.619	0.074	0.181
Proteobacteria	*Enterobacteriaceae*	*Enterobacter*	*hormaechei*	0.019	0.349	-0.012	0.547	0.086	0.002	-0.041	0.343
Proteobacteria	*Enterobacteriaceae*	*Klebsiella*	*michiganensis*	0.055	0.070	0.066	0.033	0.014	0.754	-0.006	0.934
Proteobacteria	*Enterobacteriaceae*	*Klebsiella*	*pneumoniae*	0.020	0.436	0.023	0.309	-0.006	0.862	-0.118	0.014
Proteobacteria	*Enterobacteriaceae*	*Kosakonia*	*sp._CCTCC_M2018092*	0.037	0.163	-0.002	0.931	0.109	0.035	-0.032	0.483
Proteobacteria	*Enterobacteriaceae*	*Leclercia*	*adecarboxylata*	-0.020	0.501	-0.039	0.172	0.086	0.035	-0.068	0.185
Proteobacteria	*Enterobacteriaceae*	*Leclercia*	*sp._29361*	0.046	0.296	-0.009	0.840	0.134	0.012	-0.060	0.284
Proteobacteria	*Enterobacteriaceae*	*Lelliottia*	*amnigena*	-0.004	0.894	-0.026	0.378	0.051	0.186	-0.086	0.036
Proteobacteria	*Enterobacteriaceae*	*Pseudescherichia*	*vulneris*	0.061	0.024	0.061	0.027	0.010	0.813	-0.022	0.607
Proteobacteria	*Erwiniaceae*	*Pantoea*	*ananatis*	0.024	0.343	0.026	0.248	-0.010	0.789	-0.115	0.005
Proteobacteria	*Erwiniaceae*	*Pantoea*	*sp._SO10*	-0.058	0.036	-0.028	0.190	-0.097	0.027	0.103	0.017
Proteobacteria	*Hyphomicrobiaceae*	*Devosia*	*sp._S02*	0.056	0.006	0.067	4.6E-04	-0.004	0.907	0.036	0.393
Proteobacteria	*Methylobacteriaceae*	*Methylobacterium*	*sp._AMS5*	-0.066	0.043	-0.054	0.146	-0.005	0.909	0.040	0.399
Proteobacteria	*Methylobacteriaceae*	*Methylorubrum*	*populi*	0.042	0.021	0.045	0.013	0.027	0.455	-0.010	0.783
Proteobacteria	*Moraxellaceae*	*Acinetobacter*	*bereziniae*	0.016	0.261	0.002	0.855	0.063	0.015	0.040	0.216
Proteobacteria	*Moraxellaceae*	*Acinetobacter*	*dispersus*	0.026	0.159	0.016	0.412	0.049	0.035	0.004	0.911
Proteobacteria	*Moraxellaceae*	*Acinetobacter*	*junii*	-0.015	0.490	-0.003	0.920	-0.007	0.805	-0.080	0.008
Proteobacteria	*Moraxellaceae*	*Acinetobacter*	*piscicola*	-0.020	0.241	0.002	0.881	-0.052	0.049	-0.021	0.452
Proteobacteria	*Moraxellaceae*	*Acinetobacter*	*soli*	-0.034	0.084	-0.049	0.002	0.050	0.092	-0.077	0.003
Proteobacteria	*Moraxellaceae*	*Acinetobacter*	*sp._10FS3-1*	-0.057	0.018	-0.039	0.106	0.006	0.850	0.025	0.501
Proteobacteria	*Moraxellaceae*	*Acinetobacter*	*sp._FDAARGOS_724*	-0.014	0.523	-0.024	0.257	0.061	0.004	0.020	0.541
Proteobacteria	*Moraxellaceae*	*Acinetobacter*	*sp._NEB149*	0.027	0.299	-0.011	0.697	0.121	0.001	-0.049	0.302

Proteobacteria	*Moraxellaceae*	*Acinetobacter*	*sp._WCHA45*	-0.004	0.838	-0.008	0.626	0.045	0.039	-0.011	0.557
Proteobacteria	*Moraxellaceae*	*Acinetobacter*	*sp._WCHA55*	-0.040	0.072	-0.026	0.209	-0.026	0.434	-0.113	0.002
Proteobacteria	*Moraxellaceae*	*Acinetobacter*	*sp._YH12138*	-0.047	0.057	-0.057	0.036	0.027	0.348	0.013	0.694
Proteobacteria	*Moraxellaceae*	*Acinetobacter*	*venetianus*	-0.047	0.023	-0.054	0.005	0.019	0.539	-0.081	0.007
Proteobacteria	*Moraxellaceae*	*Moraxella*	*osloensis*	0.009	0.693	-0.017	0.397	0.047	0.046	-0.004	0.895
Proteobacteria	*Moraxellaceae*	*Psychrobacter*	*alimentarius*	-0.003	0.932	-0.037	0.242	0.085	0.046	-0.079	0.206
Proteobacteria	*Moraxellaceae*	*Psychrobacter*	*cryohalolentis*	-0.043	0.102	-0.046	0.093	0.040	0.451	0.111	0.017
Proteobacteria	*Moraxellaceae*	*Psychrobacter*	*sp._DAB_AL43B*	0.071	0.071	0.070	0.047	-0.012	0.835	-0.069	0.287
Proteobacteria	*Moraxellaceae*	*Psychrobacter*	*sp._P11G5*	0.107	0.006	0.100	0.011	0.042	0.371	-0.020	0.738
Proteobacteria	*Moraxellaceae*	*Psychrobacter*	*urativorans*	0.035	0.024	0.026	0.117	0.030	0.204	-0.031	0.472
Proteobacteria	*Morganellaceae*	*Proteus*	*vulgaris*	0.023	0.123	-0.006	0.695	0.067	0.004	-0.028	0.423
Proteobacteria	*Neisseriaceae*	*Neisseria*	*elongata*	0.016	0.483	-0.015	0.442	0.078	0.018	-0.001	0.981
Proteobacteria	*Neisseriaceae*	*Neisseria*	*flavescens*	0.067	0.037	0.036	0.220	0.091	0.041	0.036	0.497
Proteobacteria	*Oxalobacteraceae*	*Duganella*	*sp._AF9R3*	-0.051	0.145	-0.032	0.284	-0.037	0.512	-0.108	0.033
Proteobacteria	*Oxalobacteraceae*	*Massilia*	*albidiflava*	-0.068	0.005	-0.059	0.002	-0.048	0.251	-0.065	0.086
Proteobacteria	*Oxalobacteraceae*	*Massilia*	*sp._LPB0304*	0.030	0.257	-0.009	0.710	0.086	0.021	-0.015	0.724
Proteobacteria	*Oxalobacteraceae*	*Massilia*	*sp._YMA4*	-0.028	0.149	-0.035	0.033	-0.010	0.761	0.060	0.133
Proteobacteria	*Pasteurellaceae*	*Frederiksenia*	*canicola*	0.015	0.609	0.004	0.926	0.054	0.372	-0.187	6.8E-04
Proteobacteria	*Pasteurellaceae*	*Haemophilus*	*parahaemolyticus*	0.034	0.113	0.017	0.381	0.059	0.031	-0.028	0.489
Proteobacteria	*Pasteurellaceae*	*Haemophilus*	*parainfluenzae*	0.084	0.003	0.098	3.4E-04	-0.015	0.682	-0.044	0.416
Proteobacteria	*Pasteurellaceae*	*Pasteurella*	*multocida*	-0.026	0.256	-0.042	0.048	-0.001	0.978	-0.037	0.393
Proteobacteria	*Pseudomonadaceae*	*Pseudomonas*	*aeruginosa*	-0.038	0.157	0.006	0.788	-0.105	0.008	0.094	0.039
Proteobacteria	*Pseudomonadaceae*	*Pseudomonas*	*entomophila*	-0.014	0.536	-0.024	0.131	0.054	0.288	0.109	0.018
Proteobacteria	*Pseudomonadaceae*	*Pseudomonas*	*parafulva*	-0.039	0.091	-0.047	0.013	-0.001	0.991	-0.011	0.746
Proteobacteria	*Pseudomonadaceae*	*Pseudomonas*	*poae*	0.030	0.089	0.036	0.031	-0.003	0.929	0.046	0.166
Proteobacteria	*Pseudomonadaceae*	*Pseudomonas*	*psychrophila*	-0.023	0.121	-0.033	0.005	0.034	0.133	0.002	0.929
Proteobacteria	*Pseudomonadaceae*	*Pseudomonas*	*psychrotolerans*	-0.042	0.129	0.000	0.998	-0.119	0.003	-0.034	0.413
Proteobacteria	*Pseudomonadaceae*	*Pseudomonas*	*putida*	0.016	0.645	-0.003	0.915	0.055	0.236	-0.175	0.010
Proteobacteria	*Pseudomonadaceae*	*Pseudomonas*	*simiae*	-0.040	0.091	-0.012	0.614	-0.080	0.048	0.082	0.041
Proteobacteria	*Pseudomonadaceae*	*Pseudomonas*	*sp._C27(2019)*	0.024	0.257	0.028	0.169	-0.011	0.762	-0.080	0.033
Proteobacteria	*Pseudomonadaceae*	*Pseudomonas*	*sp._gcc21*	-0.020	0.256	-0.009	0.646	-0.040	0.052	0.100	0.011
Proteobacteria	*Pseudomonadaceae*	*Pseudomonas*	*sp._phDV1*	0.028	0.318	0.035	0.187	-0.012	0.775	-0.091	0.038
Proteobacteria	*Pseudomonadaceae*	*Pseudomonas*	*sp._TUM18999*	-0.039	0.029	-0.031	0.017	0.009	0.715	0.009	0.718
Proteobacteria	*Pseudomonadaceae*	*Pseudomonas*	*synxantha*	-0.019	0.322	0.007	0.750	-0.051	3.5E-04	-0.028	0.453
Proteobacteria	*Pseudomonadaceae*	*Pseudomonas*	*syringae_group_genomosp._3*	-0.024	0.213	0.001	0.953	-0.062	0.038	0.011	0.756
Proteobacteria	*Pseudomonadaceae*	*Pseudomonas*	*viridiflava*	0.045	0.021	0.040	0.014	0.007	0.739	0.015	0.660
Proteobacteria	*Rhizobiaceae*	*Shinella*	*sp._HZN7*	0.014	0.551	-0.019	0.363	0.075	0.044	-0.034	0.435
Proteobacteria	*Rhodobacteraceae*	*Pannonibacter*	*phragmitetus*	0.007	0.738	0.014	0.372	-0.018	0.655	0.076	0.014
Proteobacteria	*Rickettsiaceae*	*Rickettsia*	*bellii*	-0.017	0.737	0.039	0.567	-0.220	0.007	0.066	0.364
Proteobacteria	*Sphingomonadaceae*	*Sphingomonas*	*daechungensis*	-0.074	0.001	-0.066	0.002	-0.046	0.129	-0.006	0.868
Proteobacteria	*Sphingomonadaceae*	*Sphingomonas*	*indica*	0.028	0.097	0.005	0.741	0.084	4.6E-04	-0.041	0.265
Proteobacteria	*Sphingomonadaceae*	*Sphingomonas*	*insulae*	-0.045	0.063	-0.046	0.038	-0.019	0.613	-0.118	0.003
Proteobacteria	*Sphingomonadaceae*	*Sphingomonas*	*sp._AAP5*	0.043	0.028	0.040	0.029	-0.008	0.800	-0.027	0.483
Proteobacteria	*Sphingomonadaceae*	*Sphingomonas*	*sp._MM-1*	0.036	0.082	0.039	0.018	0.004	0.881	0.024	0.476
Proteobacteria	*Sphingomonadaceae*	*Sphingosinithalassobacter*	*sp._CS137*	-0.040	0.029	-0.029	0.084	-0.038	0.251	0.035	0.358
Proteobacteria	*Xanthomonadaceae*	*Luteimonas*	*chenhongjianii*	0.070	0.003	0.042	0.053	0.045	0.141	0.050	0.180
Proteobacteria	*Xanthomonadaceae*	*Lysobacter*	*enzymogenes*	0.018	0.210	-0.001	0.959	0.032	0.156	0.087	0.003
Proteobacteria	*Xanthomonadaceae*	*Stenotrophomonas*	*acidaminiphila*	0.054	0.125	0.057	0.105	0.018	0.722	0.142	0.028
Proteobacteria	*Xanthomonadaceae*	*Stenotrophomonas*	*maltophilia*	-0.008	0.707	0.008	0.692	-0.041	0.163	0.133	1.2E-04
Proteobacteria	*Xanthomonadaceae*	*Stenotrophomonas*	*sp._169*	0.027	0.276	0.021	0.261	0.027	0.395	0.064	0.046

Proteobacteria	*Xanthomonadaceae*	*Stenotrophomonas*	*sp._LM091*	-0.017	0.552	-0.002	0.936	-0.038	0.365	0.088	0.002
Proteobacteria	*Xanthomonadaceae*	*Thermomonas*	*sp._XSG*	-0.011	0.576	0.021	0.251	-0.062	0.035	0.032	0.348
Proteobacteria	*Xanthomonadaceae*	*Xanthomonas*	*arboricola*	-0.070	6.3E-05	-0.038	0.032	-0.076	0.020	0.059	0.068
Proteobacteria	*Yersiniaceae*	*Ewingella*	*americana*	0.036	0.410	0.070	0.018	-0.057	0.445	0.032	0.519
Proteobacteria	*Yersiniaceae*	*Rouxiella*	*badensis*	0.072	0.126	0.084	0.073	-0.023	0.695	0.179	0.038
Proteobacteria	*Yersiniaceae*	*Serratia*	*liquefaciens*	0.022	0.252	0.042	0.057	-0.035	0.297	-0.067	0.013
Proteobacteria	*Yersiniaceae*	*Serratia*	*symbiotica*	0.011	0.750	0.054	0.021	-0.106	0.031	0.069	0.155

Table S8. Species level association results for the 76 genera related to lung function parameters (p-value<0.05) in the genus level differential abundance analysis results.

				FEV_1		FVC		FEV_1/FVC	
Phylum	Genus	N species	Species	Coef	P-value	Coef	P-value	Coef	P-value
Acidobacteria	*Luteitalea*	1	*pratensis*	-0.058	0.010	-0.023	0.232	-0.082	0.017
Actinobacteria	*Ilumatobacter*	1	*coccineus*	0.061	0.003	0.048	0.016	0.028	0.318
Actinobacteria	*Pauljensenia*	1	*hongkongensis*	0.026	0.186	0.009	0.576	0.066	0.029
Actinobacteria	*Bifidobacterium*	11	*adolescentis*	0.007	0.807	0.004	0.881	0.020	0.323
Actinobacteria	*Bifidobacterium*	11	*bifidum*	0.039	0.044	0.027	0.168	0.051	0.040
Actinobacteria	*Bifidobacterium*	11	*breve*	0.026	0.220	0.025	0.131	0.001	0.981
Actinobacteria	*Bifidobacterium*	11	*catenulatum*	0.005	0.835	-0.016	0.445	0.062	0.040
Actinobacteria	*Bifidobacterium*	11	*choerinum*	-0.013	0.550	0.009	0.638	-0.048	0.145
Actinobacteria	*Bifidobacterium*	11	*dentium*	0.008	0.694	0.000	0.994	0.003	0.917
Actinobacteria	*Bifidobacterium*	11	*longum*	-0.041	0.010	-0.041	0.007	0.018	0.450
Actinobacteria	*Bifidobacterium*	11	*pseudocatenulatum*	-0.056	0.011	-0.066	0.001	0.004	0.912
Actinobacteria	*Bifidobacterium*	11	*pseudolongum*	-0.005	0.912	-0.008	0.842	-0.012	0.808
Actinobacteria	*Bifidobacterium*	11	*pullorum*	-0.010	0.783	0.028	0.426	-0.040	0.489
Actinobacteria	*Bifidobacterium*	11	*thermophilum*	0.039	0.322	0.022	0.522	0.028	0.417
Actinobacteria	*Parascardovia*	1	*denticolens*	-0.060	0.007	-0.030	0.154	-0.103	0.004
Actinobacteria	*Cellulomonas*	9	*fimi*	-0.045	0.037	-0.040	0.028	-0.007	0.820
Actinobacteria	*Cellulomonas*	9	*flavigena*	-0.009	0.733	-0.017	0.497	0.025	0.544
Actinobacteria	*Cellulomonas*	9	*gilvus*	-0.032	0.267	-0.029	0.250	-0.022	0.449
Actinobacteria	*Cellulomonas*	9	*shaoxiangyii*	-0.029	0.172	-0.027	0.208	0.020	0.560
Actinobacteria	*Cellulomonas*	9	*sp._40-2*	-0.024	0.214	-0.014	0.347	-0.043	0.364
Actinobacteria	*Cellulomonas*	9	*sp._H30R-01*	-0.007	0.783	-0.003	0.886	0.014	0.720
Actinobacteria	*Cellulomonas*	9	*sp._JZ18*	-0.005	0.833	-0.018	0.344	0.007	0.860
Actinobacteria	*Cellulomonas*	9	*sp._PSBB021*	0.007	0.690	-0.002	0.929	0.021	0.476
Actinobacteria	*Cellulomonas*	9	*sp._Y8*	-0.018	0.547	-0.017	0.515	-0.008	0.838
Actinobacteria	*Kytococcus*	1	*sedentarius*	0.044	0.060	0.040	0.098	0.026	0.470
Actinobacteria	*Ornithinimicrobium*	5	*flavum*	-0.022	0.647	0.015	0.793	-0.079	0.114
Actinobacteria	*Ornithinimicrobium*	5	*pratense*	-0.014	0.597	0.017	0.422	-0.082	0.088
Actinobacteria	*Ornithinimicrobium*	5	*sp._AMA3305*	0.018	0.602	0.020	0.553	-0.023	0.583
Actinobacteria	*Ornithinimicrobium*	5	*sp._H23M54*	0.007	0.784	0.018	0.359	-0.043	0.335
Actinobacteria	*Ornithinimicrobium*	5	*sp._HY006*	0.033	0.217	0.017	0.462	0.011	0.783
Actinobacteria	*Phycicoccus*	3	*dokdonensis*	-0.013	0.678	-0.039	0.202	0.083	0.053
Actinobacteria	*Phycicoccus*	3	*endophyticus*	0.026	0.155	0.015	0.402	0.037	0.222
Actinobacteria	*Phycicoccus*	3	*sp._HDW14*	-0.013	0.681	-0.019	0.515	0.034	0.472
Actinobacteria	*Cnuibacter*	1	*physcomitrellae*	0.006	0.831	-0.005	0.838	0.049	0.152
Actinobacteria	*Cryobacterium*	3	*arcticum*	0.028	0.274	0.028	0.203	-0.026	0.449
Actinobacteria	*Cryobacterium*	3	*soli*	0.015	0.467	-0.017	0.359	0.068	0.034
Actinobacteria	*Cryobacterium*	3	*sp._LW097*	0.024	0.447	0.032	0.228	-0.019	0.648
Actinobacteria	*Frigoribacterium*	1	*sp._NBH87*	0.036	0.206	0.042	0.113	0.012	0.783
Actinobacteria	*Herbiconiux*	1	*sp._SALV-R1*	-0.018	0.317	0.012	0.450	-0.050	0.066
Actinobacteria	*Microbacterium*	32	*aurum*	0.033	0.131	0.014	0.432	0.055	0.141
Actinobacteria	*Microbacterium*	32	*chocolatum*	0.016	0.399	0.021	0.304	0.008	0.772
Actinobacteria	*Microbacterium*	32	*esteraromaticum*	0.019	0.313	0.017	0.307	0.009	0.778
Actinobacteria	*Microbacterium*	32	*foliorum*	-0.015	0.581	-0.025	0.314	0.007	0.854
Actinobacteria	*Microbacterium*	32	*hominis*	-0.021	0.425	-0.013	0.589	-0.037	0.333
Actinobacteria	*Microbacterium*	32	*lemovicicum*	0.011	0.694	-0.013	0.597	0.034	0.420
Actinobacteria	*Microbacterium*	32	*oleivorans*	0.040	0.121	0.037	0.096	0.007	0.868

Actinobacteria	*Microbacterium*	32	*oryzae*	0.002	0.934	-0.012	0.479	0.036	0.295
Actinobacteria	*Microbacterium*	32	*oxydans*	0.058	0.052	0.037	0.126	0.071	0.104
Actinobacteria	*Microbacterium*	32	*paraoxydans*	0.031	0.124	0.024	0.180	0.018	0.458
Actinobacteria	*Microbacterium*	32	*pygmaeum*	-0.019	0.477	-0.013	0.583	-0.016	0.675
Actinobacteria	*Microbacterium*	32	*sediminis*	-0.009	0.596	-0.020	0.221	0.010	0.666
Actinobacteria	*Microbacterium*	32	*sp._10M-3C3*	-0.013	0.485	-0.019	0.277	0.006	0.831
Actinobacteria	*Microbacterium*	32	*sp._4R-513*	0.008	0.715	0.002	0.926	0.024	0.398
Actinobacteria	*Microbacterium*	32	*sp._A18JL241*	0.010	0.666	0.024	0.280	-0.027	0.464
Actinobacteria	*Microbacterium*	32	*sp._ABRD_28*	0.020	0.346	0.004	0.846	0.059	0.051
Actinobacteria	*Microbacterium*	32	*sp._BH-3-3-3*	-0.048	0.157	-0.011	0.673	-0.113	0.052
Actinobacteria	*Microbacterium*	32	*sp._CBA3102*	0.015	0.393	0.023	0.172	0.000	0.990
Actinobacteria	*Microbacterium*	32	*sp._HY82*	-0.060	0.020	-0.042	0.076	-0.066	0.112
Actinobacteria	*Microbacterium*	32	*sp._LKL04*	-0.024	0.447	-0.024	0.398	0.028	0.556
Actinobacteria	*Microbacterium*	32	*sp._No._7*	-0.023	0.354	-0.034	0.141	0.014	0.643
Actinobacteria	*Microbacterium*	32	*sp._Nx66*	0.045	0.063	0.011	0.597	0.078	0.011
Actinobacteria	*Microbacterium*	32	*sp._NY27*	0.024	0.199	0.013	0.422	0.033	0.161
Actinobacteria	*Microbacterium*	32	*sp._PAMC_28756*	0.013	0.672	-0.003	0.901	0.036	0.423
Actinobacteria	*Microbacterium*	32	*sp._PM5*	0.008	0.711	-0.016	0.450	0.029	0.409
Actinobacteria	*Microbacterium*	32	*sp._RG1*	-0.005	0.845	-0.003	0.877	0.000	0.999
Actinobacteria	*Microbacterium*	32	*sp._TPU_3598*	-0.007	0.690	-0.025	0.122	0.025	0.371
Actinobacteria	*Microbacterium*	32	*sp._WY121*	-0.003	0.855	-0.004	0.809	0.007	0.771
Actinobacteria	*Microbacterium*	32	*sp._XT11*	0.005	0.767	-0.007	0.688	0.047	0.071
Actinobacteria	*Microbacterium*	32	*sp._Y-01*	0.018	0.404	0.016	0.407	0.003	0.927
Actinobacteria	*Microbacterium*	32	*sp._YJN-G*	-0.021	0.369	-0.004	0.853	-0.029	0.401
Actinobacteria	*Microbacterium*	32	*testaceum*	0.043	0.147	0.043	0.122	0.037	0.405
Actinobacteria	*Rathayibacter*	8	*festucae*	-0.010	0.710	0.005	0.846	-0.053	0.259
Actinobacteria	*Rathayibacter*	8	*sp._VKM_Ac-2759*	0.024	0.170	0.016	0.326	0.008	0.786
Actinobacteria	*Rathayibacter*	8	*sp._VKM_Ac-2760*	0.033	0.073	0.015	0.336	0.026	0.434
Actinobacteria	*Rathayibacter*	8	*sp._VKM_Ac-2762*	0.007	0.706	-0.005	0.784	0.020	0.561
Actinobacteria	*Rathayibacter*	8	*sp._VKM_Ac-2801*	0.050	0.193	0.024	0.496	0.106	0.052
Actinobacteria	*Rathayibacter*	8	*sp._VKM_Ac-2804*	0.020	0.319	0.023	0.215	-0.012	0.699
Actinobacteria	*Rathayibacter*	8	*sp._VKM_Ac-2805*	0.007	0.753	0.009	0.657	-0.023	0.507
Actinobacteria	*Rathayibacter*	8	*toxicus*	-0.036	0.420	-0.025	0.578	-0.013	0.869
Actinobacteria	*Arthrobacter*	6	*agilis*	0.028	0.329	0.024	0.328	0.009	0.786
Actinobacteria	*Arthrobacter*	6	*alpinus*	0.002	0.930	0.020	0.405	-0.038	0.294
Actinobacteria	*Arthrobacter*	6	*crystallopoietes*	-0.026	0.181	-0.016	0.363	-0.023	0.320
Actinobacteria	*Arthrobacter*	6	*sp._NEB_688*	-0.020	0.593	-0.009	0.808	-0.014	0.764
Actinobacteria	*Arthrobacter*	6	*sp._U41*	0.010	0.672	-0.006	0.753	0.058	0.097
Actinobacteria	*Arthrobacter*	6	*sp._YC-RL1*	-0.040	0.089	-0.052	0.031	0.076	0.018
Actinobacteria	*Kocuria*	10	*flava*	0.003	0.887	0.021	0.336	-0.057	0.183
Actinobacteria	*Kocuria*	10	*indica*	-0.025	0.530	-0.025	0.445	0.013	0.811
Actinobacteria	*Kocuria*	10	*palustris*	0.055	0.014	0.052	0.019	0.005	0.844
Actinobacteria	*Kocuria*	10	*rhizophila*	0.018	0.548	0.002	0.942	0.060	0.236
Actinobacteria	*Kocuria*	10	*rosea*	0.018	0.634	0.054	0.158	-0.054	0.204
Actinobacteria	*Kocuria*	10	*sp._BT304*	0.002	0.958	0.019	0.485	-0.072	0.122
Actinobacteria	*Kocuria*	10	*sp._KD4*	0.027	0.340	0.018	0.480	0.024	0.503
Actinobacteria	*Kocuria*	10	*sp._TGY1120_3*	0.018	0.354	0.022	0.198	-0.022	0.483
Actinobacteria	*Kocuria*	10	*turfanensis*	0.019	0.411	0.009	0.673	0.029	0.365
Actinobacteria	*Kocuria*	10	*varians*	0.010	0.570	0.001	0.964	0.018	0.550
Actinobacteria	*Haloactinobacterium*	1	*sp._HY164*	-0.023	0.257	-0.002	0.907	-0.067	0.016

Actinobacteria	*Micromonospora*	5	*echinofusca*	0.011	0.652	0.006	0.787	-0.014	0.664
Actinobacteria	*Micromonospora*	5	*sagamiensis*	0.021	0.447	0.007	0.774	0.032	0.421
Actinobacteria	*Micromonospora*	5	*siamensis*	0.034	0.103	0.032	0.090	0.006	0.850
Actinobacteria	*Micromonospora*	5	*tulbaghiae*	0.028	0.260	0.022	0.356	0.001	0.966
Actinobacteria	*Micromonospora*	5	*zamorensis*	0.056	0.007	0.022	0.260	0.070	0.003
Actinobacteria	*Friedmanniella*	2	*luteola*	0.021	0.387	-0.013	0.600	0.076	0.016
Actinobacteria	*Friedmanniella*	2	*sagamiharensis*	0.017	0.602	-0.023	0.465	0.122	0.006
Actinobacteria	*Cutibacterium*	3	*acnes*	0.021	0.572	0.032	0.303	-0.059	0.216
Actinobacteria	*Cutibacterium*	3	*avidum*	-0.012	0.586	-0.011	0.539	-0.002	0.956
Actinobacteria	*Cutibacterium*	3	*granulosum*	-0.043	0.082	-0.016	0.378	-0.066	0.170
Actinobacteria	*Pseudopropionibacterium*	1	*propionicum*	0.022	0.183	0.016	0.286	0.052	0.089
Actinobacteria	*Nocardiopsis*	2	*alba*	0.026	0.201	0.031	0.103	-0.021	0.500
Actinobacteria	*Nocardiopsis*	2	*dassonvillei*	0.012	0.480	0.027	0.135	-0.024	0.176
Actinobacteria	*Streptosporangium*	1	*sp._caverna*	0.026	0.124	0.013	0.473	0.050	0.062
Proteobacteria	*Variibacter*	1	*gotjawalensis*	0.011	0.624	-0.018	0.333	0.102	0.001
Proteobacteria	*Ochrobactrum*	3	*anthropi*	-0.037	0.305	-0.010	0.742	-0.072	0.255
Proteobacteria	*Ochrobactrum*	3	*pituitosum*	-0.016	0.520	0.000	0.995	-0.016	0.708
Proteobacteria	*Ochrobactrum*	3	*pseudogrignonense*	0.041	0.073	0.029	0.130	0.022	0.537
Proteobacteria	*Devosia*	5	*ginsengisoli*	0.039	0.065	0.022	0.253	0.022	0.460
Proteobacteria	*Devosia*	5	*sp._1566*	0.027	0.136	0.009	0.638	0.038	0.144
Proteobacteria	*Devosia*	5	*sp._A16*	-0.023	0.302	-0.012	0.530	-0.021	0.537
Proteobacteria	*Devosia*	5	*sp._I507*	0.010	0.706	0.007	0.758	0.002	0.940
Proteobacteria	*Devosia*	5	*sp._S02*	0.056	0.006	0.067	4.6E-04	-0.004	0.907
Proteobacteria	*Methylorubrum*	2	*extorquens*	0.028	0.275	0.024	0.303	-0.002	0.951
Proteobacteria	*Methylorubrum*	2	*populi*	0.042	0.021	0.045	0.013	0.027	0.455
Proteobacteria	*Shinella*	1	*sp._HZN7*	0.014	0.551	-0.019	0.363	0.075	0.044
Proteobacteria	*Haematobacter*	1	*massiliensis*	-0.016	0.396	-0.018	0.295	-0.006	0.876
Proteobacteria	*Tabrizicola*	1	*piscis*	-0.004	0.895	-0.029	0.283	0.083	0.114
Proteobacteria	*Wolbachia*	2	*endosymbiont_of_Ctenocephalides_felis_wCfeT*	-0.028	0.184	-0.016	0.427	-0.013	0.724
Proteobacteria	*Wolbachia*	2	*pipientis*	0.007	0.584	-0.007	0.547	0.049	0.022
Proteobacteria	*Sphingosinithalassobacter*	1	*sp._CS137*	-0.040	0.029	-0.029	0.084	-0.038	0.251
Firmicutes	*Saccharibacillus*	1	*brassicae*	0.023	0.215	0.019	0.228	-0.001	0.962
Firmicutes	*Planococcus*	3	*antarcticus*	0.028	0.195	0.015	0.517	0.013	0.637
Firmicutes	*Planococcus*	3	*rifietoensis*	0.020	0.406	-0.001	0.979	0.053	0.137
Firmicutes	*Planococcus*	3	*sp._MB-3u-03*	-0.062	0.012	-0.052	0.002	-0.037	0.353
Firmicutes	*Auricoccus*	1	*indicus*	0.007	0.709	-0.014	0.437	0.056	0.012
Firmicutes	*Enterococcus*	6	*casseliflavus*	-0.009	0.693	0.011	0.579	-0.004	0.917
Firmicutes	*Enterococcus*	6	*cecorum*	-0.046	0.167	-0.033	0.272	0.055	0.364
Firmicutes	*Enterococcus*	6	*faecalis*	-0.017	0.329	-0.020	0.271	0.064	0.023
Firmicutes	*Enterococcus*	6	*faecium*	-0.055	0.112	-0.071	0.031	0.047	0.272
Firmicutes	*Enterococcus*	6	*hirae*	0.005	0.834	-0.006	0.801	0.019	0.477
Firmicutes	*Enterococcus*	6	*mundtii*	0.006	0.815	-0.011	0.674	0.020	0.602
Firmicutes	*Limosilactobacillus*	4	*fermentum*	-0.011	0.614	0.026	0.289	-0.070	0.004
Firmicutes	*Limosilactobacillus*	4	*Lactobacillus_mucosae*	-0.007	0.812	-0.003	0.910	-0.030	0.416
Firmicutes	*Limosilactobacillus*	4	*Lactobacillus_vaginalis*	-0.004	0.866	0.012	0.573	-0.035	0.346
Firmicutes	*Limosilactobacillus*	4	*reuteri*	-0.031	0.476	0.002	0.952	-0.024	0.719
Firmicutes	*Lactococcus*	3	*garvieae*	0.000	0.992	-0.004	0.822	-0.007	0.802
Firmicutes	*Lactococcus*	3	*lactis*	0.021	0.417	0.038	0.072	-0.004	0.914
Firmicutes	*Lactococcus*	3	*raffinolactis*	-0.072	0.177	-0.079	0.093	-0.043	0.537
Firmicutes	*Streptococcus*	30	*agalactiae*	-0.016	0.460	-0.023	0.272	-0.030	0.389

Firmicutes	*Streptococcus*	30	*anginosus*	-0.017	0.362	-0.014	0.421	-0.019	0.612
Firmicutes	*Streptococcus*	30	*australis*	-0.017	0.523	-0.014	0.496	-0.044	0.354
Firmicutes	*Streptococcus*	30	*canis*	0.018	0.712	-0.014	0.768	0.098	0.279
Firmicutes	*Streptococcus*	30	*cristatus*	-0.017	0.421	-0.032	0.123	0.021	0.525
Firmicutes	*Streptococcus*	30	*equinus*	0.053	0.002	0.032	0.040	0.016	0.535
Firmicutes	*Streptococcus*	30	*gordonii*	-0.065	0.011	-0.091	7.5E-05	-0.033	0.513
Firmicutes	*Streptococcus*	30	*gwangjuense*	0.019	0.472	-0.016	0.542	0.089	0.016
Firmicutes	*Streptococcus*	30	*intermedius*	0.021	0.353	0.032	0.126	-0.006	0.852
Firmicutes	*Streptococcus*	30	*lutetiensis*	-0.019	0.595	0.003	0.845	-0.039	0.539
Firmicutes	*Streptococcus*	30	*mitis*	-0.039	0.415	-0.030	0.475	-0.043	0.588
Firmicutes	*Streptococcus*	30	*mutans*	-0.020	0.317	-0.026	0.115	-0.005	0.907
Firmicutes	*Streptococcus*	30	*oralis*	-0.050	0.202	-0.043	0.223	-0.046	0.570
Firmicutes	*Streptococcus*	30	*parasanguinis*	-0.058	0.166	-0.029	0.472	-0.120	0.097
Firmicutes	*Streptococcus*	30	*pneumoniae*	0.025	0.237	0.034	0.118	-0.029	0.516
Firmicutes	*Streptococcus*	30	*pseudopneumoniae*	0.016	0.637	0.042	0.250	-0.093	0.054
Firmicutes	*Streptococcus*	30	*pyogenes*	-0.032	0.148	-0.049	0.011	0.016	0.707
Firmicutes	*Streptococcus*	30	*salivarius*	0.050	0.133	0.014	0.632	0.043	0.402
Firmicutes	*Streptococcus*	30	*sanguinis*	-0.028	0.397	0.004	0.864	-0.125	0.028
Firmicutes	*Streptococcus*	30	*sobrinus*	-0.001	0.965	0.014	0.569	-0.069	0.172
Firmicutes	*Streptococcus*	30	*sp._116-D4*	-0.071	0.026	-0.065	0.034	-0.050	0.435
Firmicutes	*Streptococcus*	30	*sp._A12*	0.037	0.140	0.040	0.063	0.009	0.823
Firmicutes	*Streptococcus*	30	*sp._HSISM1*	0.048	0.199	0.029	0.396	0.016	0.831
Firmicutes	*Streptococcus*	30	*sp._LPB0220*	-0.020	0.591	-0.035	0.281	0.004	0.950
Firmicutes	*Streptococcus*	30	*sp._oral_taxon_061*	0.038	0.291	-0.003	0.930	0.067	0.243
Firmicutes	*Streptococcus*	30	*sp._oral_taxon_064*	0.015	0.587	0.005	0.833	0.015	0.740
Firmicutes	*Streptococcus*	30	*sp._oral_taxon_431*	0.010	0.752	0.019	0.521	-0.007	0.876
Firmicutes	*Streptococcus*	30	*suis*	-0.058	0.139	-0.060	0.118	-0.008	0.779
Firmicutes	*Streptococcus*	30	*thermophilus*	0.031	0.250	0.041	0.040	-0.029	0.468
Firmicutes	*Streptococcus*	30	*vestibularis*	0.022	0.405	0.026	0.333	0.010	0.840
Bacteroidetes	*Alistipes*	7	*communis*	0.043	0.216	0.041	0.184	0.001	0.987
Bacteroidetes	*Alistipes*	7	*dispar*	0.004	0.885	-0.001	0.963	0.040	0.422
Bacteroidetes	*Alistipes*	7	*finegoldii*	0.023	0.195	0.018	0.215	0.046	0.175
Bacteroidetes	*Alistipes*	7	*indistinctus*	-0.009	0.703	-0.003	0.866	-0.005	0.896
Bacteroidetes	*Alistipes*	7	*megaguti*	0.003	0.892	0.013	0.444	-0.047	0.195
Bacteroidetes	*Alistipes*	7	*onderdonkii*	-0.033	0.208	0.003	0.911	-0.085	0.074
Bacteroidetes	*Alistipes*	7	*shahii*	0.000	0.992	-0.012	0.742	-0.007	0.902
Proteobacteria	*Achromobacter*	2	*spanius*	0.018	0.484	0.000	0.993	0.028	0.457
Proteobacteria	*Achromobacter*	2	*xylosoxidans*	-0.068	0.001	-0.044	0.019	-0.077	0.011
Proteobacteria	*Alcaligenes*	1	*faecalis*	-0.007	0.810	-0.031	0.237	0.065	0.012
Proteobacteria	*Rhizobacter*	1	*gummiphilus*	-0.048	0.006	-0.033	0.026	-0.033	0.288
Proteobacteria	*Rubrivivax*	1	*gelatinosus*	-0.011	0.583	-0.037	0.039	0.047	0.103
Firmicutes	*Anaerobutyricum*	1	*hallii*	-0.057	0.113	-0.083	0.005	0.039	0.499
Firmicutes	*Blautia*	4	*argi*	0.046	0.346	0.048	0.271	0.047	0.273
Firmicutes	*Blautia*	4	*hansenii*	0.047	0.297	0.064	0.104	-0.049	0.260
Firmicutes	*Blautia*	4	*producta*	0.002	0.937	0.034	0.224	-0.083	0.047
Firmicutes	*Blautia*	4	*sp._SC05B48*	0.059	0.124	0.048	0.167	0.065	0.283
Firmicutes	*Lachnoclostridium*	3	*Clostridium_scindens*	0.032	0.408	0.004	0.885	0.083	0.144
Firmicutes	*Lachnoclostridium*	3	*phocaeense*	-0.050	0.128	-0.030	0.264	-0.057	0.304
Firmicutes	*Lachnoclostridium*	3	*sp._YL32*	-0.044	0.187	-0.054	0.096	0.012	0.656
Firmicutes	*Clostridioides*	1	*difficile*	0.032	0.451	0.072	0.059	-0.060	0.264

Firmicutes	*Flintibacter*	1	*sp._KGMB00164*	0.041	0.129	0.075	0.002	-0.059	0.140
Firmicutes	*Monoglobus*	1	*pectinilyticus*	0.042	0.065	0.031	0.106	0.039	0.328
Bacteroidetes	*Candidatus_Cardinium*	1	*Cardinium_endosymbiont_of_Sogatella_furcifera*	0.038	0.100	0.010	0.651	0.052	0.037
Bacteroidetes	*Spirosoma*	4	*pollinicola*	0.017	0.624	-0.017	0.585	0.089	0.117
Bacteroidetes	*Spirosoma*	4	*radiotolerans*	0.008	0.785	0.023	0.381	-0.025	0.466
Bacteroidetes	*Spirosoma*	4	*rigui*	0.062	0.022	0.050	0.070	0.031	0.462
Bacteroidetes	*Spirosoma*	4	*sp._CJU-R4*	0.026	0.315	0.022	0.332	0.023	0.502
Deinococcus-Thermus	*Deinococcus*	6	*ficus*	0.000	0.990	-0.010	0.652	0.043	0.243
Deinococcus-Thermus	*Deinococcus*	6	*gobiensis*	-0.015	0.531	-0.011	0.611	0.009	0.792
Deinococcus-Thermus	*Deinococcus*	6	*sp._D7000*	-0.031	0.396	-0.011	0.748	-0.015	0.782
Deinococcus-Thermus	*Deinococcus*	6	*sp._NW-56*	0.008	0.699	0.025	0.163	-0.032	0.224
Deinococcus-Thermus	*Deinococcus*	6	*swuensis*	-0.003	0.935	-0.030	0.372	0.049	0.393
Deinococcus-Thermus	*Deinococcus*	6	*wulumuqiensis*	-0.031	0.143	-0.022	0.288	-0.014	0.592
Firmicutes	*Erysipelatoclostridium*	2	*Clostridium_innocuum*	-0.040	0.269	-0.028	0.385	-0.057	0.220
Firmicutes	*Erysipelatoclostridium*	2	*ramosum*	-0.065	0.017	-0.071	3.3E-04	-0.014	0.747
Bacteroidetes	*Candidatus_Sulcia*	1	*muelleri*	0.034	0.080	0.034	0.057	0.002	0.949
Bacteroidetes	*Elizabethkingia*	1	*miricola*	0.013	0.563	-0.002	0.929	0.051	0.067
Proteobacteria	*Shewanella*	1	*sp._FDAARGOS_354*	0.034	0.057	0.020	0.210	0.036	0.110
Proteobacteria	*Klebsiella*	6	*aerogenes*	0.007	0.789	0.034	0.064	-0.033	0.329
Proteobacteria	*Klebsiella*	6	*michiganensis*	0.055	0.070	0.066	0.033	0.014	0.754
Proteobacteria	*Klebsiella*	6	*oxytoca*	0.009	0.731	0.013	0.547	-0.011	0.776
Proteobacteria	*Klebsiella*	6	*pneumoniae*	0.020	0.436	0.023	0.309	-0.006	0.862
Proteobacteria	*Klebsiella*	6	*quasipneumoniae*	0.009	0.767	0.001	0.958	0.013	0.752
Proteobacteria	*Klebsiella*	6	*variicola*	-0.021	0.324	-0.003	0.843	-0.046	0.278
Proteobacteria	*Leclercia*	2	*adecarboxylata*	-0.020	0.501	-0.039	0.172	0.086	0.035
Proteobacteria	*Leclercia*	2	*sp._29361*	0.046	0.296	-0.009	0.840	0.134	0.012
Proteobacteria	*Lelliottia*	1	*amnigena*	-0.004	0.894	-0.026	0.378	0.051	0.186
Proteobacteria	*Buchnera*	1	*aphidicola*	0.035	0.170	0.015	0.545	0.051	0.054
Proteobacteria	*Ewingella*	1	*americana*	0.036	0.410	0.070	0.018	-0.057	0.445
Proteobacteria	*Halomonas*	1	*hydrothermalis*	0.007	0.742	0.006	0.796	0.035	0.174
Proteobacteria	*Haemophilus*	4	*haemolyticus*	0.003	0.922	0.006	0.823	0.011	0.775
Proteobacteria	*Haemophilus*	4	*influenzae*	0.002	0.936	0.013	0.465	-0.007	0.856
Proteobacteria	*Haemophilus*	4	*parahaemolyticus*	0.034	0.113	0.017	0.381	0.059	0.031
Proteobacteria	*Haemophilus*	4	*parainfluenzae*	0.084	0.003	0.098	3.4E-04	-0.015	0.682
Proteobacteria	*Moraxella*	2	*catarrhalis*	-0.032	0.257	-0.019	0.365	-0.027	0.591
Proteobacteria	*Moraxella*	2	*osloensis*	0.009	0.693	-0.017	0.397	0.047	0.046
Proteobacteria	*Pseudomonas*	68	*aeruginosa*	-0.038	0.157	0.006	0.788	-0.105	0.008
Proteobacteria	*Pseudomonas*	68	*alcaligenes*	-0.008	0.733	-0.017	0.352	0.033	0.226
Proteobacteria	*Pseudomonas*	68	*alcaliphila*	-0.008	0.781	-0.007	0.775	-0.004	0.944
Proteobacteria	*Pseudomonas*	68	*azotoformans*	-0.022	0.562	-0.033	0.306	-0.025	0.579
Proteobacteria	*Pseudomonas*	68	*chlororaphis*	-0.028	0.058	-0.010	0.413	-0.045	0.301
Proteobacteria	*Pseudomonas*	68	*entomophila*	-0.014	0.536	-0.024	0.131	0.054	0.288
Proteobacteria	*Pseudomonas*	68	*extremorientalis*	-0.018	0.586	-0.046	0.175	0.043	0.254
Proteobacteria	*Pseudomonas*	68	*fluorescens*	-0.007	0.668	-0.017	0.241	0.008	0.835
Proteobacteria	*Pseudomonas*	68	*fragi*	0.002	0.926	-0.006	0.811	0.027	0.398
Proteobacteria	*Pseudomonas*	68	*fulva*	-0.005	0.896	0.010	0.728	-0.083	0.108
Proteobacteria	*Pseudomonas*	68	*graminis*	0.001	0.980	0.001	0.982	-0.017	0.636
Proteobacteria	*Pseudomonas*	68	*koreensis*	-0.035	0.335	-0.056	0.065	0.036	0.551
Proteobacteria	*Pseudomonas*	68	*libanensis*	-0.018	0.510	-0.014	0.566	-0.007	0.808
Proteobacteria	*Pseudomonas*	68	*litoralis*	-0.013	0.614	-0.007	0.736	-0.016	0.684

Proteobacteria	*Pseudomonas*	68	*lundensis*	-0.028	0.160	-0.031	0.141	0.029	0.456
Proteobacteria	*Pseudomonas*	68	*lurida*	0.012	0.647	0.010	0.749	0.003	0.962
Proteobacteria	*Pseudomonas*	68	*luteola*	0.173	0.211	0.202	0.125	-0.018	0.918
Proteobacteria	*Pseudomonas*	68	*mendocina*	-0.029	0.086	-0.006	0.676	-0.038	0.160
Proteobacteria	*Pseudomonas*	68	*migulae*	-0.016	0.512	0.007	0.627	-0.048	0.286
Proteobacteria	*Pseudomonas*	68	*monteilii*	-0.006	0.844	0.013	0.571	-0.040	0.370
Proteobacteria	*Pseudomonas*	68	*moraviensis*	-0.008	0.864	0.033	0.578	-0.123	0.401
Proteobacteria	*Pseudomonas*	68	*nitroreducens*	0.005	0.772	-0.008	0.600	0.050	0.156
Proteobacteria	*Pseudomonas*	68	*oleovorans*	-0.014	0.609	-0.010	0.712	-0.025	0.566
Proteobacteria	*Pseudomonas*	68	*orientalis*	-0.022	0.220	-0.004	0.656	-0.042	0.247
Proteobacteria	*Pseudomonas*	68	*oryzihabitans*	-0.019	0.340	-0.021	0.175	-0.012	0.708
Proteobacteria	*Pseudomonas*	68	*parafulva*	-0.039	0.091	-0.047	0.013	-0.001	0.991
Proteobacteria	*Pseudomonas*	68	*poae*	0.030	0.089	0.036	0.031	-0.003	0.929
Proteobacteria	*Pseudomonas*	68	*prosekii*	0.009	0.710	0.002	0.938	0.013	0.633
Proteobacteria	*Pseudomonas*	68	*protegens*	0.000	0.999	-0.001	0.931	0.027	0.238
Proteobacteria	*Pseudomonas*	68	*psychrophila*	-0.023	0.121	-0.033	0.005	0.034	0.133
Proteobacteria	*Pseudomonas*	68	*psychrotolerans*	-0.042	0.129	0.000	0.998	-0.119	0.003
Proteobacteria	*Pseudomonas*	68	*putida*	0.016	0.645	-0.003	0.915	0.055	0.236
Proteobacteria	*Pseudomonas*	68	*rhizosphaerae*	-0.008	0.796	0.007	0.825	-0.038	0.318
Proteobacteria	*Pseudomonas*	68	*rhodesiae*	-0.039	0.094	-0.026	0.203	-0.003	0.936
Proteobacteria	*Pseudomonas*	68	*saudiphocaensis*	-0.012	0.632	-0.028	0.211	0.030	0.358
Proteobacteria	*Pseudomonas*	68	*sediminis*	-0.026	0.272	-0.020	0.422	-0.018	0.586
Proteobacteria	*Pseudomonas*	68	*sihuiensis*	-0.016	0.473	-0.015	0.509	-0.024	0.446
Proteobacteria	*Pseudomonas*	68	*simiae*	-0.040	0.091	-0.012	0.614	-0.080	0.048
Proteobacteria	*Pseudomonas*	68	*sp._02C_26*	-0.032	0.350	-0.011	0.696	-0.063	0.256
Proteobacteria	*Pseudomonas*	68	*sp._11K1*	0.010	0.654	-0.001	0.950	0.016	0.607
Proteobacteria	*Pseudomonas*	68	*sp._ATCC_13867*	-0.006	0.772	-0.005	0.816	0.010	0.762
Proteobacteria	*Pseudomonas*	68	*sp._B10*	0.020	0.399	0.037	0.173	-0.033	0.402
Proteobacteria	*Pseudomonas*	68	*sp._C27(2019)*	0.024	0.257	0.028	0.169	-0.011	0.762
Proteobacteria	*Pseudomonas*	68	*sp._gcc21*	-0.020	0.256	-0.009	0.646	-0.040	0.052
Proteobacteria	*Pseudomonas*	68	*sp._J380*	-0.009	0.689	0.011	0.574	-0.048	0.234
Proteobacteria	*Pseudomonas*	68	*sp._LG1E9*	-0.031	0.091	-0.023	0.106	-0.033	0.286
Proteobacteria	*Pseudomonas*	68	*sp._LTJR-52*	-0.046	0.607	-0.060	0.435	-0.001	0.994
Proteobacteria	*Pseudomonas*	68	*sp._Lz4W*	-0.029	0.362	-0.031	0.324	0.001	0.970
Proteobacteria	*Pseudomonas*	68	*sp._MRSN12121*	0.027	0.208	0.023	0.192	0.001	0.975
Proteobacteria	*Pseudomonas*	68	*sp._NIBRBAC000502773*	0.013	0.437	0.021	0.166	-0.005	0.861
Proteobacteria	*Pseudomonas*	68	*sp._NP-1*	0.023	0.295	0.016	0.402	0.005	0.900
Proteobacteria	*Pseudomonas*	68	*sp._NS1(2017)*	0.018	0.438	0.009	0.644	0.049	0.121
Proteobacteria	*Pseudomonas*	68	*sp._OIL-1*	0.030	0.139	0.030	0.136	0.029	0.346
Proteobacteria	*Pseudomonas*	68	*sp._Os17*	-0.012	0.604	0.003	0.855	-0.022	0.541
Proteobacteria	*Pseudomonas*	68	*sp._phDV1*	0.028	0.318	0.035	0.187	-0.012	0.775
Proteobacteria	*Pseudomonas*	68	*sp._SGAir0191*	0.037	0.188	0.030	0.241	0.026	0.462
Proteobacteria	*Pseudomonas*	68	*sp._SNU_WT1*	0.012	0.678	-0.008	0.803	0.031	0.517
Proteobacteria	*Pseudomonas*	68	*sp._StFLB209*	-0.015	0.553	-0.025	0.179	-0.016	0.613
Proteobacteria	*Pseudomonas*	68	*sp._TUM18999*	-0.039	0.029	-0.031	0.017	0.009	0.715
Proteobacteria	*Pseudomonas*	68	*stutzeri*	0.005	0.868	0.038	0.167	-0.029	0.549
Proteobacteria	*Pseudomonas*	68	*synxantha*	-0.019	0.322	0.007	0.750	-0.051	3.5E-04
Proteobacteria	*Pseudomonas*	68	*syringae*	0.016	0.595	-0.003	0.912	0.027	0.484
Proteobacteria	*Pseudomonas*	68	*syringae_group_genomosp._3*	-0.024	0.213	0.001	0.953	-0.062	0.038
Proteobacteria	*Pseudomonas*	68	*taetrolens*	0.011	0.739	0.017	0.556	-0.021	0.529

Proteobacteria	*Pseudomonas*	68	*umsongensis*	-0.008	0.733	0.007	0.754	-0.020	0.661
Proteobacteria	*Pseudomonas*	68	*versuta*	0.004	0.870	0.016	0.562	-0.023	0.523
Proteobacteria	*Pseudomonas*	68	*viridiflava*	0.045	0.021	0.040	0.014	0.007	0.739
Proteobacteria	*Pseudomonas*	68	*xanthomarina*	-0.021	0.401	-0.015	0.527	-0.015	0.683
Firmicutes	*Acidaminococcus*	1	*intestini*	-0.035	0.077	-0.028	0.086	-0.022	0.575
Firmicutes	*Megasphaera*	1	*elsdenii*	0.045	0.129	0.021	0.528	0.068	0.069
Actinobacteria	*Rubrobacter*	1	*sp._SCSIO_52909*	-0.001	0.926	-0.013	0.352	0.059	0.015
Actinobacteria	*Conexibacter*	1	*woesei*	0.068	0.012	0.050	0.060	0.022	0.565
Firmicutes	*Finegoldia*	1	*magna*	-0.052	0.105	-0.057	0.034	0.013	0.775
Cyanobacteria	*Chroococcidiopsis*	1	*thermalis*	0.038	0.045	0.042	0.012	0.003	0.923
Cyanobacteria	*Nostoc*	4	*flagelliforme*	-0.021	0.248	-0.029	0.126	0.000	0.992
Cyanobacteria	*Nostoc*	4	*sp._Lobaria_pulmonaria_(5183)_cyanobiont*	-0.066	0.030	-0.050	0.063	-0.044	0.341
Cyanobacteria	*Nostoc*	4	*sp._NIES-2111*	-0.030	0.337	-0.015	0.616	-0.033	0.424
Cyanobacteria	*Nostoc*	4	*sphaeroides*	-0.008	0.760	-0.013	0.547	-0.009	0.850
Cyanobacteria	*Scytonema*	2	*sp._HK-05*	0.016	0.582	0.006	0.808	0.034	0.433
Cyanobacteria	*Scytonema*	2	*sp._NIES-4073*	-0.048	0.021	-0.038	0.063	-0.048	0.087
Cyanobacteria	*Oscillatoria*	1	*nigro-viridis*	0.031	0.221	0.047	0.048	-0.017	0.643

Table S9. Species level association results for the 30 genera related to FeNO (P<0.05) in the genus level differential abundance analysis results.

Phylum	Genus	N species	Species	Coef	P-value
Actinobacteria	*Actinomyces*	9	*israelii*	-0.019	0.592
Actinobacteria	*Actinomyces*	9	*naeslundii*	-0.048	0.346
Actinobacteria	*Actinomyces*	9	*oris*	0.048	0.324
Actinobacteria	*Actinomyces*	9	*radicidentis*	-0.032	0.306
Actinobacteria	*Actinomyces*	9	*sp._oral_taxon_169*	-0.030	0.576
Actinobacteria	*Actinomyces*	9	*sp._oral_taxon_171*	0.025	0.525
Actinobacteria	*Actinomyces*	9	*sp._oral_taxon_414*	-0.082	0.073
Actinobacteria	*Actinomyces*	9	*sp._oral_taxon_897*	-0.135	0.001
Actinobacteria	*Actinomyces*	9	*viscosus*	0.019	0.711
Actinobacteria	*Blastococcus*	1	*saxobsidens*	-0.073	0.033
Actinobacteria	*Intrasporangium*	1	*calvum*	-0.156	9.9E-05
Actinobacteria	*Janibacter*	4	*indicus*	0.001	0.981
Actinobacteria	*Janibacter*	4	*limosus*	0.002	0.962
Actinobacteria	*Janibacter*	4	*melonis*	-0.022	0.508
Actinobacteria	*Janibacter*	4	*sp._YB324*	-0.054	0.122
Actinobacteria	*Microbacterium*	32	*aurum*	-0.125	0.001
Actinobacteria	*Microbacterium*	32	*chocolatum*	-0.075	0.080
Actinobacteria	*Microbacterium*	32	*esteraromaticum*	-0.002	0.946
Actinobacteria	*Microbacterium*	32	*foliorum*	0.036	0.436
Actinobacteria	*Microbacterium*	32	*hominis*	-0.011	0.802
Actinobacteria	*Microbacterium*	32	*lemovicicum*	0.034	0.486
Actinobacteria	*Microbacterium*	32	*oleivorans*	-0.129	0.004
Actinobacteria	*Microbacterium*	32	*oryzae*	0.014	0.660
Actinobacteria	*Microbacterium*	32	*oxydans*	0.006	0.913
Actinobacteria	*Microbacterium*	32	*paraoxydans*	0.008	0.754
Actinobacteria	*Microbacterium*	32	*pygmaeum*	0.014	0.771
Actinobacteria	*Microbacterium*	32	*sediminis*	0.041	0.286
Actinobacteria	*Microbacterium*	32	*sp._10M-3C3*	0.006	0.859
Actinobacteria	*Microbacterium*	32	*sp._4R-513*	0.059	0.144
Actinobacteria	*Microbacterium*	32	*sp._A18JL241*	0.031	0.421
Actinobacteria	*Microbacterium*	32	*sp._ABRD_28*	0.030	0.459
Actinobacteria	*Microbacterium*	32	*sp._BH-3-3-3*	0.084	0.065
Actinobacteria	*Microbacterium*	32	*sp._CBA3102*	0.023	0.551
Actinobacteria	*Microbacterium*	32	*sp._HY82*	0.032	0.518
Actinobacteria	*Microbacterium*	32	*sp._LKL04*	0.038	0.501
Actinobacteria	*Microbacterium*	32	*sp._No._7*	-0.012	0.803
Actinobacteria	*Microbacterium*	32	*sp._Nx66*	0.031	0.423
Actinobacteria	*Microbacterium*	32	*sp._NY27*	-0.015	0.671
Actinobacteria	*Microbacterium*	32	*sp._PAMC_28756*	-0.091	0.100
Actinobacteria	*Microbacterium*	32	*sp._PM5*	-0.049	0.294
Actinobacteria	*Microbacterium*	32	*sp._RG1*	0.078	0.033
Actinobacteria	*Microbacterium*	32	*sp._TPU_3598*	0.018	0.707
Actinobacteria	*Microbacterium*	32	*sp._WY121*	-0.097	0.018
Actinobacteria	*Microbacterium*	32	*sp._XT11*	0.013	0.689
Actinobacteria	*Microbacterium*	32	*sp._Y-01*	-0.038	0.224
Actinobacteria	*Microbacterium*	32	*sp._YJN-G*	0.009	0.811
Actinobacteria	*Microbacterium*	32	*testaceum*	-0.157	0.006
Actinobacteria	*Kribbella*	2	*flavida*	-0.072	0.052
Actinobacteria	*Kribbella*	2	*qitaiheensis*	-0.067	0.029
Proteobacteria	*Ochrobactrum*	3	*anthropi*	-0.056	0.462
Proteobacteria	*Ochrobactrum*	3	*pituitosum*	0.059	0.154
Proteobacteria	*Ochrobactrum*	3	*pseudogrignonense*	0.088	0.025
Proteobacteria	*Rhodopseudomonas*	1	*palustris*	0.063	0.073
Proteobacteria	*Pannonibacter*	1	*phragmitetus*	0.076	0.014
Proteobacteria	*Erythrobacter*	1	*sp._A30-3*	-0.026	0.586
Firmicutes	*Jeotgalicoccus*	1	*saudimassiliensis*	0.126	0.140
Firmicutes	*Planococcus*	3	*antarcticus*	0.074	0.094
Firmicutes	*Planococcus*	3	*rifietoensis*	0.030	0.530
Firmicutes	*Planococcus*	3	*sp._MB-3u-03*	0.020	0.477
Firmicutes	*Salicibibacter*	1	*halophilus*	0.093	0.032
Firmicutes	*Solibacillus*	1	*silvestris*	-0.043	0.180
Firmicutes	*Limosilactobacillus*	4	*fermentum*	-0.061	0.013
Firmicutes	*Limosilactobacillus*	4	*Lactobacillus_mucosae*	0.103	0.044
Firmicutes	*Limosilactobacillus*	4	*Lactobacillus_vaginalis*	-0.070	0.061

Firmicutes	*Limosilactobacillus*	4	*reuteri*	0.011	0.867
Firmicutes	*Massilistercora*	1	*timonensis*	-0.047	0.305
Actinobacteria	*Olsenella*	1	*umbonata*	0.083	0.229
Bacteroidetes	*Adhaeribacter*	2	*sp._KUDC8001*	-0.090	0.015
Bacteroidetes	*Adhaeribacter*	2	*swui*	-0.025	0.564
Bacteroidetes	*Candidatus_Sulcia*	1	*muelleri*	-0.078	0.029
Bacteroidetes	*Capnocytophaga*	6	*canimorsus*	0.026	0.555
Bacteroidetes	*Capnocytophaga*	6	*cynodegmi*	0.026	0.664
Bacteroidetes	*Capnocytophaga*	6	*gingivalis*	0.075	0.102
Bacteroidetes	*Capnocytophaga*	6	*leadbetteri*	-0.022	0.718
Bacteroidetes	*Capnocytophaga*	6	*sp._FDAARGOS_737*	0.051	0.279
Bacteroidetes	*Capnocytophaga*	6	*sputigena*	0.004	0.937
Bacteroidetes	*Flavobacterium*	9	*anhuiense*	0.109	0.003
Bacteroidetes	*Flavobacterium*	9	*crocinum*	0.012	0.759
Bacteroidetes	*Flavobacterium*	9	*johnsoniae*	0.024	0.414
Bacteroidetes	*Flavobacterium*	9	*psychrophilum*	0.018	0.560
Bacteroidetes	*Flavobacterium*	9	*sangjuense*	-0.027	0.456
Bacteroidetes	*Flavobacterium*	9	*sp._HYN0086*	0.033	0.333
Bacteroidetes	*Flavobacterium*	9	*sp._KBS0721*	0.066	0.103
Bacteroidetes	*Flavobacterium*	9	*sp._M31R6*	-0.005	0.906
Bacteroidetes	*Flavobacterium*	9	*sp._MDT1-60*	0.095	0.010
Bacteroidetes	*Weeksella*	1	*virosa*	0.043	0.055
Proteobacteria	*Lelliottia*	1	*amnigena*	-0.086	0.036
Proteobacteria	*Rouxiella*	1	*badensis*	0.179	0.038
Proteobacteria	*Alcanivorax*	1	*sp._N3-2A*	0.096	1.5E-04
Proteobacteria	*Frederiksenia*	1	*canicola*	-0.187	6.8E-04
Proteobacteria	*Lysobacter*	4	*enzymogenes*	0.087	0.003
Proteobacteria	*Lysobacter*	4	*oculi*	0.076	0.130
Proteobacteria	*Lysobacter*	4	*soli*	0.036	0.434
Proteobacteria	*Lysobacter*	4	*sp._TY2-98*	0.059	0.065
Proteobacteria	*Stenotrophomonas*	7	*acidaminiphila*	0.142	0.028
Proteobacteria	*Stenotrophomonas*	7	*maltophilia*	0.133	1.2E-04
Proteobacteria	*Stenotrophomonas*	7	*rhizophila*	-0.061	0.116
Proteobacteria	*Stenotrophomonas*	7	*sp._169*	0.064	0.046
Proteobacteria	*Stenotrophomonas*	7	*sp._364*	0.052	0.109
Proteobacteria	*Stenotrophomonas*	7	*sp._LM091*	0.088	0.002
Proteobacteria	*Stenotrophomonas*	7	*sp._SAU14A_NAIMI4_5*	-0.015	0.683
Planctomycetes	*Planctomyces*	1	*sp._SH-PL62*	0.083	0.009
Cyanobacteria	*Scytonema*	2	*sp._HK-05*	0.044	0.443
Cyanobacteria	*Scytonema*	2	*sp._NIES-4073*	0.033	0.485

Table S10. Associations of lung function parameters with 31 genera examined in our 16S data.

			FEV_1		FVC		FEV_1/FVC	
Phylum	Family	Genus	Coef	P-value	Coef	P-value	Coef	P-value
[Thermi]	*Deinococcaceae*	*Deinococcus*	-0.047	0.052	-0.030	0.176	-0.024	0.543
Actinobacteria	*Cellulomonadaceae*	*Cellulomonas*	0.049	0.196	0.021	0.590	0.089	0.050
Actinobacteria	*Intrasporangiaceae*	*Kytococcus*	0.011	0.521	0.007	0.651	0.011	0.677
Actinobacteria	*Microbacteriaceae*	*Frigoribacterium*	-0.008	0.827	0.010	0.775	-0.074	0.123
Actinobacteria	*Microbacteriaceae*	*Microbacterium*	-0.018	0.724	-0.003	0.941	-0.073	0.264
Actinobacteria	*Micrococcaceae*	*Arthrobacter*	0.016	0.667	0.039	0.217	-0.037	0.452
Actinobacteria	*Micrococcaceae*	*Kocuria*	-0.010	0.600	-0.010	0.616	-0.007	0.819
Actinobacteria	*Nocardioidaceae*	*Friedmanniella*	0.077	0.037	0.028	0.405	0.118	0.002
Actinobacteria	*Bifidobacteriaceae*	*Bifidobacterium*	-0.011	0.615	-0.032	0.148	0.025	0.444
Actinobacteria	*Rubrobacteraceae*	*Rubrobacter*	0.016	0.371	0.014	0.388	-0.007	0.794
Bacteroidetes	*Cytophagaceae*	*Spirosoma*	0.013	0.709	-0.010	0.772	0.063	0.153
Cyanobacteria	*Nostocaceae*	*Nostoc*	-0.058	0.022	-0.053	0.037	-0.001	0.978
Cyanobacteria	*Xenococcaceae*	*Chroococcidiopsis*	0.016	0.560	-0.014	0.614	0.064	0.100
Firmicutes	*Paenibacillaceae*	*Saccharibacillus*	0.036	0.118	0.012	0.611	0.063	0.082
Firmicutes	*Enterococcaceae*	*Enterococcus*	-0.002	0.942	-0.012	0.685	0.070	0.004
Firmicutes	*Streptococcaceae*	*Lactococcus*	-0.002	0.940	0.019	0.335	-0.037	0.491
Firmicutes	*Streptococcaceae*	*Streptococcus*	-0.020	0.543	0.006	0.803	-0.091	0.133
Firmicutes	*.Tissierellaceae..*	*Finegoldia*	-0.026	0.542	-0.024	0.519	-0.013	0.826
Firmicutes	*Lachnospiraceae*	*Blautia*	-0.011	0.817	0.021	0.577	-0.036	0.657
Firmicutes	*Veillonellaceae*	*Acidaminococcus*	-0.004	0.853	-0.016	0.348	0.035	0.173
Firmicutes	*Veillonellaceae*	*Megasphaera*	0.022	0.248	0.017	0.342	0.010	0.689
Proteobacteria	*Brucellaceae*	*Ochrobactrum*	0.006	0.848	0.010	0.742	-0.021	0.514
Proteobacteria	*Hyphomicrobiaceae*	*Devosia*	-0.024	0.627	0.003	0.939	-0.079	0.245
Proteobacteria	*Rickettsiaceae*	*Wolbachia*	-0.009	0.344	-0.001	0.931	0.005	0.834
Proteobacteria	*Alcaligenaceae*	*Achromobacter*	-0.005	0.873	-0.002	0.958	-0.028	0.425
Proteobacteria	*Shewanellaceae*	*Shewanella*	0.030	0.190	0.045	0.007	-0.022	0.634
Proteobacteria	*Enterobacteriaceae*	*Buchnera*	0.028	0.053	0.017	0.198	0.025	0.340
Proteobacteria	*Halomonadaceae*	*Halomonas*	-0.027	0.380	-0.010	0.614	-0.037	0.533
Proteobacteria	*Pasteurellaceae*	*Haemophilus*	0.058	0.015	0.056	0.016	0.013	0.665
Proteobacteria	*Moraxellaceae*	*Moraxella*	0.027	0.059	0.006	0.558	0.052	0.002
Proteobacteria	*Pseudomonadaceae*	*Pseudomonas*	0.040	0.095	0.029	0.264	0.043	0.287

Table S11. Associations of FeNO with 11 genera examined in our 16S data.

Phylum	Family	Genus	Coef	P-value
Actinobacteria	*Actinomycetaceae*	*Actinomyces*	-0.001	0.983
Actinobacteria	*Microbacteriaceae*	*Microbacterium*	-0.025	0.706
Actinobacteria	*Nocardioidaceae*	*Kribbella*	-0.035	0.478
Bacteroidetes	*Cytophagaceae*	*Adhaeribacter*	-0.068	0.226
Bacteroidetes	*Flavobacteriaceae*	*Capnocytophaga*	0.062	0.362
Bacteroidetes	*Flavobacteriaceae*	*Flavobacterium*	0.002	0.974
Firmicutes	*Staphylococcaceae*	*Jeotgalicoccus*	-0.034	0.405
Proteobacteria	*Brucellaceae*	*Ochrobactrum*	0.053	0.126
Proteobacteria	*Alcanivoracaceae*	*Alcanivorax*	0.035	0.437
Proteobacteria	*Xanthomonadaceae*	*Lysobacter*	0.003	0.935
Proteobacteria	*Xanthomonadaceae*	*Stenotrophomonas*	0.094	0.008
