# Supplementary material for: House dust metagenome and pulmonary function in a US farming population
Source: Microbiome. 2024 Jul 18;12:129. doi: 10.1186/s40168-024-01823-y (PMC11256371; doi:10.1186/s40168-024-01823-y)
Supplement: Supplementary file 4 — Additional file 3: Online Supplement: Complete association results. [file 40168_2024_1823_MOESM3_ESM.pdf]

Online Supplement. Complete association results.

						FEV1			FVC			FEV1/FVC			FeNO		
Kingdom	Phylum	Class	Order	Family	Genus	Coef	SE	P	Coef	SE	P	Coef	SE	P	Coef	SE	P
Bacteria	Acidobacteria	Acidobacteriia	Acidobacteriales	Acidobacteriaceae	Terriglobus	-0.006	0.028	0.8400	0.013	0.025	0.6082	-0.047	0.045	0.3051	-0.016	0.055	0.7701
Bacteria	Acidobacteria	Vicinamibacteria		Vicinamibacteraceae	Luteitalea	-0.066	0.026	0.0122	-0.021	0.019	0.2858	-0.096	0.040	0.0164	0.041	0.034	0.2251
Bacteria	Actinobacteria	Acidimicrobiia	Acidimicrobiales	Iamiaceae	Actinomarinicola	0.003	0.025	0.9082	0.016	0.023	0.5024	-0.016	0.035	0.6515	-0.018	0.039	0.6512
Bacteria	Actinobacteria	Acidimicrobiia	Acidimicrobiales	Ilumatobacteraceae	Ilumatobacter	0.068	0.021	0.0012	0.051	0.021	0.0147	0.041	0.028	0.1350	0.028	0.038	0.4550
Bacteria	Actinobacteria	Actinobacteria	Actinomycetales	Actinomycetaceae	Actinomyces	-0.033	0.033	0.3223	-0.035	0.030	0.2453	-0.032	0.049	0.5132	-0.125	0.048	0.0095
Bacteria	Actinobacteria	Actinobacteria	Actinomycetales	Actinomycetaceae	Actinotignum	-0.006	0.022	0.7762	-0.005	0.019	0.7940	0.004	0.036	0.9013	0.014	0.042	0.7438
Bacteria	Actinobacteria	Actinobacteria	Actinomycetales	Actinomycetaceae	Flaviflexus	-0.007	0.030	0.8149	0.010	0.028	0.7139	-0.035	0.032	0.2843	0.037	0.038	0.3304
Bacteria	Actinobacteria	Actinobacteria	Actinomycetales	Actinomycetaceae	Pauljensenia	0.036	0.021	0.0867	0.013	0.016	0.4131	0.072	0.033	0.0259	-0.038	0.049	0.4402
Bacteria	Actinobacteria	Actinobacteria	Actinomycetales	Actinomycetaceae	Schaalia	0.007	0.026	0.7903	-0.002	0.024	0.9413	0.013	0.035	0.7194	-0.048	0.040	0.2322
Bacteria	Actinobacteria	Actinobacteria	Bifidobacteriales	Bifidobacteriaceae	Bifidobacterium	0.013	0.021	0.5290	-0.012	0.019	0.5380	0.067	0.024	0.0055	-0.016	0.034	0.6424
Bacteria	Actinobacteria	Actinobacteria	Bifidobacteriales	Bifidobacteriaceae	Gardnerella	-0.009	0.022	0.6701	-0.007	0.018	0.7154	-0.033	0.032	0.3017	-0.042	0.041	0.2977
Bacteria	Actinobacteria	Actinobacteria	Bifidobacteriales	Bifidobacteriaceae	Parascardovia	-0.055	0.022	0.0122	-0.032	0.021	0.1280	-0.103	0.035	0.0030	0.006	0.042	0.8896
Bacteria	Actinobacteria	Actinobacteria	Corynebacteriales	Corynebacteriaceae	Corynebacterium	0.030	0.041	0.4667	0.017	0.037	0.6344	0.100	0.058	0.0836	0.092	0.072	0.2015
Bacteria	Actinobacteria	Actinobacteria	Corynebacteriales	Dietziaceae	Dietzia	0.018	0.021	0.4057	0.014	0.022	0.5196	-0.006	0.025	0.8026	0.050	0.034	0.1366
Bacteria	Actinobacteria	Actinobacteria	Corynebacteriales	Gordoniaceae	Gordonia	-0.003	0.019	0.8538	-0.013	0.021	0.5351	0.028	0.028	0.3181	0.009	0.036	0.7949
Bacteria	Actinobacteria	Actinobacteria	Corynebacteriales	Mycobacteriaceae	Lawsonella	-0.044	0.031	0.1548	-0.040	0.029	0.1598	-0.007	0.037	0.8417	-0.023	0.042	0.5965
Bacteria	Actinobacteria	Actinobacteria	Corynebacteriales	Mycobacteriaceae	Mycobacterium	0.003	0.018	0.8561	0.013	0.017	0.4392	-0.033	0.023	0.1558	0.034	0.027	0.2095
Bacteria	Actinobacteria	Actinobacteria	Corynebacteriales	Mycobacteriaceae	Mycobacteroides	-0.013	0.017	0.4436	-0.023	0.021	0.2525	0.044	0.026	0.0828	0.020	0.026	0.4525
Bacteria	Actinobacteria	Actinobacteria	Corynebacteriales	Mycobacteriaceae	Mycolicibacterium	0.003	0.022	0.8819	0.005	0.023	0.8344	-0.013	0.029	0.6492	0.007	0.030	0.8260
Bacteria	Actinobacteria	Actinobacteria	Corynebacteriales	Nocardiaceae	Nocardia	0.011	0.017	0.5247	0.000	0.017	0.9834	0.031	0.025	0.2256	0.021	0.030	0.4840
Bacteria	Actinobacteria	Actinobacteria	Corynebacteriales	Nocardiaceae	Rhodococcus	0.045	0.024	0.0648	0.033	0.027	0.2276	0.036	0.031	0.2353	0.044	0.037	0.2306
Bacteria	Actinobacteria	Actinobacteria	Corynebacteriales	Tsukamurellaceae	Tsukamurella	-0.010	0.022	0.6576	0.005	0.023	0.8317	-0.035	0.028	0.2206	-0.022	0.037	0.5572
Bacteria	Actinobacteria	Actinobacteria	Geodermatophilales	Geodermatophilaceae	Blastococcus	0.032	0.018	0.0797	0.020	0.017	0.2253	0.027	0.027	0.3118	-0.066	0.032	0.0386
Bacteria	Actinobacteria	Actinobacteria	Geodermatophilales	Geodermatophilaceae	Geodermatophilus	0.010	0.019	0.5786	0.008	0.020	0.6899	-0.016	0.026	0.5309	0.030	0.033	0.3490
Bacteria	Actinobacteria	Actinobacteria	Geodermatophilales	Geodermatophilaceae	Modestobacter	-0.008	0.031	0.8011	-0.016	0.025	0.5329	-0.004	0.041	0.9239	-0.027	0.041	0.5173
Bacteria	Actinobacteria	Actinobacteria	Kineosporiales	Kineosporiaceae	Kineococcus	-0.027	0.028	0.3375	-0.012	0.025	0.6430	-0.068	0.043	0.1191	0.032	0.046	0.4820
Bacteria	Actinobacteria	Actinobacteria	Micrococcales	Beutenbergiaceae	Miniimonas	-0.003	0.017	0.8714	0.001	0.014	0.9353	-0.022	0.028	0.4368	-0.077	0.042	0.0647
Bacteria	Actinobacteria	Actinobacteria	Micrococcales	Bogoriellaceae	Georgenia	-0.010	0.025	0.6847	0.003	0.023	0.9038	-0.006	0.036	0.8782	-0.051	0.037	0.1652
Bacteria	Actinobacteria	Actinobacteria	Micrococcales	Brevibacteriaceae	Brevibacterium	-0.005	0.047	0.9073	-0.014	0.044	0.7522	-0.025	0.064	0.6975	-0.011	0.076	0.8847
Bacteria	Actinobacteria	Actinobacteria	Micrococcales	Cellulomonadaceae	Actinotalea	0.014	0.023	0.5410	-0.001	0.020	0.9463	0.044	0.035	0.2187	0.018	0.040	0.6514
Bacteria	Actinobacteria	Actinobacteria	Micrococcales	Cellulomonadaceae	Cellulomonas	-0.062	0.022	0.0042	-0.057	0.019	0.0034	-0.054	0.058	0.3470	0.045	0.036	0.2172
Bacteria	Actinobacteria	Actinobacteria	Micrococcales	Cellulomonadaceae	Oerskovia	-0.039	0.021	0.0698	-0.021	0.021	0.3192	-0.032	0.032	0.3225	-0.025	0.031	0.4200
Bacteria	Actinobacteria	Actinobacteria	Micrococcales	Dermabacteraceae	Brachybacterium	-0.018	0.034	0.5867	-0.016	0.035	0.6504	-0.038	0.047	0.4158	-0.011	0.051	0.8250
Bacteria	Actinobacteria	Actinobacteria	Micrococcales	Dermabacteraceae	Dermabacter	0.004	0.026	0.8656	0.010	0.021	0.6469	-0.039	0.042	0.3569	-0.039	0.038	0.3028
Bacteria	Actinobacteria	Actinobacteria	Micrococcales	Dermacoccaceae	Dermacoccus	0.006	0.018	0.7527	0.015	0.022	0.5057	-0.008	0.030	0.7873	-0.010	0.042	0.8173
Bacteria	Actinobacteria	Actinobacteria	Micrococcales	Dermacoccaceae	Kytococcus	0.046	0.022	0.0381	0.047	0.021	0.0252	0.013	0.036	0.7180	0.009	0.037	0.8135
Bacteria	Actinobacteria	Actinobacteria	Micrococcales	Intrasporangiaceae	Arsenicicoccus	-0.004	0.018	0.8131	0.014	0.015	0.3419	-0.050	0.037	0.1771	-0.035	0.037	0.3432
Bacteria	Actinobacteria	Actinobacteria	Micrococcales	Intrasporangiaceae	Intrasporangium	-0.026	0.027	0.3382	-0.022	0.024	0.3549	-0.032	0.038	0.3908	-0.145	0.041	3.41E-04
Bacteria	Actinobacteria	Actinobacteria	Micrococcales	Intrasporangiaceae	Janibacter	-0.009	0.027	0.7471	-0.020	0.024	0.4016	0.006	0.025	0.8068	-0.069	0.033	0.0366
Bacteria	Actinobacteria	Actinobacteria	Micrococcales	Intrasporangiaceae	Ornithinimicrobium	-0.006	0.032	0.8429	0.037	0.035	0.2909	-0.112	0.039	0.0041	0.043	0.037	0.2490
Bacteria	Actinobacteria	Actinobacteria	Micrococcales	Intrasporangiaceae	Phycicoccus	-0.015	0.036	0.6779	-0.038	0.032	0.2397	0.084	0.042	0.0467	-0.070	0.049	0.1534
Bacteria	Actinobacteria	Actinobacteria	Micrococcales	Intrasporangiaceae	Serinicoccus	0.028	0.017	0.1000	0.019	0.019	0.3204	0.029	0.017	0.0929	-0.002	0.024	0.9208
Bacteria	Actinobacteria	Actinobacteria	Micrococcales	Intrasporangiaceae	Tetrasphaera	-0.010	0.021	0.6323	0.018	0.020	0.3644	-0.036	0.030	0.2344	0.011	0.037	0.7616
Bacteria	Actinobacteria	Actinobacteria	Micrococcales	Jonesiaceae	Jonesia	-0.023	0.027	0.4024	-0.017	0.023	0.4748	-0.048	0.038	0.2093	-0.017	0.036	0.6333
Bacteria	Actinobacteria	Actinobacteria	Micrococcales	Microbacteriaceae	Agrococcus	-0.024	0.030	0.4152	-0.020	0.026	0.4343	-0.049	0.046	0.2904	-0.026	0.043	0.5524
Bacteria	Actinobacteria	Actinobacteria	Micrococcales	Microbacteriaceae	Agromyces	-0.004	0.025	0.8857	-0.001	0.024	0.9598	-0.038	0.039	0.3221	-0.053	0.042	0.2115
Bacteria	Actinobacteria	Actinobacteria	Micrococcales	Microbacteriaceae	Clavibacter	0.012	0.026	0.6367	0.021	0.027	0.4308	-0.013	0.031	0.6728	0.010	0.045	0.8165
Bacteria	Actinobacteria	Actinobacteria	Micrococcales	Microbacteriaceae	Cnuibacter	0.004	0.027	0.8874	-0.014	0.025	0.5753	0.066	0.033	0.0418	-0.051	0.047	0.2776
Bacteria	Actinobacteria	Actinobacteria	Micrococcales	Microbacteriaceae	Cryobacterium	0.076	0.030	0.0127	0.058	0.028	0.0402	0.051	0.033	0.1251	0.035	0.044	0.4293
Bacteria	Actinobacteria	Actinobacteria	Micrococcales	Microbacteriaceae	Curtobacterium	0.020	0.038	0.6029	0.018	0.035	0.6008	0.041	0.056	0.4661	-0.044	0.055	0.4214
Bacteria	Actinobacteria	Actinobacteria	Micrococcales	Microbacteriaceae	Frigoribacterium	0.064	0.023	0.0054	0.078	0.024	0.0011	-0.005	0.032	0.8853	-0.013	0.035	0.7037
Bacteria	Actinobacteria	Actinobacteria	Micrococcales	Microbacteriaceae	Frondihabitans	0.014	0.024	0.5429	0.017	0.024	0.4911	-0.007	0.027	0.8098	-0.049	0.039	0.2132
Bacteria	Actinobacteria	Actinobacteria	Micrococcales	Microbacteriaceae	Herbiconiux	-0.015	0.018	0.4049	0.016	0.017	0.3473	-0.064	0.026	0.0159	-0.015	0.025	0.5398
Bacteria	Actinobacteria	Actinobacteria	Micrococcales	Microbacteriaceae	Leifsonia	-0.026	0.032	0.4172	-0.006	0.028	0.8341	-0.049	0.043	0.2462	0.070	0.041	0.0872

Bacteria	Actinobacteria	Actinobacteria	Micrococcales	Microbacteriaceae	Leucobacter	0.026	0.020	0.2054	0.028	0.020	0.1655	0.008	0.026	0.7666	-0.017	0.033	0.6099
Bacteria	Actinobacteria	Actinobacteria	Micrococcales	Microbacteriaceae	Microbacterium	0.044	0.028	0.1205	0.004	0.024	0.8814	0.099	0.043	0.0199	-0.106	0.050	0.0338
Bacteria	Actinobacteria	Actinobacteria	Micrococcales	Microbacteriaceae	Microterricola	0.006	0.018	0.7371	-0.004	0.019	0.8161	0.025	0.025	0.3228	0.021	0.030	0.4900
Bacteria	Actinobacteria	Actinobacteria	Micrococcales	Microbacteriaceae	Plantibacter	-0.034	0.026	0.1932	-0.019	0.023	0.4083	-0.020	0.040	0.6204	0.043	0.042	0.3009
Bacteria	Actinobacteria	Actinobacteria	Micrococcales	Microbacteriaceae	Protaetiibacter	-0.010	0.025	0.6938	-0.023	0.021	0.2667	0.025	0.031	0.4226	-0.028	0.038	0.4620
Bacteria	Actinobacteria	Actinobacteria	Micrococcales	Microbacteriaceae	Rathayibacter	-0.016	0.019	0.3906	0.008	0.014	0.5596	-0.079	0.021	2.08E-04	-0.002	0.031	0.9456
Bacteria	Actinobacteria	Actinobacteria	Micrococcales	Micrococcaceae	Arthrobacter	-0.021	0.025	0.4009	-0.048	0.025	0.0584	0.070	0.035	0.0430	0.034	0.042	0.4218
Bacteria	Actinobacteria	Actinobacteria	Micrococcales	Micrococcaceae	Citricoccus	-0.003	0.021	0.8929	0.002	0.018	0.9168	-0.012	0.031	0.7032	0.054	0.031	0.0845
Bacteria	Actinobacteria	Actinobacteria	Micrococcales	Micrococcaceae	Glutamicibacter	-0.031	0.029	0.2808	-0.002	0.028	0.9348	-0.066	0.041	0.1055	0.048	0.053	0.3701
Bacteria	Actinobacteria	Actinobacteria	Micrococcales	Micrococcaceae	Kocuria	0.043	0.021	0.0395	0.044	0.020	0.0261	0.016	0.027	0.5718	0.025	0.035	0.4779
Bacteria	Actinobacteria	Actinobacteria	Micrococcales	Micrococcaceae	Micrococcus	-0.024	0.028	0.3913	-0.014	0.028	0.6073	-0.016	0.032	0.6120	0.051	0.037	0.1737
Bacteria	Actinobacteria	Actinobacteria	Micrococcales	Micrococcaceae	Paenarthrobacter	-0.039	0.024	0.1021	-0.008	0.023	0.7299	-0.054	0.032	0.0958	0.005	0.033	0.8848
Bacteria	Actinobacteria	Actinobacteria	Micrococcales	Micrococcaceae	Pseudarthrobacter	0.039	0.023	0.0840	0.026	0.025	0.2878	0.025	0.029	0.3833	-0.064	0.036	0.0765
Bacteria	Actinobacteria	Actinobacteria	Micrococcales	Micrococcaceae	Rothia	0.045	0.028	0.1023	0.037	0.026	0.1494	0.032	0.052	0.5400	0.022	0.052	0.6793
Bacteria	Actinobacteria	Actinobacteria	Micrococcales	Promicromonosporaceae	Cellulosimicrobium	0.003	0.028	0.9248	-0.007	0.026	0.8021	0.036	0.039	0.3480	-0.046	0.042	0.2720
Bacteria	Actinobacteria	Actinobacteria	Micrococcales	Ruaniaceae	Haloactinobacterium	-0.020	0.021	0.3429	0.008	0.022	0.7288	-0.066	0.029	0.0220	0.026	0.032	0.4234
Bacteria	Actinobacteria	Actinobacteria	Micrococcales	Ruaniaceae	Luteimicrobium	-0.033	0.029	0.2487	-0.040	0.027	0.1335	0.005	0.028	0.8472	-0.029	0.035	0.4109
Bacteria	Actinobacteria	Actinobacteria	Micrococcales	Ruaniaceae	Ruania	-0.007	0.018	0.7015	-0.007	0.019	0.6996	0.015	0.023	0.5248	-0.026	0.037	0.4923
Bacteria	Actinobacteria	Actinobacteria	Micrococcales	Sanguibacteraceae	Sanguibacter	0.022	0.021	0.2852	0.017	0.020	0.3954	0.015	0.025	0.5551	0.006	0.032	0.8496
Bacteria	Actinobacteria	Actinobacteria	Micromonosporales	Micromonosporaceae	Actinoplanes	-0.015	0.034	0.6596	-0.017	0.030	0.5751	-0.001	0.050	0.9890	-0.042	0.060	0.4923
Bacteria	Actinobacteria	Actinobacteria	Micromonosporales	Micromonosporaceae	Micromonospora	0.051	0.028	0.0696	0.045	0.023	0.0440	0.034	0.045	0.4550	-0.042	0.047	0.3654
Bacteria	Actinobacteria	Actinobacteria	Micromonosporales	Micromonosporaceae	Phytohabitans	0.013	0.022	0.5481	0.013	0.019	0.4944	-0.008	0.041	0.8376	0.002	0.050	0.9647
Bacteria	Actinobacteria	Actinobacteria	Nakamurellales	Nakamurellaceae	Nakamurella	-0.007	0.019	0.7131	-0.024	0.016	0.1405	0.042	0.023	0.0627	-0.006	0.026	0.8123
Bacteria	Actinobacteria	Actinobacteria	Propionibacteriales	Nocardioidaceae	Aeromicrobium	0.030	0.026	0.2492	0.022	0.024	0.3451	0.018	0.039	0.6377	-0.058	0.040	0.1467
Bacteria	Actinobacteria	Actinobacteria	Propionibacteriales	Nocardioidaceae	Friedmanniella	0.059	0.026	0.0230	0.017	0.026	0.5153	0.117	0.037	0.0015	0.071	0.043	0.0968
Bacteria	Actinobacteria	Actinobacteria	Propionibacteriales	Nocardioidaceae	Kribbella	0.017	0.017	0.2982	0.014	0.017	0.4270	-0.003	0.028	0.9076	-0.081	0.032	0.0120
Bacteria	Actinobacteria	Actinobacteria	Propionibacteriales	Nocardioidaceae	Marmoricola	-0.039	0.028	0.1714	-0.030	0.027	0.2548	-0.031	0.035	0.3807	-0.026	0.050	0.6054
Bacteria	Actinobacteria	Actinobacteria	Propionibacteriales	Nocardioidaceae	Micropruina	-0.032	0.020	0.1110	-0.020	0.018	0.2662	-0.034	0.031	0.2769	-0.057	0.032	0.0710
Bacteria	Actinobacteria	Actinobacteria	Propionibacteriales	Nocardioidaceae	Mumia	-0.025	0.025	0.3072	-0.031	0.020	0.1293	0.009	0.031	0.7764	0.018	0.030	0.5406
Bacteria	Actinobacteria	Actinobacteria	Propionibacteriales	Nocardioidaceae	Nocardioides	-0.061	0.038	0.1090	-0.054	0.035	0.1190	-0.028	0.057	0.6197	0.109	0.059	0.0642
Bacteria	Actinobacteria	Actinobacteria	Propionibacteriales	Nocardioidaceae	Pimelobacter	0.014	0.019	0.4529	0.003	0.017	0.8738	0.028	0.028	0.3101	0.002	0.029	0.9569
Bacteria	Actinobacteria	Actinobacteria	Propionibacteriales	Propionibacteriaceae	Acidipropionibacterium	-0.003	0.018	0.8505	-0.015	0.015	0.3074	0.019	0.036	0.5958	0.016	0.038	0.6744
Bacteria	Actinobacteria	Actinobacteria	Propionibacteriales	Propionibacteriaceae	Auraticoccus	0.028	0.024	0.2410	0.025	0.019	0.1816	0.023	0.035	0.5201	-0.006	0.032	0.8642
Bacteria	Actinobacteria	Actinobacteria	Propionibacteriales	Propionibacteriaceae	Cutibacterium	-0.014	0.031	0.6459	0.013	0.028	0.6459	-0.101	0.040	0.0115	-0.002	0.047	0.9687
Bacteria	Actinobacteria	Actinobacteria	Propionibacteriales	Propionibacteriaceae	Microlunatus	-0.014	0.028	0.6104	-0.013	0.026	0.6098	0.000	0.029	0.9942	0.038	0.052	0.4694
Bacteria	Actinobacteria	Actinobacteria	Propionibacteriales	Propionibacteriaceae	Propionibacterium	-0.015	0.018	0.4124	-0.012	0.014	0.3839	-0.016	0.032	0.6220	-0.042	0.024	0.0866
Bacteria	Actinobacteria	Actinobacteria	Propionibacteriales	Propionibacteriaceae	Propioniciclava	-0.011	0.024	0.6469	-0.013	0.025	0.6045	-0.003	0.024	0.8983	0.008	0.023	0.7246
Bacteria	Actinobacteria	Actinobacteria	Propionibacteriales	Propionibacteriaceae	Propionimicrobium	0.001	0.016	0.9563	-0.001	0.015	0.9401	0.008	0.024	0.7390	-0.033	0.027	0.2228
Bacteria	Actinobacteria	Actinobacteria	Propionibacteriales	Propionibacteriaceae	Pseudopropionibacterium	0.020	0.017	0.2254	0.008	0.016	0.6280	0.053	0.027	0.0492	-0.010	0.031	0.7598
Bacteria	Actinobacteria	Actinobacteria	Propionibacteriales	Propionibacteriaceae	Tessaracoccus	-0.022	0.025	0.3736	-0.032	0.021	0.1219	0.028	0.038	0.4682	-0.003	0.047	0.9503
Bacteria	Actinobacteria	Actinobacteria	Pseudonocardiales	Pseudonocardiaceae	Amycolatopsis	0.012	0.025	0.6428	0.002	0.021	0.9190	0.033	0.034	0.3299	-0.028	0.046	0.5508
Bacteria	Actinobacteria	Actinobacteria	Pseudonocardiales	Pseudonocardiaceae	Lentzea	0.028	0.020	0.1657	0.008	0.018	0.6684	0.020	0.029	0.4859	-0.031	0.040	0.4380
Bacteria	Actinobacteria	Actinobacteria	Pseudonocardiales	Pseudonocardiaceae	Pseudonocardia	-0.019	0.020	0.3343	0.002	0.014	0.9018	-0.053	0.033	0.1075	0.018	0.035	0.5997
Bacteria	Actinobacteria	Actinobacteria	Pseudonocardiales	Pseudonocardiaceae	Saccharomonospora	0.007	0.023	0.7626	0.016	0.022	0.4725	-0.034	0.034	0.3052	-0.010	0.046	0.8263
Bacteria	Actinobacteria	Actinobacteria	Pseudonocardiales	Pseudonocardiaceae	Saccharopolyspora	0.017	0.026	0.5146	0.020	0.028	0.4647	-0.012	0.032	0.7037	0.053	0.028	0.0545
Bacteria	Actinobacteria	Actinobacteria	Sporichthyales	Sporichthyaceae	Epidermidibacterium	-0.022	0.024	0.3756	-0.029	0.020	0.1606	0.002	0.026	0.9338	-0.015	0.037	0.6887
Bacteria	Actinobacteria	Actinobacteria	Streptomycetales	Streptomycetaceae	Streptomyces	0.003	0.024	0.8914	-0.017	0.023	0.4600	0.003	0.035	0.9223	-0.034	0.034	0.3260
Bacteria	Actinobacteria	Actinobacteria	Streptosporangiales	Nocardiopsaceae	Nocardiopsis	0.028	0.018	0.1321	0.043	0.019	0.0262	-0.036	0.028	0.1927	0.027	0.030	0.3790
Bacteria	Actinobacteria	Actinobacteria	Streptosporangiales	Nocardiopsaceae	Thermobifida	0.008	0.022	0.7071	-0.011	0.020	0.6042	0.033	0.031	0.2903	0.016	0.041	0.6974
Bacteria	Actinobacteria	Actinobacteria	Streptosporangiales	Streptosporangiaceae	Nonomuraea	-0.044	0.029	0.1223	-0.007	0.017	0.7040	-0.091	0.047	0.0520	0.012	0.027	0.6657
Bacteria	Actinobacteria	Actinobacteria	Streptosporangiales	Streptosporangiaceae	Streptosporangium	0.035	0.016	0.0309	0.020	0.017	0.2296	0.040	0.025	0.1097	0.005	0.027	0.8518
Bacteria	Actinobacteria	Coriobacteriia	Coriobacteriales	Atopobiaceae	Lancefieldella	0.006	0.022	0.7833	0.002	0.029	0.9345	0.017	0.050	0.7299	-0.019	0.036	0.5948
Bacteria	Actinobacteria	Coriobacteriia	Coriobacteriales	Atopobiaceae	Libanicoccus	-0.047	0.027	0.0869	-0.057	0.030	0.0544	0.020	0.035	0.5714	-0.060	0.042	0.1574
Bacteria	Actinobacteria	Coriobacteriia	Coriobacteriales	Atopobiaceae	Olsenella	-0.009	0.022	0.6991	-0.004	0.020	0.8467	0.009	0.025	0.7009	0.106	0.045	0.0187
Bacteria	Actinobacteria	Coriobacteriia	Coriobacteriales	Coriobacteriaceae	Collinsella	0.004	0.032	0.9144	-0.014	0.028	0.6043	0.078	0.052	0.1317	-0.075	0.047	0.1062
Bacteria	Actinobacteria	Coriobacteriia	Eggerthellales	Eggerthellaceae	Denitrobacterium	0.016	0.026	0.5398	0.024	0.027	0.3762	-0.019	0.031	0.5461	0.069	0.044	0.1159
Bacteria	Actinobacteria	Coriobacteriia	Eggerthellales	Eggerthellaceae	Eggerthella	0.026	0.032	0.4146	0.000	0.029	0.9914	0.048	0.041	0.2404	0.003	0.044	0.9484

Bacteria	Actinobacteria	Coriobacteriia	Eggerthellales	Eggerthellaceae	Gordonibacter	0.017	0.022	0.4309	0.024	0.024	0.3053	0.008	0.031	0.7902	-0.041	0.030	0.1711
Bacteria	Actinobacteria	Rubrobacteria	Rubrobacterales	Baekduiaceae	Baekduia	-0.009	0.032	0.7844	-0.037	0.031	0.2299	0.060	0.044	0.1791	-0.043	0.062	0.4923
Bacteria	Actinobacteria	Rubrobacteria	Rubrobacterales	Rubrobacteraceae	Rubrobacter	-0.004	0.016	0.7853	-0.023	0.015	0.1109	0.061	0.024	0.0116	0.017	0.030	0.5783
Bacteria	Actinobacteria	Thermoleophilia	Solirubrobacterales	Conexibacteraceae	Conexibacter	0.081	0.035	0.0197	0.060	0.034	0.0759	0.040	0.049	0.4181	-0.067	0.065	0.3080
Bacteria	Bacteroidetes	Bacteroidia	Bacteroidales	Bacteroidaceae	Bacteroides	-0.023	0.055	0.6705	0.022	0.041	0.5880	-0.071	0.075	0.3446	-0.083	0.076	0.2754
Bacteria	Bacteroidetes	Bacteroidia	Bacteroidales	Bacteroidaceae	Phocaeicola	0.030	0.036	0.4058	-0.007	0.027	0.8087	0.094	0.055	0.0876	-0.015	0.047	0.7522
Bacteria	Bacteroidetes	Bacteroidia	Bacteroidales	Odoribacteraceae	Butyricimonas	0.007	0.023	0.7508	0.000	0.018	0.9941	0.028	0.040	0.4892	-0.045	0.039	0.2474
Bacteria	Bacteroidetes	Bacteroidia	Bacteroidales	Odoribacteraceae	Odoribacter	-0.022	0.026	0.4061	0.001	0.020	0.9597	-0.078	0.046	0.0896	-0.066	0.042	0.1206
Bacteria	Bacteroidetes	Bacteroidia	Bacteroidales	Porphyromonadaceae	Porphyromonas	-0.009	0.023	0.7028	-0.015	0.022	0.4827	0.043	0.039	0.2655	0.005	0.047	0.9086
Bacteria	Bacteroidetes	Bacteroidia	Bacteroidales	Prevotellaceae	Paraprevotella	-0.002	0.023	0.9344	-0.015	0.022	0.5042	0.047	0.033	0.1466	0.033	0.035	0.3442
Bacteria	Bacteroidetes	Bacteroidia	Bacteroidales	Prevotellaceae	Prevotella	0.016	0.029	0.5883	0.000	0.026	0.9993	0.018	0.039	0.6430	-0.032	0.046	0.4855
Bacteria	Bacteroidetes	Bacteroidia	Bacteroidales	Rikenellaceae	Alistipes	-0.014	0.038	0.7169	0.045	0.034	0.1918	-0.132	0.059	0.0249	-0.027	0.054	0.6194
Bacteria	Bacteroidetes	Bacteroidia	Bacteroidales	Tannerellaceae	Parabacteroides	0.001	0.029	0.9699	0.007	0.023	0.7471	0.014	0.046	0.7609	0.065	0.047	0.1711
Bacteria	Bacteroidetes	Bacteroidia	Bacteroidales	Tannerellaceae	Tannerella	0.020	0.022	0.3646	0.003	0.018	0.8862	0.051	0.035	0.1489	-0.071	0.043	0.1034
Bacteria	Bacteroidetes	Bacteroidia	Bacteroidales		Phocaeicola	-0.047	0.029	0.1085	-0.048	0.031	0.1287	0.003	0.054	0.9563	0.001	0.050	0.9927
Bacteria	Bacteroidetes	Chitinophagia	Chitinophagales	Chitinophagaceae	Flavisolibacter	-0.031	0.026	0.2316	-0.031	0.024	0.2026	-0.018	0.033	0.5884	0.003	0.037	0.9351
Bacteria	Bacteroidetes	Cytophagia	Cytophagales	Amoebophilaceae	Candidatus_Cardinium	0.040	0.024	0.1011	0.012	0.024	0.6098	0.048	0.024	0.0488	0.057	0.031	0.0681
Bacteria	Bacteroidetes	Cytophagia	Cytophagales	Cytophagaceae	Dyadobacter	-0.010	0.020	0.6152	-0.004	0.021	0.8697	-0.010	0.030	0.7349	-0.040	0.038	0.2968
Bacteria	Bacteroidetes	Cytophagia	Cytophagales	Cytophagaceae	Fibrella	0.004	0.024	0.8821	0.005	0.022	0.8395	0.017	0.033	0.6103	-0.019	0.037	0.6077
Bacteria	Bacteroidetes	Cytophagia	Cytophagales	Cytophagaceae	Rhodocytophaga	0.002	0.030	0.9443	0.006	0.021	0.7631	0.017	0.047	0.7152	0.021	0.041	0.6183
Bacteria	Bacteroidetes	Cytophagia	Cytophagales	Cytophagaceae	Spirosoma	0.058	0.026	0.0284	0.039	0.026	0.1337	0.050	0.033	0.1295	-0.018	0.040	0.6574
Bacteria	Bacteroidetes	Cytophagia	Cytophagales	Hymenobacteraceae	Adhaeribacter	-0.023	0.023	0.3268	-0.029	0.022	0.1993	0.018	0.037	0.6344	-0.077	0.036	0.0358
Bacteria	Bacteroidetes	Cytophagia	Cytophagales	Hymenobacteraceae	Hymenobacter	0.046	0.028	0.0977	0.011	0.026	0.6599	0.028	0.037	0.4506	-0.075	0.044	0.0892
Bacteria	Bacteroidetes	Cytophagia	Cytophagales	Hymenobacteraceae	Pontibacter	0.028	0.028	0.3096	-0.009	0.027	0.7441	0.052	0.034	0.1330	0.067	0.048	0.1649
Bacteria	Bacteroidetes	Flavobacteriia	Flavobacteriales	Blattabacteriaceae	Blattabacterium	0.002	0.021	0.9333	-0.010	0.016	0.5454	0.031	0.026	0.2365	0.020	0.025	0.4248
Bacteria	Bacteroidetes	Flavobacteriia	Flavobacteriales	Blattabacteriaceae	Candidatus_Sulcia	0.033	0.020	0.0859	0.036	0.018	0.0495	0.005	0.031	0.8587	-0.073	0.034	0.0327
Bacteria	Bacteroidetes	Flavobacteriia	Flavobacteriales	Flavobacteriaceae	Capnocytophaga	0.011	0.026	0.6792	0.010	0.023	0.6741	0.001	0.040	0.9833	0.132	0.052	0.0108
Bacteria	Bacteroidetes	Flavobacteriia	Flavobacteriales	Flavobacteriaceae	Flavobacterium	0.026	0.027	0.3197	0.013	0.025	0.6076	0.032	0.034	0.3430	0.089	0.043	0.0381
Bacteria	Bacteroidetes	Flavobacteriia	Flavobacteriales	Flavobacteriaceae	Myroides	-0.009	0.027	0.7450	-0.002	0.017	0.9136	-0.020	0.043	0.6480	-0.035	0.028	0.2117
Bacteria	Bacteroidetes	Flavobacteriia	Flavobacteriales	Flavobacteriaceae	Salegentibacter	0.012	0.017	0.4718	0.017	0.023	0.4699	0.003	0.026	0.9127	-0.005	0.028	0.8554
Bacteria	Bacteroidetes	Flavobacteriia	Flavobacteriales	Weeksellaceae	Chryseobacterium	0.009	0.024	0.7086	-0.001	0.031	0.9714	0.001	0.035	0.9693	0.015	0.037	0.6823
Bacteria	Bacteroidetes	Flavobacteriia	Flavobacteriales	Weeksellaceae	Cruoricaptor	-0.049	0.027	0.0637	-0.038	0.027	0.1517	-0.018	0.032	0.5717	0.022	0.038	0.5633
Bacteria	Bacteroidetes	Flavobacteriia	Flavobacteriales	Weeksellaceae	Elizabethkingia	0.006	0.020	0.7870	-0.011	0.020	0.5827	0.055	0.027	0.0450	-0.030	0.036	0.4025
Bacteria	Bacteroidetes	Flavobacteriia	Flavobacteriales	Weeksellaceae	Empedobacter	-0.015	0.021	0.4809	-0.008	0.022	0.7027	-0.023	0.031	0.4514	0.008	0.045	0.8516
Bacteria	Bacteroidetes	Flavobacteriia	Flavobacteriales	Weeksellaceae	Epilithonimonas	-0.025	0.025	0.3197	-0.015	0.028	0.6093	-0.023	0.034	0.5040	-0.015	0.043	0.7237
Bacteria	Bacteroidetes	Flavobacteriia	Flavobacteriales	Weeksellaceae	Kaistella	-0.001	0.016	0.9607	0.007	0.016	0.6613	0.001	0.024	0.9688	-0.027	0.035	0.4414
Bacteria	Bacteroidetes	Flavobacteriia	Flavobacteriales	Weeksellaceae	Planobacterium	0.014	0.012	0.2560	0.014	0.012	0.2077	0.014	0.028	0.6085	0.010	0.023	0.6536
Bacteria	Bacteroidetes	Flavobacteriia	Flavobacteriales	Weeksellaceae	Weeksella	-0.015	0.010	0.1600	-0.014	0.011	0.1839	0.011	0.024	0.6559	0.039	0.017	0.0237
Bacteria	Bacteroidetes	Sphingobacteriia	Sphingobacteriales	Sphingobacteriaceae	Pedobacter	-0.035	0.028	0.2100	-0.019	0.028	0.4990	-0.048	0.039	0.2180	0.091	0.048	0.0591
Bacteria	Bacteroidetes	Sphingobacteriia	Sphingobacteriales	Sphingobacteriaceae	Sphingobacterium	0.020	0.022	0.3653	0.027	0.022	0.2288	-0.024	0.023	0.2914	0.058	0.034	0.0852
Bacteria	Cyanobacteria		Chroococcidiopsidales	Chroococcidiopsidaceae	Chroococcidiopsis	0.026	0.013	0.0463	0.036	0.013	0.0070	-0.008	0.023	0.7156	0.002	0.019	0.9090
Bacteria	Cyanobacteria		Nostocales	Nostocaceae	Cylindrospermum	0.040	0.032	0.2074	0.038	0.033	0.2450	0.017	0.026	0.5049	-0.034	0.030	0.2602
Bacteria	Cyanobacteria		Nostocales	Nostocaceae	Nostoc	-0.076	0.036	0.0365	-0.060	0.033	0.0664	-0.040	0.042	0.3402	-0.028	0.036	0.4306
Bacteria	Cyanobacteria		Nostocales	Scytonemataceae	Scytonema	-0.035	0.022	0.1067	-0.037	0.018	0.0441	-0.014	0.026	0.5908	0.074	0.037	0.0463
Bacteria	Cyanobacteria		Oscillatoriales	Microcoleaceae	Microcoleus	-0.005	0.029	0.8747	-0.007	0.028	0.8026	0.030	0.039	0.4385	0.028	0.040	0.4752
Bacteria	Cyanobacteria		Oscillatoriales	Oscillatoriaceae	Oscillatoria	0.038	0.025	0.1289	0.053	0.025	0.0333	-0.020	0.035	0.5550	-0.053	0.039	0.1702
Bacteria	Deinococcus-Thermus	Deinococci	Deinococcales	Deinococcaceae	Deinococcus	-0.037	0.019	0.0491	-0.033	0.018	0.0609	0.012	0.026	0.6570	-0.020	0.029	0.4888
Archaea	Euryarchaeota	Halobacteria	Halobacteriales	Halobacteriaceae	Halalkalicoccus	0.018	0.017	0.3055	-0.005	0.018	0.7640	0.037	0.024	0.1160	-0.011	0.022	0.6213
Archaea	Euryarchaeota	Halobacteria	Natrialbales	Natrialbaceae	Haloterrigena	-0.013	0.016	0.4191	-0.018	0.018	0.3261	0.027	0.030	0.3722	0.003	0.024	0.8869
Archaea	Euryarchaeota	Methanobacteria	Methanobacteriales	Methanobacteriaceae	Methanobrevibacter	-0.016	0.029	0.5878	-0.006	0.023	0.7988	-0.012	0.049	0.8145	-0.023	0.046	0.6190
Bacteria	Firmicutes	Bacilli	Bacillales	Bacillaceae	Alkalihalobacillus	0.034	0.021	0.1110	0.039	0.020	0.0564	-0.011	0.021	0.5992	0.066	0.035	0.0567
Bacteria	Firmicutes	Bacilli	Bacillales	Bacillaceae	Bacillus	-0.011	0.022	0.6340	-0.020	0.021	0.3557	0.015	0.026	0.5711	-0.029	0.029	0.3285
Bacteria	Firmicutes	Bacilli	Bacillales	Bacillaceae	Exiguobacterium	0.003	0.019	0.8622	-0.016	0.018	0.3856	0.030	0.022	0.1648	-0.021	0.038	0.5787
Bacteria	Firmicutes	Bacilli	Bacillales	Bacillaceae	Gemella	-0.020	0.033	0.5428	-0.013	0.028	0.6387	0.038	0.048	0.4251	-0.030	0.050	0.5394
Bacteria	Firmicutes	Bacilli	Bacillales	Bacillaceae	Salicibibacter	0.001	0.020	0.9692	-0.001	0.016	0.9546	-0.003	0.036	0.9367	0.098	0.045	0.0298
Bacteria	Firmicutes	Bacilli	Bacillales	Listeriaceae	Brochothrix	-0.024	0.022	0.2633	-0.023	0.018	0.2055	-0.037	0.036	0.3089	0.019	0.043	0.6590
Bacteria	Firmicutes	Bacilli	Bacillales	Paenibacillaceae	Saccharibacillus	0.034	0.018	0.0495	0.030	0.015	0.0470	0.003	0.026	0.9120	-0.034	0.034	0.3123

Bacteria	Firmicutes	Bacilli	Bacillales	Planococcaceae	Exiguobacterium	0.044	0.027	0.1093	0.053	0.032	0.0999	0.006	0.027	0.8150	-0.012	0.049	0.8054
Bacteria	Firmicutes	Bacilli	Bacillales	Planococcaceae	Planococcus	-0.046	0.025	0.0623	-0.050	0.020	0.0141	-0.006	0.036	0.8693	0.070	0.033	0.0354
Bacteria	Firmicutes	Bacilli	Bacillales	Planococcaceae	Planomicrobium	-0.018	0.028	0.5173	-0.018	0.027	0.5108	-0.024	0.034	0.4841	-0.014	0.040	0.7188
Bacteria	Firmicutes	Bacilli	Bacillales	Planococcaceae	Solibacillus	0.008	0.036	0.8300	0.025	0.025	0.3261	-0.015	0.042	0.7187	-0.081	0.023	4.94E-04
Bacteria	Firmicutes	Bacilli	Bacillales	Planococcaceae	Sporosarcina	0.000	0.020	0.9884	-0.010	0.020	0.6021	0.028	0.027	0.2867	0.040	0.034	0.2469
Bacteria	Firmicutes	Bacilli	Bacillales	Staphylococcaceae	Auricoccus	0.006	0.017	0.7339	-0.014	0.016	0.4096	0.052	0.023	0.0225	-0.013	0.027	0.6354
Bacteria	Firmicutes	Bacilli	Bacillales	Staphylococcaceae	Jeotgalicoccus	0.028	0.034	0.4109	0.023	0.031	0.4613	0.036	0.050	0.4705	0.124	0.056	0.0274
Bacteria	Firmicutes	Bacilli	Bacillales	Staphylococcaceae	Macrococcus	0.027	0.018	0.1254	0.016	0.019	0.4114	0.031	0.024	0.1872	-0.031	0.042	0.4558
Bacteria	Firmicutes	Bacilli	Bacillales	Staphylococcaceae	Salinicoccus	0.024	0.020	0.2402	0.009	0.022	0.6815	0.051	0.027	0.0598	-0.025	0.032	0.4279
Bacteria	Firmicutes	Bacilli	Bacillales	Staphylococcaceae	Staphylococcus	-0.044	0.031	0.1530	-0.042	0.031	0.1683	-0.027	0.044	0.5435	-0.061	0.047	0.1977
Bacteria	Firmicutes	Bacilli	Bacillales	Thermoactinomycetaceae	Thermoactinomyces	-0.001	0.015	0.9322	-0.001	0.015	0.9317	0.000	0.020	0.9951	-0.041	0.038	0.2796
Bacteria	Firmicutes	Bacilli	Lactobacillales	Aerococcaceae	Abiotrophia	-0.012	0.025	0.6425	-0.006	0.020	0.7699	-0.006	0.041	0.8819	0.008	0.037	0.8360
Bacteria	Firmicutes	Bacilli	Lactobacillales	Aerococcaceae	Aerococcus	0.044	0.023	0.0576	0.033	0.026	0.1945	0.041	0.030	0.1815	-0.016	0.036	0.6699
Bacteria	Firmicutes	Bacilli	Lactobacillales	Carnobacteriaceae	Carnobacterium	0.072	0.038	0.0582	0.043	0.031	0.1709	0.031	0.053	0.5520	0.013	0.056	0.8227
Bacteria	Firmicutes	Bacilli	Lactobacillales	Carnobacteriaceae	Dolosigranulum	0.029	0.025	0.2480	0.009	0.023	0.6799	0.016	0.036	0.6546	-0.018	0.047	0.7007
Bacteria	Firmicutes	Bacilli	Lactobacillales	Carnobacteriaceae	Jeotgalibaca	0.005	0.032	0.8745	-0.011	0.031	0.7121	0.040	0.039	0.3135	0.067	0.045	0.1333
Bacteria	Firmicutes	Bacilli	Lactobacillales	Enterococcaceae	Enterococcus	-0.024	0.017	0.1673	-0.030	0.019	0.1180	0.061	0.023	0.0080	-0.004	0.023	0.8812
Bacteria	Firmicutes	Bacilli	Lactobacillales	Enterococcaceae	Tetragenococcus	-0.011	0.017	0.5163	-0.006	0.013	0.6690	-0.025	0.024	0.2947	0.008	0.042	0.8505
Bacteria	Firmicutes	Bacilli	Lactobacillales	Lactobacillaceae	Amylolactobacillus	-0.001	0.042	0.9816	-0.015	0.036	0.6768	0.010	0.050	0.8360	0.048	0.062	0.4427
Bacteria	Firmicutes	Bacilli	Lactobacillales	Lactobacillaceae	Lacticaseibacillus	0.023	0.025	0.3387	0.012	0.022	0.5944	0.014	0.037	0.7015	-0.012	0.037	0.7356
Bacteria	Firmicutes	Bacilli	Lactobacillales	Lactobacillaceae	Lactiplantibacillus	0.008	0.020	0.6770	0.018	0.021	0.4049	-0.020	0.031	0.5053	0.030	0.031	0.3350
Bacteria	Firmicutes	Bacilli	Lactobacillales	Lactobacillaceae	Lactobacillus	0.000	0.032	0.9981	0.005	0.034	0.8778	-0.006	0.029	0.8359	0.045	0.037	0.2290
Bacteria	Firmicutes	Bacilli	Lactobacillales	Lactobacillaceae	Latilactobacillus	-0.023	0.025	0.3634	-0.012	0.015	0.4403	-0.031	0.052	0.5526	0.056	0.033	0.0897
Bacteria	Firmicutes	Bacilli	Lactobacillales	Lactobacillaceae	Lentilactobacillus	0.010	0.026	0.6898	0.021	0.025	0.3913	-0.029	0.021	0.1595	0.038	0.029	0.1929
Bacteria	Firmicutes	Bacilli	Lactobacillales	Lactobacillaceae	Levilactobacillus	-0.025	0.019	0.1872	-0.020	0.021	0.3269	-0.004	0.024	0.8836	0.033	0.024	0.1729
Bacteria	Firmicutes	Bacilli	Lactobacillales	Lactobacillaceae	Ligilactobacillus	-0.030	0.023	0.1836	-0.035	0.022	0.1158	0.002	0.037	0.9498	0.010	0.034	0.7632
Bacteria	Firmicutes	Bacilli	Lactobacillales	Lactobacillaceae	Limosilactobacillus	-0.032	0.030	0.2932	0.023	0.033	0.4849	-0.085	0.041	0.0366	-0.087	0.039	0.0266
Bacteria	Firmicutes	Bacilli	Lactobacillales	Lactobacillaceae	Loigolactobacillus	-0.023	0.023	0.3104	-0.005	0.020	0.7958	-0.052	0.031	0.0963	-0.025	0.034	0.4502
Bacteria	Firmicutes	Bacilli	Lactobacillales	Lactobacillaceae	Pediococcus	-0.016	0.022	0.4660	-0.016	0.020	0.4144	0.003	0.025	0.9039	-0.013	0.036	0.7105
Bacteria	Firmicutes	Bacilli	Lactobacillales	Leuconostocaceae	Leuconostoc	0.011	0.026	0.6830	-0.006	0.030	0.8332	0.043	0.029	0.1369	-0.013	0.030	0.6568
Bacteria	Firmicutes	Bacilli	Lactobacillales	Leuconostocaceae	Weissella	0.017	0.026	0.5156	0.013	0.029	0.6417	0.008	0.034	0.8233	0.023	0.040	0.5660
Bacteria	Firmicutes	Bacilli	Lactobacillales	Streptococcaceae	Lactococcus	0.045	0.025	0.0663	0.060	0.021	0.0044	0.012	0.032	0.7032	-0.031	0.030	0.2941
Bacteria	Firmicutes	Bacilli	Lactobacillales	Streptococcaceae	Streptococcus	-0.047	0.032	0.1436	-0.023	0.026	0.3871	-0.144	0.057	0.0111	0.074	0.083	0.3761
Bacteria	Firmicutes	Clostridia	Clostridiales	Clostridiaceae	Clostridium	-0.030	0.037	0.4164	0.016	0.026	0.5485	-0.074	0.050	0.1369	-0.022	0.041	0.5966
Bacteria	Firmicutes	Clostridia	Clostridiales	Hungateiclostridiaceae	Fastidiosipila	-0.016	0.030	0.5963	0.020	0.044	0.6489	-0.071	0.046	0.1288	0.000	0.044	0.9982
Bacteria	Firmicutes	Clostridia	Clostridiales	Lachnospiraceae	Anaerobutyricum	-0.072	0.036	0.0455	-0.078	0.028	0.0061	0.015	0.057	0.7943	-0.030	0.048	0.5364
Bacteria	Firmicutes	Clostridia	Clostridiales	Lachnospiraceae	Anaerostipes	-0.006	0.032	0.8517	-0.016	0.026	0.5277	-0.002	0.056	0.9740	0.058	0.042	0.1733
Bacteria	Firmicutes	Clostridia	Clostridiales	Lachnospiraceae	Blautia	0.091	0.068	0.1799	0.139	0.067	0.0383	0.012	0.073	0.8682	0.011	0.081	0.8930
Bacteria	Firmicutes	Clostridia	Clostridiales	Lachnospiraceae	Enterocloster	-0.051	0.034	0.1299	-0.060	0.032	0.0630	0.027	0.040	0.5046	0.042	0.042	0.3064
Bacteria	Firmicutes	Clostridia	Clostridiales	Lachnospiraceae	Lachnoclostridium	-0.064	0.042	0.1293	-0.089	0.042	0.0318	0.003	0.051	0.9563	0.014	0.056	0.7982
Bacteria	Firmicutes	Clostridia	Clostridiales	Lachnospiraceae	Lachnospira	0.020	0.022	0.3567	0.012	0.020	0.5352	-0.011	0.036	0.7641	0.005	0.037	0.9000
Bacteria	Firmicutes	Clostridia	Clostridiales	Lachnospiraceae	Mediterraneibacter	0.024	0.026	0.3570	0.014	0.026	0.5865	0.013	0.034	0.7069	-0.015	0.039	0.7084
Bacteria	Firmicutes	Clostridia	Clostridiales	Lachnospiraceae	Roseburia	-0.026	0.043	0.5413	0.008	0.039	0.8315	-0.093	0.062	0.1354	0.096	0.057	0.0935
Bacteria	Firmicutes	Clostridia	Clostridiales	Oscillospiraceae	Dysosmobacter	0.043	0.045	0.3319	0.032	0.037	0.3887	0.076	0.067	0.2595	0.024	0.065	0.7076
Bacteria	Firmicutes	Clostridia	Clostridiales	Oscillospiraceae	Oscillibacter	-0.016	0.035	0.6524	0.020	0.030	0.4915	-0.074	0.057	0.1937	-0.049	0.052	0.3520
Bacteria	Firmicutes	Clostridia	Clostridiales	Peptostreptococcaceae	Clostridioides	0.036	0.034	0.2974	0.080	0.032	0.0111	-0.064	0.053	0.2229	0.006	0.047	0.9062
Bacteria	Firmicutes	Clostridia	Clostridiales	Peptostreptococcaceae	Flintibacter	0.030	0.024	0.2199	0.059	0.025	0.0203	-0.054	0.040	0.1726	0.034	0.049	0.4938
Bacteria	Firmicutes	Clostridia	Clostridiales	Peptostreptococcaceae	Intestinimonas	0.058	0.038	0.1241	0.055	0.034	0.1053	0.012	0.044	0.7830	-0.006	0.047	0.8936
Bacteria	Firmicutes	Clostridia	Clostridiales	Peptostreptococcaceae	Massilistercora	-0.006	0.022	0.7976	0.001	0.018	0.9702	-0.005	0.044	0.9162	-0.076	0.032	0.0173
Bacteria	Firmicutes	Clostridia	Clostridiales	Peptostreptococcaceae	Monoglobus	0.051	0.022	0.0224	0.037	0.020	0.0595	0.049	0.037	0.1822	-0.059	0.034	0.0870
Bacteria	Firmicutes	Clostridia	Clostridiales	Peptostreptococcaceae	Peptacetobacter	-0.025	0.032	0.4323	-0.035	0.030	0.2435	-0.005	0.042	0.9095	0.045	0.066	0.4965
Bacteria	Firmicutes	Clostridia	Clostridiales	Peptostreptococcaceae	Romboutsia	-0.026	0.033	0.4393	0.011	0.022	0.6108	-0.077	0.055	0.1581	-0.015	0.041	0.7116
Bacteria	Firmicutes	Clostridia	Clostridiales	Ruminococcaceae	Faecalibacterium	0.050	0.046	0.2762	0.016	0.039	0.6790	0.015	0.074	0.8421	-0.143	0.086	0.0982
Bacteria	Firmicutes	Clostridia	Clostridiales	Ruminococcaceae	Flavonifractor	-0.009	0.049	0.8548	-0.037	0.050	0.4575	0.036	0.069	0.6034	-0.159	0.081	0.0509
Bacteria	Firmicutes	Clostridia	Clostridiales	Ruminococcaceae	Ruminococcus	0.021	0.020	0.2915	0.013	0.020	0.5292	0.032	0.030	0.2882	0.059	0.040	0.1421
Bacteria	Firmicutes	Clostridia	Clostridiales	Ruminococcaceae	Ruthenibacterium	0.044	0.031	0.1567	0.034	0.031	0.2771	-0.008	0.044	0.8622	0.005	0.040	0.8968
Bacteria	Firmicutes	Erysipelotrichia	Erysipelotrichales	Erysipelotrichaceae	Amedibacterium	0.026	0.017	0.1331	0.028	0.015	0.0662	0.002	0.034	0.9594	-0.050	0.042	0.2340

Bacteria	Firmicutes	Erysipelotrichia	Erysipelotrichales	Erysipelotrichaceae	Erysipelatoclostridium	-0.075	0.034	0.0286	-0.073	0.030	0.0138	-0.057	0.050	0.2580	0.027	0.047	0.5648
Bacteria	Firmicutes	Erysipelotrichia	Erysipelotrichales	Erysipelotrichaceae	Faecalitalea	0.019	0.029	0.5123	-0.010	0.023	0.6511	0.034	0.054	0.5266	0.011	0.058	0.8558
Bacteria	Firmicutes	Erysipelotrichia	Erysipelotrichales	Erysipelotrichaceae	Longibaculum	-0.022	0.031	0.4876	0.005	0.026	0.8579	-0.073	0.050	0.1430	-0.064	0.049	0.1939
Bacteria	Firmicutes	Erysipelotrichia	Erysipelotrichales	Erysipelotrichaceae	Turicibacter	0.031	0.039	0.4280	-0.017	0.037	0.6467	0.073	0.048	0.1325	-0.017	0.051	0.7332
Bacteria	Firmicutes	Negativicutes	Acidaminococcales	Acidaminococcaceae	Acidaminococcus	-0.043	0.019	0.0221	-0.034	0.015	0.0240	-0.039	0.039	0.3121	0.014	0.054	0.7883
Bacteria	Firmicutes	Negativicutes	Acidaminococcales	Acidaminococcaceae	Phascolarctobacterium	-0.035	0.027	0.1881	-0.048	0.027	0.0744	-0.001	0.039	0.9811	0.040	0.032	0.2120
Bacteria	Firmicutes	Negativicutes	Selenomonadales	Selenomonadaceae	Megamonas	-0.016	0.033	0.6225	-0.016	0.029	0.5870	-0.012	0.053	0.8203	0.027	0.045	0.5554
Bacteria	Firmicutes	Negativicutes	Veillonellales	Veillonellaceae	Megasphaera	0.044	0.025	0.0741	0.010	0.030	0.7374	0.073	0.037	0.0492	-0.005	0.050	0.9140
Bacteria	Firmicutes	Negativicutes	Veillonellales	Veillonellaceae	Veillonella	-0.060	0.038	0.1153	-0.057	0.031	0.0649	-0.063	0.068	0.3489	0.074	0.061	0.2258
Bacteria	Firmicutes	Tissierellia	Tissierellales	Peptoniphilaceae	Anaerococcus	0.101	0.078	0.1956	0.138	0.101	0.1695	-0.034	0.070	0.6245	0.093	0.072	0.1949
Bacteria	Firmicutes	Tissierellia	Tissierellales	Peptoniphilaceae	Ezakiella	-0.018	0.020	0.3605	-0.026	0.015	0.0880	0.021	0.034	0.5332	-0.024	0.046	0.6054
Bacteria	Firmicutes	Tissierellia	Tissierellales	Peptoniphilaceae	Finegoldia	-0.050	0.032	0.1206	-0.056	0.028	0.0442	0.030	0.039	0.4376	0.016	0.048	0.7473
Bacteria	Firmicutes	Tissierellia	Tissierellales	Peptoniphilaceae	Peptoniphilus	0.006	0.033	0.8625	-0.030	0.037	0.4181	0.103	0.065	0.1114	-0.039	0.051	0.4536
Bacteria	Fusobacteria	Fusobacteriia	Fusobacteriales	Fusobacteriaceae	Fusobacterium	-0.006	0.036	0.8620	0.000	0.028	0.9911	-0.036	0.051	0.4859	-0.045	0.053	0.4002
Bacteria	Fusobacteria	Fusobacteriia	Fusobacteriales	Leptotrichiaceae	Leptotrichia	-0.022	0.025	0.3772	-0.013	0.025	0.5914	-0.018	0.047	0.7079	-0.055	0.053	0.3042
Bacteria	Gemmatimonadetes	Gemmatimonadetes	Gemmatimonadales	Gemmatimonadaceae	Gemmatirosa	-0.042	0.032	0.1894	-0.027	0.029	0.3521	-0.011	0.041	0.7876	0.095	0.050	0.0600
Bacteria	Planctomycetes	Planctomycetia	Isosphaerales	Isosphaeraceae	Aquisphaera	-0.027	0.031	0.3735	-0.013	0.029	0.6534	-0.006	0.048	0.9073	0.057	0.049	0.2398
Bacteria	Planctomycetes	Planctomycetia	Isosphaerales	Isosphaeraceae	Tautonia	0.003	0.023	0.9098	-0.001	0.019	0.9448	-0.013	0.036	0.7265	0.050	0.028	0.0730
Bacteria	Planctomycetes	Planctomycetia	Planctomycetales	Planctomycetaceae	Planctomyces	0.013	0.017	0.4255	0.021	0.015	0.1445	-0.006	0.031	0.8436	0.079	0.031	0.0112
Bacteria	Proteobacteria	Alphaproteobacteria	Caulobacterales	Caulobacteraceae	Brevundimonas	-0.044	0.027	0.0979	-0.013	0.025	0.5860	-0.066	0.042	0.1169	0.016	0.048	0.7385
Bacteria	Proteobacteria	Alphaproteobacteria	Caulobacterales	Caulobacteraceae	Caulobacter	0.010	0.022	0.6506	-0.002	0.021	0.9391	0.036	0.032	0.2507	0.026	0.037	0.4854
Bacteria	Proteobacteria	Alphaproteobacteria	Caulobacterales	Caulobacteraceae	Phenylobacterium	-0.014	0.026	0.6040	0.018	0.023	0.4226	-0.070	0.039	0.0704	0.032	0.045	0.4789
Bacteria	Proteobacteria	Alphaproteobacteria	Rhizobiales	Aurantimonadaceae	Aureimonas	-0.014	0.021	0.4895	-0.014	0.020	0.4814	-0.016	0.029	0.5758	-0.053	0.040	0.1851
Bacteria	Proteobacteria	Alphaproteobacteria	Rhizobiales	Bartonellaceae	Bartonella	-0.008	0.016	0.6216	-0.016	0.018	0.3565	0.011	0.012	0.3400	0.021	0.017	0.2172
Bacteria	Proteobacteria	Alphaproteobacteria	Rhizobiales	Bradyrhizobiaceae	Bosea	-0.002	0.026	0.9499	-0.004	0.024	0.8575	0.003	0.032	0.9347	0.008	0.033	0.8136
Bacteria	Proteobacteria	Alphaproteobacteria	Rhizobiales	Bradyrhizobiaceae	Bradyrhizobium	0.009	0.018	0.6263	0.012	0.018	0.5023	0.003	0.020	0.8918	-0.031	0.029	0.2973
Bacteria	Proteobacteria	Alphaproteobacteria	Rhizobiales	Bradyrhizobiaceae	Rhodopseudomonas	-0.020	0.015	0.1775	-0.004	0.015	0.7939	-0.042	0.033	0.2078	0.071	0.034	0.0349
Bacteria	Proteobacteria	Alphaproteobacteria	Rhizobiales	Bradyrhizobiaceae	Tardiphaga	-0.005	0.024	0.8348	-0.001	0.020	0.9516	-0.031	0.035	0.3660	-0.043	0.035	0.2215
Bacteria	Proteobacteria	Alphaproteobacteria	Rhizobiales	Bradyrhizobiaceae	Variibacter	0.018	0.022	0.4205	-0.014	0.018	0.4411	0.091	0.029	0.0018	0.026	0.036	0.4722
Bacteria	Proteobacteria	Alphaproteobacteria	Rhizobiales	Brucellaceae	Brucella	0.004	0.020	0.8543	-0.004	0.022	0.8463	0.033	0.018	0.0766	0.022	0.030	0.4629
Bacteria	Proteobacteria	Alphaproteobacteria	Rhizobiales	Brucellaceae	Ochrobactrum	0.033	0.027	0.2239	0.045	0.022	0.0384	-0.002	0.040	0.9669	0.086	0.032	0.0071
Bacteria	Proteobacteria	Alphaproteobacteria	Rhizobiales	Hyphomicrobiaceae	Devosia	0.060	0.028	0.0310	0.061	0.027	0.0229	0.001	0.040	0.9734	0.029	0.044	0.5110
Bacteria	Proteobacteria	Alphaproteobacteria	Rhizobiales	Hyphomicrobiaceae	Rhodoplanes	-0.013	0.021	0.5376	-0.013	0.020	0.5122	0.023	0.030	0.4376	-0.061	0.037	0.0997
Bacteria	Proteobacteria	Alphaproteobacteria	Rhizobiales	Methylobacteriaceae	Methylobacterium	0.002	0.033	0.9490	0.001	0.031	0.9726	0.004	0.044	0.9262	0.020	0.052	0.7059
Bacteria	Proteobacteria	Alphaproteobacteria	Rhizobiales	Methylobacteriaceae	Methylorubrum	0.055	0.019	0.0043	0.056	0.020	0.0060	0.011	0.037	0.7717	-0.018	0.035	0.6130
Bacteria	Proteobacteria	Alphaproteobacteria	Rhizobiales	Methylobacteriaceae	Microvirga	-0.010	0.030	0.7416	-0.003	0.030	0.9132	-0.004	0.038	0.9188	-0.085	0.049	0.0824
Bacteria	Proteobacteria	Alphaproteobacteria	Rhizobiales	Phyllobacteriaceae	Mesorhizobium	-0.006	0.023	0.8115	-0.013	0.023	0.5700	0.013	0.035	0.7068	0.051	0.031	0.1007
Bacteria	Proteobacteria	Alphaproteobacteria	Rhizobiales	Rhizobiaceae	Agrobacterium	0.012	0.022	0.5718	0.011	0.021	0.6243	0.015	0.037	0.6928	0.009	0.042	0.8266
Bacteria	Proteobacteria	Alphaproteobacteria	Rhizobiales	Rhizobiaceae	Ensifer	-0.021	0.020	0.2965	-0.022	0.019	0.2597	-0.009	0.028	0.7442	0.018	0.032	0.5818
Bacteria	Proteobacteria	Alphaproteobacteria	Rhizobiales	Rhizobiaceae	Neorhizobium	0.001	0.019	0.9619	-0.016	0.018	0.3499	0.051	0.030	0.0916	0.030	0.034	0.3808
Bacteria	Proteobacteria	Alphaproteobacteria	Rhizobiales	Rhizobiaceae	Rhizobium	-0.003	0.029	0.9298	-0.012	0.025	0.6248	0.013	0.040	0.7533	0.003	0.042	0.9352
Bacteria	Proteobacteria	Alphaproteobacteria	Rhizobiales	Rhizobiaceae	Shinella	0.000	0.019	0.9815	-0.033	0.017	0.0518	0.076	0.034	0.0234	-0.018	0.036	0.6132
Bacteria	Proteobacteria	Alphaproteobacteria	Rhizobiales	Rhizobiaceae	Sinorhizobium	-0.023	0.022	0.3022	-0.018	0.024	0.4590	-0.012	0.026	0.6439	0.005	0.027	0.8408
Bacteria	Proteobacteria	Alphaproteobacteria	Rhodobacterales	Rhodobacteraceae	Haematobacter	-0.029	0.013	0.0295	-0.036	0.013	0.0073	0.012	0.028	0.6623	0.009	0.029	0.7488
Bacteria	Proteobacteria	Alphaproteobacteria	Rhodobacterales	Rhodobacteraceae	Pannonibacter	0.009	0.022	0.6954	0.021	0.018	0.2372	-0.031	0.043	0.4790	0.075	0.033	0.0227
Bacteria	Proteobacteria	Alphaproteobacteria	Rhodobacterales	Rhodobacteraceae	Paracoccus	0.018	0.029	0.5229	0.038	0.028	0.1810	-0.051	0.039	0.1993	-0.060	0.041	0.1473
Bacteria	Proteobacteria	Alphaproteobacteria	Rhodobacterales	Rhodobacteraceae	Phaeobacter	-0.042	0.028	0.1403	-0.007	0.023	0.7818	-0.071	0.047	0.1302	0.019	0.037	0.6192
Bacteria	Proteobacteria	Alphaproteobacteria	Rhodobacterales	Rhodobacteraceae	Pseudooceanicola	-0.013	0.021	0.5402	0.006	0.016	0.7195	-0.066	0.046	0.1543	0.020	0.053	0.7085
Bacteria	Proteobacteria	Alphaproteobacteria	Rhodobacterales	Rhodobacteraceae	Rhodobacter	0.009	0.019	0.6487	0.015	0.016	0.3652	0.004	0.028	0.8866	0.032	0.035	0.3667
Bacteria	Proteobacteria	Alphaproteobacteria	Rhodobacterales	Rhodobacteraceae	Roseivivax	-0.022	0.026	0.3966	-0.017	0.023	0.4713	-0.023	0.030	0.4410	-0.007	0.033	0.8209
Bacteria	Proteobacteria	Alphaproteobacteria	Rhodobacterales	Rhodobacteraceae	Tabrizicola	-0.012	0.017	0.4846	-0.022	0.016	0.1649	0.068	0.028	0.0140	-0.005	0.029	0.8731
Bacteria	Proteobacteria	Alphaproteobacteria	Rhodospirillales	Acetobacteraceae	Acetobacter	-0.011	0.016	0.4901	-0.018	0.017	0.3123	0.006	0.022	0.7892	0.002	0.034	0.9455
Bacteria	Proteobacteria	Alphaproteobacteria	Rhodospirillales	Acetobacteraceae	Roseococcus	-0.020	0.023	0.3961	-0.006	0.020	0.7808	-0.049	0.036	0.1760	-0.006	0.038	0.8753
Bacteria	Proteobacteria	Alphaproteobacteria	Rhodospirillales	Acetobacteraceae	Roseomonas	0.009	0.019	0.6349	0.027	0.029	0.3468	-0.037	0.042	0.3747	-0.020	0.032	0.5273
Bacteria	Proteobacteria	Alphaproteobacteria	Rhodospirillales	Rhodospirillaceae	Skermanella	0.001	0.027	0.9768	0.003	0.019	0.8833	-0.002	0.026	0.9434	0.009	0.026	0.7305
Bacteria	Proteobacteria	Alphaproteobacteria	Rickettsiales	Anaplasmataceae	Wolbachia	-0.009	0.012	0.4799	-0.018	0.012	0.1386	0.049	0.022	0.0269	0.019	0.028	0.5010
Bacteria	Proteobacteria	Alphaproteobacteria	Rickettsiales	Rickettsiaceae	Rickettsia	0.007	0.026	0.7739	0.011	0.025	0.6514	-0.046	0.039	0.2395	0.021	0.024	0.3794

Bacteria	Proteobacteria	Alphaproteobacteria	Sphingomonadales	Erythrobacteraceae	Altererythrobacter	0.008	0.021	0.6982	0.005	0.020	0.8207	-0.002	0.035	0.9446	-0.019	0.035	0.5939
Bacteria	Proteobacteria	Alphaproteobacteria	Sphingomonadales	Erythrobacteraceae	Erythrobacter	-0.001	0.017	0.9467	0.023	0.015	0.1401	-0.030	0.024	0.2065	-0.041	0.014	0.0035
Bacteria	Proteobacteria	Alphaproteobacteria	Sphingomonadales	Erythrobacteraceae	Qipengyuania	0.004	0.020	0.8250	-0.010	0.017	0.5384	0.042	0.029	0.1382	-0.011	0.035	0.7484
Bacteria	Proteobacteria	Alphaproteobacteria	Sphingomonadales	Sphingomonadaceae	Novosphingobium	0.020	0.027	0.4623	0.022	0.026	0.3977	0.010	0.043	0.8263	0.042	0.045	0.3431
Bacteria	Proteobacteria	Alphaproteobacteria	Sphingomonadales	Sphingomonadaceae	Sphingobium	0.003	0.023	0.9013	0.003	0.021	0.8954	0.003	0.032	0.9346	-0.011	0.031	0.7160
Bacteria	Proteobacteria	Alphaproteobacteria	Sphingomonadales	Sphingomonadaceae	Sphingomonas	0.026	0.032	0.4152	0.029	0.028	0.2958	-0.049	0.050	0.3257	-0.071	0.049	0.1444
Bacteria	Proteobacteria	Alphaproteobacteria	Sphingomonadales	Sphingomonadaceae	Sphingopyxis	-0.001	0.020	0.9446	-0.001	0.019	0.9566	0.002	0.031	0.9586	-0.003	0.030	0.9328
Bacteria	Proteobacteria	Alphaproteobacteria	Sphingomonadales	Sphingomonadaceae	Sphingosinicella	-0.009	0.019	0.6309	-0.019	0.019	0.3091	0.025	0.029	0.3940	-0.015	0.032	0.6304
Bacteria	Proteobacteria	Alphaproteobacteria	Sphingomonadales	Sphingomonadaceae	Sphingosinithalassobacter	-0.049	0.019	0.0095	-0.038	0.017	0.0263	-0.045	0.031	0.1465	0.036	0.037	0.3281
Bacteria	Proteobacteria	Betaproteobacteria	Burkholderiales	Alcaligenaceae	Achromobacter	-0.052	0.019	0.0053	-0.042	0.019	0.0284	-0.053	0.037	0.1520	-0.034	0.052	0.5092
Bacteria	Proteobacteria	Betaproteobacteria	Burkholderiales	Alcaligenaceae	Alcaligenes	-0.006	0.028	0.8187	-0.032	0.026	0.2116	0.069	0.028	0.0123	0.025	0.038	0.5070
Bacteria	Proteobacteria	Betaproteobacteria	Burkholderiales	Alcaligenaceae	Bordetella	-0.018	0.023	0.4229	-0.028	0.023	0.2076	0.023	0.034	0.5078	-0.042	0.038	0.2723
Bacteria	Proteobacteria	Betaproteobacteria	Burkholderiales	Alcaligenaceae	Oligella	0.006	0.011	0.5760	-0.011	0.015	0.4550	0.047	0.038	0.2183	-0.012	0.020	0.5252
Bacteria	Proteobacteria	Betaproteobacteria	Burkholderiales	Alcaligenaceae	Rhizobacter	-0.052	0.018	0.0041	-0.036	0.016	0.0252	-0.032	0.030	0.2786	0.053	0.031	0.0866
Bacteria	Proteobacteria	Betaproteobacteria	Burkholderiales	Alcaligenaceae	Rubrivivax	-0.010	0.020	0.6348	-0.035	0.018	0.0498	0.044	0.028	0.1199	-0.021	0.036	0.5643
Bacteria	Proteobacteria	Betaproteobacteria	Burkholderiales	Alcaligenaceae	Xylophilus	-0.019	0.019	0.3328	-0.019	0.016	0.2335	-0.025	0.031	0.4116	-0.016	0.038	0.6677
Bacteria	Proteobacteria	Betaproteobacteria	Burkholderiales	Burkholderiaceae	Burkholderia	-0.011	0.026	0.6692	0.003	0.016	0.8801	-0.054	0.046	0.2435	0.039	0.032	0.2153
Bacteria	Proteobacteria	Betaproteobacteria	Burkholderiales	Burkholderiaceae	Cupriavidus	0.003	0.028	0.9018	0.011	0.022	0.6243	-0.017	0.040	0.6766	-0.042	0.037	0.2529
Bacteria	Proteobacteria	Betaproteobacteria	Burkholderiales	Burkholderiaceae	Lautropia	-0.010	0.020	0.6359	0.003	0.016	0.8362	-0.057	0.032	0.0725	0.041	0.031	0.1880
Bacteria	Proteobacteria	Betaproteobacteria	Burkholderiales	Burkholderiaceae	Ralstonia	0.027	0.023	0.2357	0.038	0.021	0.0706	-0.018	0.034	0.5902	-0.006	0.035	0.8569
Bacteria	Proteobacteria	Betaproteobacteria	Burkholderiales	Comamonadaceae	Acidovorax	-0.003	0.030	0.9317	-0.016	0.026	0.5540	0.048	0.038	0.2004	-0.031	0.039	0.4254
Bacteria	Proteobacteria	Betaproteobacteria	Burkholderiales	Comamonadaceae	Alicycliphilus	-0.015	0.025	0.5613	-0.031	0.025	0.2211	0.019	0.033	0.5576	0.024	0.038	0.5277
Bacteria	Proteobacteria	Betaproteobacteria	Burkholderiales	Comamonadaceae	Comamonas	0.010	0.027	0.7024	-0.008	0.024	0.7486	0.035	0.028	0.2128	-0.011	0.043	0.8042
Bacteria	Proteobacteria	Betaproteobacteria	Burkholderiales	Comamonadaceae	Delftia	-0.010	0.019	0.5939	-0.019	0.018	0.2814	0.045	0.030	0.1352	-0.028	0.033	0.3872
Bacteria	Proteobacteria	Betaproteobacteria	Burkholderiales	Comamonadaceae	Hydrogenophaga	-0.038	0.023	0.0984	-0.036	0.020	0.0737	-0.026	0.037	0.4863	0.001	0.035	0.9677
Bacteria	Proteobacteria	Betaproteobacteria	Burkholderiales	Comamonadaceae	Mitsuaria	-0.060	0.073	0.4101	-0.074	0.083	0.3709	0.025	0.046	0.5818	-0.142	0.137	0.2997
Bacteria	Proteobacteria	Betaproteobacteria	Burkholderiales	Comamonadaceae	Ottowia	-0.004	0.021	0.8320	-0.016	0.020	0.4231	0.013	0.030	0.6497	-0.005	0.026	0.8412
Bacteria	Proteobacteria	Betaproteobacteria	Burkholderiales	Comamonadaceae	Polaromonas	0.005	0.020	0.8031	0.013	0.020	0.5244	-0.026	0.029	0.3721	0.002	0.036	0.9567
Bacteria	Proteobacteria	Betaproteobacteria	Burkholderiales	Comamonadaceae	Ramlibacter	-0.043	0.028	0.1208	-0.020	0.015	0.1913	-0.047	0.047	0.3230	0.036	0.026	0.1577
Bacteria	Proteobacteria	Betaproteobacteria	Burkholderiales	Comamonadaceae	Rhodoferax	-0.005	0.025	0.8296	0.013	0.024	0.5979	-0.021	0.041	0.6038	-0.037	0.042	0.3796
Bacteria	Proteobacteria	Betaproteobacteria	Burkholderiales	Comamonadaceae	Serpentinomonas	-0.015	0.020	0.4564	-0.008	0.017	0.6523	-0.003	0.029	0.9185	0.010	0.034	0.7695
Bacteria	Proteobacteria	Betaproteobacteria	Burkholderiales	Comamonadaceae	Variovorax	0.007	0.030	0.8121	-0.005	0.031	0.8645	0.002	0.036	0.9496	0.059	0.047	0.2036
Bacteria	Proteobacteria	Betaproteobacteria	Burkholderiales	Oxalobacteraceae	Duganella	-0.032	0.023	0.1625	-0.026	0.022	0.2415	-0.018	0.039	0.6439	-0.073	0.038	0.0552
Bacteria	Proteobacteria	Betaproteobacteria	Burkholderiales	Oxalobacteraceae	Janthinobacterium	0.021	0.028	0.4537	0.026	0.027	0.3339	-0.010	0.038	0.7961	0.045	0.041	0.2726
Bacteria	Proteobacteria	Betaproteobacteria	Burkholderiales	Oxalobacteraceae	Massilia	0.019	0.030	0.5310	0.014	0.027	0.6131	0.000	0.039	0.9916	0.042	0.047	0.3768
Bacteria	Proteobacteria	Betaproteobacteria	Burkholderiales		Methylibium	0.009	0.023	0.6972	0.021	0.021	0.3095	-0.011	0.043	0.8042	0.010	0.032	0.7651
Bacteria	Proteobacteria	Betaproteobacteria	Neisseriales	Neisseriaceae	Conchiformibius	-0.001	0.036	0.9775	-0.006	0.035	0.8583	0.005	0.053	0.9277	0.106	0.074	0.1528
Bacteria	Proteobacteria	Betaproteobacteria	Neisseriales	Neisseriaceae	Kingella	0.012	0.019	0.5364	0.010	0.017	0.5678	0.006	0.033	0.8581	-0.001	0.034	0.9838
Bacteria	Proteobacteria	Betaproteobacteria	Neisseriales	Neisseriaceae	Neisseria	0.014	0.031	0.6620	-0.018	0.027	0.4951	0.078	0.041	0.0592	0.081	0.050	0.1051
Bacteria	Proteobacteria	Betaproteobacteria	Rhodocyclales	Rhodocyclaceae	Aromatoleum	0.022	0.020	0.2762	0.019	0.018	0.2756	0.017	0.029	0.5476	0.055	0.029	0.0613
Bacteria	Proteobacteria	Betaproteobacteria	Rhodocyclales	Zoogloeaceae	Azoarcus	-0.012	0.022	0.5796	-0.008	0.027	0.7602	-0.013	0.024	0.6032	0.024	0.039	0.5416
Bacteria	Proteobacteria	Betaproteobacteria	Rhodocyclales	Zoogloeaceae	Thauera	-0.002	0.027	0.9433	0.002	0.028	0.9480	-0.006	0.017	0.7057	-0.010	0.043	0.8144
Bacteria	Proteobacteria	Deltaproteobacteria	Desulfovibrionales	Desulfovibrionaceae	Desulfovibrio	-0.027	0.023	0.2331	-0.019	0.017	0.2503	-0.046	0.045	0.3050	-0.033	0.050	0.5137
Bacteria	Proteobacteria	Deltaproteobacteria	Myxococcales	Archangiaceae	Archangium	0.011	0.022	0.6287	0.024	0.025	0.3486	-0.025	0.029	0.3810	-0.001	0.048	0.9882
Bacteria	Proteobacteria	Deltaproteobacteria	Myxococcales	Archangiaceae	Cystobacter	0.007	0.027	0.8057	0.008	0.024	0.7383	-0.008	0.034	0.8066	0.018	0.029	0.5418
Bacteria	Proteobacteria	Deltaproteobacteria	Myxococcales	Archangiaceae	Melittangium	-0.014	0.022	0.5408	-0.001	0.017	0.9644	-0.058	0.045	0.2010	0.036	0.050	0.4716
Bacteria	Proteobacteria	Deltaproteobacteria	Myxococcales	Archangiaceae	Stigmatella	-0.032	0.019	0.0928	-0.031	0.016	0.0635	-0.044	0.036	0.2223	0.000	0.036	0.9965
Bacteria	Proteobacteria	Deltaproteobacteria	Myxococcales	Myxococcaceae	Corallococcus	-0.005	0.023	0.8107	-0.008	0.023	0.7195	0.007	0.029	0.8045	0.033	0.031	0.2902
Bacteria	Proteobacteria	Deltaproteobacteria	Myxococcales	Myxococcaceae	Myxococcus	0.022	0.017	0.1937	0.009	0.015	0.5625	0.010	0.028	0.7232	0.045	0.027	0.0993
Bacteria	Proteobacteria	Deltaproteobacteria	Myxococcales	Polyangiaceae	Sorangium	0.005	0.020	0.8022	0.004	0.018	0.8098	0.005	0.030	0.8642	0.038	0.034	0.2625
Bacteria	Proteobacteria	Epsilonproteobacteria	Campylobacterales	Campylobacteraceae	Arcobacter	-0.022	0.018	0.2309	-0.009	0.013	0.4937	-0.030	0.027	0.2691	-0.023	0.020	0.2420
Bacteria	Proteobacteria	Epsilonproteobacteria	Campylobacterales	Campylobacteraceae	Campylobacter	-0.063	0.052	0.2272	-0.065	0.056	0.2452	-0.054	0.060	0.3658	0.028	0.070	0.6907
Bacteria	Proteobacteria	Epsilonproteobacteria	Campylobacterales	Helicobacteraceae	Helicobacter	0.004	0.020	0.8352	0.020	0.023	0.3943	-0.047	0.025	0.0606	0.011	0.050	0.8213
Bacteria	Proteobacteria	Gammaproteobacteria	Aeromonadales	Aeromonadaceae	Aeromonas	-0.019	0.019	0.3247	-0.022	0.018	0.2129	0.014	0.026	0.5966	0.002	0.039	0.9570
Bacteria	Proteobacteria	Gammaproteobacteria	Alteromonadales	Shewanellaceae	Shewanella	0.031	0.012	0.0075	0.016	0.013	0.2052	0.037	0.014	0.0059	0.011	0.035	0.7523
Bacteria	Proteobacteria	Gammaproteobacteria	Cellvibrionales	Cellvibrionaceae	Cellvibrio	0.010	0.021	0.6386	-0.004	0.016	0.7847	0.027	0.035	0.4353	0.013	0.032	0.6853
Bacteria	Proteobacteria	Gammaproteobacteria	Chromatiales	Chromatiaceae	Rheinheimera	-0.027	0.019	0.1638	-0.006	0.017	0.7082	-0.055	0.034	0.1096	-0.009	0.025	0.7106

Bacteria	Proteobacteria	Gammaproteobacteria	Enterobacterales	Enterobacteriaceae	Atlantibacter	-0.011	0.023	0.6402	-0.004	0.020	0.8405	0.001	0.038	0.9814	-0.042	0.026	0.0986
Bacteria	Proteobacteria	Gammaproteobacteria	Enterobacterales	Enterobacteriaceae	Candidatus_Hamiltonella	0.006	0.018	0.7547	-0.001	0.017	0.9533	0.000	0.031	0.9947	-0.013	0.052	0.7948
Bacteria	Proteobacteria	Gammaproteobacteria	Enterobacterales	Enterobacteriaceae	Cedecea	0.019	0.020	0.3520	0.023	0.022	0.3001	-0.001	0.033	0.9721	0.015	0.037	0.6934
Bacteria	Proteobacteria	Gammaproteobacteria	Enterobacterales	Enterobacteriaceae	Citrobacter	-0.029	0.024	0.2295	-0.007	0.021	0.7555	-0.053	0.037	0.1567	-0.026	0.053	0.6170
Bacteria	Proteobacteria	Gammaproteobacteria	Enterobacterales	Enterobacteriaceae	Enterobacter	-0.008	0.023	0.7174	-0.034	0.021	0.1010	0.061	0.038	0.1132	-0.027	0.038	0.4798
Bacteria	Proteobacteria	Gammaproteobacteria	Enterobacterales	Enterobacteriaceae	Escherichia	0.001	0.035	0.9756	0.006	0.034	0.8641	-0.013	0.044	0.7625	-0.012	0.046	0.7937
Bacteria	Proteobacteria	Gammaproteobacteria	Enterobacterales	Enterobacteriaceae	Klebsiella	0.050	0.035	0.1448	0.071	0.032	0.0257	-0.007	0.043	0.8670	-0.078	0.056	0.1635
Bacteria	Proteobacteria	Gammaproteobacteria	Enterobacterales	Enterobacteriaceae	Kosakonia	0.023	0.023	0.3234	0.023	0.020	0.2390	-0.012	0.026	0.6608	0.014	0.028	0.6171
Bacteria	Proteobacteria	Gammaproteobacteria	Enterobacterales	Enterobacteriaceae	Leclercia	-0.021	0.025	0.3980	-0.045	0.024	0.0683	0.083	0.038	0.0293	-0.050	0.046	0.2736
Bacteria	Proteobacteria	Gammaproteobacteria	Enterobacterales	Enterobacteriaceae	Lelliottia	0.003	0.015	0.8504	-0.021	0.014	0.1227	0.042	0.011	2.69E-04	-0.046	0.014	7.98E-04
Bacteria	Proteobacteria	Gammaproteobacteria	Enterobacterales	Enterobacteriaceae	Pseudescherichia	0.028	0.018	0.1304	0.032	0.017	0.0684	0.005	0.033	0.8735	0.034	0.031	0.2709
Bacteria	Proteobacteria	Gammaproteobacteria	Enterobacterales	Enterobacteriaceae	Raoultella	0.015	0.027	0.5899	0.014	0.027	0.6120	0.042	0.046	0.3622	0.082	0.063	0.1920
Bacteria	Proteobacteria	Gammaproteobacteria	Enterobacterales	Enterobacteriaceae	Salmonella	0.007	0.027	0.7796	0.001	0.022	0.9812	-0.006	0.035	0.8622	-0.028	0.040	0.4741
Bacteria	Proteobacteria	Gammaproteobacteria	Enterobacterales	Erwiniaceae	Buchnera	0.031	0.023	0.1856	0.015	0.027	0.5827	0.036	0.016	0.0262	-0.007	0.024	0.7650
Bacteria	Proteobacteria	Gammaproteobacteria	Enterobacterales	Erwiniaceae	Erwinia	0.001	0.029	0.9698	0.015	0.030	0.6343	-0.041	0.037	0.2694	-0.025	0.043	0.5623
Bacteria	Proteobacteria	Gammaproteobacteria	Enterobacterales	Erwiniaceae	Mixta	0.023	0.020	0.2486	0.008	0.019	0.6652	0.047	0.030	0.1107	-0.031	0.031	0.3171
Bacteria	Proteobacteria	Gammaproteobacteria	Enterobacterales	Erwiniaceae	Pantoea	-0.035	0.030	0.2348	-0.043	0.030	0.1549	-0.007	0.039	0.8482	0.007	0.039	0.8611
Bacteria	Proteobacteria	Gammaproteobacteria	Enterobacterales	Morganellaceae	Morganella	-0.041	0.024	0.0889	-0.033	0.027	0.2220	-0.006	0.029	0.8286	0.019	0.047	0.6816
Bacteria	Proteobacteria	Gammaproteobacteria	Enterobacterales	Morganellaceae	Proteus	0.027	0.024	0.2694	0.004	0.024	0.8674	0.032	0.032	0.3170	0.030	0.031	0.3317
Bacteria	Proteobacteria	Gammaproteobacteria	Enterobacterales	Morganellaceae	Providencia	-0.025	0.028	0.3638	-0.018	0.028	0.5273	-0.002	0.032	0.9574	-0.064	0.043	0.1352
Bacteria	Proteobacteria	Gammaproteobacteria	Enterobacterales	Pectobacteriaceae	Pectobacterium	-0.014	0.017	0.3961	-0.015	0.015	0.3258	-0.026	0.029	0.3643	0.027	0.025	0.2807
Bacteria	Proteobacteria	Gammaproteobacteria	Enterobacterales	Yersiniaceae	Ewingella	0.034	0.045	0.4600	0.061	0.031	0.0457	-0.039	0.080	0.6316	0.015	0.054	0.7846
Bacteria	Proteobacteria	Gammaproteobacteria	Enterobacterales	Yersiniaceae	Rahnella	0.015	0.028	0.6033	0.000	0.024	0.9955	0.006	0.035	0.8673	0.002	0.047	0.9684
Bacteria	Proteobacteria	Gammaproteobacteria	Enterobacterales	Yersiniaceae	Rouxiella	0.080	0.047	0.0921	0.074	0.045	0.0960	0.023	0.059	0.6973	0.190	0.084	0.0230
Bacteria	Proteobacteria	Gammaproteobacteria	Enterobacterales	Yersiniaceae	Serratia	-0.021	0.034	0.5308	-0.010	0.028	0.7345	-0.047	0.040	0.2397	-0.013	0.040	0.7461
Bacteria	Proteobacteria	Gammaproteobacteria	Oceanospirillales	Alcanivoracaceae	Alcanivorax	-0.023	0.032	0.4849	-0.027	0.028	0.3409	-0.003	0.026	0.9162	0.096	0.023	3.16E-05
Bacteria	Proteobacteria	Gammaproteobacteria	Oceanospirillales	Halomonadaceae	Halomonas	-0.001	0.013	0.9518	-0.009	0.015	0.5610	0.040	0.016	0.0116	0.021	0.013	0.1116
Bacteria	Proteobacteria	Gammaproteobacteria	Pasteurellales	Pasteurellaceae	Aggregatibacter	0.026	0.019	0.1696	0.008	0.017	0.6537	0.051	0.026	0.0515	-0.016	0.039	0.6820
Bacteria	Proteobacteria	Gammaproteobacteria	Pasteurellales	Pasteurellaceae	Frederiksenia	0.023	0.021	0.2703	0.023	0.026	0.3784	0.027	0.046	0.5676	-0.151	0.038	6.36E-05
Bacteria	Proteobacteria	Gammaproteobacteria	Pasteurellales	Pasteurellaceae	Haemophilus	0.071	0.030	0.0199	0.085	0.029	0.0037	-0.014	0.038	0.7198	-0.042	0.051	0.4117
Bacteria	Proteobacteria	Gammaproteobacteria	Pasteurellales	Pasteurellaceae	Pasteurella	-0.005	0.021	0.8272	-0.011	0.019	0.5588	-0.005	0.034	0.8788	-0.037	0.045	0.4137
Bacteria	Proteobacteria	Gammaproteobacteria	Pseudomonadales	Moraxellaceae	Acinetobacter	-0.018	0.036	0.6249	-0.003	0.036	0.9291	-0.002	0.046	0.9592	-0.084	0.050	0.0935
Bacteria	Proteobacteria	Gammaproteobacteria	Pseudomonadales	Moraxellaceae	Moraxella	0.015	0.021	0.4568	-0.013	0.020	0.5251	0.060	0.023	0.0086	0.003	0.027	0.9039
Bacteria	Proteobacteria	Gammaproteobacteria	Pseudomonadales	Moraxellaceae	Psychrobacter	0.003	0.036	0.9329	-0.007	0.033	0.8323	0.049	0.036	0.1706	-0.038	0.037	0.3024
Bacteria	Proteobacteria	Gammaproteobacteria	Pseudomonadales	Pseudomonadaceae	Oblitimonas	-0.014	0.016	0.3858	-0.016	0.015	0.2606	0.006	0.037	0.8699	-0.030	0.043	0.4892
Bacteria	Proteobacteria	Gammaproteobacteria	Pseudomonadales	Pseudomonadaceae	Pseudomonas	-0.074	0.037	0.0474	-0.055	0.033	0.0948	-0.075	0.051	0.1425	-0.015	0.065	0.8137
Bacteria	Proteobacteria	Gammaproteobacteria	Vibrionales	Vibrionaceae	Vibrio	0.010	0.017	0.5415	0.019	0.016	0.2309	-0.032	0.045	0.4866	-0.015	0.048	0.7592
Bacteria	Proteobacteria	Gammaproteobacteria	Xanthomonadales	Rhodanobacteraceae	Luteibacter	-0.012	0.021	0.5573	0.002	0.018	0.9085	-0.041	0.034	0.2340	0.031	0.030	0.3043
Bacteria	Proteobacteria	Gammaproteobacteria	Xanthomonadales	Rhodanobacteraceae	Rhodanobacter	-0.027	0.026	0.3014	-0.017	0.025	0.4963	-0.039	0.032	0.2356	-0.029	0.041	0.4828
Bacteria	Proteobacteria	Gammaproteobacteria	Xanthomonadales	Xanthomonadaceae	Arenimonas	-0.034	0.020	0.0926	-0.018	0.018	0.2930	-0.031	0.031	0.3189	0.045	0.035	0.2031
Bacteria	Proteobacteria	Gammaproteobacteria	Xanthomonadales	Xanthomonadaceae	Luteimonas	0.053	0.030	0.0727	0.048	0.028	0.0876	0.011	0.036	0.7576	-0.033	0.047	0.4793
Bacteria	Proteobacteria	Gammaproteobacteria	Xanthomonadales	Xanthomonadaceae	Lysobacter	-0.012	0.027	0.6608	-0.002	0.027	0.9363	-0.040	0.032	0.2123	0.096	0.038	0.0115
Bacteria	Proteobacteria	Gammaproteobacteria	Xanthomonadales	Xanthomonadaceae	Pseudoxanthomonas	0.005	0.024	0.8193	-0.008	0.020	0.6954	0.023	0.029	0.4346	-0.032	0.034	0.3379
Bacteria	Proteobacteria	Gammaproteobacteria	Xanthomonadales	Xanthomonadaceae	Stenotrophomonas	-0.012	0.022	0.5929	0.006	0.019	0.7304	-0.034	0.031	0.2746	0.115	0.032	2.91E-04
Bacteria	Proteobacteria	Gammaproteobacteria	Xanthomonadales	Xanthomonadaceae	Thermomonas	-0.010	0.021	0.6190	0.000	0.022	0.9984	0.002	0.029	0.9431	0.008	0.033	0.8025
Bacteria	Proteobacteria	Gammaproteobacteria	Xanthomonadales	Xanthomonadaceae	Xanthomonas	-0.017	0.018	0.3679	-0.017	0.018	0.3476	-0.002	0.031	0.9535	0.012	0.033	0.7233
Bacteria	Spirochaetes	Spirochaetia	Spirochaetales	Spirochaetaceae	Treponema	0.025	0.019	0.1894	0.012	0.017	0.4919	0.013	0.025	0.6089	-0.030	0.040	0.4526
Bacteria	Tenericutes	Mollicutes	Mycoplasmatales	Mycoplasmataceae	Mycoplasma	0.005	0.010	0.6328	0.000	0.008	0.9781	-0.001	0.019	0.9764	0.001	0.019	0.9596
Archaea	Thaumarchaeota	Nitrososphaeria	Nitrososphaerales	Nitrososphaeraceae	Candidatus_Nitrosocosmicus	0.025	0.021	0.2333	0.008	0.018	0.6436	0.029	0.031	0.3401	0.049	0.033	0.1351
Bacteria	Verrucomicrobia	Verrucomicrobiae	Verrucomicrobiales	Akkermansiaceae	Akkermansia	-0.022	0.031	0.4789	-0.021	0.028	0.4539	0.014	0.043	0.7365	0.058	0.047	0.2099
